# Novel pyrrolizines bearing 3,4,5-trimethoxyphenyl moiety: design, synthesis, molecular docking, and biological evaluation as potential multi-target cytotoxic agents

**DOI:** 10.1080/14756366.2021.1937618

**Published:** 2021-06-21

**Authors:** Ahmed M. Shawky, Nashwa A. Ibrahim, Ashraf N. Abdalla, Mohammed A. S. Abourehab, Ahmed M. Gouda

**Affiliations:** a Science and Technology Unit (STU), Umm Al-Qura University, Makkah, Saudi Arabia; b Central Laboratory for Micro-analysis, Minia University, Minia, Egypt; c Department of Pharmaceutical Chemistry, Faculty of Pharmacy, Umm Al-Qura University, Makkah, Saudi Arabia; d Medicinal Chemistry Department, Faculty of Pharmacy, Beni-Suef University, Beni-Suef, Egypt; e Department of Pharmacology and Toxicology, Faculty of Pharmacy, Umm Al-Qura University, Makkah, Saudi Arabia; f Department of Pharmacology and Toxicology, Medicinal and Aromatic Plants Research Institute, National Center for Research, Khartoum, Sudan; g Department of Pharmaceutics, Faculty of Pharmacy, Umm Al-Qura University, Makkah, Saudi Arabia; h Department of Pharmaceutics, Faculty of Pharmacy, Minia University, Minia, Egypt

**Keywords:** Pyrrolizine, cytotoxicity, apoptosis, kinase inhibitor, cell cycle

## Abstract

In the present study, two new series of pyrrolizines bearing 3,4,5-trimethoxyphenyl moiety were designed, synthesised, and evaluated for their cytotoxic activity. The benzamide derivatives **16a–e** showed higher cytotoxicity than their corresponding Schiff bases **15a–e**. Compounds **16a**,**b**,**d** also inhibited the growth of MCF-7/ADR cells with IC_50_ in the range of 0.52–6.26 μM. Interestingly, the new compounds were less cytotoxic against normal MRC-5 cells (IC_50_=0.155–17.08 μM). Mechanistic studies revealed the ability of compounds **16a**,**b**,**d** to inhibit tubulin polymerisation and multiple oncogenic kinases. Moreover, compounds **16a**,**b**,**d** induced preG_1_ and G_2_/M cell cycle arrest and early apoptosis in MCF-7 cells. The molecular docking analyses of compounds **16a**,**b**,**d** into the active site in tubulin, CDK-2, and EGFR proteins revealed higher binding affinities compared to the co-crystallised ligands. These preliminary results suggested that compounds **16a**,**b**,**d** could serve as promising lead compounds for the future development of new potent anticancer agents.

**Highlights**
1. Two new series of pyrrolizines bearing 3,4,5-trimethoxyphenyl moieties were synthesized.
2. Compounds **16a**,**b**,**d** displayed the highest cytotoxicity against the three cancer cell lines.
3. Kinase profiling test revealed inhibition of multiple oncogenic kinases by compounds **16a**,**b**,**d**.
4. Compounds **16a**,**b**,**d** exhibited weak to moderate inhibition of tubulin-polymerization.
5. Compounds **16a**,**b**,**d** induced preG_1_ and G_2_/M cell cycle arrest and early apoptosis in MCF-7 cells.
6. Docking studies revealed high binding affinities for compounds **16a**,**b** towards tubulin and CDK-2.

## Graphical Abstract

**Figure:**
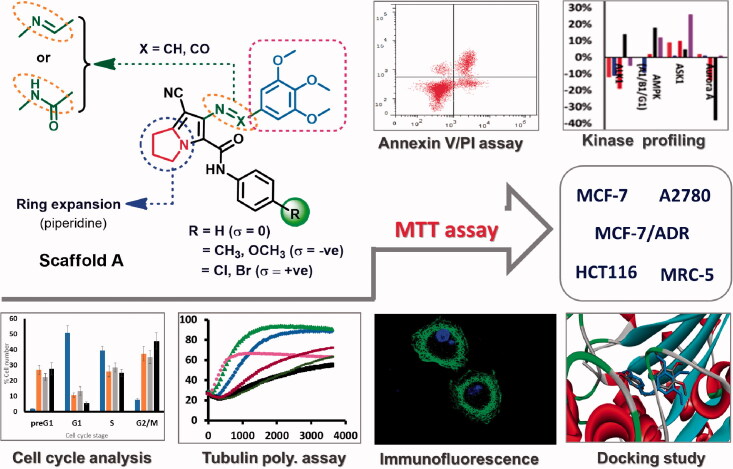

## 1. Introduction

Despite the presence of clinically effective anticancer agents, cancer is still one of the most leading causes of death in the world^1^. In addition, the development of multidrug resistance to many of the currently used anticancer drugs represents another challenge in this field^2^. To overcome these problems, several approaches have been developed and proved better efficacy in cancer treatment. Of these approaches, combination therapy has gained momentum in the treatment of different types of cancers^3^. However, the high cost, toxicity, and high potential for drug–drug interactions have limited the widespread use of combination therapy in the treatment of cancers^3^^,^^4^.

Recently, multi-target anticancer agents also emerged as a new approach to cancer chemotherapy^5–7^. This approach attracted much attention as it could provide a better alternative for combination therapy with lower toxicity and fewer drug–drug interaction problems. Although most of the multi-target agents were discovered by serendipity, several rationally designed multi-target anticancer agents have also been reported^7^. The rational design of these agents was achieved by combining pharmacophoric groups of two or more different anticancer drugs in a single scaffold^5–7^. Accordingly, the resulting multi-pharmacophore scaffold could hit multiple targets, simultaneously.

**Figure 1.**
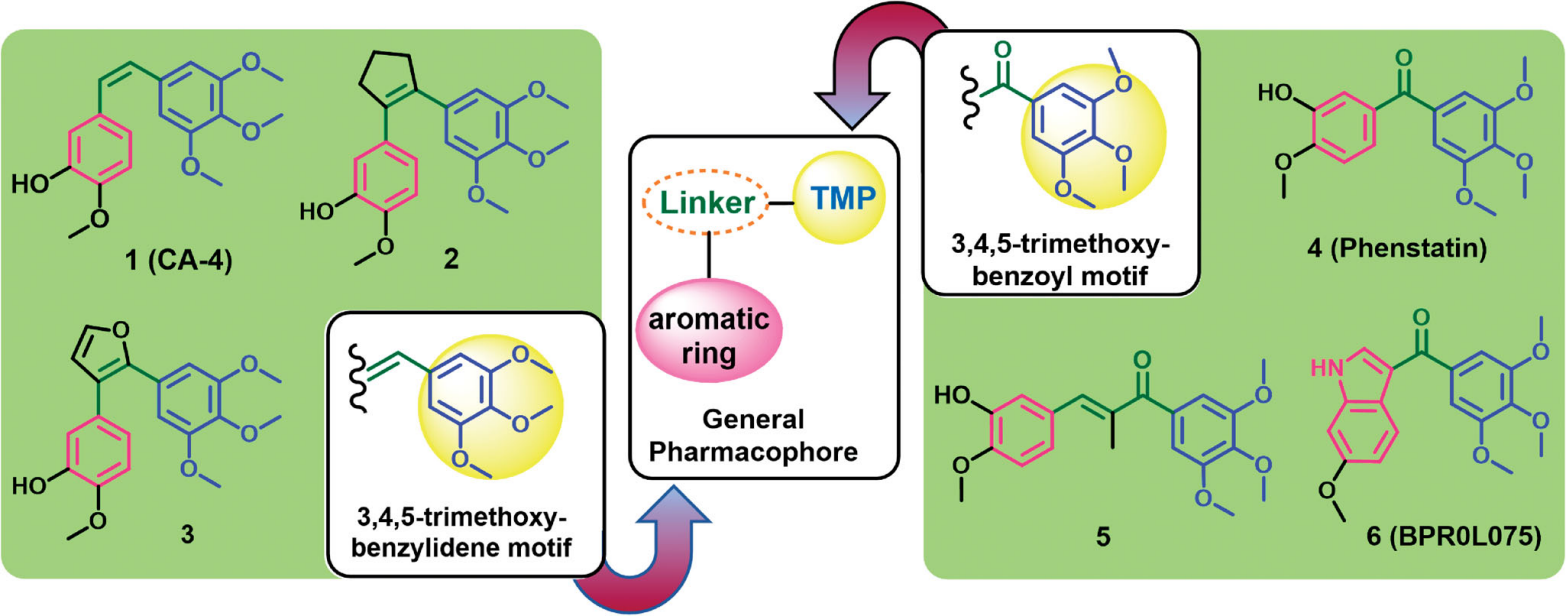
TMP bearing tubulin polymerisation inhibitors **1**–**6** and their pharmacophore features.

Tubulin polymerisation is one of the promising targets in the development of new anticancer agents^8–10^. Combretastatin A-4 (CA-4) **1**, and its analogues **2**, and **3** (Figure 1) are examples of tubulin polymerisation inhibitors (TBIs) that exhibited potent anticancer activities. The mechanism of action of these compounds is mediated by their binding to the colchicine binding site in tubulin resulting in inhibition of the polymerisation^8^. In addition, the trimethoxybenzoyl derivatives **4**–**6** (Figure 1) were also reported as TBIs with potent anticancer activities^8–10^. The mechanism of action of compounds **4**–**6** depends also on the inhibition of tubulin assembly resulting in suppression of microtubule formation^8^. Considering the chemical structure of compounds **1**–**6**, it was observed that they all have similar pharmacophoric features which include 3,4,5-trimethoxyphenyl (TMP) moiety attached through a linker of 1–3 atom length to another substituted phenyl ring. A 2D pharmacophore of these TPIs **1**–**6** is generated in Figure 1.

The TMP moiety is considered as a tubulin-binding moiety and plays an essential role in the antitubulin activity of compounds **1**–**6** [9]. In addition, the linker between the two aromatic rings could be an olefinic (ethenyl) group which restricts the free rotation and keep the two aromatic rings in *cis*-conformation. However, this type of restriction is absent in compounds **4**–**6**, where the two phenyl rings can adopt different orientations relative to each other (Figure 1).

**Figure 2.**
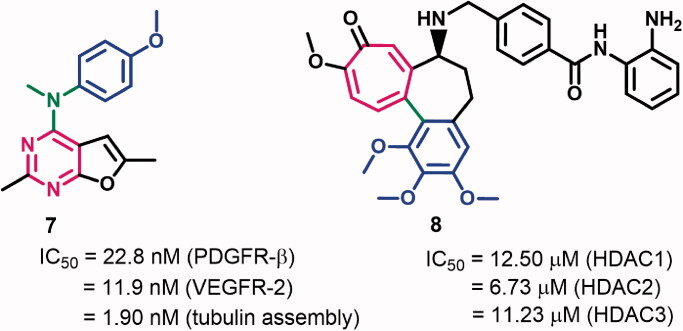
Rationally designed multi-target anticancer agents incorporating tubulin binding moieties.

Incorporation of tubulin-binding moieties such as the TMP moiety into anticancer agents was succeeded as a new strategy to produce multi-target anticancer agents (Figure 2). Compound **7**, a multi-target anticancer agent designed by combining the pharmacophoric groups which can target tubulin polymerisation and tyrosine kinase, simultaneously^11^. Biological evaluation of compound **7** revealed potent inhibitory activity against tubulin polymerisation, vascular endothelial growth factor receptor-2 (VEGFR-2), and platelet-derived growth factor receptor β (PDGFR-β). Compound **7** also displayed superior *in vivo* activity in reducing tumour size and vascularity compared to sunitinib, and docetaxel^11^.

Compound **8**, another multi-target anticancer agent designed by combining the pharmacophoric groups of the TBI (colchicine) with those needed to inhibit HDAC^12^. Compound **8** displayed potent cytotoxic activity (IC_50_=2–105 nM) against a panel of cancer cell lines mediated by moderate inhibition of HDAC and tubulin polymerisation and activity (Figure 2).

### 1.1. Rational design

**Figure 3.**
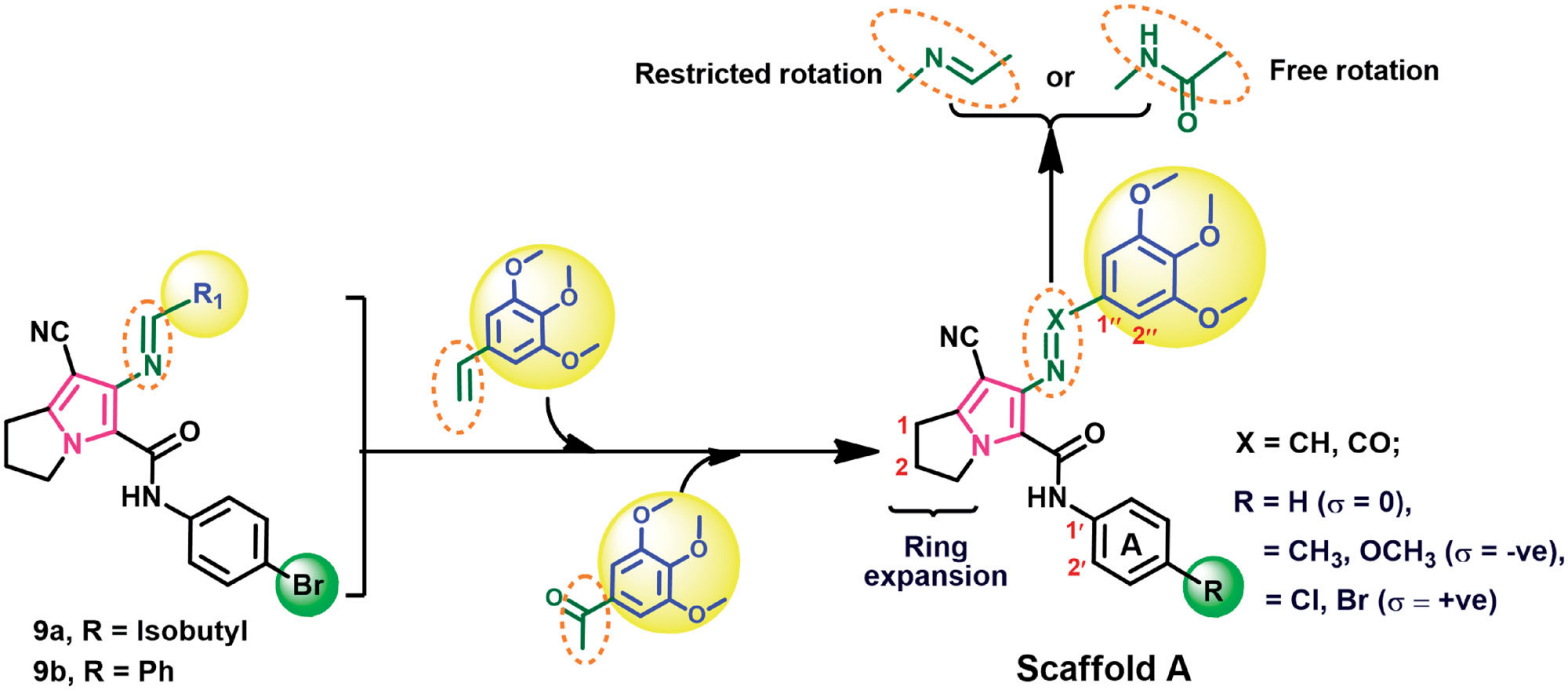
Rational design and structural modifications of scaffold A.

Recently, we reported compounds **9a**,**b** (Figure 3) among a series of pyrrolizine derivatives with cytotoxic activity against MCF-7, A2780, and HT29 cell lines (IC_50_=0.10–4.16 μM)^13^. Mechanistic studies of compounds **9a**,**b** revealed inhibitory activities against COX-2 (IC_50_=13.49 and 1.49 μM, respectively) and/or multiple oncogenic kinases. However, the results of the MTT assay revealed the ability of the two compounds to inhibit the growth of MCF-7 cells at a concentration lower than those required for COX-2 inhibition. In addition, compound **9b** exhibited poor selectivity towards A2780 and HT29 cells (SI = 2.16 and 3.03, respectively). Accordingly, we perform the current study to optimise the cytotoxic potential of compounds **9b** and investigate other potential targets which could contribute to their cytotoxic activities. However, to keep the ability of the new derivatives targeting oncogenic kinases, only small structural modifications in the scaffold of compound **9a** were allowed.

To optimise the cytotoxic potential of the new compounds, scaffold A was designed by replacement of the 3-methylbutylidene/benzylidene moieties in compounds **9a**,**b** by trimethoxy-benzylidene/benzoyl moieties (Figure 3). The new compounds designed bearing these pharmacophoric features will be evaluated for their ability to interfere with the activity of oncogenic kinases and tubulin polymerisation.

The study of structure–activity relationship (SAR) of scaffold A (Figure 3) was achieved through a series of structural modifications which include: (1) variation of the linker between the TMP moiety and pyrrolizine nucleus to restrict/allow the free rotation of the two moieties, (2) expansion of pyrrolidine ring to the six-membered piperidine ring, and (3) variation of the type of substituents (R) on the phenyl ring (A) to include electron-donating/electron-withdrawing groups. A set of 52 derivatives were designed based on these modifications (Supplementary data, Tables S1 and S2).

In addition, a preliminary docking study was performed to evaluate the binding affinities of the designed derivatives into the binding site of colchicine in tubulin protein. The derivatives which displayed high binding scores towards tubulin were also evaluated for their binding affinities towards two of the oncogenic kinases (CDK-2, and EGFR). The selection of these kinases was based on the results of the mechanistic study of compound **9a**^13^. Moreover, the synthetic accessibility and drug-likeness scores of the designed analogues were also evaluated. The results of these studies are provided in Supplementary data (Tables S1 and S2). Based on these results, 10 of the designed derivatives which showed good scores were selected for the synthesis.

## 2. Experimental

### 2.1. Chemistry

All the chemical reagents and solvents used were purchased from Sigma-Aldrich (St. Louis, MO). Solvents were dried according to the literature when necessary. The purity of the new compounds was checked with TLC using the benzene–ethanol mixture (9:1). Melting points (m.p.) are uncorrected and were determined by IA 9100MK-Digital melting point apparatus. BRUKER TENSOR 37 spectrophotometer (Billerica, MA) was used to perform the infra-red (IR) spectra of the new compounds, the spectra were recorded using KBr disc and were expressed in wavenumber (cm^−1^). The proton magnetic spectra were recorded on BRUKER AVANCE III at 500 MHz (Faculty of Pharmacy, Umm Al-Qura University, Mecca, Saudi Arabia) in CDCl_3_/DMSO-d_6_. The *J* constant is given in Hz. The ^13^C NMR spectra of the new compounds in CDCl_3_/DMSO-d_6_ were done at 125 MHz. Mass spectra were recorded on Shimadzu GCMS QP5050A spectrometer (Kyoto, Japan), at 70 eV (EI) at the regional centre for mycology and biotechnology, Al-Azhar University (Cairo, Egypt). Elemental analyses were done in the microanalytical centre, Cairo University (Giza, Egypt). Compounds **11**^14^, **13a–e**^15^^,^^16^, **14a–e**^17^, **18**^15^, and **19**^15^ were prepared according to the previous report^13^. Copies of spectral data including IR, ^1^H NMR, ^13^C NMR, ^13^C NMR, DEPT C^135^, and mass spectra for each of the new compounds are provided in Supplementary (Figs. S1–S141).

For easy identification of different protons/carbons and their chemical shifts, the atoms were numbered as illustrated in scaffold A (Figure 3). The readers should also note that the indolizine nucleus is numbered in a reverse direction.

#### 2.1.1. General procedure (A) for the preparation of compound 15a–e

**Scheme 1.**
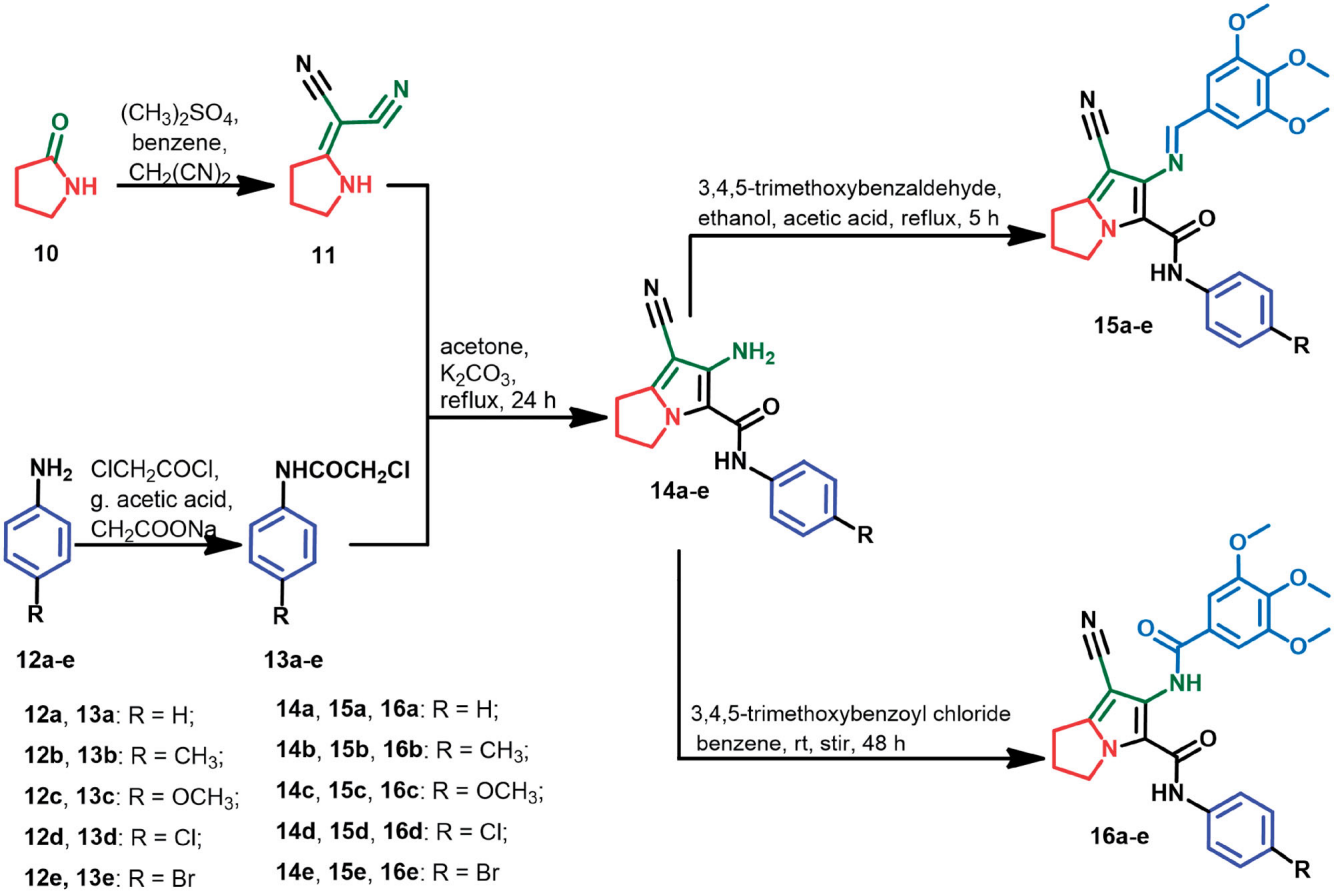
Synthesis of compounds 15a-e and 16a-e.

A mixture of pyrrolizine-5-carboxamides **14a–e** (3 mmol) and 3,4,5-trimethoxybenzaldehyde (0.8 g, 4 mmol), 0.5 mL glacial acetic acid in absolute ethanol (30 mL) was stirred under reflux for 5 h (Scheme 1). The solvent was then evaporated under reduced pressure. The solid obtained was collected and recrystallised from acetone–chloroform (1:1).

##### 2.1.1.1. 7-Cyano-N-phenyl-6-((3,4,5-trimethoxybenzylidene)amino)-2,3-dihydro-1H-pyrrolizine-5-carboxamide (15a)

The title compound was prepared from the reaction of compound **14a** (0.8 g, 3 mmol) with trimethoxy benzoyl chloride (0.8 g, 4 mmol) according to the general procedure A. Compound **15a** was obtained as a yellow amorphous solid product, m.p. 242–4 °C, yield 71%. IR*ʋ*_max_/cm^−1^ 3271, 3236, 3183 (NH), 3076, 3024 (aromatic C–H), 2967, 2941 (aliphatic C–H) 2211 (CN), 1672 (COs), 1609, 1578, 1556 (C═C, C═N), 1432, 1318, 1232 (C–N, C–O, C–C). ^1^H NMR (CDCl_3_, 500 MHz, *δ* ppm): 2.57–2.63 (m, 2H, pyrrolizine CH_2_-2), 3.09 (t, 2H, *J*= 7.5 Hz, pyrrolizine CH_2_-1), 3.97 (s, 6H, 3″-OCH_3_+5″-OCH_3_), 3.98 (s, 3H, 4″-OCH_3_), 4.58 (t, 2H, *J*= 7.1 Hz, pyrrolizine CH_2_-3), 7.13 (t, 1H, *J*= 7.3 Hz, CH-4′), 7.19 (s, 2H, Ph CH-2″+CH-6″), 7.35 (t, 2H, *J*= 7.6 Hz, Ph CH-3′+CH-5′), 7.68 (d, 2H, *J*= 7.8 Hz, Ph CH-2′+CH-6′), 9.11 (s, H, N═CH), 10.68 (s, H, CONH). ^13^C NMR (CDCl_3_, 125 MHz, *δ* ppm): 24.59 (pyrrolizine CH_2_-2), 25.46 (pyrrolizine CH_2_-1), 50.14 (pyrrolizine CH_2_-3), 56.35 (2C, 3″-OCH_3_+5″-OCH_3_), 61.09 (4″-OCH_3_), 84.71 (pyrrolizine C-7), 105.90 (2C, Ph CH-2″+CH-6″), 116.31 (pyrrolizine C-6), 117.71 (CN), 119.79 (2C, Ph CH-2′+CH-6′), 124.12 (Ph CH-4′), 129.04 (2C, Ph CH-3′+CH-5′), 130.75 (Ph C-1″), 138.23 (pyrrolizine C-5), 139.40 (pyrrolizine C-7a), 142.18 (Ph CH-1′), 148.19 (Ph C-4″), 153.82 (2C, Ph C-3″+C-5″), 158.48 (PhNHCO) 159.52 (N═CH). DEPT^135^ (CDCl_3_, 125 MHz, *δ* ppm): *δ* 24.59 (pyrrolizine CH_2_-2), 25.47 (pyrrolizine CH_2_-1), 50.14 (pyrrolizine CH_2_-3), 56.35 (2C, 3″-OCH_3_+5″-OCH_3_), 61.09 (4″-OCH_3_), 105.90 (2C, Ph CH-2″+CH-6″), 119.79 (2C, Ph CH-2′+CH-6′), 124.12 (Ph CH-4′), 129.04 (2C, Ph CH-3′+CH-5′), 159.52 (N═CH). MS (EI): *m/z* (%) 445 ([M + 1]^+^, 4), 444 (M^+^, 12) 443 ([M–1]^+^, 2), 367 (4), 352 (36), 322 (5), 308 (6), 294 (4), 278 (12), 277 (45), 249 (13), 222 (7), 196 (13), 195 (100), 194 (7), 186 (10), 174 (13), 168 (15), 154 (5), 139 (11), 119 (5), 122 (2), 107 (10), 92 (6), 77 (10). Anal. Calcd. for C_25_H_24_N_4_O_4_ (444.48): C, 67.55; H, 5.44; N, 12.60. Found: C, 67.38; H, 4.98; N, 12.69.

##### 2.1.1.2. 7-Cyano-N-(p-tolyl)-6-((3,4,5-trimethoxybenzylidene)amino) -2,3-dihydro-1H-pyrrolizine-5-carboxamide (15b)

The title compound was prepared from the reaction of compound **14b** (0.84 g, 3 mmol) with 3,4,5-trimethoxybenzaldehyde (0.8 g, 4 mmol) according to the general procedure A. Compound **15b** was obtained as a yellow amorphous solid product, m.p. 251–3 °C, yield 78%. IR*ʋ*_max_/cm^−1^ 3275, 3232, 3179 (NH), 3070 (aromatic C–H), 2962, 2926 (aliphatic C–H), 2211 (CN), 1668 (COs), 1611, 1576, 1552 (C═C, C═N), 1459, 1337, 1233 (C–N, C–O, C–C). ^1^H NMR (CDCl_3_, 500 MHz, *δ* ppm): 2.33 (s, 3H, 4′-CH_3_), 2.54–2.60 (m, 2H, pyrrolizine CH_2_-2), 3.06 (t, 2H, *J*= 7.4 Hz, pyrrolizine CH_2_-1), 3.94 (s, 6H, 3′′-OCH_3_+5′′-OCH_3_), 3.95 (s, 3H, 4′′-OCH_3_), 4.54 (t, 2H, *J*= 7.1 Hz, pyrrolizine CH_2_-3), 7.12 (d, 2H, *J*= 7.7 Hz, Ph CH-3′+CH-5′), 7.16 (s, 2H, Ph CH-2″+CH-6″), 7.54 (d, 2H, *J*= 7.6 Hz, Ph CH-2′+CH-6′), 9.08 (s, H, N═CH), 10.58 (s, H, CONH). ^13^C NMR (CDCl_3_, 125 MHz, *δ* ppm): *δ* 20.88 (CH_3_), 24.58 (pyrrolizine CH_2_-2), 25.47 (pyrrolizine CH_2_-1), 50.13 (pyrrolizine CH_2_-3), 56.35 (2C, 3″-OCH_3_+5″-OCH_3_), 61.09 (4″-OCH_3_), 105.87 (2C, Ph CH-2″+CH-6″), 116.36 (pyrrolizine C-7), 117.82 (CN), 119.75 (2C, Ph CH-2′+CH-6′), 129.52 (2C, Ph CH-3′+CH-5′), 130.79 (pyrrolizine C-6), 133.74 (Ph C-1″), 135.67 (pyrrolizine C-5), 139.23 (pyrrolizine C-7a), 142.13 (Ph C-1′), 142.69 (Ph C-4′), 148.08 (Ph C-4″), 153.80 (2C, Ph C-3″+C-5″), 158.39 (PhNHCO), 159.39 (N═CH). DEPT135 (CDCl_3_, 125 MHz, *δ* ppm): *δ* 20.88 (CH_3_), 24.58 (pyrrolizine CH_2_-2), 25.47 (pyrrolizine CH_2_-1), 50.13 (pyrrolizine CH_2_-3), 56.36 (2C, 3″-OCH_3_+5″-OCH_3_), 61.09 (4″-OCH_3_), 105.82 (2C, Ph CH-2″+CH-6″), 119.75 (2C, Ph CH-2′+CH-6′), 129.52 (2C, Ph CH-3′+CH-5′). MS (EI): *m/z* (%) 460 ([M + 2]^+^, 3), 459 ([M + 1]^+^, 19), 458 (M^+^, 72), 457 (7), 429 (4), 353 (21), 352 (100), 336 (4), 322 (7), 308 (7), 292 (11), 291 (54), 280 (2), 278 (3), 263 (8), 229 (4), 196 (3), 195 (21), 194 (2), 186 (9), 168 (9), 154 (3), 106 (2), 91 (2), 77 (4). Anal. Calcd. for C_26_H_26_N_4_O_4_ (458.51): C, 68.11; H, 5.72; N, 12.22. Found: C, 68.53; H, 5.32; N, 12.67.

##### 2.1.1.3. 7-Cyano-N-(4-methoxyphenyl)-6-((3,4,5-trimethoxybenzylidene)amino)-2,3-dihydro-1H-pyrrolizine-5-carboxamide (15c)

The title compound was prepared from the reaction of compound **14c** (0.9 g, 3 mmol) with 3,4,5-trimethoxybenzaldehyde (0.8 g, 4 mmol) according to the general procedure A. Compound **15c** was obtained as a yellow amorphous solid product, m.p. 263–5 °C, yield 74%. IR*ʋ*_max_/cm^−1^ 3281, 3240, 3185 (NH), 3077, 3000 (aromatic C–H), 2942, 2841 (aliphatic C–H) 2208 (CN), 1674 (COs), 1605, 1578, 1511 (C═C, C═N), 1334, 1234 (C–N, C–O). ^1^H NMR (CDCl_3_, 500 MHz, *δ* ppm): *δ* 2.55–2.61 (m, 2H, pyrrolizine CH_2_-2), 3.06 (t, 2H, *J*= 7.4 Hz, pyrrolizine CH_2_-1), 3.82 (s, 3H, 4′-OCH_3_), 3.95 (s, 6H, 3″-OCH_3_+5″-OCH_3_), 3.99 (s, 3H, 4″-OCH_3_), 4.52 (t, 2H, *J*= 7.1 Hz, pyrrolizine CH_2_-3), 6.88 (d, 2H, *J*= 8.6 Hz, Ph CH-3′+CH-5′), 7.28 (s, 2H, Ph CH-2″+CH-6″), 7.61 (d, 2H, *J*= 8.3 Hz, Ph CH-2′+CH-6′), 8.96 (s, 1H, CONH), 10.70 (s, 1H, CONH). ^13^C NMR (CDCl_3_, 125 MHz, *δ* ppm): *δ* 24.62 (pyrrolizine CH_2_-2), 25.48 (pyrrolizine CH_2_-1), 50.10 (pyrrolizine CH_2_-3), 55.54 (4′-OCH_3_), 56.42 (2C, 3″-OCH_3_+5″-OCH_3_), 61.20 (4″-OCH_3_), 106.45 (2C, Ph CH-2″+CH-6″), 106.72 (pyrrolizine C-7), 114.16 (2C, Ph CH-3′+CH-5′), 114.77 (CN), 116.08 (pyrrolizine C-6), 118.05 (Ph C-1′), 121.49 (2C, Ph CH-2′+CH-6′), 129.60 (Ph C-1″), 131.29 (pyrrolizine C-7a), 148.00 (pyrrolizine C-5), 153.66 (Ph C-4″), 153.71 (2C, Ph C-3″+C-5″), 156.32 (Ph C-4′), 158.06 (CONH), 160.30 (N═CH). DEPT C^135^ (CDCl_3_, 125 MHz, *δ* ppm): *δ* 24.62 (pyrrolizine CH_2_-2), 25.48 (pyrrolizine CH_2_-1), 50.10 (pyrrolizine CH_2_-3), 55.54 (4′-OCH_3_), 56.41 (2C, 3″-OCH_3_+5″-OCH_3_), 61.20 (4″-OCH_3_), 106.42 (2C, Ph CH-2″+CH-6″), 114.16 (2C, Ph CH-3′+CH-5′), 121.48 (2C, Ph CH-2′+CH-6′). MS (EI): *m/z* (%) 474 (M^+^, 23), 449 (8), 422 (5), 396 (34), 383 (43), 361 (58), 332 (100), 320 (37), 261 (28), 190 (11), 120 (22), 93 (8), 69 (9). Anal. Calcd. for C_26_H_26_N_4_O_5_ (474.51): C, 65.81; H, 5.52; N, 11.81. Found: C, 65.44; H, 5.21; N, 12.15.

##### 2.1.1.4. N-(4-Chlorophenyl)-7-cyano-6-((3,4,5-trimethoxybenzylidene)amino)-2,3-dihydro-1H-pyrrolizine-5-carboxamide (15d)

The title compound was prepared from the reaction of compound **14d** (0.9 g, 3 mmol) with 3,4,5-trimethoxybenzaldehyde (0.8 g, 4 mmol) according to the general procedure A. Compound **15d** was obtained as a yellow amorphous solid product, m.p. 271–3 °C, yield 64%. IR*ʋ*_max_/cm^−1^ 3277, 3234, 3177 (NH), 3065 (aromatic C–H), 2996, 2939 (aliphatic C–H) 2208 (CN), 1676 (COs), 1611, 1577, 1547 (C═C, C═N), 1417, 1398, 1291 (C–N, C–O). ^1^H NMR (CDCl_3_, 500 MHz, *δ* ppm): 2.55–2.61 (m, 2H, pyrrolizine CH_2_-2), 3.07 (t, 2H, *J*= 7.4 Hz, pyrrolizine CH_2_-1), 3.94 (s, 6H, 3″-OCH_3_+5″-OCH_3_), 3.96 (s, 3H, 4″-OCH_3_), 4.53 (t, 2H, *J*= 7.1 Hz, pyrrolizine CH_2_-3), 7.15 (s, 2H, Ph CH-2″+CH-6″), 7.28 (d, 2H, *J*= 7.8 Hz, Ph CH-3′+CH-5′), 7.60 (d, 2H, *J*= 7.8 Hz, Ph CH-2′+CH-6′), 9.08 (s, H, N═CH), 10.67 (s, H, CONH). ^13^C NMR (CDCl_3_, 125 MHz, *δ* ppm): *δ* 24.60 (pyrrolizine CH_2_-2), 25.45 (pyrrolizine CH_2_-1), 50.14 (pyrrolizine CH_2_-3), 56.37 (2C, 3″-OCH_3_+5″-OCH_3_), 61.11 (4″-OCH_3_), 105.95 (2C, Ph CH-2″+CH-6″), 116.18 (pyrrolizine C-7), 117.42 (CN), 120.90 (2C, Ph CH-2′+CH-6′), 126.45 (pyrrolizine C-6), 128.98 (Ph C-1″), 129.05 (2C, Ph CH-3′+CH-5′), 130.65 (Ph C-4′), 138.87 (pyrrolizine C-5), 139.60 (pyrrolizine C-7a), 142.34 (Ph C-1′), 148.37 (Ph C-4″), 153.85 (2C, Ph C-3″+C-5″), 158.46 (PhNHCO), 159.75 (N═CH). DEPT135 (CDCl_3_, 125 MHz, *δ* ppm): *δ* 24.61 (pyrrolizine CH_2_-2), 25.45 (pyrrolizine CH_2_-1), 50.14 (pyrrolizine CH_2_-3), 56.37 (2C, 3″-OCH_3_+5″-OCH_3_), 61.12 (4″-OCH_3_), 105.95 (2C, Ph CH-2″+CH-6″), 120.90 (2C, Ph CH-2′+CH-6′), 129.05 (2C, Ph CH-3′+CH-5′), 159.75 (N═CH). MS (EI): *m/z* (%) 480 ([M + 2]^+^, 1), 479 ([M + 1]^+^, 1), 478 (M^+^, 4), 458 (7), 353 (13), 352 (73), 323 (4), 322 (8), 311 (11), 308 (10), 292 (12), 291 (44), 278 (8), 263 (13), 252 (9), 229 (8), 222 (11), 196 (15), 195 (100), 186 (20), 168 (28), 154 (12), 147 (6), 111 (4), 106 (5), 91 (7), 77 (12). Anal. Calcd. for C_25_H_23_ClN_4_O_4_ (478.93): C, 62.70; H, 4.84; N, 11.70. Found: C, 63.14; H, 5.07; N, 11.22.

##### 2.1.1.5. N-(4-Bromophenyl)-7-cyano-6-((3,4,5-trimethoxybenzylidene)amino)-2,3-dihydro-1H-pyrrolizine-5-carboxamide (15e)

The title compound was prepared from the reaction of compound **14e** (1.04 g, 3 mmol) with 3,4,5-trimethoxybenzaldehyde (0.8 g, 4 mmol) according to the general procedure A. Compound **15e** was obtained as a yellow amorphous solid product, m.p. 277–80 °C, yield 68%. IR*ʋ*_max_/cm^−1^ 3172 (NH), 3063 (aromatic C–H), 2961, 2939, 2838 (aliphatic C–H) 2209 (CN), 1679 (COs), 1610, 1578, 1545 (C═C, C═N), 1418, 1334, 1232 (C–N, C–O, C–C). ^1^H NMR (CDCl_3_, 500 MHz, *δ* ppm): *δ* 2.57–2.62 (m, 2H, pyrrolizine CH_2_-2), 3.08 (t, 2H, *J*= 7.5 Hz, pyrrolizine CH_2_-1), 3.96 (s, 6H, 3″-OCH_3_+5″-OCH_3_), 3.99 (s, 3H, 4″-OCH_3_), 4.52 (t, 2H, *J*= 6.9 Hz, pyrrolizine CH_2_-3), 7.21 (s, 2H, Ph CH-2″+CH-6″), 7.44 (d, 2H, *J*= 7.7 Hz, Ph CH-3′+CH-5′), 7.59 (d, 2H, *J*= 8.2 Hz, Ph CH-2′+CH-6′), 9.01 (s, 1H, CONH), 10.77 (s, 1H, CONH). ^13^C NMR (CDCl_3_, 125 MHz, *δ* ppm): *δ* 24.64 (pyrrolizine CH_2_-2), 25.45 (pyrrolizine CH_2_-1), 50.15 (pyrrolizine CH_2_-3), 56.42 (2C, 3″-OCH_3_+5″-OCH_3_), 61.20 (4″-OCH_3_), 106.22 (2C, Ph CH-2″+CH-6″), 106.72 (pyrrolizine C-7), 116.55 (CN), 117.54 (pyrrolizine C-6), 121.28 (2C, Ph CH-2′+CH-6′), 130.46 (Ph C-4′), 131.73 (Ph C-1″), 131.97 (2C, Ph CH-3′+CH-5′), 132.62 (pyrrolizine C-5), 137.34 (pyrrolizine C-7a), 148.41 (Ph C-1′), 153.67 (Ph C-4″), 153.78 (2C, Ph C-3″+C-5″), 158.31 (PhNHCO), 160.29 (N═CH). DEPT C^135^ (CDCl_3_, 125 MHz, *δ* ppm): *δ* 24.64 (pyrrolizine CH_2_-2), 25.44 (pyrrolizine CH_2_-1), 50.15 (pyrrolizine CH_2_-3), 56.40 (2C, 3″-OCH_3_+5″-OCH_3_), 61.20 (4″-OCH_3_), 106.18 (2C, Ph CH-2″+CH-6″), 121.25 (2C, Ph CH-2′+CH-6′), 131.97 (2C, Ph CH-3′+CH-5′). MS (EI): *m/z* (%) 525 ([M + 3]^+^, 6), 524 ([M + 2]^+^, 21), 523 ([M + 1]^+^, 8), 522 (M^+^, 14), 368 (28), 352 (100), 313 (22), 236 (13), 195 (17), 109 (16), 97 (26), 69 (35). Anal. Calcd. for C_25_H_23_BrN_4_O_4_ (523.38): C, 57.37; H, 4.43; N, 10.70. Found: C, 56.93; H, 4.57; N, 11.16.

#### 2.1.2. General procedure (B) for the preparation of compounds 16a–e

A mixture of pyrrolizine-5-carboxamides **14a–e** (3 mmol) and 3,4,5-trimethoxybenzoyl chloride (1 g, 4.4 mmol) in dry benzene (30 mL) was stirred for 48 h at room temperature (Scheme 1). The reaction mixture was filtered, set a side. The formed precipitate was recrystallised from ethanol–acetone (1:1).

##### 2.1.2.1. 7-Cyano-N-phenyl-6-(3,4,5-trimethoxybenzamido)-2,3-dihydro-1H-pyrrolizine-5-carboxamide (16a)

The title compound was prepared from the reaction of compound **14a** (0.8 g, 3 mmol) with 3,4,5-trimethoxy benzoyl chloride (1 g, 4.4 mmol) according to the general procedure B. Compound **16a** was obtained as a white solid product, m.p. 253–5 °C, yield 63%. IR*ʋ*_max_/cm^−1^ 3190, 3131 (NHs), 3064, 3014 (aromatic C–H), 2968, 2909 (aliphatic C–H) 2224 (CN), 1656, 1639 (COs), 1584, 1567 (C═C, C═N), 1465, 1338, 1297 (C–N, C–O). ^1^H NMR (DMSO-d_6_, 500 MHz, *δ* ppm): 2.47–2.51 (m, 2H, pyrrolizine CH_2_-2), 3.04 (t, 2H, *J*= 7.4 Hz, pyrrolizine CH_2_-1), 3.73 (s, 3H, 4″-OCH_3_), 3.84 (s, 6H, 3″-OCH_3_+5″-OCH_3_), 4.32 (t, 2H, *J*= 7.2 Hz, pyrrolizine CH_2_-3), 7.06 (t, H, *J*= 7.4 Hz, Ph CH-4′), 7.31 (t, 2H, *J*= 7.9 Hz, Ph CH-3′+CH-5′), 7.35 (s, 2H, Ph CH-2″+CH-6″), 7.58 (d, 2H, *J*= 7.7 Hz, Ph CH-2′+CH-6′), 9.78 (s, H, CONH), 10.34 (s, H, CONH). ^13^C NMR (DMSO-d_6_, 125 MHz, *δ* ppm): *δ* 24.88 (pyrrolizine CH_2_-2), 25.71 (pyrrolizine CH_2_-1), 49.64 (pyrrolizine CH_2_-3), 56.57 (2C, Ph 3″-OCH_3_+5″-OCH_3_), 60.63 (4″-OCH_3_), 84.98 (pyrrolizine C-7), 105.80 (2C, Ph CH-2″+CH-6″), 115.26 (CN), 119.06 (Ph C-1″), 119.75 (2C, Ph CH-2′+CH-6′), 124.16 (Ph CH-4′), 128.80 (pyrrolizine C-5), 128.84 (pyrrolizine C-7a), 129.34 (2C, Ph CH-3′+CH-5′), 139.00 (pyrrolizine C-6), 141.23 (Ph C-1′), 146.44 (Ph C-4″), 153.21 (2C, Ph C-3″+C-5″), 157.92 (PhNHCO), 166.38 (PhCONH). DEPT C^135^ (DMSO-d_6_, 125 MHz, *δ* ppm): *δ* 24.88 (pyrrolizine CH_2_-2), 25.71 (pyrrolizine CH_2_-1), 49.64 (pyrrolizine CH_2_-3), 56.56 (2C, Ph 3″-OCH_3_+5″-OCH_3_), 60.63 (4″-OCH_3_), 105.78 (2C, Ph CH-2″+CH-6″), 119.75 (2C, Ph CH-2′+CH-6′), 124.16 (Ph CH-4′), 129.34 (2C, Ph CH-3′+CH-5′). MS (EI): *m/z* (%) 460 (M^+^, 1), 373 (2), 368 (2), 367 (6), 344 (2), 266 (1), 169 (10), 195 (100), 173 (11), 167 (3), 147 (9), 145 (22), 122 (5), 117 (20), 106 (5), 105 (17), 93 (6), 91 (40), 78 (2). Anal. Calcd. for C_25_H_24_N_4_O_5_ (460.48): C, 65.21; H, 5.25; N, 12.17. Found: C, 65.54; H, 5.17; N, 12.64.

##### 2.1.2.2. 7-Cyano-N-(p-tolyl)-6-(3,4,5-trimethoxybenzamido)-2,3-dihydro-1H-pyrrolizine-5-carboxamide (16b)

The title compound was prepared from the reaction of compound **14b** (0.84 g, 3 mmol) with 3,4,5-trimethoxy benzoyl chloride (1 g, 4.4 mmol) according to the general procedure B. Compound **16b** was obtained as a white solid product, m.p. 265–7 °C, yield 66%. IR*ʋ*_max_/cm^−1^ 3187, 3112 (NHs), 3038 (aromatic C–H), 2968, 2870 (aliphatic C–H) 2221 (CN), 1656, 1638 (COs), 1586, 1549 (C═C, C═N), 1466, 1389, 1263 (C–N, C–O). ^1^H NMR (DMSO-d_6_, 500 MHz, *δ* ppm): 2.23 (s, 3H, Ph-CH_3_), 2.46–2.54 (m, 2H, pyrrolizine CH_2_-2), 3.03 (t, 2H, *J*= 7.3 Hz, pyrrolizine CH_2_-1), 3.74 (s, 3H, 4″-OCH_3_), 3.85 (s, 6H, 3″-OCH_3_+5″-OCH_3_), 4.31 (t, 2H, *J*= 7.0 Hz, pyrrolizine CH_2_-3), 7.10 (d, 2H, *J*= 7.9 Hz, Ph CH-3′+CH-5′), 7.35 (s, 2H, Ph CH-2″+CH-6″), 7.47 (d, 2H, *J*= 7.8 Hz, Ph CH-2′+CH-6′), 9.65 (s, H, CONH), 10.32 (s, H, CONH). ^13^C NMR (DMSO-d_6_, 125 MHz, *δ* ppm): *δ* 20.87 (CH_3_), 24.88 (pyrrolizine CH_2_-2), 25.72 (pyrrolizine CH_2_-1), 49.63 (pyrrolizine CH_2_-3), 56.63 (2C, 3″-OCH_3_+5″-OCH_3_), 60.64 (4″-OCH_3_), 85.07 (pyrrolizine C-7), 105.89 (2C, Ph CH-2″+CH-6″), 115.21 (CN), 119.32 (Ph C-1″), 119.77 (2C, Ph CH-2′+CH-6′), 127.09 (pyrrolizine C-5), 128.70 (pyrrolizine C-7a), 129.69 (2C, Ph CH-3′+CH-5′), 133.23 (Ph C-1′), 136.47 (pyrrolizine C-6), 141.36 (Ph C-4′), 146.32 (Ph C-4″), 153.24 (2C, Ph C-3″+C-5″), 157.69 (PhNHCO), 166.40 (PhCONH). DEPT C^135^ (DMSO-d_6_, 125 MHz, *δ* ppm): *δ* 20.87 (CH_3_), 24.88 (pyrrolizine CH_2_-2), 25.72 (pyrrolizine CH_2_-1), 49.63 (pyrrolizine CH_2_-3), 56.63 (2C, 3″-OCH_3_+5″-OCH_3_), 60.64 (4″-OCH_3_), 105.89 (2C, Ph CH-2″+CH-6″), 119.77 (2C, Ph CH-2′+CH-6′), 129.69 (2C, Ph CH-3′+CH-5′). MS (EI): *m/z* (%) 475 ([M + 1]^+^, 2), 474 (M^+^, 9), 368 (34), 367 (37), 352 (6), 341 (4), 284 (6), 280 (1), 200 (11), 196 (11), 195 (100), 174 (2), 167 (6), 146 (2), 145 (1), 122 (4), 117 (2), 107 (30), 106 (5), 92 (3), 78 (2), 77 (7). Anal. Calcd. for C_26_H_26_N_4_O_5_ (474.51): C, 65.81; H, 5.52; N, 11.81. Found: C, 65.30; H, 5.71; N, 11.99.

##### 2.1.2.3. 7-Cyano-N-(4-methoxyphenyl)-6-(3,4,5-trimethoxybenzamido)-2,3-dihydro-1H-pyrrolizine-5-carboxamide (16c)

The title compound was prepared from the reaction of compound **14c** (0.90 g, 3 mmol) with 3,4,5-trimethoxy benzoyl chloride (1 g, 4.4 mmol) according to the general procedure B. Compound **16c** was obtained as a light buff solid product, m.p. 259–61 °C, yield 69%. IR*ʋ*_max_/cm^−1^ 3196 (NHs), 3068, 3003 (aromatic C–H), 2963, 2941, 2838 (aliphatic C–H) 2223 (CN), 1657, 1634 (COs), 1586, 1511 (C═C, C═N), 1464, 1339, 1234 (C–N, C–O). ^1^H NMR (DMSO-d_6_, 500 MHz, *δ* ppm): *δ* 2.39–2.44 (m, 2H, pyrrolizine CH_2_-2), 2.88 (t, 2H, *J*= 7.0 Hz, pyrrolizine CH_2_-1), 3.71 (s, 3H, 4′-OCH_3_), 3.72 (s, 3H, 4″-OCH_3_), 3.81 (s, 6H, 3″-OCH_3_+5″-OCH_3_), 4.25 (t, 2H, *J*= 7.0 Hz, pyrrolizine CH_2_-3), 6.85 (d, 2H, *J*= 8.8 Hz, Ph CH-3′+CH-5′), 7.45 (s, 2H, Ph CH-2″+CH-6″), 7.52 (d, 2H, *J*= 8.4 Hz, Ph CH-2′+CH-6′), 10.69 (broad s, 2H, two CONHs). ^13^C NMR (DMSO, 125 MHz, *δ* ppm): *δ* 24.68 (pyrrolizine CH_2_-2), 25.56 (pyrrolizine CH_2_-1), 49.47 (pyrrolizine CH_2_-3), 55.66 (4′-OCH_3_), 56.39 (2C, 3″-OCH_3_+5″-OCH_3_), 60.58 (4″-OCH_3_), 84.33 (pyrrolizine C-7), 105.76 (2C, Ph CH-2″+CH-6″), 106.44 (CN), 114.35 (2C, Ph CH-3′+CH-5′), 116.20 (Ph C-1′), 120.89 (2C, Ph CH-2′+CH-6′), 121.46 (Ph C-1″), 132.77 (pyrrolizine C-5), 140.40 (pyrrolizine C-7a), 145.92 (pyrrolizine C-6), 152.32 (Ph C-4″), 152.96 (2C, Ph C-3″+C-5″), 155.57 (Ph C-4′), 158.33 (PhNHCO), 166.33 (PhCONH). DEPT C^135^ (DMSO, 125 MHz, *δ* ppm): *δ* 24.68 (pyrrolizine CH_2_-2), 25.56 (pyrrolizine CH_2_-1), 49.47 (pyrrolizine CH_2_-3), 55.66 (4′-OCH_3_), 56.39 (2C, 3″-OCH_3_+5″-OCH_3_), 60.58 (4″-OCH_3_), 105.75 (2C, Ph CH-2″+CH-6″), 114.35 (2C, Ph CH-3′+CH-5′), 120.89 (2C, Ph CH-2′+CH-6′). MS (EI): *m/z* (%) 492 ([M + 2]^+^, 3), 491 ([M + 1]^+^, 14), 490 (M^+^, 52), 382 (4), 368 (100), 341 (16), 267 (6), 195 (45), 123 (41), 77 (7). Anal. Calcd. for C_26_H_26_N_4_O_6_ (490.51): C, 63.66; H, 5.34; N, 11.42. Found: C, 63.78; H, 5.61; N, 11.69.

##### 2.1.2.4. N-(4-Chlorophenyl)-7-cyano-6-(3,4,5-trimethoxybenzamido) -2,3-dihydro-1H-pyrrolizine-5-carboxamide (16d)

The title compound was prepared from the reaction of compound **14d** (0.9 g, 3 mmol) with 3,4,5-trimethoxy benzoyl chloride (1 g, 4.4 mmol) according to the general procedure B. Compound **16d** was obtained as a white solid product, m.p. 281–3 °C, yield 61%. IR*ʋ*_max_/cm^−1^ 3193, 3108 (NHs), 3062, 3006 (aromatic C–H), 2969, 2937 (aliphatic C–H) 2220 (CN), 1644 (COs), 1586, 1524 (C═C, C═N), 1440, 1296, 1235 (C–N, C–O). ^1^H NMR (DMSO-d_6_, 500 MHz, *δ* ppm): 2.47–2.51 (m, 2H, pyrrolizine CH_2_-2), 3.04 (t, 2H, *J*= 7.4 Hz, pyrrolizine CH_2_-1), 3.73 (s, 3H, 4″-OCH_3_), 3.84 (s, 6H, 3″-OCH_3_+5″-OCH_3_), 4.31 (t, 2H, *J*= 7.1 Hz, pyrrolizine CH_2_-3), 7.33 (s, 2H, Ph CH-2″+CH-6″), 7.37 (d, 2H, *J*= 8.8 Hz, Ph CH-3′+CH-5′), 7.63 (d, 2H, *J*= 8.8 Hz, Ph CH-2′+CH-6′), 9.94 (s, H, CONH), 10.31 (s, H, CONH). ^13^C NMR (DMSO-d_6_, 125 MHz, *δ* ppm): *δ* 24.90 (pyrrolizine CH_2_-2), 25.69 (pyrrolizine CH_2_-1), 49.62 (pyrrolizine CH_2_-3), 56.58 (2C, 3″-OCH_3_+5″-OCH_3_), 60.63 (4″-OCH_3_), 84.98 (pyrrolizine C-7), 105.82 (2C, Ph CH-2″+CH-6″), 115.23 (CN), 118.67 (Ph C-4′), 121.29 (Ph C-1″), 121.41 (2C, Ph CH-2′+CH-6′), 127.70 (pyrrolizine C-5), 128.87 (pyrrolizine C-7a), 129.21 (2C, Ph CH-3′+CH-5′), 138.03 (pyrrolizine C-6), 141.21 (Ph C-1′), 146.58 (Ph C-4″), 153.19 (2C, Ph C-3″+C-5″), 158.05 (PhNHCO), 166.24 (PhCONH). DEPT C^135^ (DMSO-d_6_, 125 MHz, *δ* ppm): *δ* 24.90 (pyrrolizine CH_2_-2), 25.70 (pyrrolizine CH_2_-1), 49.61 (pyrrolizine CH_2_-3), 56.57 (2C, 3″-OCH_3_+5″-OCH_3_), 60.63 (4″-OCH_3_), 105.80 (2C, Ph CH-2″+CH-6″), 121.40 (2C, Ph CH-2′+CH-6′), 129.21 (2C, Ph CH-3′+CH-5′). MS (EI): *m/z* (%) 496 ([M + 2]^+^, 1), 494 (M^+^, 1), 480 (1), 450 (6), 436 (4), 400 (7), 396 (5), 387 (7), 372 (6), 352 (20), 340 (14), 339 (11), 329 (17), 328 (7), 327 (9), 313 (13), 299 (12), 298 (15), 280 (4), 279 (11), 273 (21), 269 (15), 268 (48), 254 (20), 240 (26), 228 (30), 210 (40), 196 (23), 195 (31), 174 (100), 169 (31), 147 (29), 145 (17), 123 (31), 117 (29), 107 (44), 92 (36), 77 (14). Anal. Calcd. for C_25_H_23_ClN_4_O_5_ (494.93): C, 60.67; H, 4.68; N, 11.32. Found: C, 61.13; H, 4.87; N, 11.06.

##### 2.1.2.5. N-(4-Bromophenyl)-7-cyano-6-(3,4,5-trimethoxybenzamido) -2,3-dihydro-1H-pyrrolizine-5-carboxamide (16e)

The title compound was prepared from the reaction of compound **14e** (1.04 g, 3 mmol) with 3,4,5-trimethoxy benzoyl chloride (1 g, 4.4 mmol) according to the general procedure B. Compound **16e** was obtained as a light buff amorphous solid product, m.p. 286–8 °C, yield 64%. IR*ʋ*_max_/cm^−1^ 3222 (NHs), 3060 (aromatic C–H), 2972, 2938, 2835 (aliphatic C–H) 2220 (CN), 1644 (COs), 1587, 1524 (C═C, C═N), 1414, 1388, 1296 (C–N, C–O). ^1^H NMR (DMSO-d_6_, 500 MHz, *δ* ppm): *δ* 2.46–2.51 (m, 2H, pyrrolizine CH_2_-2), 3.04 (t, 2H, *J*= 7.2 Hz, pyrrolizine CH_2_-1), 3.73 (s, 3H, 4″-OCH_3_), 3.83 (s, 6H, 3″-OCH_3_+5″-OCH_3_), 4.30 (t, 2H, *J*= 7.0 Hz, pyrrolizine CH_2_-3), 7.32 (s, 2H, Ph CH-2″+CH-6″), 7.50 (d, 2H, *J*= 8.4 Hz, Ph CH-3′+CH-5′), 7.57 (d, 2H, *J*= 8.6 Hz, Ph CH-2′+CH-6′), 9.91 (s, 1H, CONH), 10.29 (s, 1H, CONH). ^13^C NMR (DMSO, 125 MHz, *δ* ppm): *δ* 24.90 (pyrrolizine CH_2_-2), 25.69 (pyrrolizine CH_2_-1), 49.61 (pyrrolizine CH_2_-3), 56.56 (2C, 3″-OCH_3_+5″-OCH_3_), 60.63 (4″-OCH_3_), 84.86 (pyrrolizine C-7), 105.77 (2C, Ph CH-2″+CH-6″), 115.23 (CN), 115.73 (Ph C-4′), 118.67 (Ph C-1″), 121.76 (2C, Ph CH-2′+CH-6′), 127.62 (pyrrolizine C-5), 128.81 (pyrrolizine C-7a), 132.13 (2C, Ph CH-3′+CH-5′), 138.43 (pyrrolizine C-6), 141.17 (Ph C-1′), 146.61 (Ph C-4″), 153.19 (2C, Ph C-3″+C-5″), 158.04 (PhNHCO), 166.23 (PhCONH). DEPT C^135^ (DMSO, 125 MHz, *δ* ppm): *δ* 24.90 (pyrrolizine CH_2_-2), 25.69 (pyrrolizine CH_2_-1), 49.61 (pyrrolizine CH_2_-3), 56.55 (2C, 3″-OCH_3_+5″-OCH_3_), 60.63 (4″-OCH_3_), 105.75 (2C, Ph CH-2″+CH-6″), 121.75 (2C, Ph CH-2′+CH-6′), 132.13 (2C, Ph CH-3′+CH-5′). MS (EI): *m/z* (%) 543 ([M + 5]^+^, 2), 541 ([M + 3]^+^, 3), 538 (M^+^, 3), 490 (31), 457 (12), 422 (89), 368 (100), 341 (46), 318 (37), 296 (18), 200 (18), 175 (18), 121 (6), 100 (17), 73 (31). Anal. Calcd. for C_25_H_23_BrN_4_O_5_ (539.38): C, 55.67; H, 4.30; N, 10.39. Found: C, 55.83; H, 3.91; N, 10.62.

#### 2.1.3. 1-Cyano-N-phenyl-2-((3,4,5-trimethoxybenzylidene)amino)-5, 6,7,8-tetrahydroindolizine-3-carboxamide (20)

**Scheme 2.**
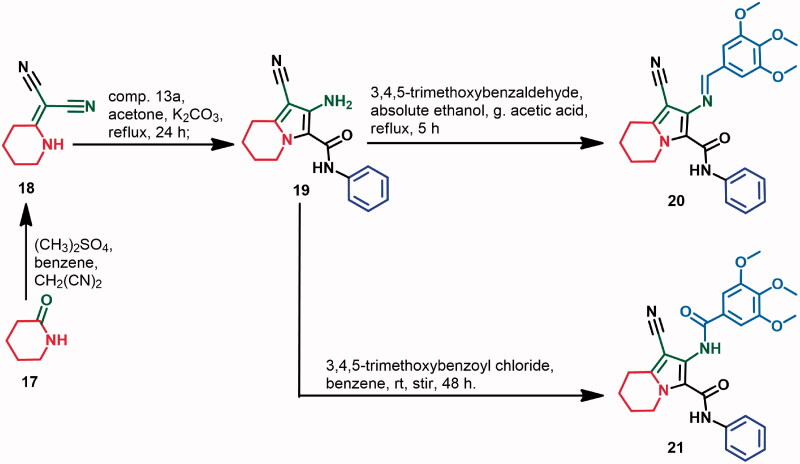
Synthesis of compounds 20 and 21.

A mixture of indolizine-3-carboxamides **19** (0.84 g, 3 mmol) and 3,4,5-trimethoxybenzaldehyde (0.8 g, 4 mmol), 0.5 mL glacial acetic acid in absolute ethanol (30 mL) was stirred under reflux for 5 h (Scheme 2). The solvent was then evaporated under reduced pressure. The solid obtained was collected and recrystallised from acetone–chloroform (1:1). Compound **20** was obtained as a yellow amorphous solid product, m.p. 247–9 °C, yield 73%. IR*ʋ*_max_/cm^−1^ 3182 (NH), 3068 (aromatic C–H), 2938, 2875, 2824 (aliphatic C–H) 2209 (CN), 1677 (COs), 1609, 1594, 1575 (C═C, C═N), 1481, 1330, 1230 (C–N, C–O). ^1^H NMR (CDCl_3_, 500 MHz, *δ* ppm): *δ* 1.91–1.95 (m, 2H, indolizine CH_2_-7), 2.02–2.05 (m, 2H, indolizine CH_2_-6), 2.97 (t, 2H, *J*= 7.3 Hz, indolizine CH_2_-8), 3.95 (s, 6H, 3′-OCH_3_+5′-OCH_3_), 3.98 (s, 3H, 4′-OCH_3_), 4.54 (t, 2H, *J*= 6.5 Hz, indolizine CH_2_-5), 7.12 (t, 1H, *J*= 7.3 Hz, Ph CH-4″), 7.26 (m, 2H, Ph CH-3″+CH-5″), 7.32 (d, 2H, *J*= 7.6 Hz, Ph CH-2″+CH-6″), 7.69 (s, 2H, Ph CH-2′+CH-6′), 8.95 (s, 1H, N═CH), 10.96 (s, 1H, PhNHCO). ^13^C NMR (CDCl_3_, 125 MHz, *δ* ppm): *δ* 18.78 (indolizine CH_2_-7), 22.94 (indolizine CH_2_-8), 23.02 (indolizine CH_2_-6), 46.96 (indolizine CH_2_-5), 56.42 (2C, 3′-OCH_3_+5′-OCH_3_), 61.18 (4′-OCH_3_), 82.19 (indolizine C-1), 106.54 (2C, Ph CH-2′+CH-6′), 106.73 (CN), 115.79 (indolizine C-2), 119.63 (Ph C-1′), 120.06 (2C, Ph CH-2″+CH-6″), 124.10 (Ph C-4″), 128.72 (indolizine C-3), 128.97 (2C, Ph CH-3″+CH-5″), 129.42 (indolizine C-8a), 138.35 (Ph C-1″), 142.92 (Ph C-4′), 153.73 (2C, Ph C-3′+C-5′), 158.80 (N═CH), 161.09 (PhNHCO). DEPT C^135^ (CDCl_3_, 125 MHz, *δ* ppm): *δ* 18.78 (indolizine CH_2_-7), 22.94 (indolizine CH_2_-8), 23.03 (indolizine CH_2_-6), 46.97 (indolizine CH_2_-5), 56.41 (2C, 3′-OCH_3_+5′-OCH_3_), 61.19 (4′-OCH_3_), 106.50 (2C, Ph CH-2′+CH-6′), 120.05 (2C, Ph CH-2′+CH-6″), 124.02 (Ph C-4″), 128.98 (2C, Ph CH-3′+CH-5″). MS (EI): *m/z* (%) 460 ([M + 2]^+^, 5), 458 (M^+^, 8), 422 (83), 396 (30), 382 (30), 356 (47), 332 (100), 296 (15), 106 (13), 89 (26), 77 (12), 69 (31). Anal. Calcd. for C_26_H_26_N_4_O_4_ (458.51): C, 68.11; H, 5.72; N, 12.22. Found: C, 67.88; H, 5.46; N, 12.53.

#### 2.1.4. 1-Cyano-N-phenyl-2-(3,4,5-trimethoxybenzamido)-5,6,7,8-tetrahydroindolizine-3-carboxamide (21)

A mixture of indolizine-3-carboxamides **19** (0.84 g, 3 mmol) and 3,4,5-trimethoxybenzoyl chloride (1 g, 4.4 mmol) in dry benzene (30 mL) was stirred for 48 h at room temperature (Scheme 2). The reaction mixture was filtered, set aside. The precipitate obtained was recrystallised from ethanol–acetone (1:1). Compound **21** was obtained as a white solid product, m.p. 233–5 °C, yield 65%. IR*ʋ*_max_/cm^−1^ 3215, 3187 (NHs), 3061, 3040, 3015 (aromatic C–H), 2960, 2944, 2839 (aliphatic C–H), 2221 (CN), 1648 (COs), 1587, 1566 (C═C, C═N), 1317, 1235 (C–N, C–O). ^1^H NMR (DMSO-d_6_, 500 MHz, *δ* ppm): *δ* 1.72–1.76 (m, 2H, indolizine CH_2_-7), 1.85–1.89 (m, 2H, indolizine CH_2_-6), 2.71 (t, 2H, *J*= 5.7 Hz, indolizine CH_2_-8), 3.71 (s, 3H, 4′-OCH_3_), 3.76 (s, 6H, 3′-OCH_3_+5′-OCH_3_), 4.24 (t, 2H, *J*= 5.6 Hz, indolizine CH_2_-5), 6.97 (d, 2H, *J*= 7.3 Hz, Ph CH-4″), 7.23 (t, 2H, *J*= 7.7 Hz, Ph CH-3″+CH-5″), 7.37 (s, 1H, PhCONH), 7.44 (s, 2H, Ph CH-2′+CH-6′), 7.58 (d, 2H, *J*= 7.9 Hz, Ph CH-2″+CH-6″), 10.92 (broad s, 1H, PhNHCO). ^13^C NMR (DMSO, 125 MHz, *δ* ppm): *δ* 19.15 (indolizine CH_2_-7), 22.75 (indolizine CH_2_-8), 22.86 (indolizine CH_2_-6), 45.90 (indolizine CH_2_-5), 56.21 (2C, 3′-OCH_3_+5′-OCH_3_), 60.56 (4′-OCH_3_), 88.27 (indolizine C-1), 105.69 (2C, Ph CH-2′+CH-6′), 116.52 (CN), 119.25 (2C, Ph CH-2″+CH-6″), 123.00 (Ph C-1′), 125.71 (indolizine C-3), 128.80 (Ph CH-4″), 129.10 (2C, Ph CH-3″+CH-5″), 129.38 (indolizine C-8a), 139.84 (indolizine C-2), 140.12 (Ph C-1″), 140.20 (Ph C-4′), 152.77 (2C, Ph C-3′+C-5′), 159.62 (PhNHCO), 166.29 (PhCONH). DEPT C^135^ (DMSO, 125 MHz, *δ* ppm): *δ* 19.15 (indolizine CH_2_-7), 22.75 (indolizine CH_2_-8), 22.86 (indolizine CH_2_-6), 45.90 (indolizine CH_2_-5), 56.21 (2C, 3′-OCH_3_+5′-OCH_3_), 60.56 (4′-OCH_3_), 105.68 (2C, Ph CH-2′+CH-6′), 119.25 (2C, Ph CH-2″+CH-6″), 128.80 (Ph CH-4″), 129.10 (2C, Ph CH-3″+CH-5″). MS (EI): *m/z* (%) 475 ([M + 1]^+^, 3), 474 (M^+^, 10), 381 (82), 366 (6), 262 (4), 195 (64), 152 (6), 109 (20), 93 (57), 81 (71), 69 (98). Anal. Calcd. for C_26_H_26_N_4_O_5_ (474.51): C, 65.81; H, 5.52; N, 11.81. Found: C, 66.21; H, 5.78; N, 11.66.

### 2.2. Biological evaluation

#### 2.2.1. Evaluation of cytotoxic activity

##### 2.2.1.1. Cell culture

MCF-7 (human breast adenocarcinoma), A2780 (human ovarian cancer) and HCT116 (human colorectal carcinoma), and MRC-5 (normal foetal lung fibroblast) cell lines were bought from the ATCC and were cultured in flasks which were incubated at 37 °C in an atmosphere of 5% CO_2_, 95% air, and 100% relative humidity, to maintain continuous logarithmic growth. MCF-7, A2780, and HCT116 were maintained in RPMI-1640 media (100 U/mL penicillin, 100 µg/mL streptomycin, and 10% foetal bovine serum, Gibco, Carlsbad, CA). MRC-5 cells were maintained in EMEM (100 U/mL penicillin, 100 µg/mL streptomycin, and 10% foetal bovine serum, Gibco, Carlsbad, CA). Doxorubicin (5 μg/mL, for three months) added for RPMI-1640 media containing MCF7 cells was used to develop doxorubicin-resistant MCF7/ADR cells. All cancer and normal cell lines were cultured according to our previous report^13^. Cells were used within 10–20 passages and were checked for mycoplasma every 6 months, by measuring the bioluminescence (Myco Alert sample detection kit; Lonza, Basel, Switzerland) using the multiplate reader (Synergy HT, BioTek, Winooski, VT).

##### 2.2.1.2. MTT cell proliferation assay

The MTT assay was used to measure the cytotoxicity and growth inhibitory activities and cytotoxicity of the new compounds according to our previous report^13^^,^^17^. Cells from flasks of 70–80% confluency were separately seeded in 96-well flat-bottom microculture plates (Nalgene-Nunc, Thermo Fisher Scientific, Roskilde, Denmark) at a density of 3 × 10^3^ cells (MCF-7, A2780, and MRC-5), 1 × 10^4^ cells (HCT116), to a volume of 180 μL/well of culture medium. A Neubauer haemocytometer was used for cell counting. Cells were incubated at 37 °C overnight to allow attachment to the wells. The final concentrations of each compound in wells were: 0–50 μM in 200 μL of media (DMSO 0.1%). Lapatinib (Cayman, Ann Arbor, MI) was used as a positive control at the same final concentrations. Medium only (20 μL) was added to each control well, and each concentration was tested in triplicates (*n* = 3). Following incubation for 72 h, 50 μL MTT was added into each well. Plates were incubated for 3 h, the supernatant was aspirated, and 100 μL of DMSO was added to each well. The optical density (OD) of the purple formazan is proportional to the number of viable cells. When the amount of formazan produced by treated cells is compared with the amount of formazan produced by untreated control cells, the strength of the drug in causing growth inhibition can be determined, through plotting growth curves of absorbance against drug concentration, thus formulation concentration causing 50% inhibition (IC_50_) compared to control cell growth (100%) were determined. GraphPad Prism version 5.00 for Windows was used for analysis (La Jolla, CA). Cytotoxicity of the new compounds was calculated after treatment of cancer/normal cell lines for 72 h. The absorbance of the reduced MTT was determined using a microplate reader A_570_ nm.

#### 2.2.2. Annexin V-FITC/PI apoptosis assay

The ability of compounds **16a**,**b**,**d** to induce apoptotic changes in MCF-7 cells was determined by Annexin V-FITC/PI staining according to the previous report^13^^,^^18^^,^^19^. Additionally, the morphological changes in MCF-7 cells were examined by microscope. Following treatment with the test compounds at 0.1 µM for 24 h, the cells were stained with annexin V and PI. The determination of necrotic, early/late apoptotic changes were performed by flow cytometer (FC500, Beckman Coulter, Miami, FL).

#### 2.2.3. Kinase inhibitory activity

##### 2.2.3.1. Kinase profiling test

Compounds **16a**,**b**,**d** and imatinib were evaluated for their inhibitory activities against 20 kinases at a single concentration (10 μM). The test was performed using the radiolabeled ATP determination method (KINEXUS Bioinformatics Corporation, Vancouver, Canada). The test was performed following the previous reports^13^^,^^20^.

##### 2.2.3.2. CDK-2 inhibitory assay

The ability of compounds **16a**,**b**,**d** to inhibit the activity of CDK-2 was determined using ADP-Glo assay (Promega, Madison, WI). The assay was done according to the manufacturer’s instructions and as described in the previous report^21^.

#### 2.2.4. Cell cycle analysis

Cell cycle perturbations of MCF-7 cells treated with compounds **16a**,**b**,**d** at 0.1 µM concentration for 24 h was determined using FC500 flow cytometer (FC500, Beckman Coulter, Miami, FL). The cell cycle analysis was performed according to our previous report^13^.

#### 2.2.5. Tubulin polymerisation

##### 2.2.5.1. Tubulin polymerisation assay

The effect of compounds **16a**,**b**,**d** on tubulin polymerisation was measured *in vitro* using tubulin polymerisation fluorescence-based assay (cytoskeleton, cat. BK011P, https://www.cytoskeleton.com/bk011p). The assay was performed following the previous report^22^ and according to the manufacturer’s instructions. Briefly, 2 mg/mL porcine tubulin was dissolved in buffer 1 (80 mM PIPES, 2 mM MgCl_2_, 0.5 mM EGTA pH 6.9, 10 µM fluorescent reporter, 1 mM GTP, 15% glycerol) to a final concentration of 10 mg/mL. Then tubulin solution was transferred to a pre-warmed 96-well plate that contained the test compounds **16a**,**b**,**d** (5 μM), paclitaxel (3 μM), CaCl_2_ (0.5 μM), and control buffer. The polymerisation of tubulin was monitored as fluorescence at 37 °C for 60 min. Relative fluorescence units (RFU) were determined at 360 nm for excitation and at 460 nm for emission using the Varioskan Flash spectral scanning multimode reader (Thermo Fisher Scientific, Roskilde, Denmark) at a reading speed of 1 cycle/min.

##### 2.2.5.2. Immunofluorescence staining

The MCF-7 cancer cells plated on coverslips on six-well plate (1 × 10^5^/well) were treated with the indicated concentration of control, colchicine (3 μM), paclitaxel (3 μM), and the test compound **16b** (5 μM) for 24 h as previously reported^23^. After treatment, cells were rinsed twice with PBS, fixed with 3.7% paraformaldehyde, and permeabilised with 0.1% Triton X-100. Cells then were blocked with 1% BSA in PBS for 1 h before further incubation with anti-β-tubulin mouse monoclonal antibody overnight at 4 °C (#86298, Cell Signaling, San Francisco, CA). Cells were incubated with Alexa Fluor^®^ 488 secondary antibodies (Abcam, Cambridge, UK), after being washed with PBS for 1 h in a darkroom. Cellular microtubules were observed with the Nikon Eclipse Ti microscope (Minato-ku, Japan).

### 2.3. Molecular docking

The molecular docking simulation of the new compounds was carried out by AutoDock 4.2^24^. The crystal structure of CDK2 bound to CAN508 (pdb code: 3TNW)^25^, EGFR bound to erlotinib (pdb code: 1M17)^26^, Aurora A bound to VX6 (pdb code: 3E5A)^27^, and tubulin bound to C-A4^28^, were obtained from protein data bank (http://www.rcsb.org/pdb). Ligand and protein files were prepared according to the previous report^13^^,^^29^. Docking and grid parameter files were prepared according to the previous reports^30^^,^^31^. The top 10 protein–ligand complexes were scored. The results including binding modes, affinities, and interactions of the best-fitting conformations of the new compounds were visualised using Discovery Studio Visualizer (v16.1.0.15350, Dassault Systems, San Diego, CA)^32^.

## 3. Results and discussion

### 3.1. Chemistry

In this work, compound **11** was prepared from the reaction of pyrrolidin-2-one **10** and malononitrile as previously reported^13^. On the other hand, the acetanilides **13a–e** were obtained from the reaction of (un)substituted anilines **13a–e** with chloroacetyl chloride according to the reported procedures^13^ (Scheme 1). The starting materials **14a–e** were prepared from the reaction of compounds **11** and **13a–e** in acetone^13^.

The Schiff bases **15a–e** were obtained on refluxing compounds **14a–e** with 3,4,5-trimethoxybenzaldehyde in absolute ethanol in the presence of glacial acetic acid. On the other hand, the benzamide derivatives **16a–e** were prepared from the reaction of compounds **14a–e** with 3,4,5-trimethoxybenzoyl chloride in dry benzene (Scheme 1). Structural elucidation of the new compounds was performed using both spectral and elemental analyses. Copies of spectral data including IR, ^1^H NMR, ^13^C NMR, DEPT C^135^, and mass spectra are provided in supplementary data (Figs. S1–S141).

In addition, compound **18** (**Scheme 2**) was obtained on the treatment of piperidin-2-one **17** with dimethyl sulphate and malononitrile according to the reported procedures^13^. The starting material, indolizine **19** was synthesised using the same reaction conditions adopted for the synthesis of compound **14a** with the replacement of 2-(pyrrolidin-2-ylidene)malononitrile **11** by 2-(piperidin-2-ylidene)malononitrile **18**.

The Schiff base **20** was also obtained from the condensation of 3,4,5-trimethoxybenzaldehyde and 2-aminoindolizine-3-carboxamide **19** in ethanol. On the other hand, acylation of compound **19** with 3,4,5-trimethoxybenzoyl chloride yielded the benzamide derivative **21**. Spectral (IR, H NMR, ^13^C NMR, DEPT C^135^, and mass spectra) and elemental analyses were performed to confirm the chemical structure of compounds **20** and **21**. The spectra of the two compounds are also provided in Supplementary data (Figs. S6, S12, S49–S54, S96–S105, S125, and S141).

### 3.2. Biological evaluation

#### 3.2.1. Cytotoxic activity

##### 3.2.1.1. Evaluation of cytotoxic activity against cancer cell lines

The cytotoxic activity of the new compounds (**15a–e**, **16a–e**, **20**, and **21**) was evaluated *in vitro* using MTT assay^13^. The MTT assay depends on the reduction of the yellow tetrazole dye (MTT) to the purple formazan by the active mitochondrial dehydrogenase enzymes indicating the viability of the cells which can be measured as OD. The new compounds were evaluated against three non-resistant cancer cell lines: MCF-7, and A2780 cell lines were selected to compare cytotoxicity of the new compounds with the previously reported results of compounds **9a**,**b**^13^. Cytotoxicity of the new compounds was also evaluated also against HCT116 cells which lacks COX-2 protein^33^^,^^34^. Selection of this cell line was based on the previous reports which revealed potent cytotoxic activity of the five membered heterocyclic derivatives against HCT116 cells^35^^,^^36^.

The results of the MTT assay (Table 1) revealed variable degrees of cytotoxic activities for the new compounds with IC_50_ values in the range of 0.030–36.880 μM against the three cancer cell lines. The results also revealed higher cytotoxic activities for the benzamide derivatives (**16a–e** and **21**) compared to their corresponding benzylidene analogues (**15a–e** and **20**).

**Table 1.** Cytotoxicity (µM, IC_50_±SD) of compounds **15a–e**, **16a–e**, **20**, and **21** against MCF-7, HCT116, and A2780 cancer cell lines in comparison to compounds **9a**,**b**, lapatinib, and colchicine. 
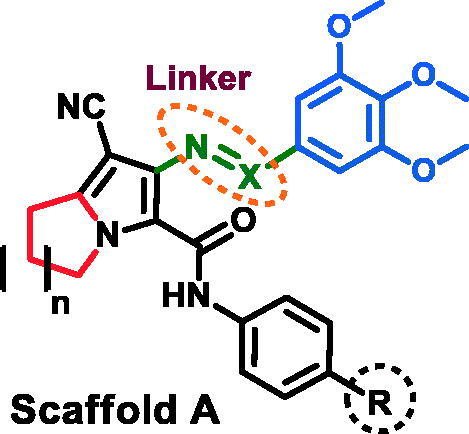

Comp. no.	Linker	*n*	R	IC_50_ (µM ± SD)^a^			Average^b^
				MCF-7	HCT116	A2780	
**15a**	–N═CH–	1	H	36.880 ± 4.400	0.353 ± 0.088	25.760 ± 4.990	20.998
**15b**	–N═CH–	1	CH_3_	35.860 ± 4.440	19.835 ± 7.756	33.360 ± 3.730	29.685
**15c**	–N═CH–	1	OCH_3_	0.616 ± 0.010	0.096 ± 0.015	0.041 ± 0.008	0.251
**15d**	–N═CH–	1	Cl	21.750 ± 3.180	0.534 ± 0.204	0.070 ± 0.002	7.451
**15e**	–N═CH–	1	Br	0.1872 ± 0.098	0.144 ± 0.049	0.311 ± 0.041	0.214
**20**	–N═CH–	2	H	5.721 ± 0.185	0.247 ± 0.069	0.938 ± 0.116	2.302
**16a**	–NHCO–	1	H	0.082 ± 0.002	0.135 ± 0.007	0.044 ± 0.010	0.087
**16b**	–NHCO–	1	CH_3_	0.068 ± 0.009	0.092 ± 0.001	0.180 ± 0.043	0.113
**16c**	–NHCO–	1	OCH_3_	1.470 ± 0.452	0.032 ± 0.014	0.144 ± 0.012	0.549
**16d**	–NHCO–	1	Cl	0.071 ± 0.001	0.097 ± 0.001	0.030 ± 0.005	0.066
**16e**	–NHCO–	1	Br	0.361 ± 0.039	0.173 ± 0.039	0.914 ± 0.092	0.483
**21**	–NHCO–	2	H	0.600 ± 0.054	0.176 ± 0.017	0.598 ± 0.039	0.458
**9a**^c^	–	–	–	0.100 ± 0.050	–	0.520 ± 0.020	0.310
**9b**^c^	–	–	–	0.50 ± 0.15	–	4.16 ± 1.27	2.33
**Lapatinib**	–	–	–	6.690 ± 1.091	10.043 ± 1.125	9.725 ± 0.845	8.833
**Colchicine**^d^	–	–	–	0.012 ± 0.007	0.050 ± 0.005	0.015 ± 0.002	0.026

^a^ IC_50_: concentration of test compound which reduce cellular growth to 50% after 72 h treatment, results represent mean IC_50_ value ± SD (*n* = 3) of three independent experiments.

^b^ Average of the three IC_50_ values against the three cancer cell lines.

^c^ IC_50_ values quoted from our previous publication^13^.

^d^ IC_50_ values quoted from the previous publications^37^^,^^38^.

Compound **16b** was the most potent in inhibiting the growth of MCF-7 cells, while compounds **16c**,**d** exhibited the highest cytotoxic activity against HCT116 and A2780 cells, respectively. On the other hand, lapatinib exhibited cytotoxic activity against the three cancer cell lines with IC_50_ values in the range of 6.690–10.043 μM (Table 1).

The new compounds exhibited cytotoxic activities against MCF-7 cells with IC_50_ values in the range 0.068–36.880 μM. In addition, they inhibited the growth of A2780 cells with values in the range 0.030–33.360 μM. Interestingly, the new compounds also exhibited potent cytotoxic activities against HCT116 cells with IC_50_ values in the range of 00.032–19.835 μM. These results indicated that COXs inhibition may not be essential for the cytotoxicity of the compounds reported in this study.

##### 3.2.1.2. Evaluation of cytotoxic activity against doxorubicin-resistant MCF-7/ADR cells

Encouraged by the high cytotoxic activity of the new compounds against MCF-7 cells (Table 1), three of these derivatives (**16a**,**b**,**d**) were also selected to evaluate their cytotoxicity against doxorubicin-resistant MCF-7/ADR cells. The evaluation was performed using MTT assay^13^. The results are presented in Table 2. The results revealed cytotoxic activity for the three compounds against MCF-7/ADR cells with IC_50_ in the range of 0.52–6.26 μM. Among the three derivatives, compound **16d** with the electron-withdrawing chloro atom exhibited the highest cytotoxic activity against MCF-7/ADR cells, while the methyl analogue **16b** was the least active. These results suggested that electron-withdrawing groups on the phenyl ring of scaffold A could be used to enhance cytotoxic activity against MCF-7/ADR cells.

**Table 2.** Cytotoxicity of compounds **16a**, **b**, **d** against MCF-7/ADR cells.

Comp. no.	IC_50_ (µM ± SD)^a^
**16a**	1.570 ± 0.040
**16b**	6.260 ± 0.710
**16d**	0.520 ± 0.066

^a^ IC_50_: concentration of test compound which reduce cellular growth of MCF-7/ADR cells to 50% after 72 h treatment, results represent mean IC_50_±SD, (*n* = 3) of three independent experiments.

##### 3.2.1.3. Evaluation of cytotoxic selectivity

Selectivity towards cancerous cells is one of the important issues which must be considered during the discovery and development of new anticancer agents. To assess the toxicity and selectivity of the new compounds (**15a–e**, **16a–e**, **20**, and **21**), their growth inhibitory activities against normal human foetal lung fibroblast MRC-5 cells were evaluated using the MTT assay. The results expressed as IC_50_ values are presented in Table 3. The results revealed variable cytotoxic activity of the new compounds against the normal MRC-5 cells (IC_50_=0.155–17.080 μM), compared to 25.810 and 9.00 μM for compounds **9a**,**b**, respectively^13^. However, compounds **15c,e**, and **16a**,**b**,**d** were more potent and/or more selective than compound **9a**. The selectivity of the new compounds towards each of the three cancer cell lines was calculated by dividing their IC_50_ values against normal MRC-5 cells by their IC_50_ values against the corresponding cancer cell line (Table 3).

**Table 3.** Cytotoxicity of compounds **9a**,**b**, **15a–e**, **16a–e**, **20**, **21**, and lapatinib against normal MRC-5 cells.

Comp. no.	IC_50_ (µM ± SD)^a^*^^*MRC-5	Selectivity index^b^		
		MCF-7	HCT116	A2780
**15a**	1.735 ± 0.149	0.05	4.92	0.07
**15b**	3.325 ± 1.001	0.09	0.17	0.10
**15c**	1.979 ± 0.561	3.21	20.47	48.27
**15d**	2.073 ± 0.054	0.10	3.88	29.61
**15e**	17.080 ± 0.552	91.24	118.61	54.92
**20**	1.517 ± 0.103	0.27	6.14	1.62
**16a**	0.155 ± 0.062	1.89	1.15	3.52
**16b**	5.851 ± 1.187	86.04	63.60	32.51
**16c**	0.665 ± 0.191	0.45	20.78	4.62
**16d**	8.100 ± 0.897	114.08	83.51	270.00
**16e**	5.259 ± 1.398	14.57	30.40	5.75
**21**	0.584 ± 0.094	0.97	3.32	0.98
**9a**	25.810 ± 0.730^c^	258.10	–	49.63
**9b**	9.00 ± 0.13^c^	18.00	–	2.16
**Lapatinib**	13.660 ± 2.900	2.04	1.36	1.40

^a^ IC_50_ against MRC-5 cells after 72 h treatment with the test compound, results represent mean IC_50_±SD (*n* = 3).

^b^ Selectively index (SI)=IC_50_ value against normal MRC-5 cells/IC_50_ value against cancer cell line.

^c^ IC_50_ value quoted from our previous publication^13^.

The five Schiff bases **15a–e** exhibited lower selectivity (SI = 0.05–118.45) towards the three cancer cell lines than their benzamide analogues **16a–e** (SI = 0.45–270). Among the new compounds, compound **16d** was the most selective towards MCF-7 and A2780 cells, while compound **15e** showed the highest selectivity to the HCT116 cancer cell line (Table 3).

Evaluation of cytotoxicity of the indolizine derivatives **20** and **21** against normal MRC-5 cells revealed IC_50_ values of 1.517 and 0.584 μM, respectively. The two compounds exhibited selectivity indices in the range of 0.27–6.14 towards the three cancer cell lines. Compound **20** was more cytotoxic towards the MRC-5 cells than its pyrrolizine analogue **15a**. On the other hand, the indolizine **21** was less toxic to the normal cells compared to the pyrrolizine analogue **16a** (Table 3).

##### 3.2.1.4. Structure–activity relationship

**Figure 4.**
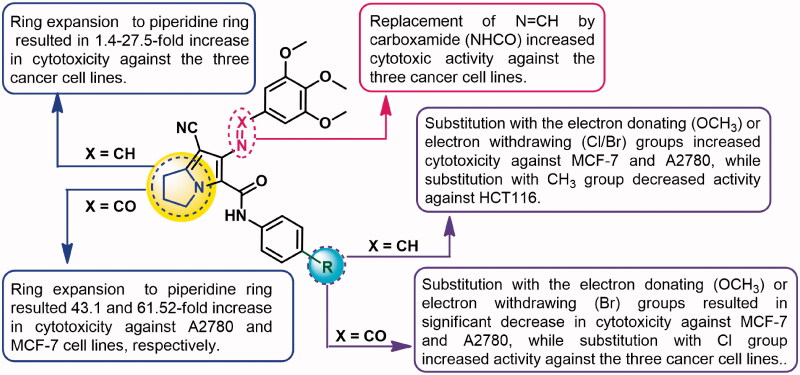
SAR of cytotoxicity of the new compounds (**15a–e**, **16a–e**, **20**, and **21**).

The study of SAR of the new compounds is represented in Figure 4. The Schiff base **15a** exhibited potent cytotoxic activity against HCT116 (IC_50_=0.353 μM) and very weak cytotoxicity against A2780 and MCF-7 cancer cell lines with IC_50_ values of 25.76 and 36.88 μM, respectively. On the other hand, replacement of the 3,4,5-trimethoxybenzylidene)amino in compound **15a** with 3,4,5-trimethoxybenzamide moiety resulted in a significant increase in the cytotoxic activities against the three cancer cell lines.

The substituents on the phenyl ring showed variable effects on cytotoxicity of the new compounds. In Schiff base derivatives **15a–e**, substitution on *para*-position of the phenyl ring of compound **15a** with electron-donating (OCH_3_) or electron-withdrawing (Cl/Br) atoms resulted in a significant increase (1.7–628.3-fold) in cytotoxic activity against MCF-7 and A2780 cells, while substitution with CH_3_ group decreased cytotoxicity against both HCT116 and A2780 cancer cell lines (Figure 4).

On the other hand, the study of SAR of the benzamide derivatives **16a–e** showed that substitution on the phenyl ring of compound **16a** with the electron-donating (OCH_3_) or electron-withdrawing (Br) groups resulted in a significant decrease (3.3–20.8-fold) in the cytotoxicity against MCF-7 and A2780, while substitution with CH_3_/Cl groups resulted in a very slight increase in cytotoxic activity against both MCF-7 and HCT116 cell lines (Figure 4).

Moreover, expansion of the pyrrolidine ring in compound **15a** to the piperidine ring (compound **20**) resulted in 1.4–27.5-fold increase in cytotoxicity against the three cancer cell lines (Figure 4).

Replacement of the 3,4,5-trimethoxybenzylidene-amino moiety in compound **15a** with 3,4,5-trimethoxybenzamido moiety resulted in a remarkable increase (2.6–585.4-fold) in cytotoxic activities against the three cell lines. Expansion of the pyrrolidine ring in compound **16a** to piperidine ring resulted in an increase in cytotoxic activity against the three cancer cell lines.

In conclusion, the 3,4,5-trimethoxybenzamido moiety and the electron-withdrawing atoms on the phenyl ring at C-5 on the pyrrolizine scaffold are favoured for high cytotoxic outcomes.

#### 3.2.2. Annexin V-FITC/PI apoptosis assay

**Figure 5.**
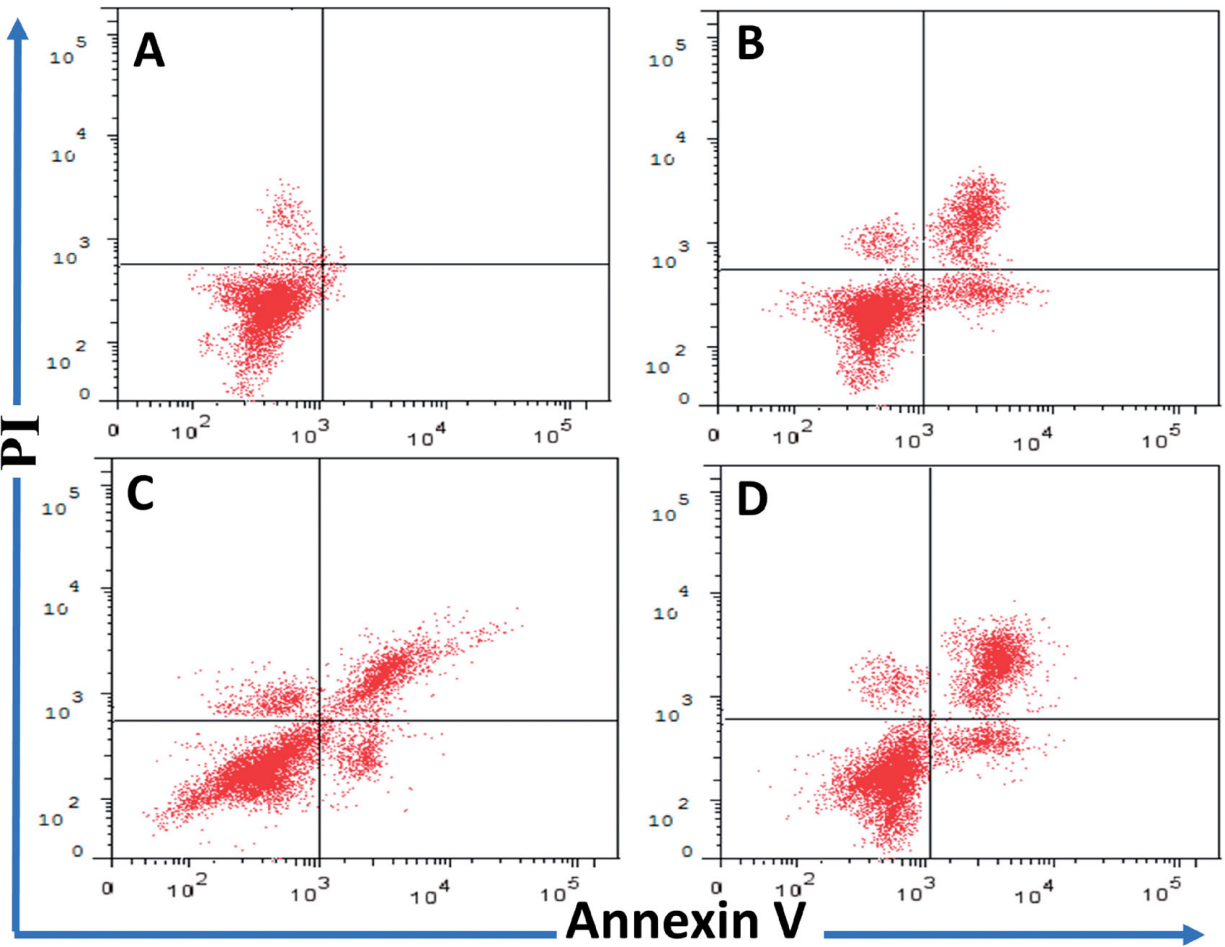
Evaluation of apoptosis-inducing activity of compounds **16a**,**b**,**d** in MCF-7 cells using annexin V FITC/PI staining assay for 24 h (*n*= 3). *x*-axis: annexin V, *y*-axis: PI. (A) Vehicle control, (B) compound **16a** (0.10 µM), (C) compound **16b** (0.1 µM), (D) compound **16d** (0.10 µM). Top left quarter: necrosis (PI+/annexin V–); top right quarter: late apoptosis (PI+/annexin V+); bottom left quarter: living cells (PI–/annexin V–); bottom right: early apoptosis (PI–/annexin V+).

The cytotoxic activity of our previously reported pyrrolizine-5-carboxamide **9a** was mediated by the induction of apoptosis in MCF-7 cells^13^^,^^19^. Accordingly, compounds **16a**,**b**,**d**, the most active in the MTT assay (Table 1) were selected to investigate their apoptosis-inducing activities. The cancer cells were treated with the test compounds (0.1 μM) for 24 h. The induction of apoptosis in MCF-7 cells by the selected compounds was investigated using annexin V fluorescein isothiocyanate (FITC)/propidium iodide (PI) staining assay. The assay procedures were the same as those applied in the previous report^13^. The results are presented in Table 4 and Figure 5. Compound **16a** increased apoptosis (early and late events) in MCF-7 cells to 19% compared to 1% in the control. Moreover, compound **16b** caused a significant increase of MCF-7 apoptotic cells which was similar to the effect of compound **16a**. However, compound **16d** was more potent in inducing apoptosis in MCF-7 cells (26%) compared to compounds **16a** and **16b**.

**Table 4.** Induction of apoptosis in MCF-7 cells treated with compounds **16a**,**b**,**d**.

Comp. no.	% Cell number^a^			
	Live cells	Early apoptosis	Late apoptosis	Necrosis
**Control**	97.20 ± 3.13	0.48 ± 0.04	0.36 ± 0.06	1.90 ± 0.20
**16a**	77.40 ± 1.39	8.40 ± 0.87	10.70 ± 1.06	3.50 ± 0.37
**16b**	75.00 ± 2.90	6.22 ± 0.22	13.01 ± 1.02	7.11 ± 0.80
**16d**	71.99 ± 2.88	7.50 ± 0.95	19.94 ± 1.30	2.51 ± 0.45

^a^ The results were obtained on treatment of MCF-7 cells by compounds **16a**,**b**,**d** (0.1 μM) or vehicle control for 24 h (*n*= 3), values represent mean%±SD of three independent experiments.

The test compounds also caused variable degrees of necrosis in MCF-7 cells. Compounds 1**6a**,**d** caused slight increases (3.50% and 2.51%, respectively) in the necrotic events compared to by the control (1.90%). On the other hand, compound **16b** caused a 7.11% increase in necrotic events.

In conclusion, the new compounds **16a**,**b**,**d** induced higher apoptotic events in MCF-7 cells at lower concentration compared to compound **9a** (Table 4).

#### 3.2.3. Kinase inhibitory activity

##### 3.2.3.1. Kinase profiling test

Compound **9a**, the lead compound in this study, was previously evaluated in a kinase profiling test to identify its mechanism of action^13^. The results revealed the ability of compound **9a** to inhibit the activity of five oncogenic kinases including ALK1, CDK-2/cyclin A1, DYRK3, GSK3 alpha, and NEK1 (inhibition%=3–25%) (Table 5).

**Table 5.** Kinases inhibitory activity by compounds **9a**, **16a**,**b**,**d** and imatinib.

Kinase	Compounds					
	16a	16b	16d	Imatinib	**9a**^a^	
ALK1	–12%	–11%	–19%	14%	–5%	
AMPK (A1/B1/G1)	0%	–9%	2%	18%	12%	
ASK1	9%	1%	10%	5%	26%	
Aurora A	2%	1%	–14%	–38%	1%	
BLK	–8%	–11%	–2%	–3%	7%	
BRAF	58%	51%	53%	13%	60%	
CDK2/cyclin A1	–28%	–21%	–19%	–2%	–4%	
CK1 alpha 1	–15%	–15%	–7%	0%	6%	
DYRK3	–4%	–12%	–17%	–10%	–25%	
EGFR	–38%	–11%	4%	13%	40%	
EPHA1	–22%	–13%	5%	22%	25%	
FLT1	21%	19%	17%	–10%	1%	
GRK1	–9%	–13%	–14%	2%	1%	
GSK3 alpha	–18%	–22%	–11%	–1%	–3%	
MSK1	–14%	–5%	23%	49%	29%	
NEK1	9%	4%	1%	–2%	–14%	
p38 alpha	–12%	–13%	22%	17%	33%	
PDK1	–10%	–1%	12%	34%	28%	
PRKG1	9%	–1%	1%	4%	3%	
SGK1	–19%	–13%	2%	3%	7%	

The negative values indicate inhibition, while the positive values indicate the activation in kinase activity.

^a^ Data quoted from our previous publication^13^.

**Figure 6.**
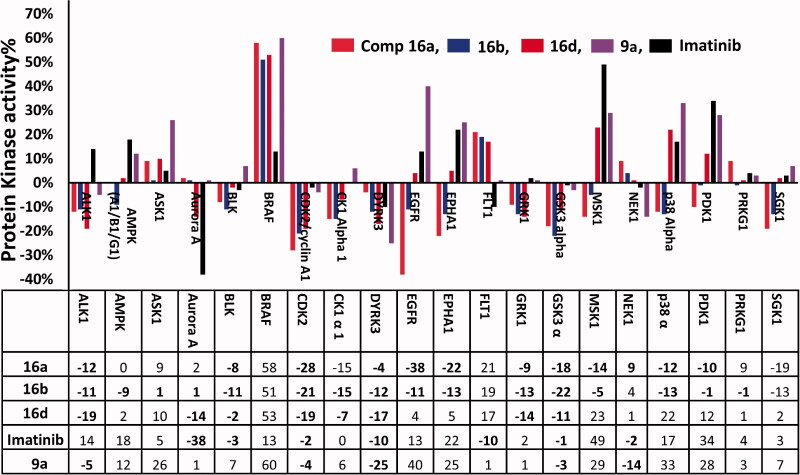
Inhibitory activity of compounds **9a**, **16a**,**b**,**d**, and imatinib against 20 protein kinases; negative values indicate inhibition in kinase activity, positive values indicate the activation of the enzyme.

In the current work, compounds **16a**,**b**,**d**, the most active in MTT assay were also selected for the kinase profiling test to investigate their potential mechanism of action. The three compounds were tested at the same concentration (10 μM) against the same 20 kinases used in the previous report^13^. The test was performed by KINEXUS Bioinformatics Corporation (Vancouver, Canada), following previous reports^13^^,^^20^. The results are presented in Table 5 and Figure 6.

The results revealed inhibition in the activities of 16 kinases by compounds **16a**,**b**,**d** with inhibition% in the range of 1–38%. The activities of seven of these kinases were inhibited by the three compounds (2–28%) (Table 5 and Figure 6).

Treatment of CDK-2/cyclin A1 with compounds **16a**,**b**,**d** resulted in 19–28% inhibition in the activity of the kinase compared to only 2% and 4% inhibition by imatinib and compound **9a**, respectively. Compounds **16a**,**b** also showed inhibitory activity against EGFR with inhibition% of 30 and 11%, respectively. Moreover, compound **16d** exhibited 14% inhibition in the activity of Aurora A kinase compared to 38% inhibition for imatinib.

In addition, weak inhibition in the activities of ALK1 (11–19%), BLK (2–11%), CK1 Alpha 1 (7–15%), and DYRK3 (4–17%) was also observed following the treatment with compounds **16a**,**b**,**d**. In addition, compounds **16a,b** displayed inhibitory activity against EPHA1, MSK1, p38 Alpha, and SGK1 with inhibition% in the range of 5–22% (Table 5). Although these inhibitions in the activities of these kinases are weak, but they may also contribute to the overall cytotoxic activities of the three compounds.

The results of the kinase profiling test also showed significant improvement in the inhibitory activities of compounds **16a**,**b**,**d** compared to compound **9a**. This improvement was observed in the number of oncogenic kinases (8–13 kinases) inhibited by the new compared to only five kinases inhibited by compound **9a**. In addition, compounds **16a**,**b**,**d** exhibited higher inhibitory activities (1–38%) than those of compound **9a** (3–25%) (Table 5 and Figure 6). These results suggested that the aromatic fragment in the side chain at C-6 of the pyrrolizine nucleus could have a better impact on the inhibitory activities of the new pyrrolizines compared to the aliphatic side chain in compound **9a**.

##### 3.2.3.2. CDK-2 inhibitory activity

The results of the kinase profiling test (Table 5 and Figure 6) revealed weak to moderate inhibitory activity against 7 kinases by compounds **16a**,**b**,**d**. Among these kinases, CDK-2 was inhibited by the three compounds with inhibition% in the range 19–28%. To confirm the activity against CDK-2, the three compounds were also evaluated for their CDK-2 inhibitory activity using the ADP-Glo kinase assay kit (Promega, Madison, WI) according to the previous reports^21^. The results expressed in the IC_50_ values of the three compounds are presented in Table 6.

**Table 6.** IC_50_ values of compounds **9a**, **16a**,**b**,**d** and staurosporine against CDK-2.

Comp. no.	IC_50_ (μM)^a^±SEM
**16a**	0.086 ± 0.016
**16b**	0.265 ± 0.021
**16d**	0.113 ± 0.017
**9a**	1.270 ± 0.066^b^
**Staurosporine**	0.021 ± 0.013

^a^ Average of three determinations; IC_50_, concentration which decrease CDK-2 activity to 50%.

^b^ Value quoted from previous publication^13^.

The results revealed higher inhibition in activity CDK-2 by compounds **16a**,**b**,**d** compared to compound **9a**, which was matched with the results of the kinase profiling test (Table 5). Among the three derivatives, compound **16a** with the unsubstituted phenyl ring was the most potent as a CDK-2 inhibitor (IC_50_=0.086 μM). Moreover, compound **16d** also exhibited potent inhibitory activity against CDK-2 (IC_50_=0.113 μM), while **16b** with the electron-donating methyl group was the least active among the three benzamide derivatives (Table 6). These results suggested that for better inhibitory activity of CDK-2, the phenyl ring in scaffold A should be unsubstituted or substituted with electron-withdrawing group(s) rather than the electron-donating (4-CH_3_) group.

#### 3.2.4. Cell cycle analysis

**Figure 7.**
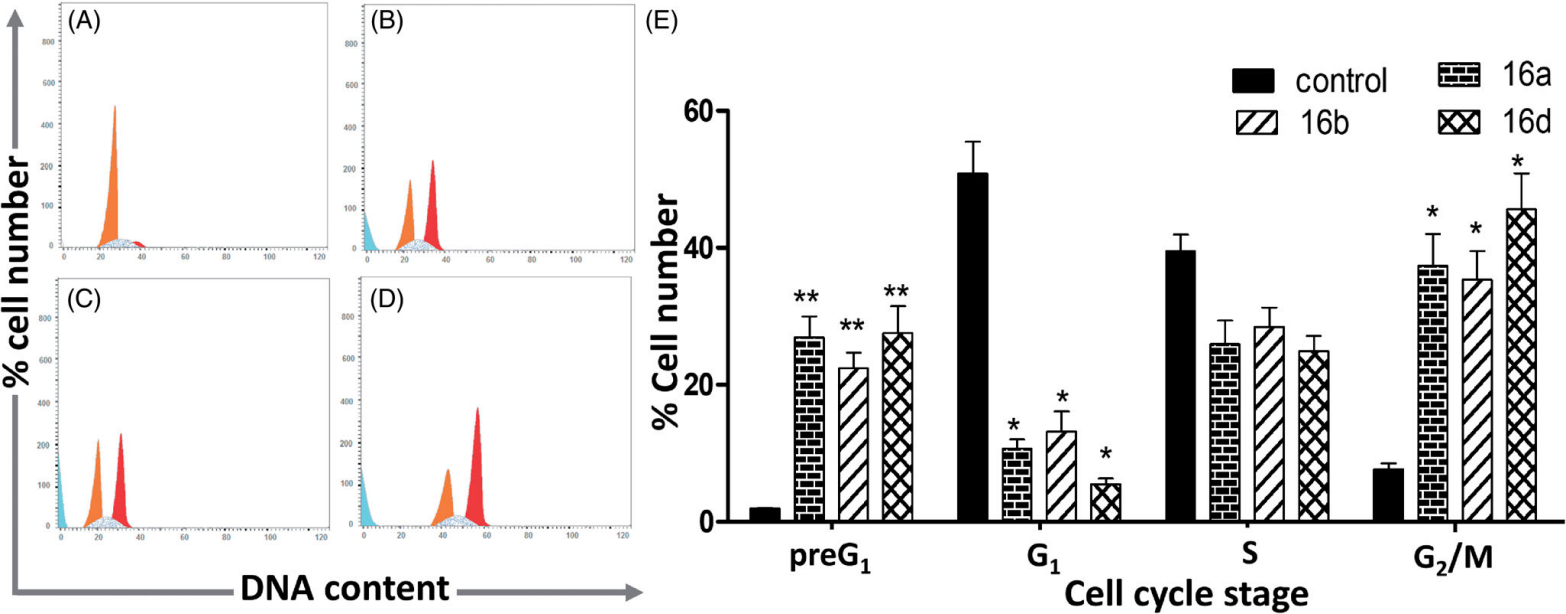
Cell cycle analysis of MCF-7 cells treated with (A) vehicle (control), (B) compound **16a** (0.1 µM), (C) compound **16b** (0.1 µM), and (D) compound **16d** (0.1 µM) for 24 h. *x*-axis: DNA content, *y*-axis: % cell number; (E) bar chart showing the effect of vehicle (control) and compounds **16a**,**b**,**d** (0.1 µM) on cell cycle stages of MCF-7 cells after 24 h treatment. Data shown in mean %±SD (*n* = 2) of three independent experiments, statistical differences, compared with control cells, were assessed by one-way ANOVA with the Tukey’s post hoc multiple comparison test (GraphPad Prism, La Jolla, CA). *p< 0.05 and **p< 0.01 were taken as significant.

Based on the results of the MTT assay (Table 1), compounds **16a**,**b**,**d** were selected to investigate their effect on cell cycle phases of MCF-7 cells. The cancer cells were treated with the test compounds at 0.1 μM and vehicle (control) for 24 h according to the previous report^13^. Cell cycle analysis was performed using FC500 flow cytometer (FC500, Beckman Coulter, Miami, FL). The revealed dramatic increases in the preG_1_ (11–14-fold) and G_2_/M (5–7-fold) cell cycle phases at the expense of 5–10-fold decrease of G_1_ events, all compared to control. On the other hand, the S phase was the least affected with less than a twofold decrease. Compound **16d** showed the strongest preG_1_ (27.56%) and G_2_/M (45.62%) cell cycle effects in MCF-7 cells (Table 7, Figure 7).

**Table 7.** Cell cycle effect of **16a**,**b**,**d** on MCF-7 cells (24 h).

Comp. no.	Cell cycle stage %			
	preG_1_	G_1_	S	G_2_/M
**Control**	1.95 ± 0.10	50.83 ± 4.62	39.51 ± 2.44	7.66 ± 0.84
**16a**	26.92 ± 3.07	10.69 ± 1.32	25.93 ± 3.47	37.38 ± 4.66
**16b**	22.43 ± 2.24	13.19 ± 2.95	28.46 ± 2.79	35.35 ± 4.19
**16d**	27.56 ± 3.93	5.46 ± 0.87	24.92 ± 2.22	45.62 ± 5.25

Data shown in mean %±SD (*n* = 2), treatment for 24 h at 0.1 μM, experiment was repeated 3×.

Cell cycle analysis of compound **9a** (1 µM, 24 h) revealed blockade of MCF-7 cells at the S phase with more than a threefold increase compared to the control (17.3–55.6%), at the expense of decreases in other cell cycle phases^13^. However, in the current study, compounds **16a**,**b**,**d** (0.1 µM) showed completely different effects on cell cycle phases of MCF-7 cells at a much lower concentration compared to compound **9a**. The three compounds **16a**,**b**,**d** increased the preG_1_ and G_2_/M cell cycle phases in MCF-7 cells at the same time point (24 h) (Table 7).

#### 3.2.5. Tubulin polymerisation

##### 3.2.5.1. Tubulin polymerisation assay (kinetic study)

In the current study, the new compounds were designed bearing the trimethoxy phenyl moiety aiming to obtain new cytotoxic agents that can interfere with tubulin polymerisation in cancer cells. Herein, compounds **16a**,**b**,**d**, the most active in MTT assay were selected to investigate their effects on the kinetics of the three phases of tubulin polymerisation (I: nucleation, II: growth, and III: steady-state equilibrium). In this assay, light scattering by microtubules which is directly proportional to the concentration of the microtubule polymer was used as a measure of tubulin polymerisation. The study was performed using a fluorescence-based tubulin polymerisation assay kit (Cytoskeleton Inc., Denver, CO; Cat # BK011P) following the manufacturer’s instructions (https://www.cytoskeleton.com/bk011p) and according to the previous report^22^.

**Figure 8.**
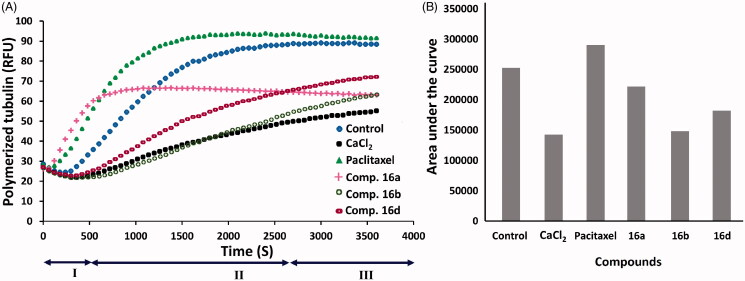
Effect of compounds **16a**,**b**,**d** on tubulin polymerisation: (A) tubulin polymerisation reactions of control, paclitaxel, CaCl_2_, and compounds **16a**,**b**,**d**, I: nucleation, II: growth, and III: steady-state equilibrium phases; (B) semi-quantitative analysis of the inhibition in tubulin polymerisation showing the AUC on treatment with vehicle (control), paclitaxel, CaCl_2_, and compounds **16a**,**b**,**d**.

Paclitaxel (3 μM) was used as a tubulin polymerisation enhancer (positive control, enhancer), while calcium chloride (CaCl_2_, 0.5 μM) was used as a positive control (inhibitor) as per the manufacturer’s instructions (https://www.cytoskeleton.com/pdf-storage/datasheets/bk011p.pdf). The vehicle was also used to evaluate the polymerisation when tubulin is incubated in the absence of the test compound. The effect of the selected compounds (5 μM) on tubulin polymerisation was evaluated and compared with those of the positive control. The results are represented in Figure 8.

The results revealed that the tubulin polymerisation enhancer, paclitaxel at 3 μM eliminated the nucleation phase **I** and enhanced the growth phase **II**. On the other hand, CaCl_2_ at 0.5 μM inhibited tubulin polymerisation and reduced the final polymer mass (Figure 8). The results also indicated moderate tubulin polymerisation inhibitory activity for compounds **16b**,**d** compared to CaCl_2_. The inhibitory effect of compound **16b** was higher than compound **16d**. The effect of compound **16d** on both nucleation and growth phases was similar to those of CaCl_2_, while its effect on the steady-state equilibrium phase was slightly lower than CaCl_2_ (Figure 8). On the other hand, compound **16a** showed a similar effect to that of paclitaxel on the nucleation phase. However, this effect reached a plateau then started to decrease in the growth phase. The inhibitory effect of compound **16a** on tubulin polymerisation was continued in the steady-state phase.

In the current work, the tubulin polymerisation assay was used as a qualitative assay to identify the potential mechanism of action of the new compounds **16a**,**b**,**d**. However, area under the curve (AUC) indicates the total light scattered and corresponds to the total mass of the polymerised tubulin (Figure 8(A)). Accordingly, AUC was calculated for the tested compounds **16a**,**b**,**d** and compared with those of the control, CaCl_2_, and paclitaxel (Figure 8(B)). The results showed a decrease in AUC of CaCl_2_ compared to the control indicating an inhibition in tubulin polymerisation and a decrease in the final polymer mass. On the other hand, an increase in AUC was observed by paclitaxel relative to control indicating a stabilisation in tubulin polymerisation. Moreover, compounds **16a**,**b**,**d** exhibited similar effects on tubulin polymerisation like CaCl_2_. Among the three compounds, compound **16b** exhibited the highest reduction in the final polymer mass. In addition, compound **16d** showed a higher reduction in the final polymer mass than compound **16a**. Considering the results of the three compounds, we can observe that compounds **16b**,**d** have moderate inhibitory activity against tubulin polymerisation compared to the control and CaCl_2_, while the effect of compound **16a** was weak.

##### 3.2.5.2. Immunofluorescence staining

To assess the effect of the new compounds on microtubule cytoskeleton in living cells, compound **16b**, the most active in tubulin polymerisation assay (Figure 8) was selected. The effect of compound **16b** was evaluated in an immune staining study using confocal immunofluorescent microscopy. The test was performed according to the previous report^23^. The cancer cells were incubated with the vehicle (control) and compound **16b** (5 μM) for 24 h. The nuclei of the cancer cell stained blue with 4′,6-diamidino-2-phenylindole (DAPI), while microtubules were stained (green) with anti-β-tubulin mouse monoclonal antibody and Alexa Fluor^®^ 488 secondary antibodies (Abcam, Cambridge, UK).

**Figure 9.**
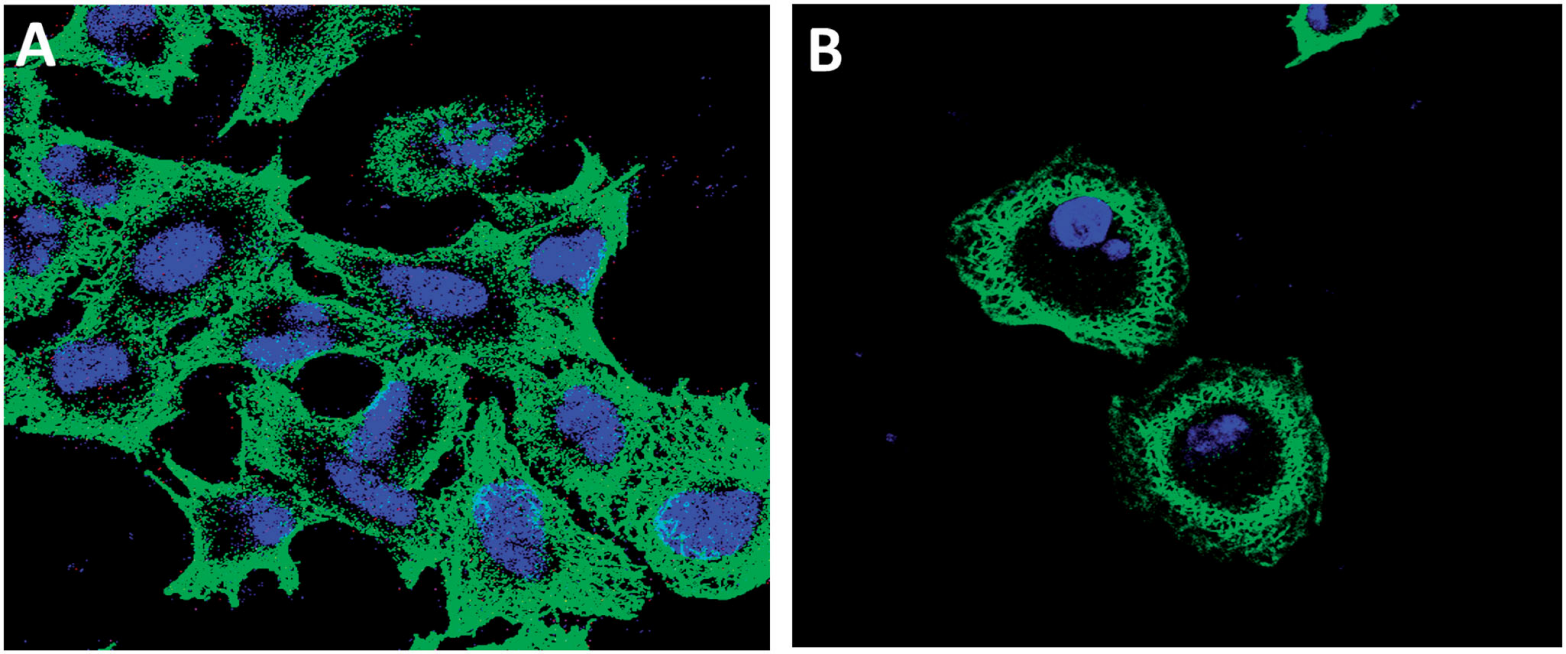
Immunofluorescence confocal microscopy images assessing cellular microtubule networks of MCF-7 cells after treatment with vehicle (A), compound **16b** (B) for 24 h, nuclei were stained blue with 4′,6-diamidino-2-phenylindole (DAPI), microtubules stained (green) with anti-β-tubulin mouse monoclonal antibody and Alexa Fluor^®^ 488 secondary antibodies (Abcam, Cambridge, UK).

The immunofluorescence images of the untreated/treated cancer cells were taken to visualise the effect of the test compounds **16b** on the microtubule cytoskeleton. The results are represented in Figure 9. The images of the control showed normal, clear slim, and fibrous microtubules which wrap around the nuclei and stretch through each cell. On the other hand, the cells treated with compound **16b** showed shrank and partially disorganised microtubules which split partially into fragments. These results indicated the ability of compound **16b** to induce a disruption in the formation of microtubules.

### 3.3. Molecular docking

In the current work, the molecular docking studies were performed into protein kinases (PKs) and tubulin to understand the mechanism of action of compounds **16a**,**b**,**d**. The binding affinities, modes, orientations, and interactions of the new compounds into the relevant protein were investigated using AutoDock 4.2^24^ and compared with those of the co-crystallised ligands. The results of the docking studies were visualised by the Discovery studio visualizer (Dassault Systems, San Diego, CA)^32^.

#### 3.3.1. Docking study into protein kinases

To rationalise the improvement of kinase inhibitory activity of the new compounds **16a**,**b**,**d** compared to the lead compound **9a**, a molecular docking study of the new compounds into the active sites of CDK-2, EGFR, and Aurora A were performed. The crystal structure of CDK-2 (pdb code: 3TNW)^25^, EGFR (pdb code: 1M17)^26^, and Aurora A (pdb code: 3E5A)^27^ was obtained from the protein data bank (https://www.rcsb.org/). AutoDock (v.4.2) was used to perform the docking study^24^. The study was performed following the previous reports^29–31^. Validation of the docking procedures into the selected kinases was also performed according to our previous report^13^. The results of the docking study of the new compounds and the co-crystallised ligands are presented in Table 8.

**Table 8.** Docking results of compounds **16a**,**b**,**d** into CDK-2 and EGFR in comparison to compounds **9a** and the co-crystallised ligands.

PK (pdb)	Ligand	Δ*G*_b_^a^	*K*_i_^b^	HBs^c^	Atoms in H-bonding		Length^d^ (Å)
					In ligands	In tubulin	
CDK-2 (3TNW)	**16a**	–9.21	0.178 μM	5	CN	NH of LEU83	2.08
					CONH	NH of LEU83	2.61
					3″-OCH_3_	NH_3_ of LYS89	2.10
					4″-OCH_3_	NH_3_ of LYS89	1.95, 2.50
	**16b**	–7.91	1.60 μM	3	CONH	C═O of ILE10	2.75
					CN	NH of LEU83	2.05
					CONH	NH_3_ of LYS89	2.32
	**16d**	–8.37	0.732 μM	3	CN	NH of LEU83	1.95
					3″-OCH_3_	NH_3_ of LYS33	2.47
					4″-OCH_3_	NH_3_ of LYS33	2.86
	**9a**	–8.04	1.290 µM	1	CN	NH of ASP145	2.07
	**CAN508**^e^	–6.47	18.20 µM	5	OH	NH of LYS33	1.74
					OH	C═O of GLU51	2.114
					NH_2_	C═O of GLU81	1.85
					Pyrazole N_1_	NH of LEU83	1.69
					OH	C═O of ASP145	2.69
EGFR (1M17)	**16a**	–9.07	223.89 nM	3	CONH	OH of THR830	2.36
					CN	NH of ASP831	2.25
					CONH	C═O of ASP831	3.05
	**16b**	–8.60	498.09 nM	3	3″-OCH_3_	NH_3_ of LYS721	2.68
					4″-OCH_3_	NH_3_ of LYS721	1.73
					CN	NH of MET769	1.89
	**16d**	–8.53	555.92 nM	1	CO	CYC773	2.42
	**9a**	–8.51	577.35 nM	2	CONH	C═O of GLN767	3.03
					CONH	NH of MET869	2.24
	**Erlotinib**	–7.39	3.84 µM	1	Pyrimidine N_1_	NH of MET769	1.64

^a^ Binding free energy (kcal/mol).

^b^ Inhibition constant.

^c^ HBs, number of hydrogen bonds.

^d^ Length in angstrom (Å).

^e^ CAN508, -4-[(*E*)-(3,5-diamino-1*H*-pyrazol-4-yl)diazenyl)phenol.

The results of the docking study of compounds **16a**,**b**,**d** into the selected kinases revealed nice fitting into the active site of CDK-2 and EGFR. These results were confirmed by the absence of any steric clashes or unfavourable interactions with the amino acids in the active sites of the two kinases. Moreover, compounds **16a**,**b**,**d** exhibited higher binding affinities towards CDK-2 and EGFR compared to CAN508, and erlotinib, respectively (Table 8). The three compounds **16a**,**b**,**d** exhibited higher affinities (–7.91 to −9.21 kcal/mol) towards CDK-2 compared to −6.47 kcal/mol for CAN508 (IC_50_=3.5 μM against CDK-2)^39^. Compound **16a** showed the highest affinity towards CDK-2 followed by the chloro analogue **16d**. This order of binding affinities was in concordance with the inhibitory activities of the three compounds against CDK-2 (Table 6).

To understand the differences in the inhibitory activity of the three compounds against CDK-2 (Table 6), the binding modes and interactions of compounds **16a**,**b**,**d** were also investigated. The results revealed that each of the two side chains in the three compounds can adopt an orientation that occupies the binding site of CAN508 in CDK-2.

**Figure 10.**
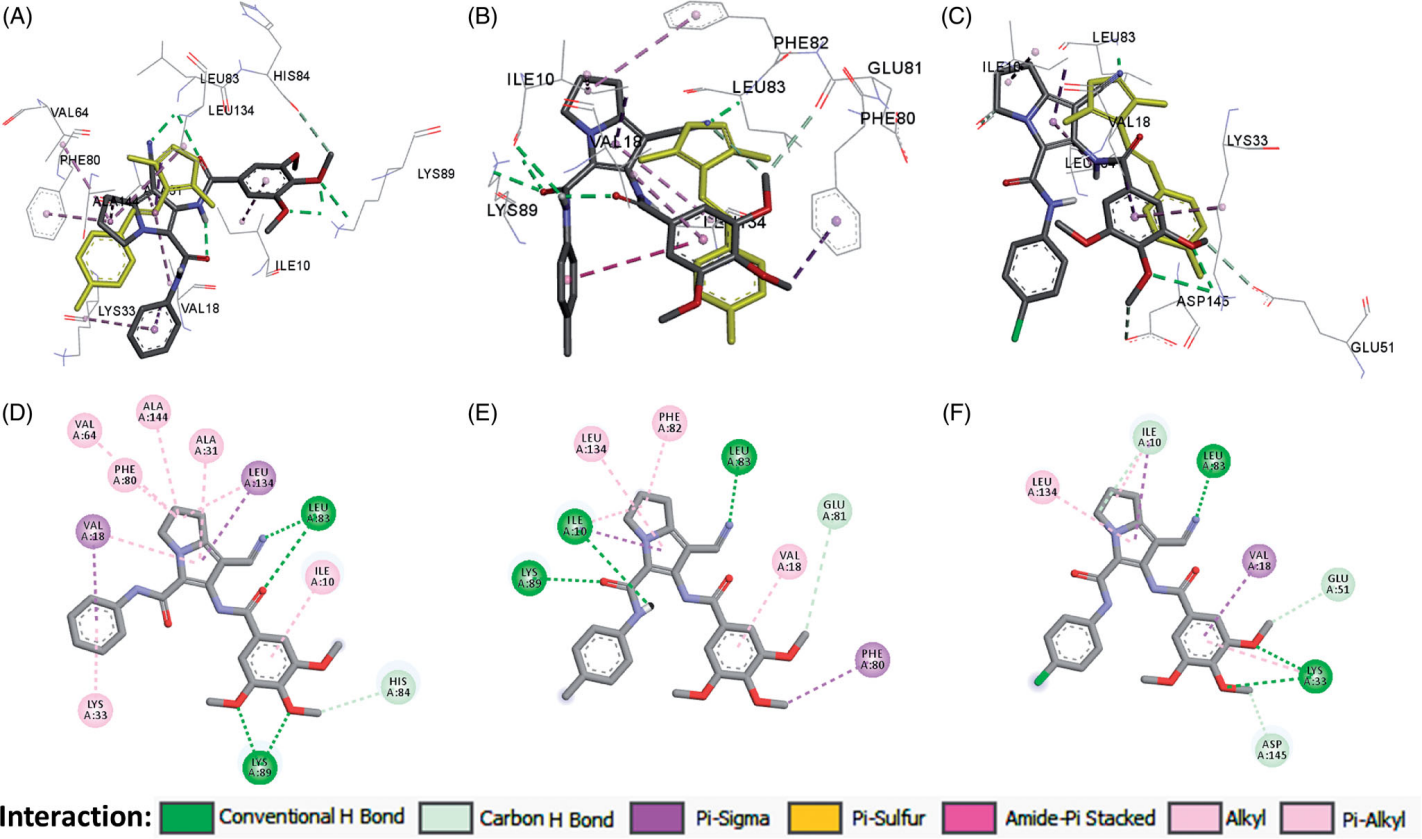
Binding modes/interactions of compounds **16a**,**b**,**d** (shown as sticks, coloured by element) and CAN508 (coloured in yellow) into CDK-2 (pdb code: 3TNW): (A) 3D binding mode of compound **16a** overlaid with CAN508; (B) 3D binding mode of compound **16b** overlaid with CAN508; (C) 3D binding mode of compound **16d** overlaid with CAN508; (D) 2D binding mode of compound **16a**; (E) 2D binding mode of compound **16b**; (F) 2D binding mode of compound **16d**, all showing H-bonding and hydrophobic interactions, hydrogen atoms were omitted for clarity.

Investigation of the binding mode of compound **16a** revealed that the pyrrole ring together with the attached phenyl carboxamide side-chain adopted an orientation nearly superposing the position of CAN508 in CDK-2 (Figure 10). On the other hand, the pyrrole ring and the trimethoxyphenyl moiety in compounds **16b**,**d** nearly superpose the positions of the imidazole and phenyl rings in CAN508, respectively. These differences in binding orientations of the three compounds can account even partially for the difference in their inhibitory activity against CDK-2.

Moreover, investigation of the binding interaction of compound **16a** into the active site of CDK-2 revealed the formation of five conventional hydrogen bonds with LEU83 and LYS89. One carbon hydrogen bond was also observed between compound **16a** and HIS84. In addition, compound **16a** displayed several hydrophobic interactions with the key amino acids (ILE10, VAL18, ALA31, LYS33, VAL64, PHE80, LEU134, and ALA144) in the ATP binding site of CDK-2. These binding interactions were similar to those reported for several CDK-2 inhibitors^40^. On the other hand, each of compounds **16b**,**d** displayed only three conventional hydrogen bonds with amino acids in CDK-2. The fewer number of conventional hydrogen bonds could also account for the lower inhibitory activities of compounds **16b**,**d** compared to compound **16a** (Figure 10).

Compounds **16a**,**b**,**d** also displayed higher binding affinities towards EGFR compared to erlotinib (Table 8). Among the three derivatives, compound **16a** showed the highest affinity for EGFR protein (Δ*G*_b_= −9.07 kcal/mol, Table 8). In addition, compound **16b** displayed a binding affinity of −8.60 kcal/mol which was lower than the binding affinity of compound **16a**. These results were matched with the inhibitory activities of the two compounds against EGFR (Table 5).

**Figure 11.**
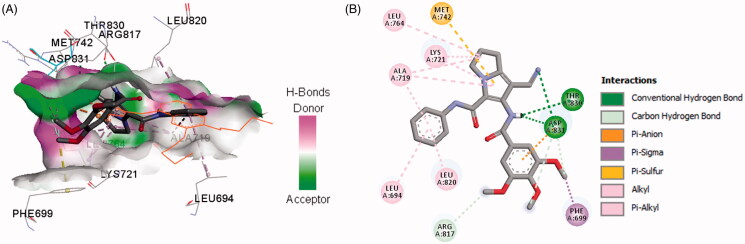
Binding modes/interactions of compound **16a** into EGFR (PDB code: 1M17): (A) 3D binding mode of compound **16a** into the active site of EGFR, the co-crystallised erlotinib shown as orange line, receptor shown as hydrogen bond surface, hydrogen atoms were omitted for clarity; (B) 2D binding mode of compound **16a** into EGFR showing different types of interactions with amino acids in the active site of the protein, hydrogen atoms were omitted for clarity.

However, compound **16d** displayed a good binding affinity (Δ*G*_b_= −8.53 kcal/mol) although it did not show *in vitro* inhibitory activity against EGFR (Table 5). To explain this difference, the binding interactions between the tested compounds (**16a**,**b**,**d**, and erlotinib) and the amino acids in EGFR were investigated. The results revealed that compound **16a** exhibited three conventional hydrogen bonds with THR830 and ASP831 (Figure 11). Compound **16b** also showed three conventional hydrogen bonds with LYS721 and MET769. However, investigation of the binding interactions of the best fit conformation of compound **16d** into EGFR revealed only one conventional hydrogen bond formed with CYC773 (Table 8). These results indicated that the binding affinity of compound **16d** towards EGFR is due mainly to the hydrophobic interactions with EGFR. The 2/3D binding modes of compounds **16b**,**d**, and erlotinib are provided in the Supplementary data (Figs. S142–144).

In addition, among the three derivatives **16a**,**b**,**d**, only compound **16d** showed weak inhibitory activity against aurora A (14% inhibition) in the kinase profiling test. The docking study of compounds into the active site of aurora A (pdb code: 3E5A) revealed the highest affinity for compound **16d**, Supplementary data (Table S3).

#### 3.3.2. Docking study into tubulin

In this part, another molecular docking study was performed into the binding site of CA-4/colchicine in tubulin protein to investigate the binding mode/interactions of the new compounds correlate the obtained results with the inhibitory effects of these compounds on tubulin polymerisation (Figures 8 and 9). The crystal structure of tubulin protein (pdb code: 5LYJ) co-crystallised with CA-4^28^ was obtained from the Protein Data Bank (https://www.rcsb.org/structure/5lyj). The protein and ligand files were prepared following the previous report^29^^,^^41^.

**Figure 12.**
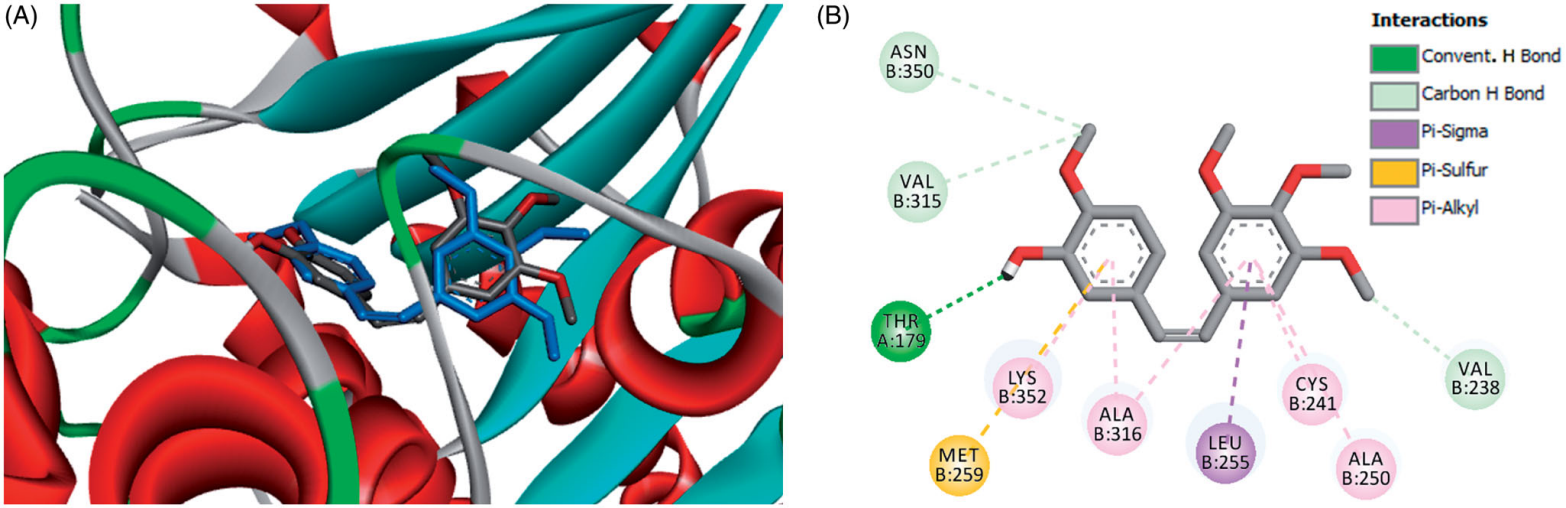
Superposition of the re-docked/co-crystallised CA-4 **1** into the binding site in tubulin (pdb code: 5LYJ): (A) 3D binding modes showing superposition of the re-docked CA-4 (shown as sticks, coloured by element) over the co-crystallised ligand (CA-4, shown as sticks, coloured in blue) into CA-4 **1** binding site in tubulin, RMSD = 0.9 Å; (B) 2D binding mode of CA-4 showing H-bonding and hydrophobic interactions with amino acids in tubulin, hydrogen atoms were omitted for clarity.

To validate the docking procedures, the co-crystallised ligand (CA-4) **1** was re-docked into the binding site of the co-crystallised ligand (7BA) in tubulin protein. The results revealed the superposition of the re-docked CA-4 with the co-crystallised ligand with RMSD of 0.9 Å. Moreover, the re-docked CA-4 showed similar binding mode, orientation, and exhibited the same binding interactions as those of the co-crystallised ligand (Figure 12).

Investigation of the binding interactions of CA-4 with the amino acids in tubulin protein revealed one conventional hydrogen bond with THR179, one pi-sulphur interaction with MET259, and three carbon hydrogen bonds with VAL238, VAL315, and ASN350. In addition, several hydrophobic interactions with CYS241, ALA250, LEU255, ALA316, and LYS352 were observed (Figure 12).

The results of the molecular docking study (Table 9) revealed a nice fitting of the new compounds into the binding site of CA-4 in tubulin, where no steric clashes or unfavourable interactions were observed. Moreover, the benzamide derivatives (**16a–e** and **21**) displayed higher binding affinity (Δ*G*_b_= −8.80 to −12.16 kcal/mol) compared to −8.72 kcal/mol for CA-4, Supplementary data (Table S4). Moreover, the benzamide derivatives (**16a–e** and **21**) also displayed higher binding free energies than their corresponding Schiff bases (**15a–e** and **20**). These results were also matched with the results of the cytotoxic activities of the two series (Table 1).

**Table 9.** Docking results of compounds **15a**,**b**,**d**, **16a**,**b**,**d** into tubulin protein (pdb code: 5LYJ) in comparison with the co-crystallised ligand 7BA (CA-4).

Comp.	Δ*G*_b_^a^	*K*_i_^b^	HBs^c^	Atoms in H-bonding		Length^d^ (Å)
				In ligands	In tubulin	
15a	–6.97	7.770 μM	2	4-CH_3_O	NH_2_ of ASN258	2.85
				C═O	NH of ALA317	2.35
			3*	MeO groups	ASN249, LEU248, ASN101, THR179	1.74–3.00
15b	–6.35	22.16 μM	2	4-CH_3_O	NH_2_ of ASN258	2.94
				C═O	NH of ALA317	2.49
			2*	MeO groups	LEU248, ASN249	1.74, 2.17
15d	–6.46	18.41 μM	–^d^	–	–	–
			3*	3/4-CH_3_O	CYS241, ASN258	2.03–2.74
				C═O	NH of ALA317	2.08
			3*	3/4-CH_3_O	LEU248, ASN249, ASN101	1.83–2.26
16a	–8.80	347.84 nM	2	C═O	NH_2_ of LYS352	2.22
				7-CN	NH of ASP251	2.46
			4*	3/4-CH_3_O, CH_2_-3	THR179, CYS241, GLY237, THR376, ALA316	2.07–2.61
16b	–10.97	9.06 nM	1	C═O	NH_2_ of LYS352	1.49
			4*	3/4-CH_3_O, CH_2_-3	CYS241, GLY237, THR376, ALA316, THR179	2.56–3.04
16d	–10.63	16.28 nM	1	7-CN	NH of ASP251	2.62
			4*	3/4-CH_3_O, CH_2_-3	ASN101, GLY237, THR179	2.43–2.80
7BA	–8.72	407.88 nM	1	OH	C═O of THR179	2.18
			3*	CH_3_O groups	VAL238, VAL315, ASN350	1.93–2.75

7BA, compound **1** (CA-4); values with asterisks indicate the carbon hydrogen bonds, the underlined atoms are the atoms involved in the H-bonding interactions.

^a^ Binding free energy (kcal/mol).

^b^ Inhibition constant.

^c^ HBs, number of hydrogen bonds.

^d^ Length in angstrom (Å).

**Figure 13.**
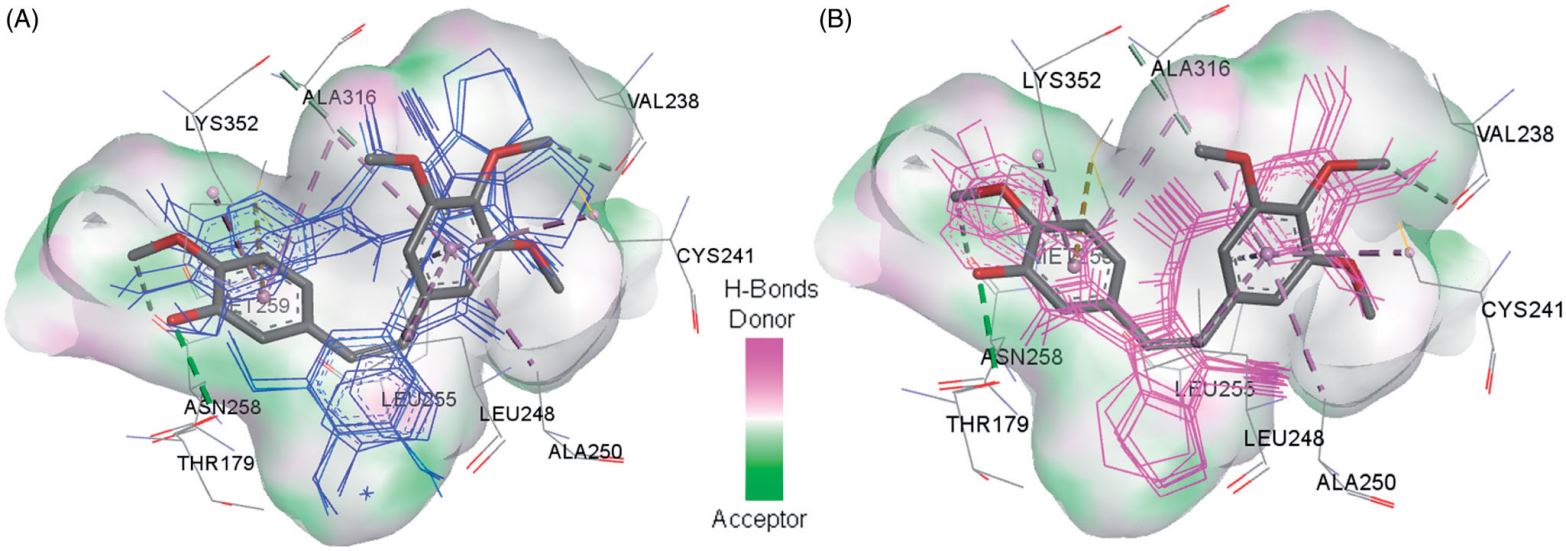
An overlay of the best fit conformation of the new compounds with the co-crystallised CA-4 shown as sticks, coloured by element (pdb code: 5LYJ): (A) compounds **15a–e** and **20** (shown as blue lines) overlaid with the co-crystallised CA-4; (B) compounds **16a–e** and **21** (shown as pink lines) overlaid with the co-crystallised CA-4, receptor in the two figs shown as hydrogen bond surface, hydrogen atoms were omitted for clarity.

To understand the reasons behind this noticeable difference in binding affinities of the two series, we have investigated their binding modes/orientations into tubulin protein. The best-fit conformations of the two series were overlaid with co-crystallised CA-4 in Figure 13. Investigation of their binding modes revealed that all the benzamide derivatives (**16a–e** and **21**) exhibited binding orientations similar to that of CA-4. The two phenyl rings in each of the six benzamide derivatives adopted orientations superposing on the two phenyl rings of CA-4 with the trimethoxyphenyl moieties extended towards the α/β interface (Figure 13).

On the other hand, the Schiff base derivatives (**15a–e** and **20**) adopted completely different orientations from CA-4. The difference in the binding orientations of the two series could account even partially for the difference in their binding affinities towards tubulin and their cytotoxic activities.

Among the new benzamide derivatives, compound **16b** displayed the highest binding affinity towards tubulin. Moreover, the chloro derivative **16d** exhibited higher binding affinity than compound **16a**. These results were quite matched with the results of tubulin polymerisation assay of the three compounds (Figure 8).

**Figure 14.**
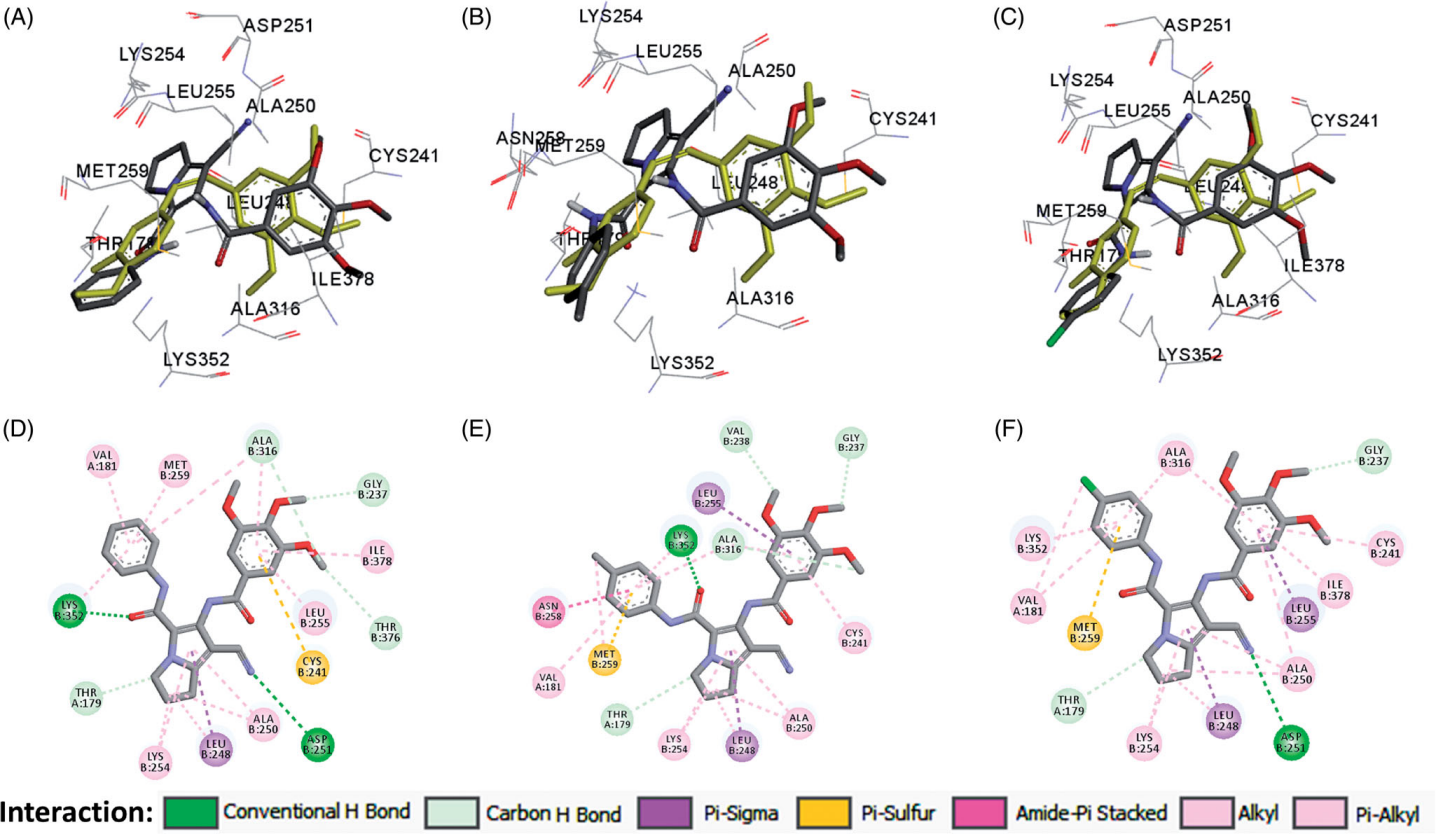
Binding modes/interactions of compounds **16a**,**b**,**d** (shown as sticks, coloured by element) into CA-4 (shown as sticks, coloured in yellow) binding site in tubulin (pdb code: 5LYJ): (A) 3D binding mode of compound **16a** overlaid with the co-crystallised CA-4; (B) 3D binding mode of compound **16b** overlaid with the co-crystallised CA-4; (C) 3D binding mode of compound **16d** overlaid with the co-crystallised CA-4; (D) 2D binding mode of compound **16a**; (E) 2D binding mode of compound **16b**; (F) 2D binding mode of compound **16d**, all showing H-bonding and hydrophobic interactions with amino acids in tubulin, hydrogen atoms were omitted for clarity.

Furthermore, the binding orientations and interactions of the benzamide derivatives **16a**,**b**,**d** into tubulin protein were deeply investigated. The results revealed that the three compounds adopted orientation like that of co-crystallised CA-4. In these orientations, the two phenyl rings of the three compounds superposed on the two phenyl rings of CA-4 (Figure 14). Like the co-crystallised CA-4, the trimethoxyphenyl moieties in the three compounds were located deeply into the β-subunit showing several interactions with important amino acids such as THR179, VAL238, and/or CYS241, through carbon–hydrogen bonding and hydrophobic interactions. Moreover, the pyrrolizine nucleus of the three derivatives was located towards the α/β interface with the double bond of the pyrrole ring (C2═C3) overlaid over the double bond in CA-4. In addition, the two phenyl rings in the three compounds were located on the same side of the pyrrole ring adopting a *cis*-like conformation similar to the *cis*-conformation of CA-4. These binding modes of compound **16a**,**b**,**d** could account for their high binding affinities towards tubulin and even partially for the high cytotoxic activities of benzamide derivative.

The docking study into the binding site of CA-4 in tubulin protein was also performed for the remaining compounds (**15c**,**e**, **16c**,**e, 20**, and **21**). The results of this study are provided in the supplementary data (Table S4).

## 4. Conclusions

In the present study, two new series of pyrrolizine derivatives (**15a–e** and **16a–e**) bearing 3,4,5-trimethoxyphenyl moiety were designed, synthesised, and evaluated for their cytotoxic activities. In addition, two new indolizines (**20** and **21**) were also synthesised and for their cytotoxic activities. Structural confirmation of these compounds was carried out by spectral (IR, ^1^H NMR, ^13^C NMR, DEPT C^135^, and mass) and quantitative elemental analyses. The results of the MTT assay revealed weak to potent cytotoxic activity for the new compounds against three (MCF-7, HCT116, and A2780) cancer cell lines with IC_50_ in the range of 0.03–36.88 μM. Among the new derivatives, compounds **16a**,**b**,**d** exhibited the highest cytotoxicity against MCF-7 cells (IC_50_=0.068–0.082 μM), while compounds **16c**,**d** were the most active against HCT116 and A2780 cells, respectively. On the other hand, the new compounds showed weak cytotoxicity against the normal MRC-5 cells (IC_50_=0.155–17.08 μM) compared to 13.66 μM for lapatinib. Compounds **16a**,**b**,**d** exhibited also cytotoxic activities against doxorubicin-resistance MCF-7/ADR cells (IC_50_=0.52–6.26 μM). The study of SAR revealed higher cytotoxic activity and selectivity for the benzamide derivatives (**16a–e** and **21)** compared to their corresponding Schiff bases (**15a–e** and **20)**. The kinase profiling test of compounds **16a**,**b**,**d** revealed improvement in their inhibitory activities against several kinases (inhibition%=1–38%) compared to compound **9a**. Moreover, the three compounds exhibited higher inhibitory activity against CDK-2 than compound **9a**. Mechanistic studies of compounds **16a**,**b**,**d** also revealed weak to moderate inhibitory activity against tubulin polymerisation. Cell cycle analyses of MCF-7 cells treated with compounds **16a**,**b**,**d** revealed dramatic increases in the preG_1_ (11–14-fold) and G_2_/M (5–7-fold) cell cycle phases at the expense of 5–10-fold decrease of G_1_ events, all compared to the control. Compound **16d** showed the strongest preG_1_ (27.56%) and G_2_/M (45.62%) cell cycle effects. The results of the docking study into tubulin/CDK-2/EGFR showed higher binding affinities for compounds **16a**,**b**,**d** compared to the co-crystallised ligands (CAN508 and erlotinib). Taken together, compounds **16a**,**b**,**d** could serve as promising lead compounds for the future development of new potent anticancer agents.

## Supplementary Material

Supplemental Material

## References

[1] Siegel RL, Miller KD, Jemal A. Cancer statistics, 2019. CA Cancer J Clin 2019;69:7–34. 30620402 10.3322/caac.21551

[2] Housman G, Byler S, Heerboth S, et al. Drug resistance in cancer: an overview. Cancers (Basel) 2014;6:1769–92. 25198391 10.3390/cancers6031769 PMC4190567

[3] Bayat Mokhtari R, Homayouni TS, Baluch N, et al. Combination therapy in combating cancer. Oncotarget 2017;8:38022–43. 28410237 10.18632/oncotarget.16723 PMC5514969

[4] Saputra EC, Huang L, Chen Y, Tucker-Kellogg L. Combination therapy and the evolution of resistance: the theoretical merits of synergism and antagonism in cancer. Cancer Res 2018;78:2419–31. 29686021 10.1158/0008-5472.CAN-17-1201

[5] Zheng W, Zhao Y, Luo Q, et al. Multi-targeted anticancer agents. Curr Top Med Chem 2017;17:3084–98. 28685693 10.2174/1568026617666170707124126

[6] Singh H, Kinarivala N, Sharma S. Multi-targeting anticancer agents: rational approaches, synthetic routes and structure activity relationship. Anticancer Agents Med Chem 2019;19:842–74. 30657048 10.2174/1871520619666190118120708

[7] Fu R-G, Sun Y, Sheng W-B, Liao D-F. Designing multi-targeted agents: an emerging anticancer drug discovery paradigm. Eur J Med Chem 2017;136:195–211. 28494256 10.1016/j.ejmech.2017.05.016

[8] Lu Y, Chen J, Xiao M, et al. An overview of tubulin inhibitors that interact with the colchicine binding site. Pharm Res 2012;29:2943–71. 22814904 10.1007/s11095-012-0828-z PMC3667160

[9] Li L, Jiang S, Li X, et al. Recent advances in trimethoxyphenyl (TMP) based tubulin inhibitors targeting the colchicine binding site. Eur J Med Chem 2018;151:482–94. 29649743 10.1016/j.ejmech.2018.04.011

[10] Ducki S, Rennison D, Woo M, et al. Combretastatin-like chalcones as inhibitors of microtubule polymerization. Part 1: synthesis and biological evaluation of antivascular activity. Bioorg Med Chem 2009;17:7698–710. 19837593 10.1016/j.bmc.2009.09.039

[11] Zhang X, Raghavan S, Ihnat M, et al. The design and discovery of water soluble 4-substituted-2,6-dimethylfuro[2,3-d]pyrimidines as multitargeted receptor tyrosine kinase inhibitors and microtubule targeting antitumor agents. Bioorg Med Chem 2014;22:3753–72. 24890652 10.1016/j.bmc.2014.04.049 PMC4089508

[12] Zhang X, Kong Y, Zhang J, et al. Design, synthesis and biological evaluation of colchicine derivatives as novel tubulin and histone deacetylase dual inhibitors. Eur J Med Chem 2015;95:127–35. 25805446 10.1016/j.ejmech.2015.03.035

[13] Shawky AM, Abourehab MAS, Abdalla AN, Gouda AM. Optimization of pyrrolizine-based Schiff bases with 4-thiazolidinone motif: design, synthesis and investigation of cytotoxicity and anti-inflammatory potency. Eur J Med Chem 2020;185:111780. 31655429 10.1016/j.ejmech.2019.111780

[14] Attallah KM, Gouda AM, Ibrahim IT, Abouzeid L. Design, synthesis, 99mTc labeling, and biological evaluation of a novel pyrrolizine derivative as potential anti-inflammatory agent. Radiochemistry 2017;59:630–8.

[15] Belal A, Gouda AM, Ahmed AS, Abdel Gawad NM. Synthesis of novel indolizine, diazepinoindolizine and pyrimidoindolizine derivatives as potent and selective anticancer agents. Res Chem Intermed 2015;41:9687–701.

[16] Shawky AM, Ibrahim NA, Abourehab MAS, et al. Pharmacophore-based virtual screening, synthesis, biological evaluation, and molecular docking study of novel pyrrolizines bearing urea/thiourea moieties with potential cytotoxicity and CDK inhibitory activities. J Enzyme Inhib Med Chem 2021;36:15–33. 33103497 10.1080/14756366.2020.1837124 PMC7594867

[17] Gouda AM, Abdelazeem AH, Abdalla AN, Ahmed M. Pyrrolizine-5-carboxamides: exploring the impact of various substituents on anti-inflammatory and anticancer activities. Acta Pharm 2018;68:251–73. 31259695 10.2478/acph-2018-0026

[18] Marzouk AA, Abdel-Aziz SA, Abdelrahman KS, et al. Design and synthesis of new 1,6-dihydropyrimidin-2-thio derivatives targeting VEGFR-2: molecular docking and antiproliferative evaluation. Bioorg Chem 2020;102:104090. 32683176 10.1016/j.bioorg.2020.104090

[19] Gomaa HAM, El-Sherief HAM, Hussein S, et al. Novel 1,2,4-triazole derivatives as apoptotic inducers targeting p53: synthesis and antiproliferative activity. Bioorg Chem 2020;105:104369. 33091670 10.1016/j.bioorg.2020.104369

[20] Elsayed MSA, El-Araby ME, Serya RAT, et al. Structure-based design and synthesis of novel pseudosaccharine derivatives as antiproliferative agents and kinase inhibitors. Eur J Med Chem 2013;61:122–31. 23063746 10.1016/j.ejmech.2012.09.039

[21] Wang Y, Chen Y, Cheng X, et al. Design, synthesis and biological evaluation of pyrimidine derivatives as novel CDK2 inhibitors that induce apoptosis and cell cycle arrest in breast cancer cells. Bioorg Med Chem 2018;26:3491–501. 29853338 10.1016/j.bmc.2018.05.024

[22] Sonawane V, Mohd Siddique MU, Jadav SS, et al. Cink4T, a quinazolinone-based dual inhibitor of Cdk4 and tubulin polymerization, identified via ligand-based virtual screening, for efficient anticancer therapy. Eur J Med Chem 2019;165:115–32. 30665142 10.1016/j.ejmech.2019.01.011

[23] Guan Q, Cong L, Wang Q, et al. Activated carbon/Brønsted acid-promoted aerobic benzylic oxidation under “on-water” condition: green and efficient synthesis of 3-benzoylquinoxalinones as potent tubulin inhibitors. Eur J Med Chem 2020;186:111894. 31787361 10.1016/j.ejmech.2019.111894

[24] Morris GM, Huey R, Lindstrom W, et al. AutoDock4 and AutoDockTools4: automated docking with selective receptor flexibility. J Comput Chem 2009;30:2785–91. 19399780 10.1002/jcc.21256 PMC2760638

[25] Baumli S, Hole AJ, Noble MEM, Endicott JA. The CDK9 C-helix exhibits conformational plasticity that may explain the selectivity of CAN508. ACS Chem Biol 2012;7:811–6. 22292676 10.1021/cb2004516 PMC3355656

[26] Stamos J, Sliwkowski MX, Eigenbrot C. Structure of the epidermal growth factor receptor kinase domain alone and in complex with a 4-anilinoquinazoline inhibitor. J Biol Chem 2002;277:46265–72. 12196540 10.1074/jbc.M207135200

[27] Zhao B, Smallwood A, Yang J, et al. Modulation of kinase-inhibitor interactions by auxiliary protein binding: crystallography studies on Aurora A interactions with VX-680 and with TPX2. Protein Sci 2008;17:1791–7. 18662907 10.1110/ps.036590.108 PMC2548374

[28] Gaspari R, Prota AE, Bargsten K, et al. Structural basis of cis- and trans-combretastatin binding to tubulin. Chem 2017;2:102–13.

[29] Abdelbaset MS, Abdelrahman MH, Bukhari SNA, et al. Design, synthesis, and biological evaluation of new series of pyrrol-2(3H)-one and pyridazin-3(2H)-one derivatives as tubulin polymerization inhibitors. Bioorg Chem 2021;107:104522. 33317838 10.1016/j.bioorg.2020.104522

[30] Almalki FA, Gouda AM, Bin AM, Almehmadi OM. Profens: a comparative molecular docking study into cyclooxygenase-1/2. Drug Invent Today 2019;11:480–7.

[31] Gouda AM, Almalki FA. Carprofen: a theoretical mechanistic study to investigate the impact of hydrophobic interactions of alkyl groups on modulation of COX-1/2 binding selectivity. SN Appl Sci 2019;1:332.

[32] BIOVIA. Dassault Systems, Discovery Studio Visualizer, v16.1.0.15350. San Diego: Dassault Systems; 2016.

[33] Sheng H, Shao J, Kirkland SC, et al. Inhibition of human colon cancer cell growth by selective inhibition of cyclooxygenase-2. J Clin Invest 1997;99:2254–9. 9151799 10.1172/JCI119400 PMC508057

[34] Agarwal B, Swaroop P, Protiva P, Raj SV, et al. Cox-2 is needed but not sufficient for apoptosis induced by Cox-2 selective inhibitors in colon cancer cells. Apoptosis 2003;8:649–54. 14739610 10.1023/A:1026199929747

[35] Gomha SM, Riyadh SM, Mahmmoud EA, Elaasser MM. Synthesis and anticancer activities of thiazoles, 1,3-thiazines, and thiazolidine using chitosan-grafted-poly(vinylpyridine) as basic catalyst. Heterocycles 2015;91:1227–43.

[36] Gomha SM, Riyadh SM, Mahmmoud EA, Elaasser MM. Synthesis and anticancer activity of arylazothiazoles and 1,3,4-thiadiazoles using chitosan-grafted-poly(4-vinylpyridine) as a novel copolymer basic catalyst. Chem of Heterocycl Compd 2015;51:1030–8.

[37] Vilanova C, Díaz-Oltra S, Murga J, Falomir E, et al. Design and synthesis of pironetin analogue/colchicine hybrids and study of their cytotoxic activity and mechanisms of interaction with tubulin. J Med Chem 2014;57:10391–403. 25426924 10.1021/jm501112q

[38] Kumar A, Singh B, Sharma PR, et al. A novel microtubule depolymerizing colchicine analogue triggers apoptosis and autophagy in HCT-116 colon cancer cells. Cell Biochem Funct 2016;34:69–81. 26919061 10.1002/cbf.3166

[39] Krystof V, Cankar P, Frysova I, et al. 4-Arylazo-3,5-diamino-1H-pyrazole CDK inhibitors: SAR study, crystal structure in complex with CDK2, selectivity, and cellular effects. J Med Chem 2006;49:6500–9. 17064068 10.1021/jm0605740

[40] Li Y, Zhang J, Gao W, et al. Insights on structural characteristics and ligand binding mechanisms of CDK2. Int J Mol Sci 2015;16:9314–40. 25918937 10.3390/ijms16059314 PMC4463590

[41] Shawky AM, Abdalla AN, Ibrahim NA, et al. Discovery of new pyrimidopyrrolizine/indolizine-based derivatives as P-glycoprotein inhibitors: design, synthesis, cytotoxicity, and MDR reversal activities. Eur J Med Chem 2021;218:113403. 33823396 10.1016/j.ejmech.2021.113403

