# Supplementary material for: Novel pyrrolizines bearing 3,4,5-trimethoxyphenyl moiety: design, synthesis, molecular docking, and biological evaluation as potential multi-target cytotoxic agents
Source: J Enzyme Inhib Med Chem. 2021 Jun 21;36(1):1313–33. doi: 10.1080/14756366.2021.1937618 (PMC8221158; doi:10.1080/14756366.2021.1937618)
Supplement: Supplemental Material [file IENZ_A_1937618_SM0547.pdf]

Novel pyrrolizines bearing 3,4,5-trimethoxyphenyl moiety: design, synthesis, molecular docking, and biological evaluation as potential multi-target cytotoxic agents

Ahmed M. Shawky[1,2], Nashwa A. Ibrahim[3,4], Ashraf N. Abdalla[5,6], Mohammed A. S. Abourehab[7,8], Ahmed M. Gouda[3,4,*]

[1]Science and Technology Unit (STU), Umm Al-Qura University, Makkah 21955, Saudi Arabia

[2]Central Laboratory for Micro-analysis, Minia University, Minia 61519, Egypt

[3]Department of Pharmaceutical Chemistry, Faculty of Pharmacy, Umm Al-Qura University, Makkah 21955, Saudi Arabia

[4]Medicinal chemistry department, Faculty of pharmacy, Beni-Suef University, Beni-Suef 62514, Egypt

[5]Department of Pharmacology and Toxicology, Faculty of Pharmacy, Umm Al-Qura University, Makkah 21955, Saudi Arabia

[6]Departmentof Pharmacology and Toxicology, Medicinal and Aromatic plants research institute, National center for research, Khartoum 2404, Sudan

[7]Department of Pharmaceutics, Faculty of Pharmacy, Umm Al-Qura University, Makkah 21955, Saudi Arabia

[8]Department of Pharmaceutics, Faculty of Pharmacy, Minia University, Minia 61519, Egypt

---

***Correspondence: Ahmed M. Gouda:** Department of Medicinal Chemistry, Faculty of Pharmacy, Beni-Suef University, Beni-Suef 62514, Egypt.

Scopus ID: 26321547200

ORCID: 0000-0003-4527-8885

Tel.: (002)-01126897483

Fax: (002)-082-2162133

E-mail address: ahmed.gouda@pharm.bsu.edu.eg or amsaid@uqu.edu.sa

Table of contents:

		Page No
Prelimenart docking study into CDK-2/EGFR/tubulin	**Table S1.** Results of the preliminary docking study (B1-B26)	4
	**Table S2.** Results of the preliminary docking study (B27-B52)	5

Spectral data	Comp. No	Fig. No	Page No
IR spectra	**15a**	S1	7
	**15b**	S2	8
	**15c**	S3	9
	**15d**	S4	10
	**15e**	S5	11
	**20**	S6	12
	**16a**	S7	13
	**16b**	S8	14
	**16c**	S9	15
	**16d**	S10	16
	**16e**	S11	17
	**21**	S12	18
H-NMR, C^{13}-NMR and DEPT C^{135} spectra	**15a**	S13-21	20-28
	**15b**	S22-29	29-36
	**15c**	S30-35	37-42
	**15d**	S36-43	43-50
	**15e**	S44-48	51-55
	**20**	S49-54	56-61
	**16a**	S55-62	62-69
	**16b**	S63-70	70-77
	**16c**	S71-80	78-87
	**16d**	S81-88	88-95
	**16e**	S89-95	96-102
	**21**	S96-105	103-112
Mass spectra	**15a**	S106-111	114-119
	**15b**	S112-115	120-123
	**15c**	S116	124
	**15d**	S117-123	125-131
	**15e**	S124	132
	**20**	S125	133
	**16a**	S126-128	134-136
	**16b**	S129-131	137-139
	**16c**	S132	140
	**16d**	S133-139	141-147
	**16e**	S140	148
	**21**	S141	149

Table of contents (continue)

Title	Fig./Tab No	Page No
2/3D binding mode of compound **16b** into EGFR.	S142	150
2/3D binding mode of compound **16d** into EGFR.	S143	150
2/3D binding mode of compound erlotinib into EGFR.	S144	151
Results of the docking study of compounds **16a**,**b**,**d** into Aurora A	Table S3	152
Results of the docking study of the new compounds into tubulin protein	Table S4	153
List of abbreviations	-	154

Preliminary docking study

The preliminary molecular docking the new compounds were carried out by AutoDock 4.2. the crystal structure of CDK2 bound to CAN508 (pdb code: 3TNW), EGFR bound to erlotinib (pdb code: 1M17), and tubulin bound to C-A4, was obtained from protein data bank (http://www.rcsb.org/pdb). Ligand and protein files were prepared according to the previous report. Docking and grid parameter files were prepared according to the previous reports. The top ten protein-ligand complexes were scored. The results including binding modes, affinities, and interactions of the best fit conformations of the new compounds were visualized using Discovery Studio Visualizer (v16.1.0.15350).

Results of the preliminary docking study

Table S1. Results of the preliminary docking study into CDK-2 (pdb code: 3TNW), EGFR (pdb code: 1M17), and tubulin (pdb code: 5LYJ), synthetic accessibility, and drug likeness score study of compounds B1-B26

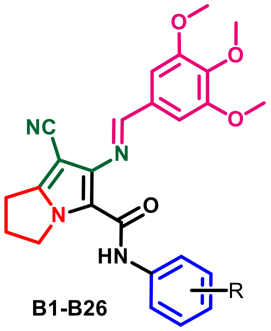

B1-B26

δ	Code	R	Tubulin		CDK-2		EGFR		*SAS* [d]	*DLS* [e]
			ΔG_b [a]	*K_i* [b]	*ΔG_b* [a]	*K_i* [b]	*ΔG_b* [a]	*K_i* [b]		
**0**	**B1**	H	-6.97	7.770 µM	-8.06	1.24 µM	-8.85	324.28 nM	3.68	0.39
+ ve (electron withdrawing substituents)	**B2**	4-F	-4.13	946.88 µM	nt	nt	nt	nt	3.67	0.72
	**B3**	3-F	-5.94	44.09 µM	nt	nt	nt	nt	3.69	0.36
	**B4**	2-F	-4.92	247.36 µM	nt	nt	nt	nt	3.71	0.42
	**B5**	4-Cl	-6.46	18.41 µM	-8.66	445.63 nM	-9.03	239.38 nM	3.67	0.83
	**B6**	3-Cl	-4.17	879.09 µM	nt	nt	nt	nt	3.68	0.42
	**B7**	2-Cl	-3.51	2.69 mM	nt	nt	nt	nt	3.70	0.56
	**B8**	4-Br	-8.07	1.21 µM	-8.52	570.62 nM	-9.10	212.73 nM	3.70	0.55
	**B9**	3-Br	-6.74	11.73 µM	nt	nt	nt	nt	3.75	0.20
	**B10**	2-Br	-4.95	236.26 nM	nt	nt	nt	nt	3.71	0.30
	**B11**	4-I	-6.72	11.86 µM	-8.54	545.73 nM	-9.23	170.24 nM	3.77	0.79
	**B12**	3-I	-6.13	31.89 µM	nt	nt	nt	nt	3.77	0.42
	**B13**	2-I	+0.63	-	nt	nt	nt	nt	3.76	0.52
	**B14**	3,5-diCl	-7.28	4.65 µM	-8.94	277.94 nM	-9.06	228.92 nM	3.70	0.46
	**B15**	2,4-diCl	-4.63	406.26 µM	nt	nt	nt	nt	3.72	0.46
	**B16**	4-COOH	-3.36	3.44 mm	nt	nt	nt	nt	3.79	0.94
	**B17**	4-CF3	-3.90	1.37 mM	nt	nt	nt	nt	3.81	0.44
- ve (electron donating substituents)	**B18**	4-CH_3	-6.35	22.16 µM	-8.44	650.86 nM	-8.78	363.84 nM	3.79	0.49
	**B19**	3-CH_3	-3.31	7.76 mM	nt	nt	nt	nt	3.79	0.37
	**B20**	2-CH_3	-2.95	6.93 mM	nt	nt	nt	nt	3.80	0.52
	**B21**	3,5-diMe	-3.75	1.87 mM	nt	nt	nt	nt	3.91	0.57
	**B22**	2,4-diMe	-5.62	75.51 µM	nt	nt	nt	nt	3.92	0.25
	**B23**	4-C_2H_5	-5.41	107.34 µM	nt	nt	nt	nt	3.90	0.80
	**B24**	4-OCH_3	-6.63	13.83 µM	-9.11	209.91 nM	-8.42	675.7 nM	3.83	0.66
	**B25**	3-OCH_3	-4.02	1.14 mM	nt	nt	nt	nt	3.87	0.34
	**B26**	2-OCH_3	-5.31	128.82 µM	nt	nt	nt	nt	3.87	0.37

[a] *Binding free energy (kcal/mol);* [b] *Inhibition constant; the study was performed by AutoDock 4.2; the protein molecules were used as rigid molecules while ligands (A1-28) were used as flexible molecules;* [d] *Synthetic accessibility Scores were determined used SwissADME (http://www.swissadme.ch/,), 1(very easy) to 10 (very difficult);* [e] *DLS, drug-likeness score was calculated using* Molsoft (http://molsoft.com/mprop/), nt, not tested. Note,

The compounds selected for the synthesis are highlighted in yellow color

Table S2. Results of the preliminary docking study into CDK-2 (pdb code: 3TNW), EGFR (pdb code: 1M17), and tubulin (pdb code: 5LYJ), synthetic accessibility, and drug likeness score study of compounds B27-B52

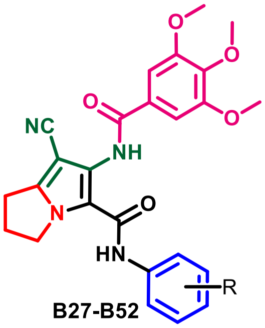

δ	Code	R	Tubulin		CDK-2		EGFR		*SAS* [d]	*DLS* [e]
			ΔG$_b$ [a]	*K*$_i$ [b]	*ΔG*$_b$ [a]	*K*$_i$ [b]	*ΔG*$_b$ [a]	*K*$_i$ [b]		
**0**	**B27**	H	-8.80	347.84 nM	-9.21	0.178 µM	-9.07	223.89 nM	3.66	0.50
**+ ve (electron withdrawing substituents)**	**B38**	4-F	-6.61	14.36 µM	nt	nt	nt	nt	3.66	0.76
	**B29**	3-F	-8.13	1.1 µM	nt	nt	nt	nt	3.67	0.43
	**B30**	2-F	-6.65	13.41 µM	nt	nt	nt	nt	3.69	0.41
	**B31**	4-Cl	-10.63	16.28 nM	-8.37	0.732 µM	-8.53	555.92 nM	3.65	0.87
	**B32**	3-Cl	-7.27	4.68 µM	nt	nt	nt	nt	3.66	0.48
	**B33**	2-Cl	-10.71	14.04 nM	nt	nt	nt	nt	3.68	0.54
	**B34**	4-Br	-11.87	1.99 nM	-9.31	150.82 nM	-9.39	131.08 nM	3.68	0.61
	**B35**	3-Br	-4.30	704.77 µM	nt	nt	nt	nt	3.75	0.29
	**B36**	2-Br	-5.61	77.32 µM	nt	nt	nt	nt	3.68	0.30
	**B37**	4-I	-4.16	895.19 µM	nt	nt	nt	nt	3.75	0.85
	**B38**	3-I	-8.17	1.03 µM	nt	nt	nt	nt	3.75	0.50
	**B39**	2-I	-10.15	36.48 nM	-8.52	564.23 nM	-9.08	220.95 nM	3.74	0.52
	**B40**	3,5-diCl	-3.67	2.04 mM	nt	nt	nt	nt	3.68	0.49
	**B41**	2,4-diCl	-8.09	1.17 µM	nt	nt	nt	nt	3.70	0.41
	**B42**	4-COOH	-3.44	3.02 mM	nt	nt	nt	nt	3.76	0.96
	**B43**	4-CF3	-0.17	746.99 mM	nt	nt	nt	nt	3.80	0.50
**- ve (electron donating substituents)**	**B44**	4-CH_3	-10.97	9.06 nM	-7.91	1.60 µM	-8.60	498.09 nM	3.77	0.59
	**B45**	3-CH_3	-9.3	152.47 nM	nt	nt	nt	nt	3.77	0.47
	**B46**	2-CH_3	-8.85	326.56 nM	nt	nt	nt	nt	3.78	0.51
	**B47**	3,5-diMe	-2.83	8.46 mM	nt	nt	nt	nt	3.89	0.61
	**B48**	2,4-diMe	-4.17	879.59 µM	nt	nt	nt	nt	3.89	0.22
	**B49**	4-C_2H_5	-7.27	4.71 µM	nt	nt	nt	nt	3.88	0.91
	**B50**	4-OCH_3	-9.08	222.49 nM	-8.71	411.8 nM	-9.11	210.87 nM	3.82	0.72
	**B51**	3-OCH_3	-7.88	1.68 µM	nt	nt	nt	nt	3.85	0.43
	**B52**	2-OCH_3	-7.21	5.17 µM	nt	nt	nt	nt	3.85	0.34

[a] *Binding free energy (kcal/mol);* [b] *Inhibition constant; the study was performed by AutoDock 4.2; the protein molecules were used as rigid molecules while ligands (A1-28) were used as flexible molecules;* [d] *Synthetic accessibility Scores were determined used SwissADME (*http://www.swissadme.ch/*,), 1(very easy) to 10 (very difficult);* [e] *DLS, drug-likeness score was calculated using* Molsoft (http://molsoft.com/mprop/), nt, not tested.

The compounds selected for the synthesis are highlighted in yellow color

IR Spectra of the new compounds

Infrared spectra (IR) were done using BRUKER TENSOR 37 spectrophotometer and absorption were expressed in wave number (cm^{-1}) using KBr Disk.

Fig. S1: **IR spectrum of compound 15a**

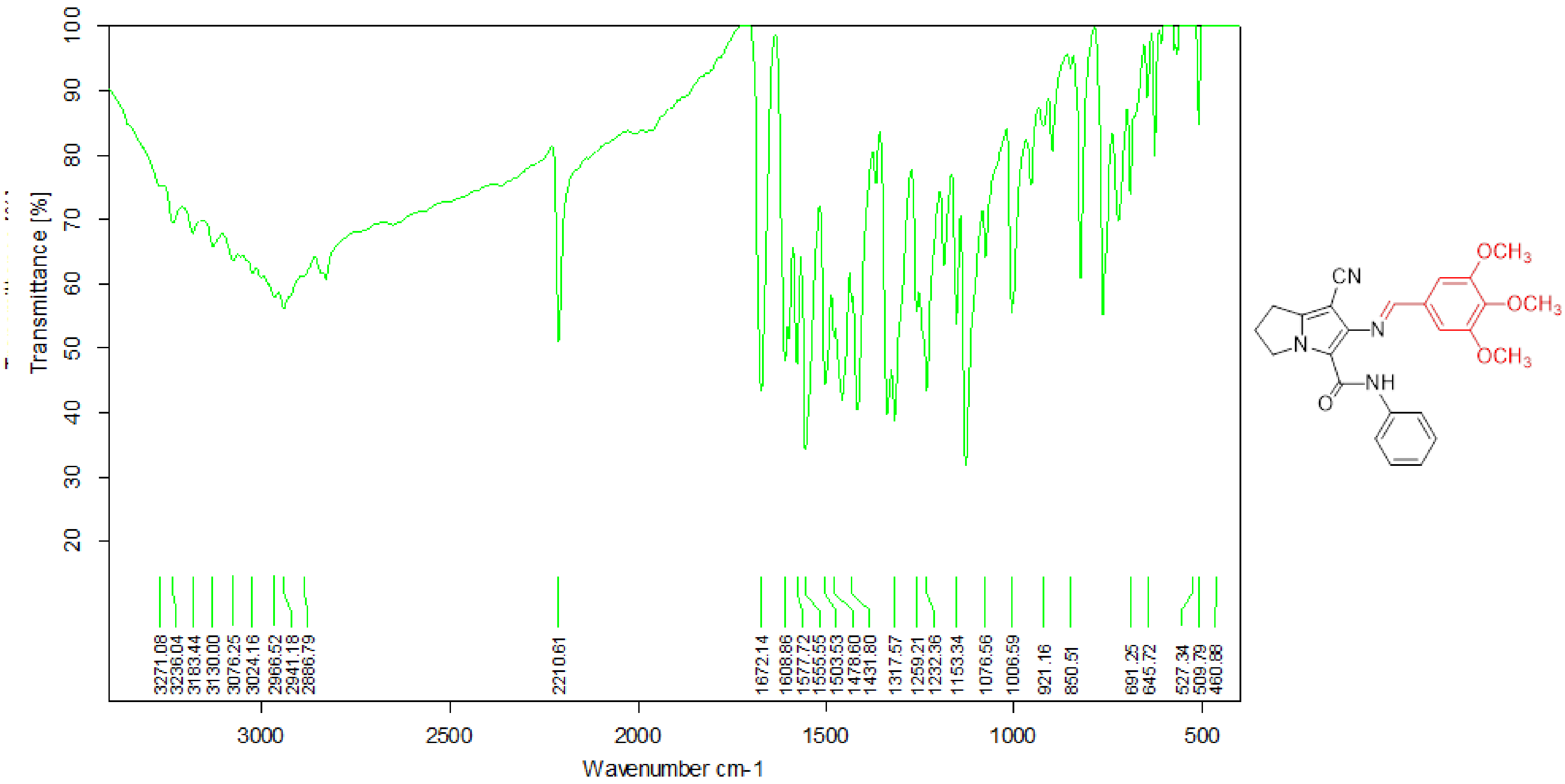

Fig. S2: **IR spectrum of compound 15b**

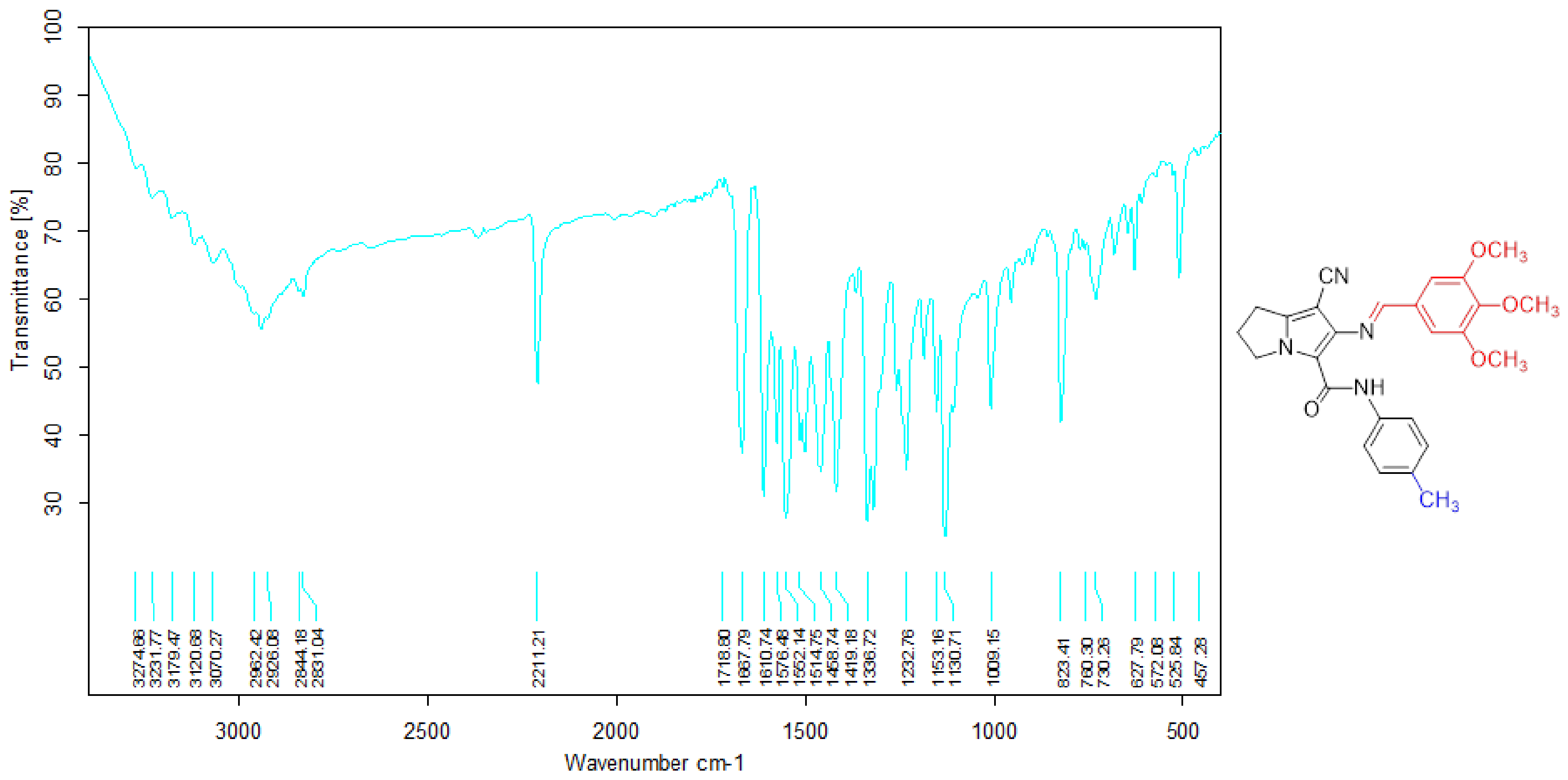

Fig. S3: **IR spectrum of compound 15c**

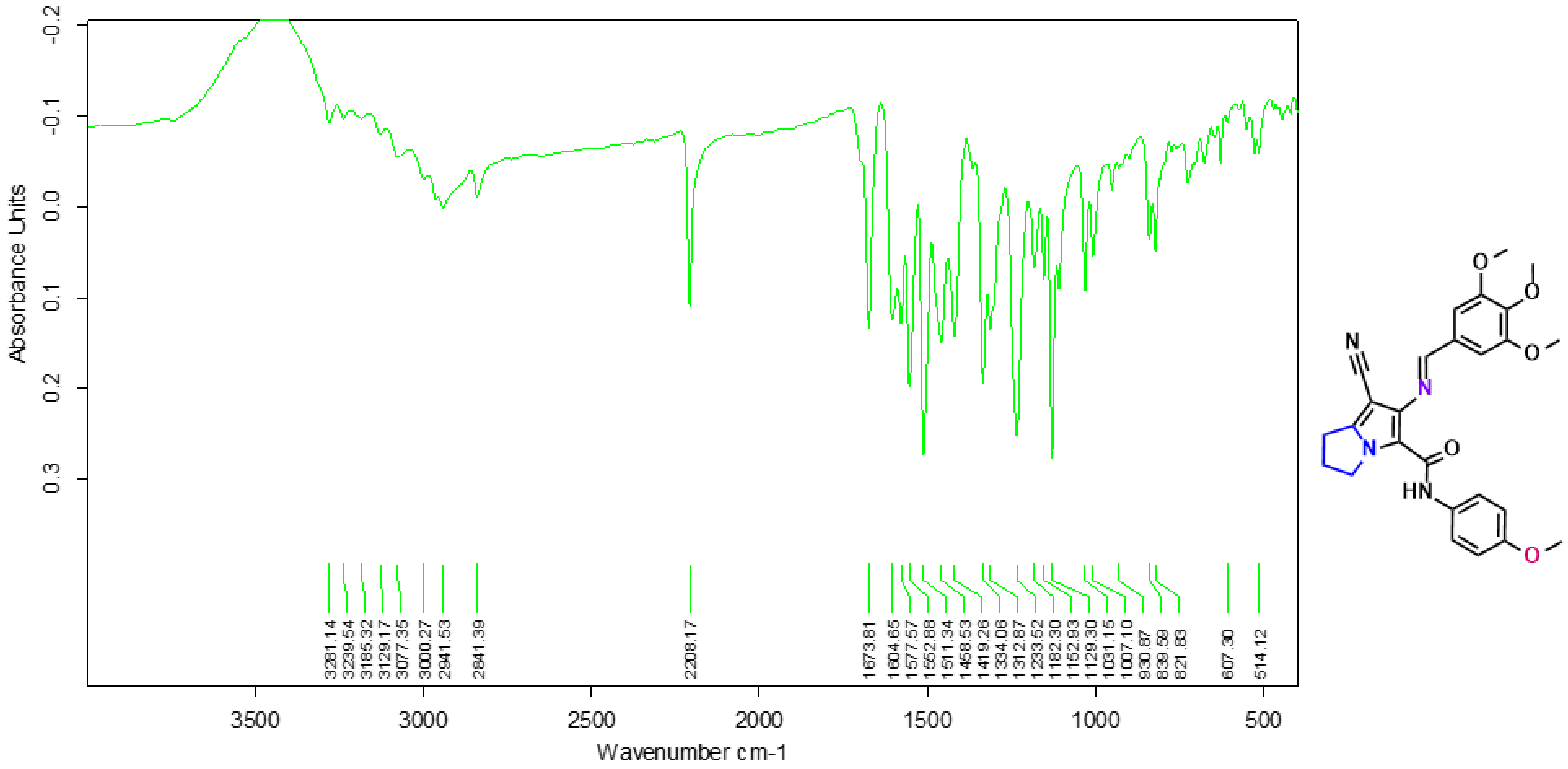

Fig. S4: **IR spectrum of compound 15d**

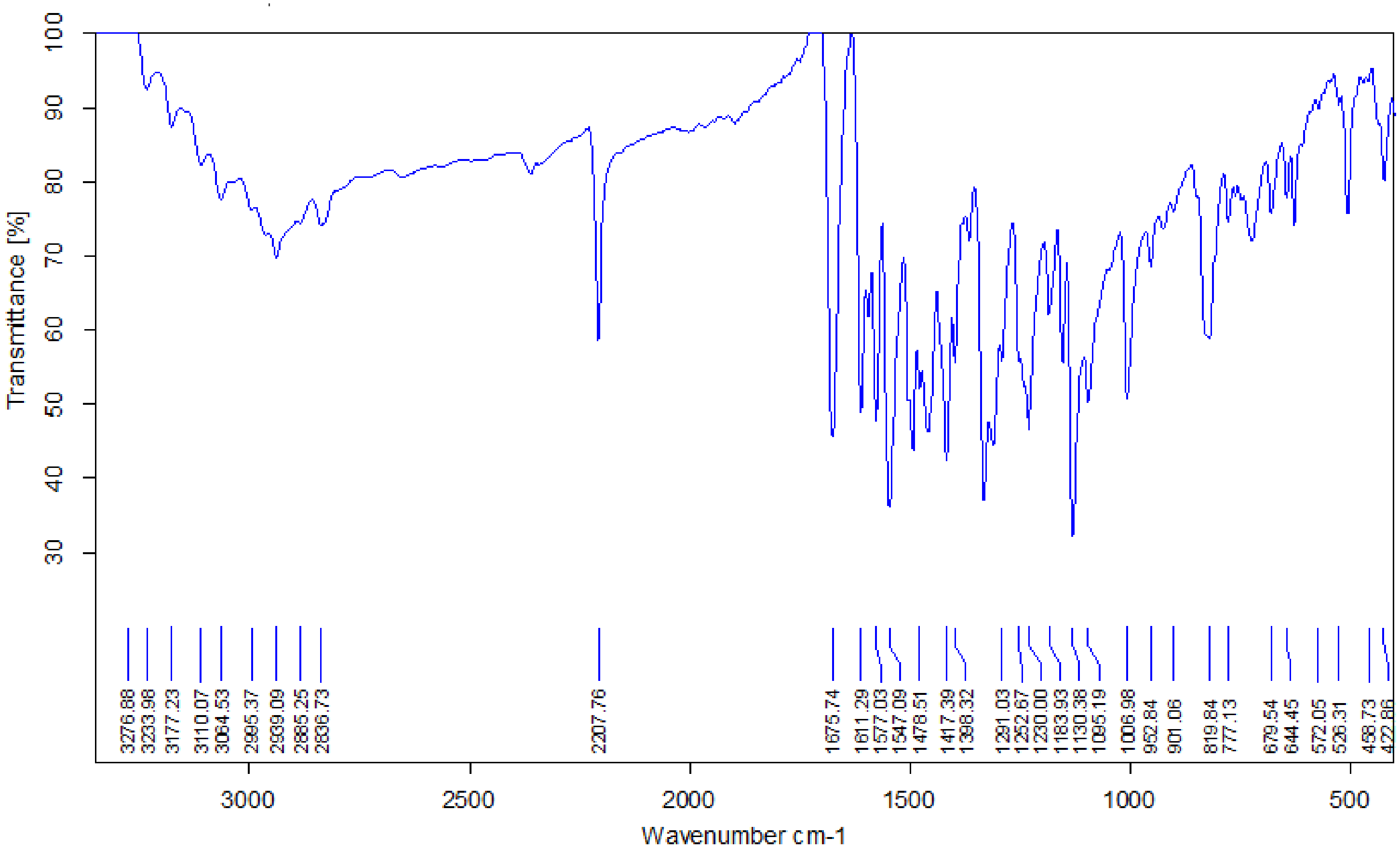

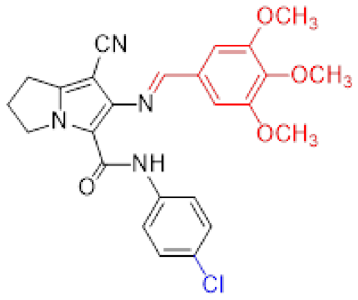

Fig. S5: **IR spectrum of compound 15e**

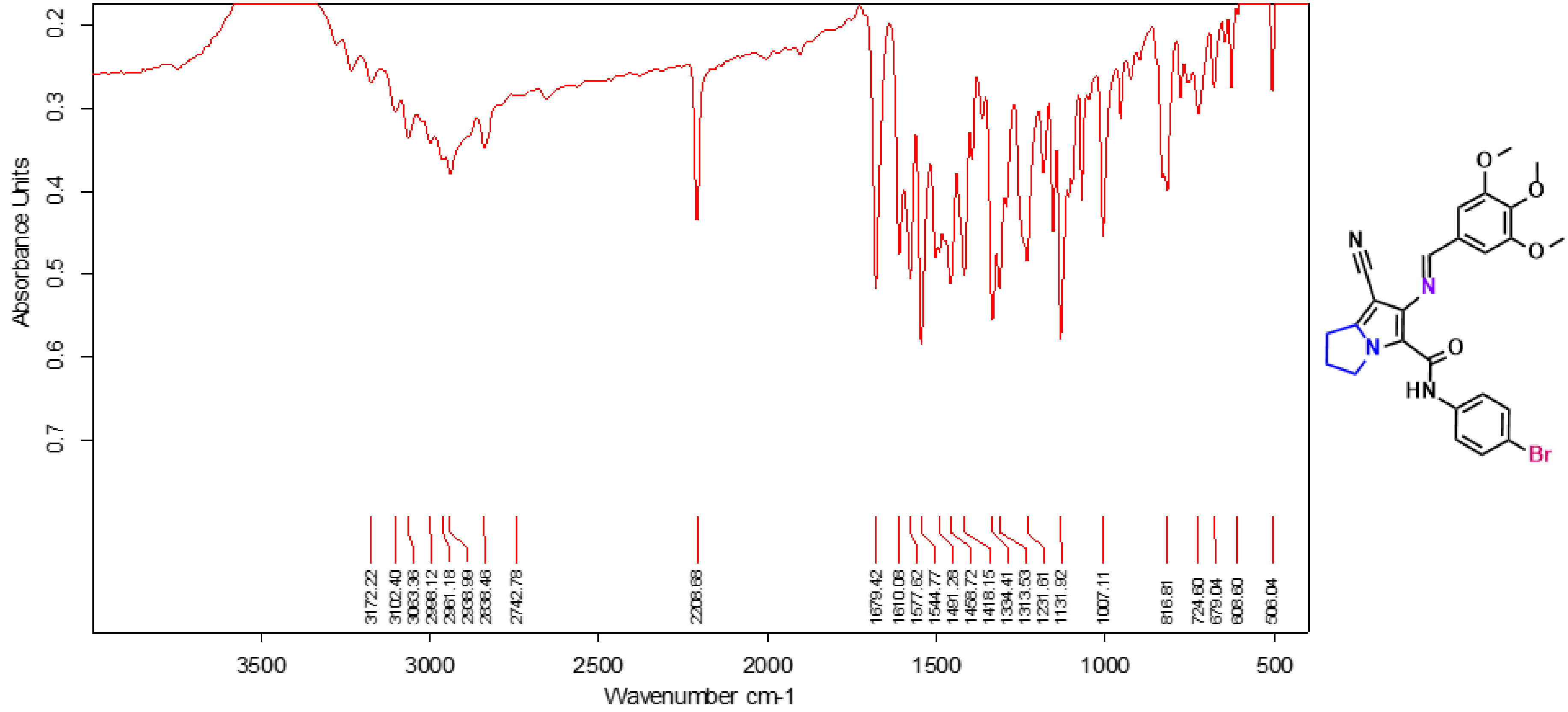

Fig. S6: **IR spectrum of compound 20**

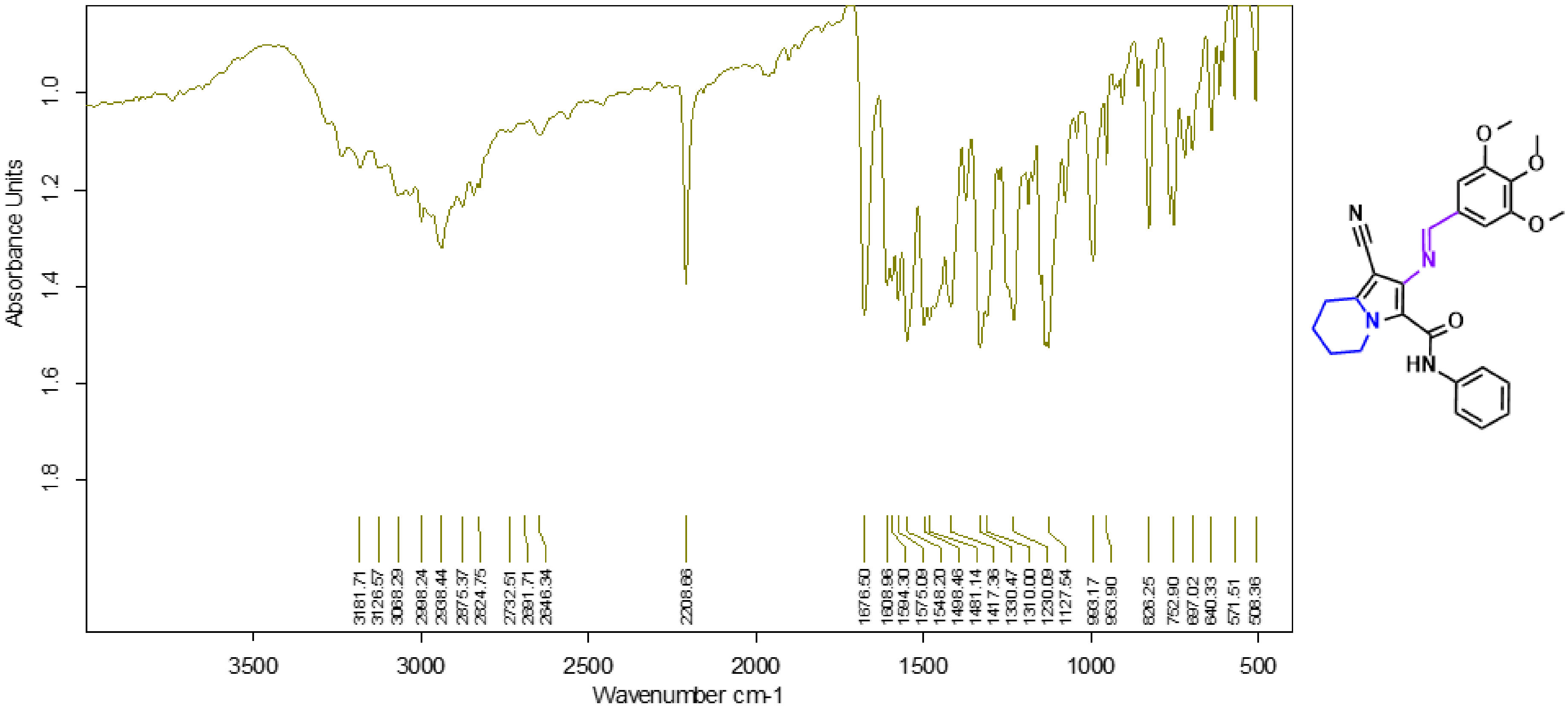

Fig. S7: **IR spectrum of compound 16a**

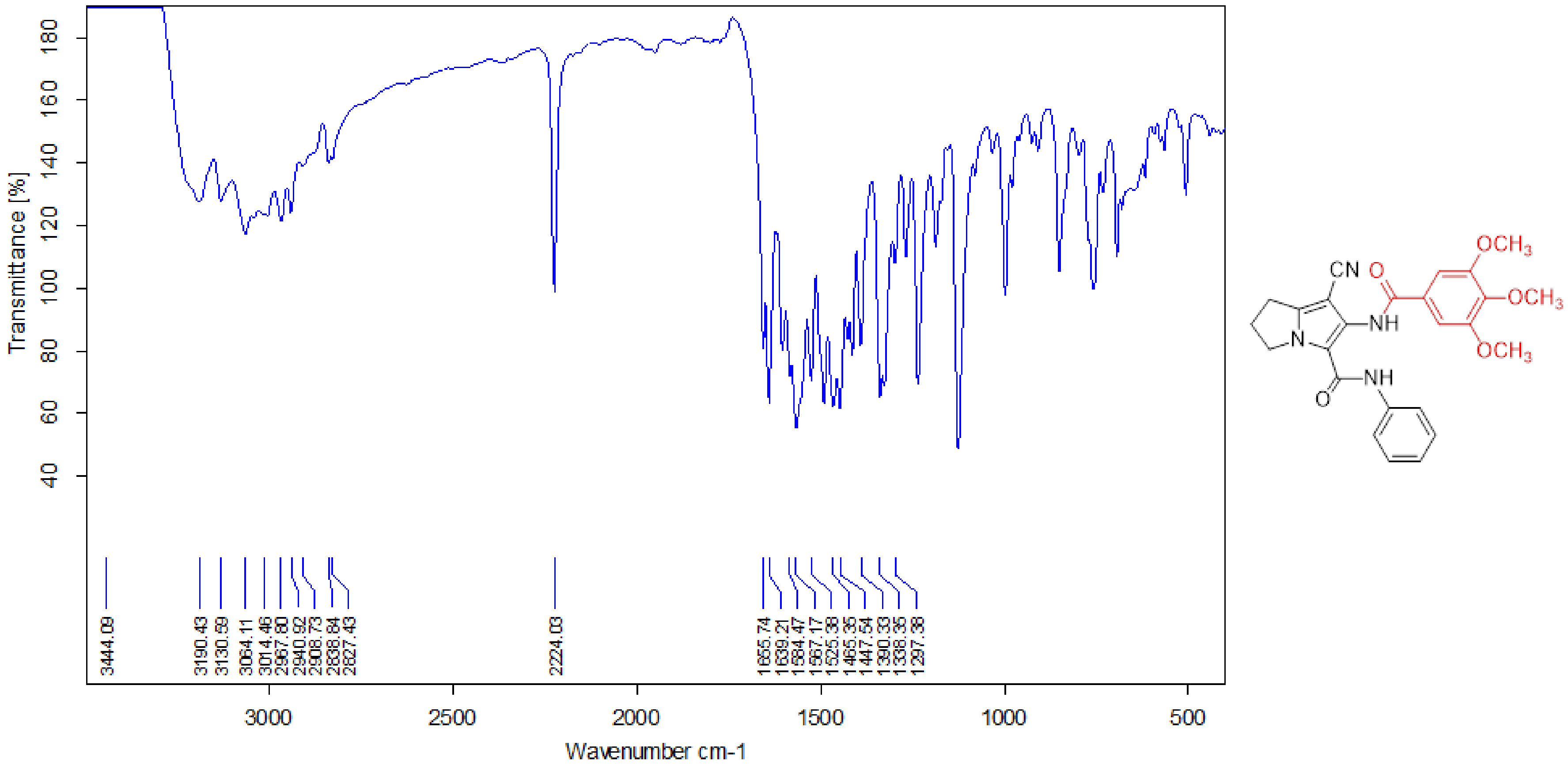

Fig. S8: **IR spectrum of compound 16b**

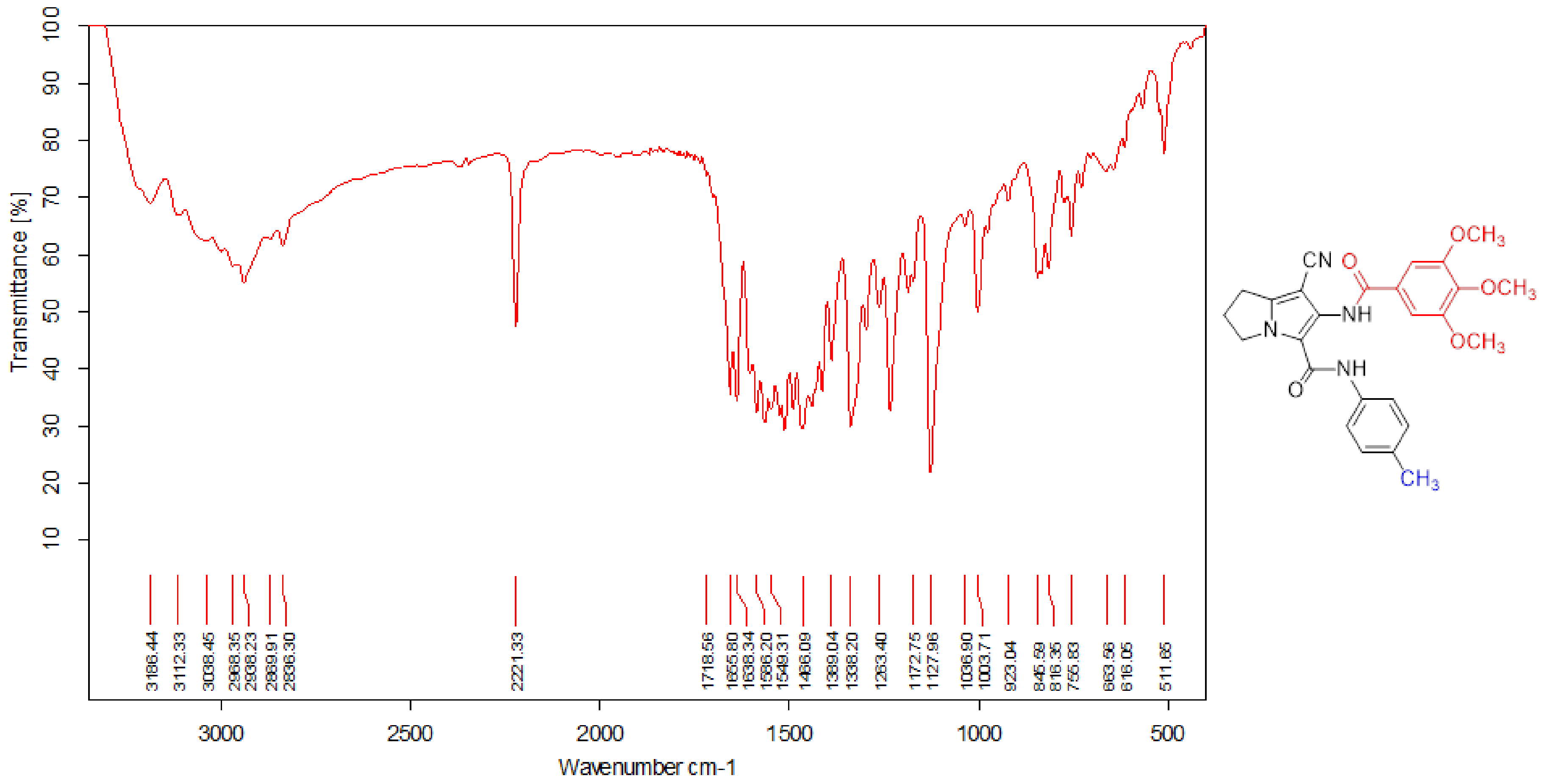

Fig. S9: **IR spectrum of compound 16c**

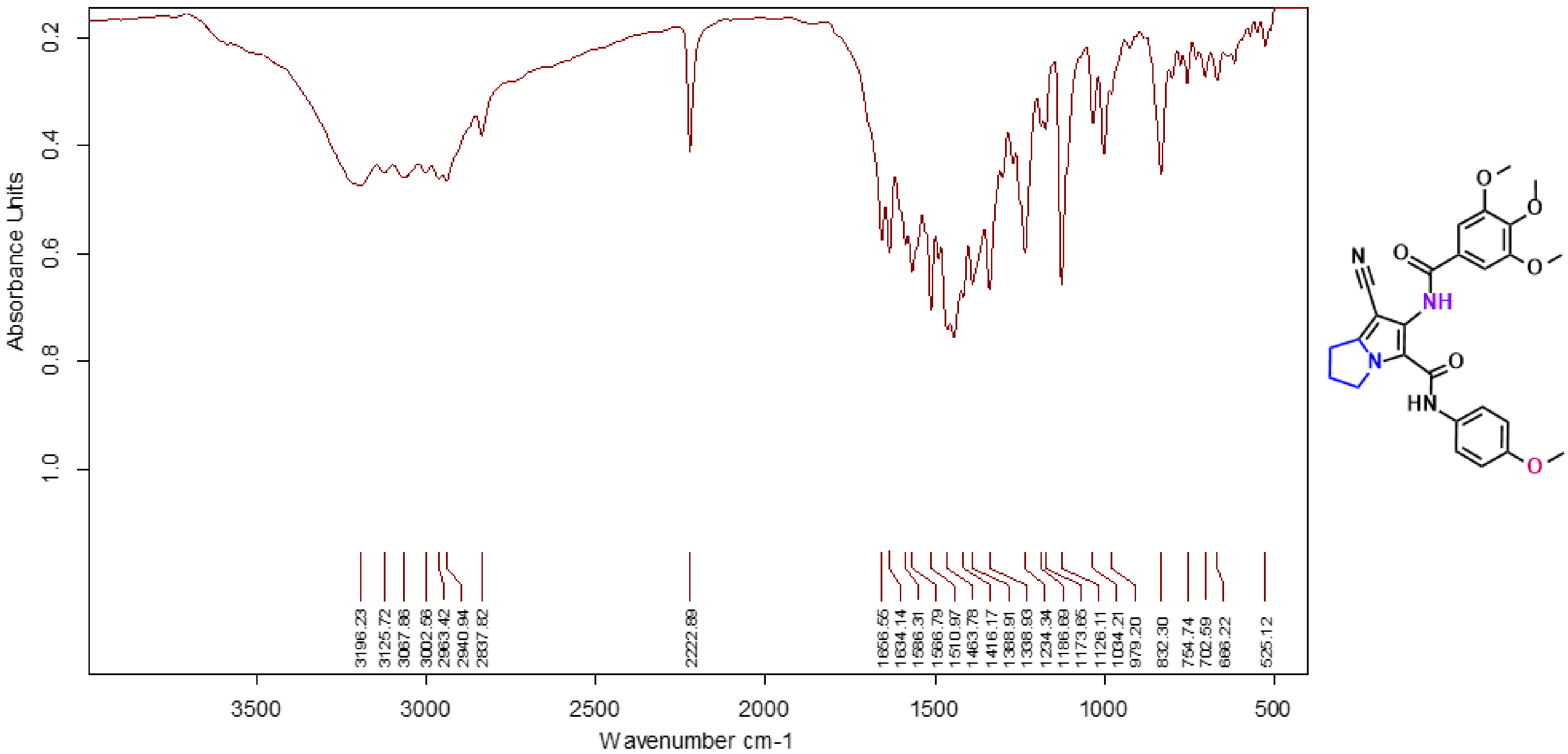

Fig. S10: IR spectrum of compound 16d

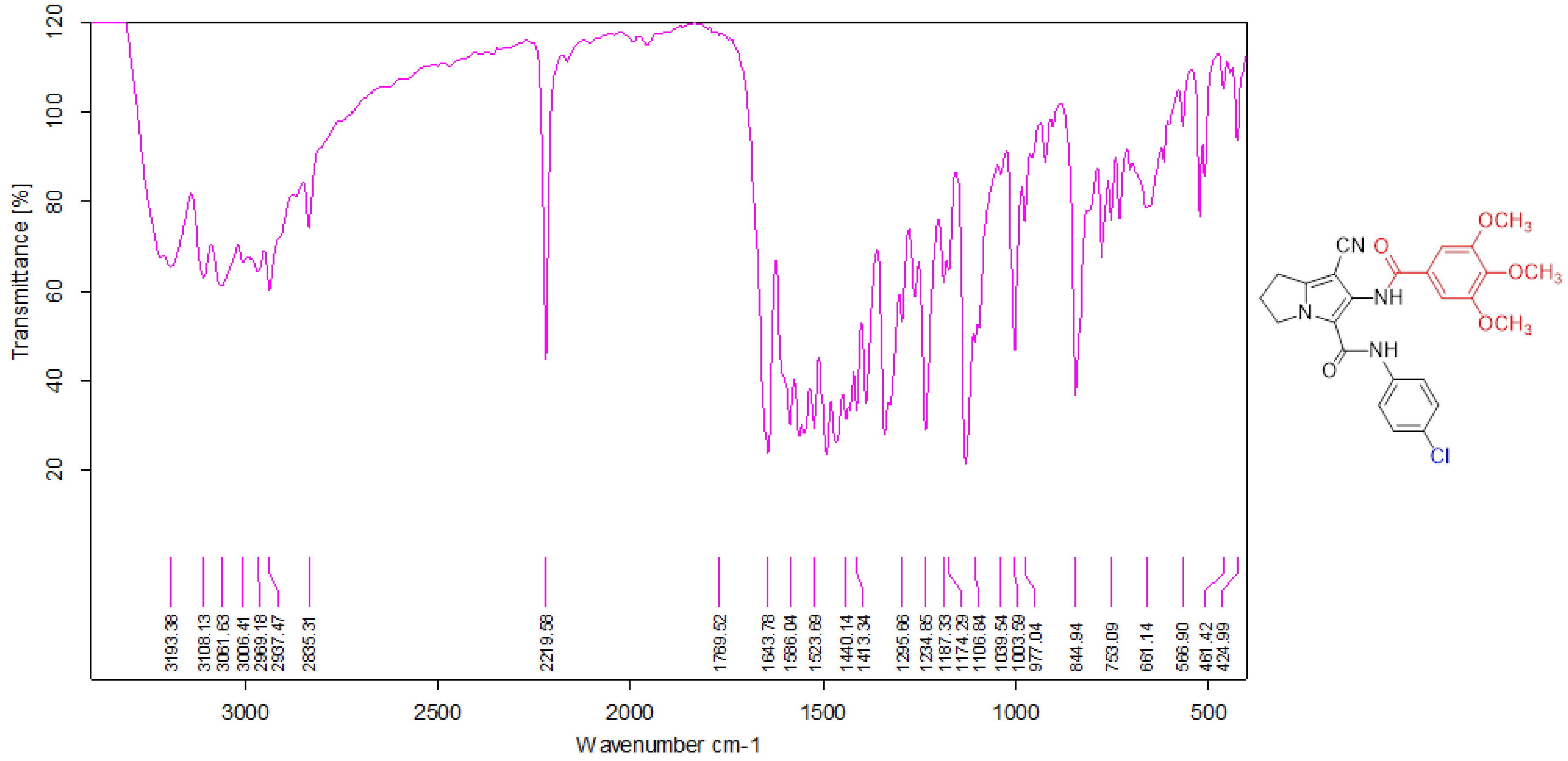

Fig. S11: **IR spectrum of compound 16e**

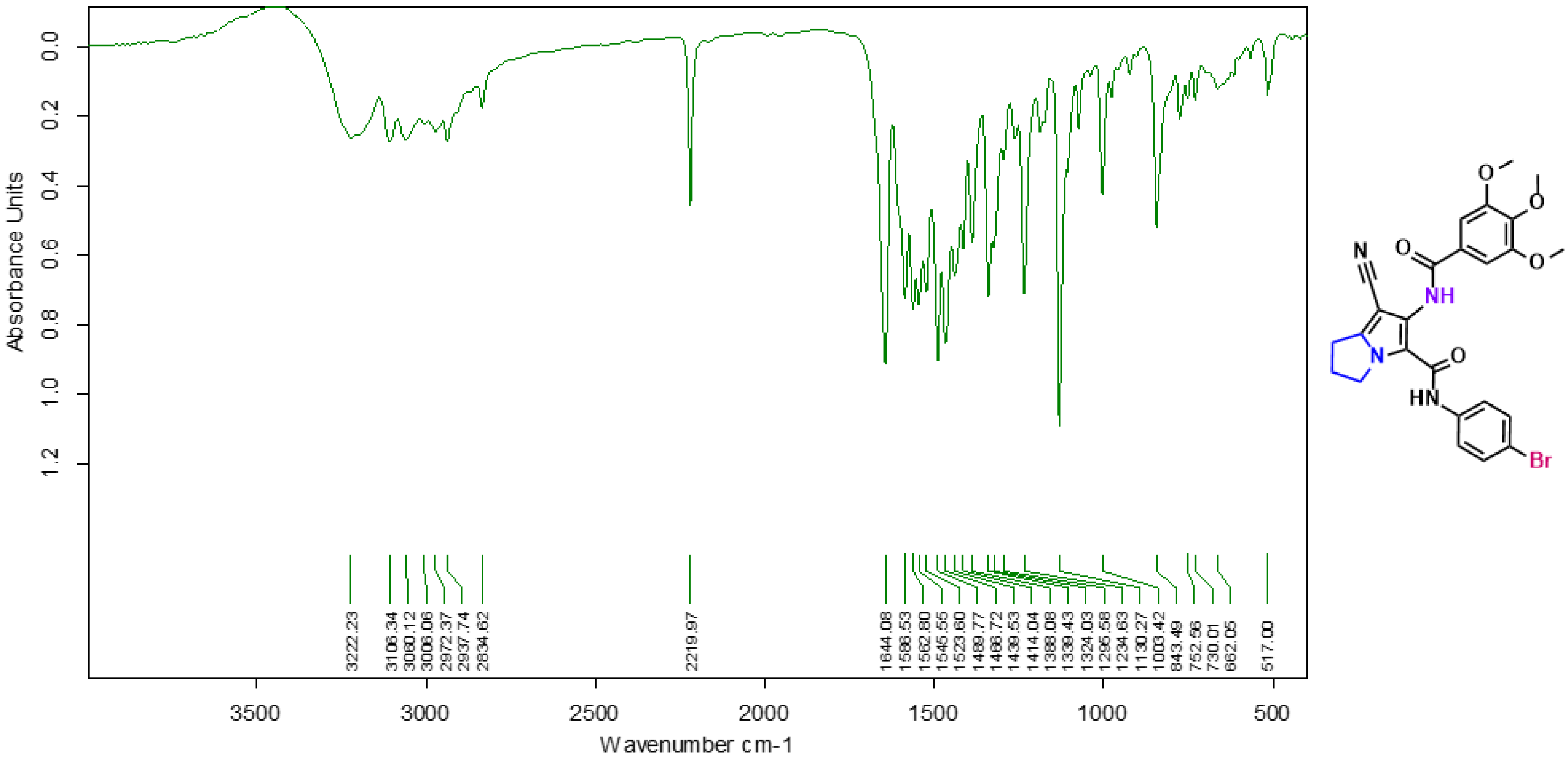

Fig. S12: **IR spectrum of compound 21**

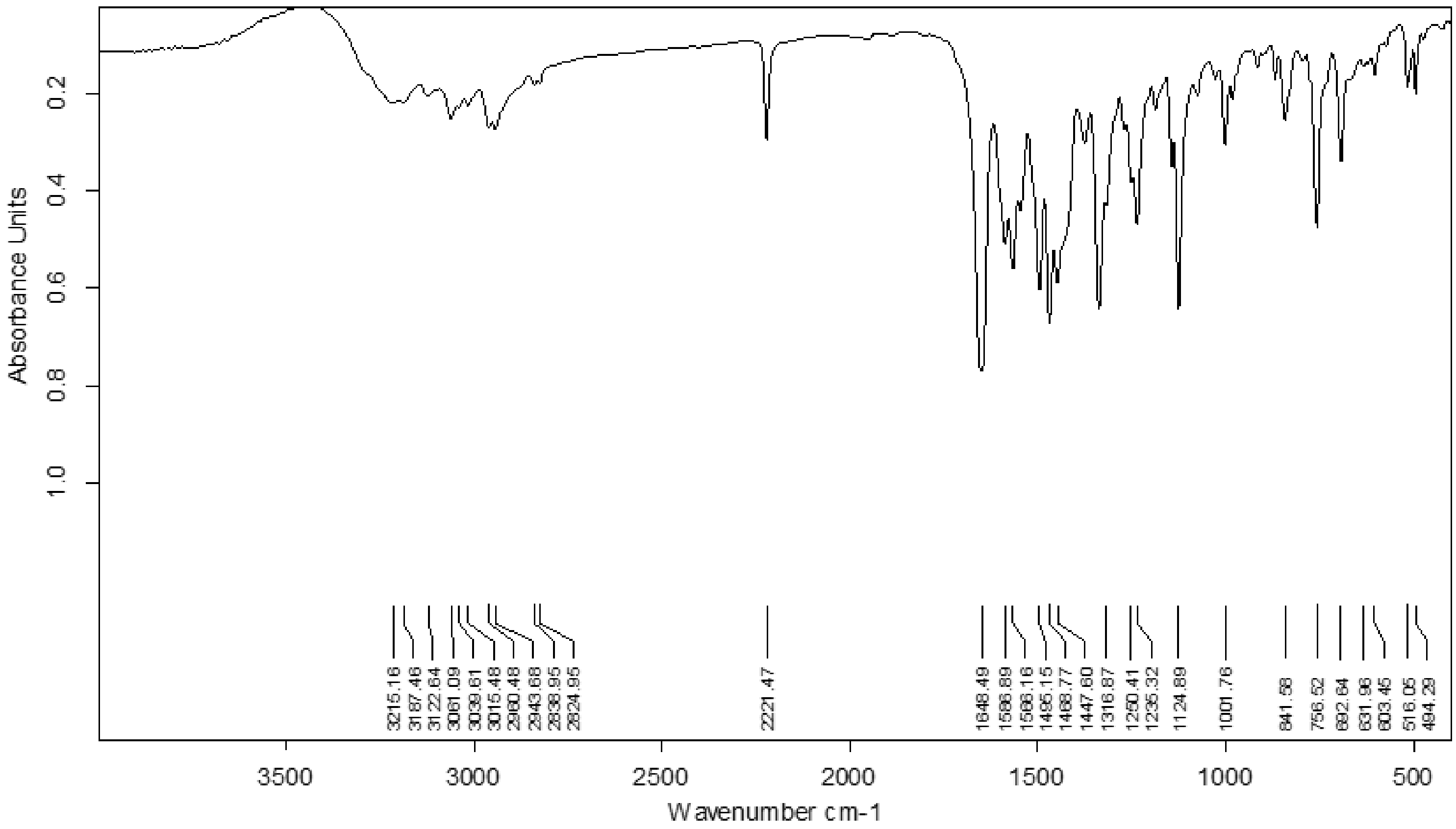

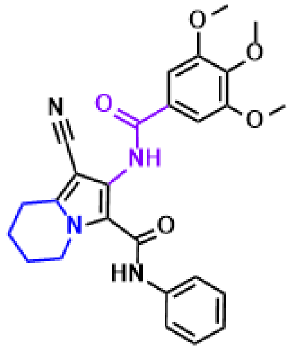

^{1}H-NMR, ^{13}C-NMR and DEPT C^{135} Spectra

^{1}H-NMR spectra were recorded on a BRUKER AVANCE III spectrometer (at the faculty of pharmacy, Umm Al-Qura University) at 500 MHz in the specified solvent, chemical shifts were reported on the δ (ppm) scale and were related to that of the solvent and *J* values are given in Hz. ^{13}C NMR and DEPT C^{135} spectra were obtained on a BRUKER AVANCE III at 125 MHz (at the faculty of pharmacy, Umm Al-Qura University).

Fig. S13: ^{1}H-NMR ($CDCl_3$, 500 MHz, δ ppm) spectrum of compound **15a**

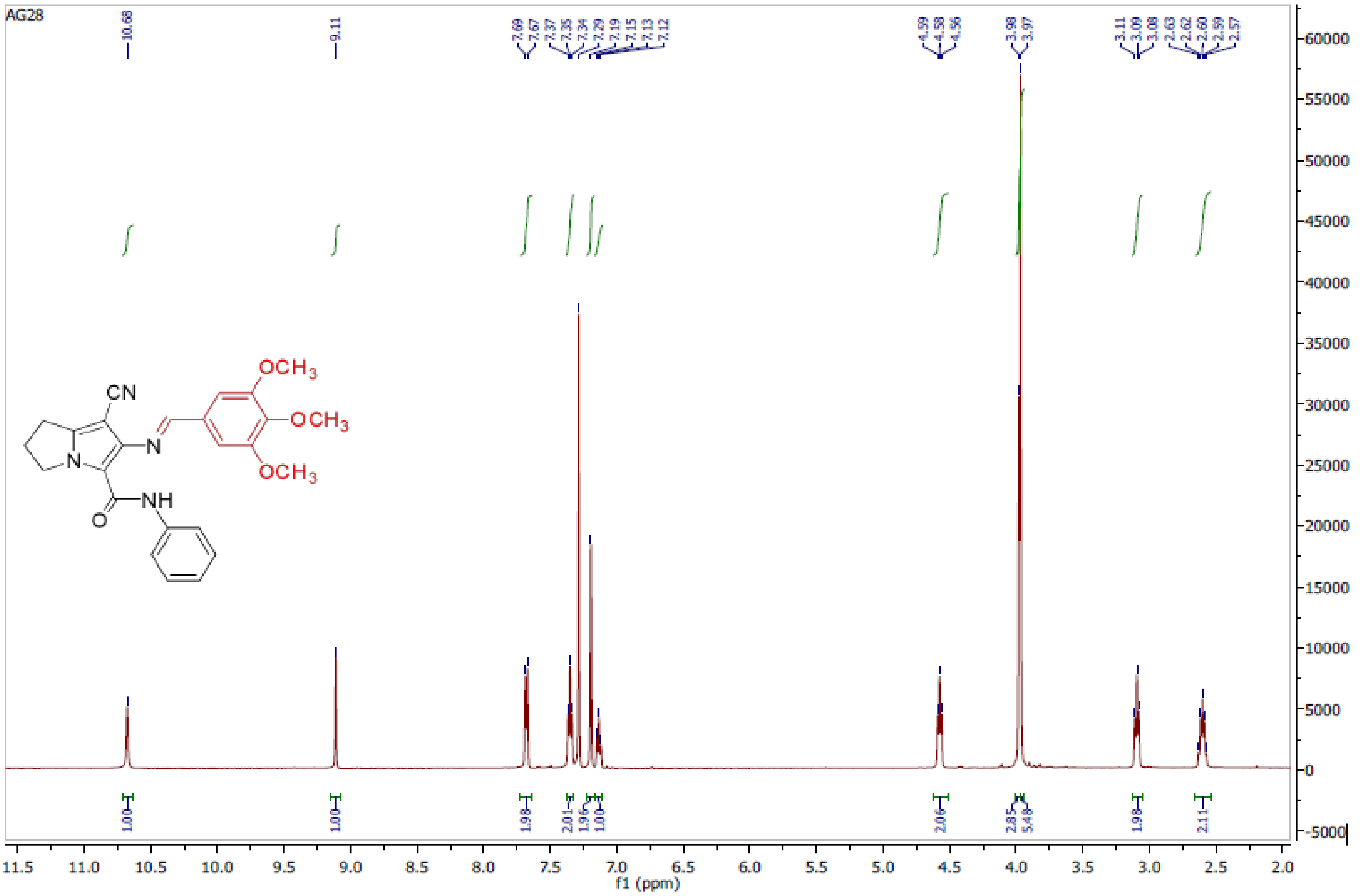

Fig. S14: ^{1}H-NMR ($CDCl_3$, 500 MHz, δ ppm) spectrum of compound **15a** (ZOOM on Aliphatic Hs)

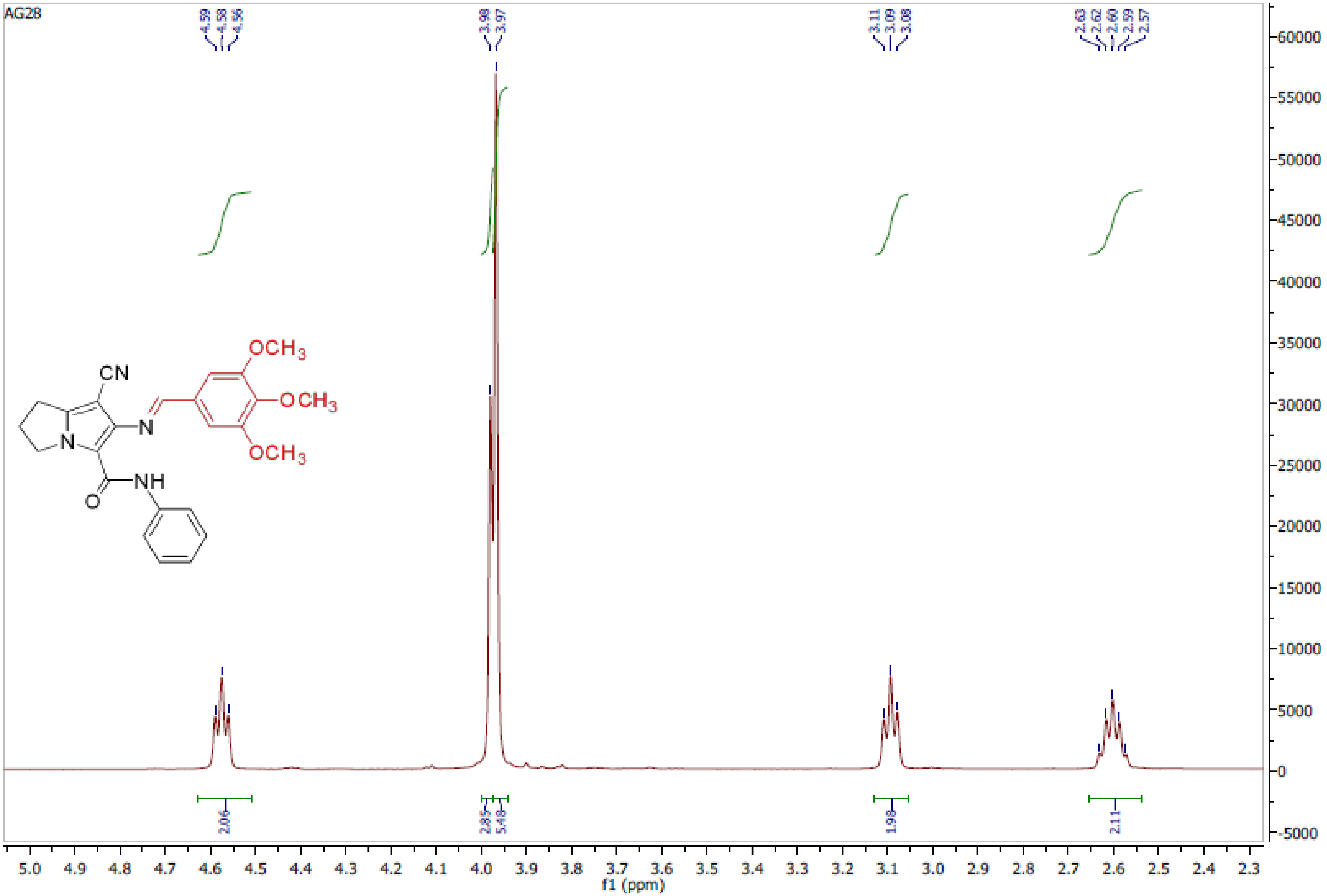

Fig. S15: ^{1}H-NMR ($CDCl_3$, 500 MHz, *δ* ppm) spectrum of compound **15a** (**ZOOM on trimethoxy Hs**)

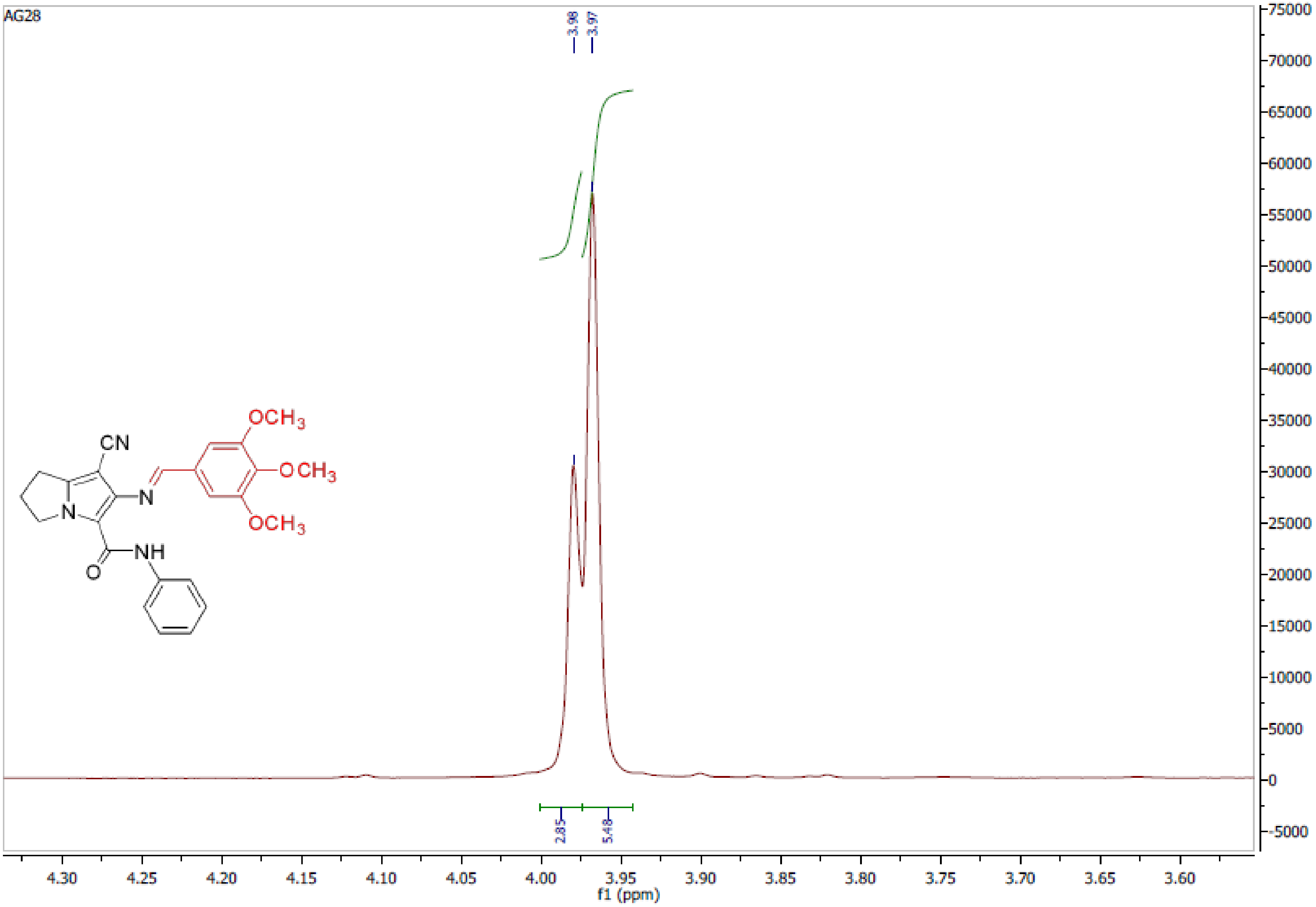

Fig. S16: ^{1}H-NMR ($CDCl_3$, 500 MHz, δ ppm) spectrum of compound **15a** (**ZOOM on NH, CH, Aromatic Hs**)

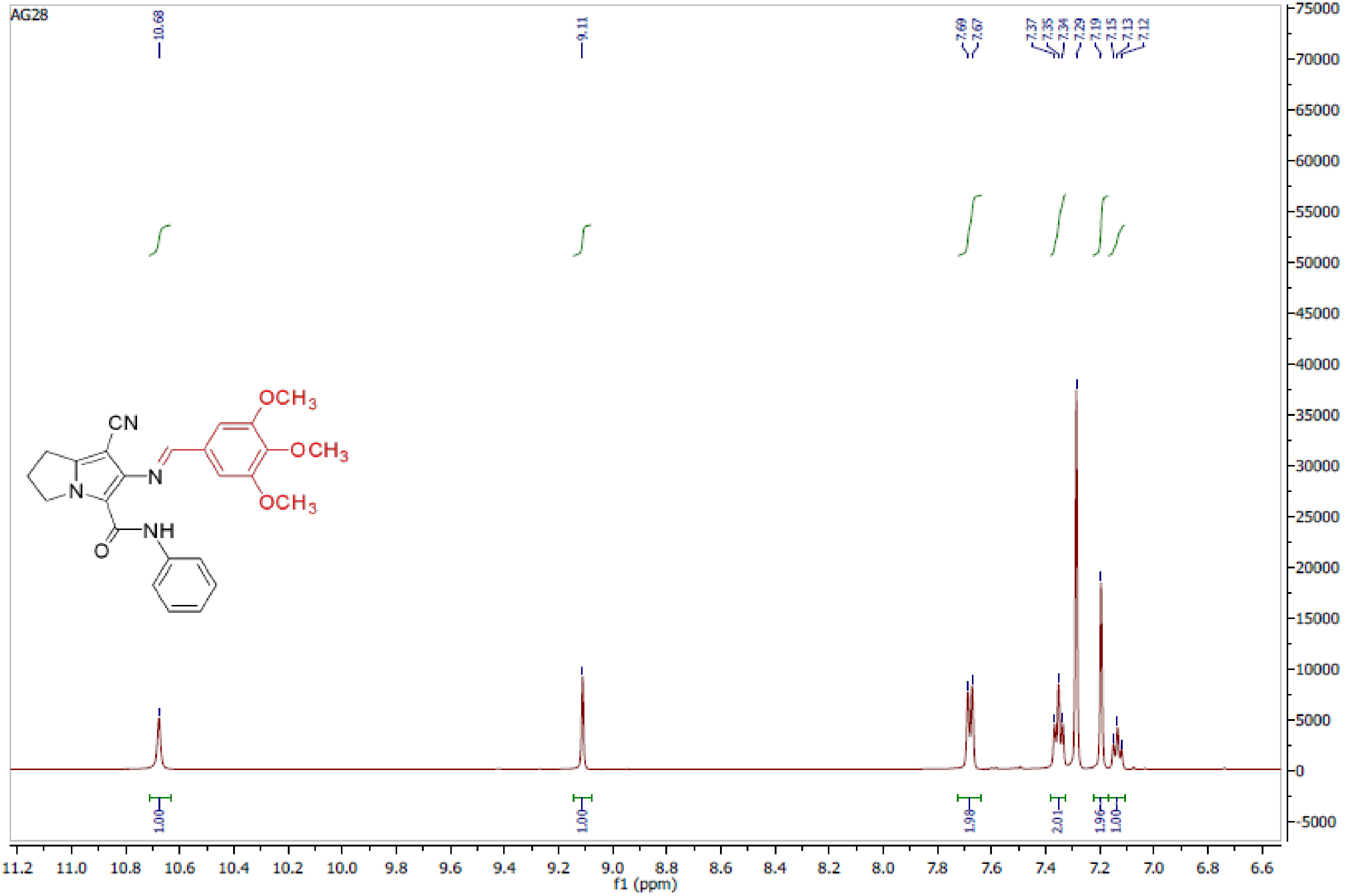

Fig. S17: ^{1}H-NMR ($CDCl_3$, 500 MHz, *δ* ppm) spectrum of compound **15a** (**ZOOM on Aromatic Hs**)

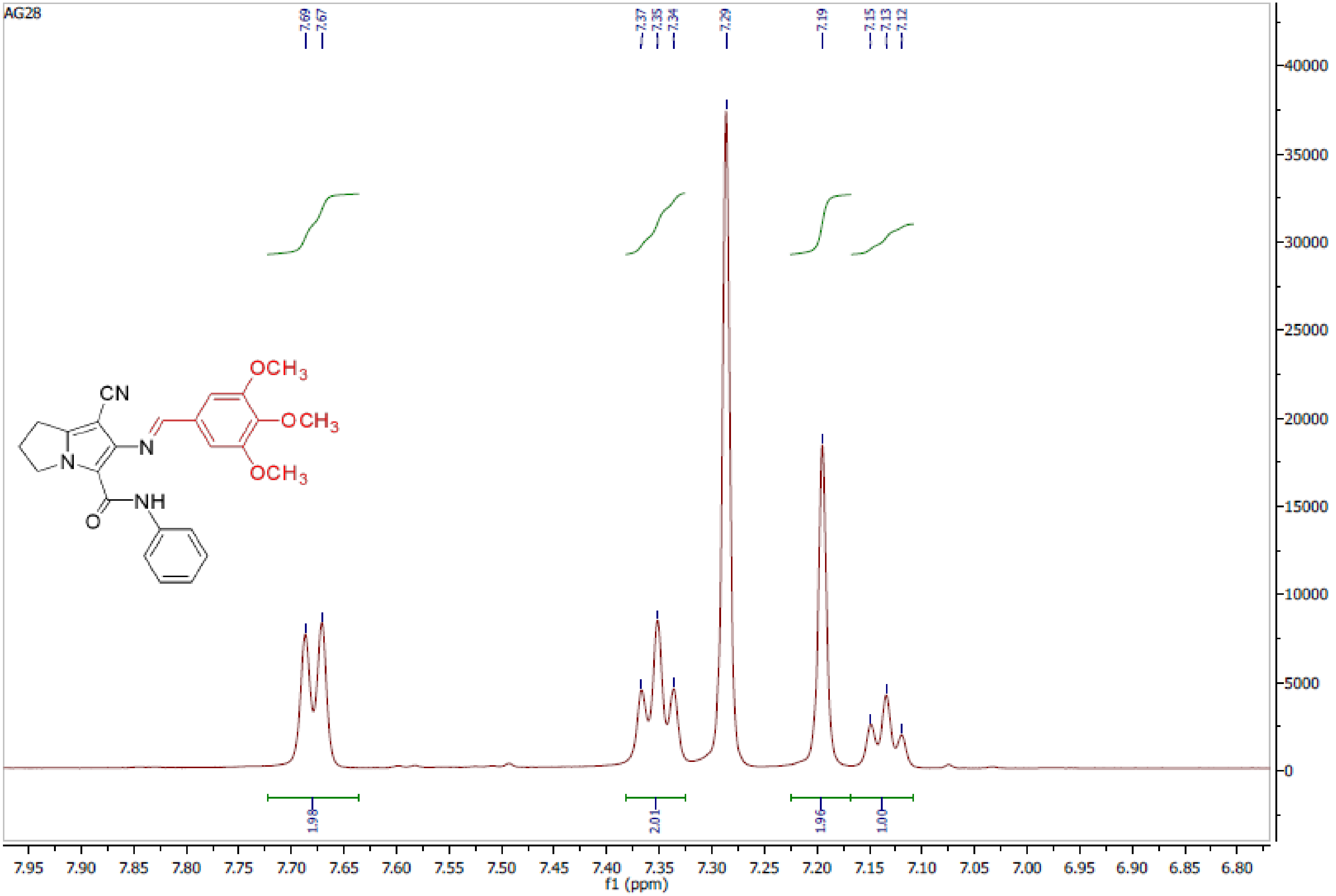

Fig. S18: ^{13}C-NMR ($CDCl_3$, 125 MHz, δ ppm) spectrum of compound **15a**

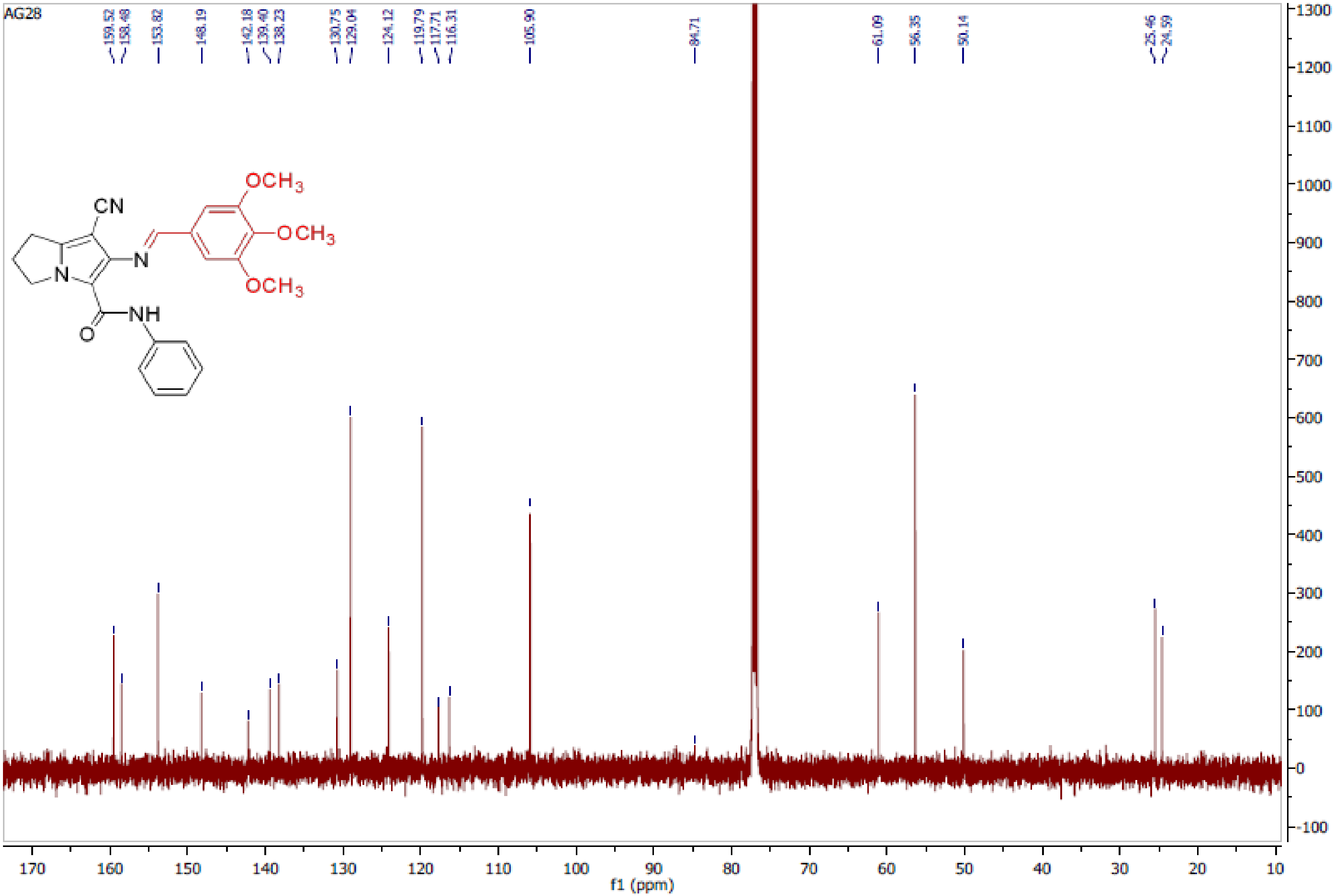

Fig. S19: ^{13}C-NMR ($CDCl_3$, 125 MHz, δ ppm) spectrum of compound **15a** (**ZOOM on Aliphatic Cs**)

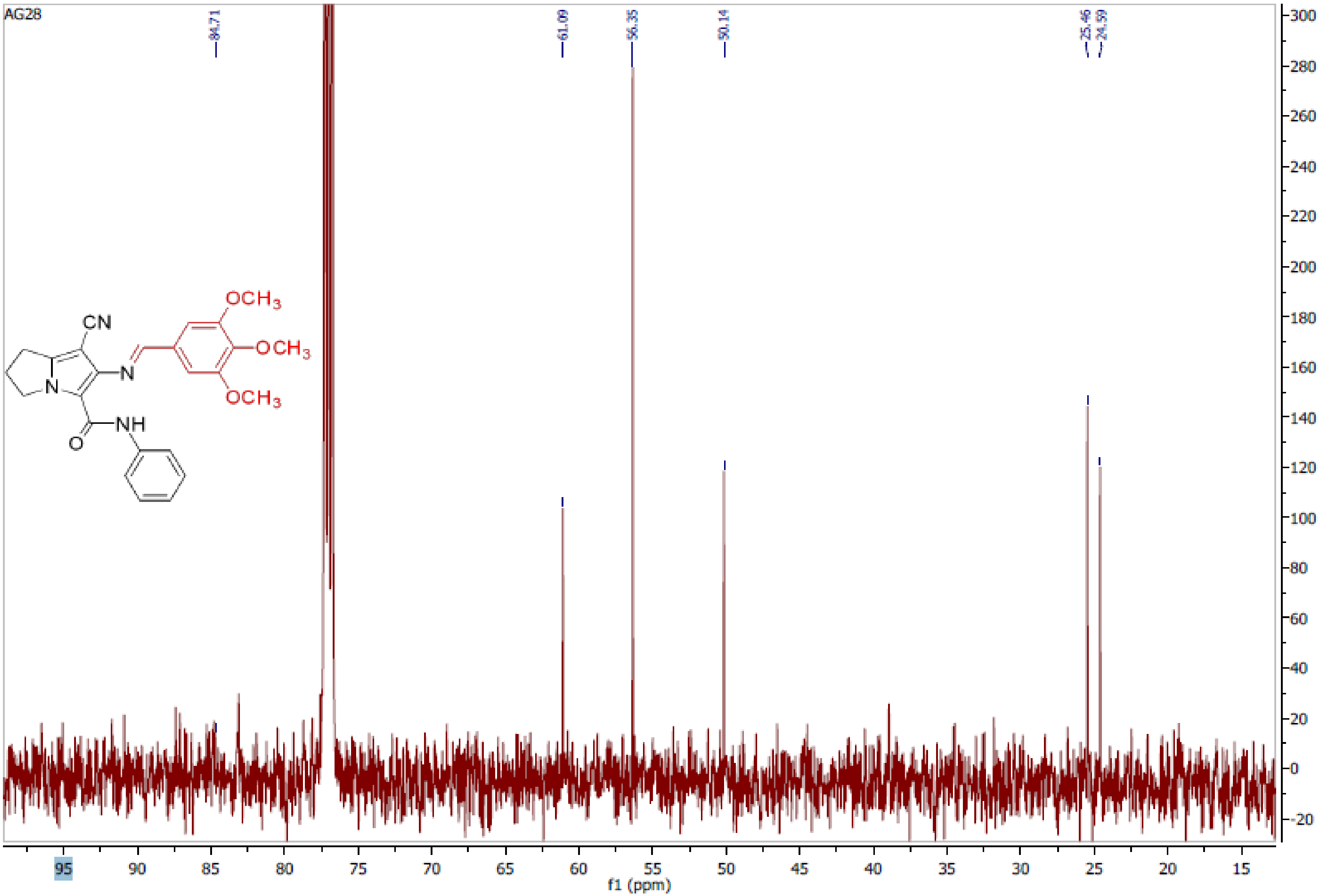

Fig. S20: ^{13}C-NMR ($CDCl_3$, 125 MHz, δ ppm) spectrum of compound **15a** (ZOOM on Aromatic Cs)

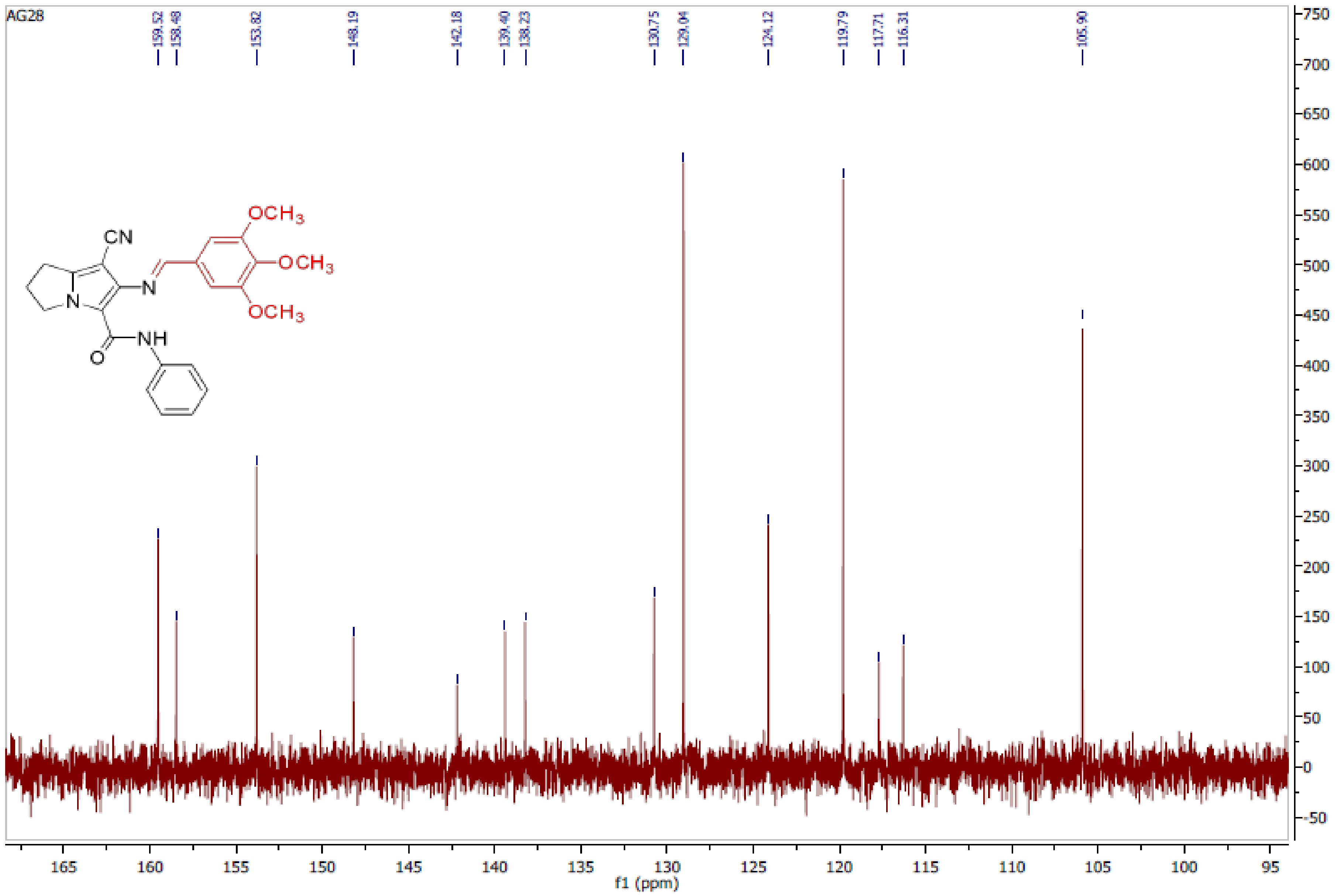

Fig. S21: DEPT C[135] spectrum of compound 15a

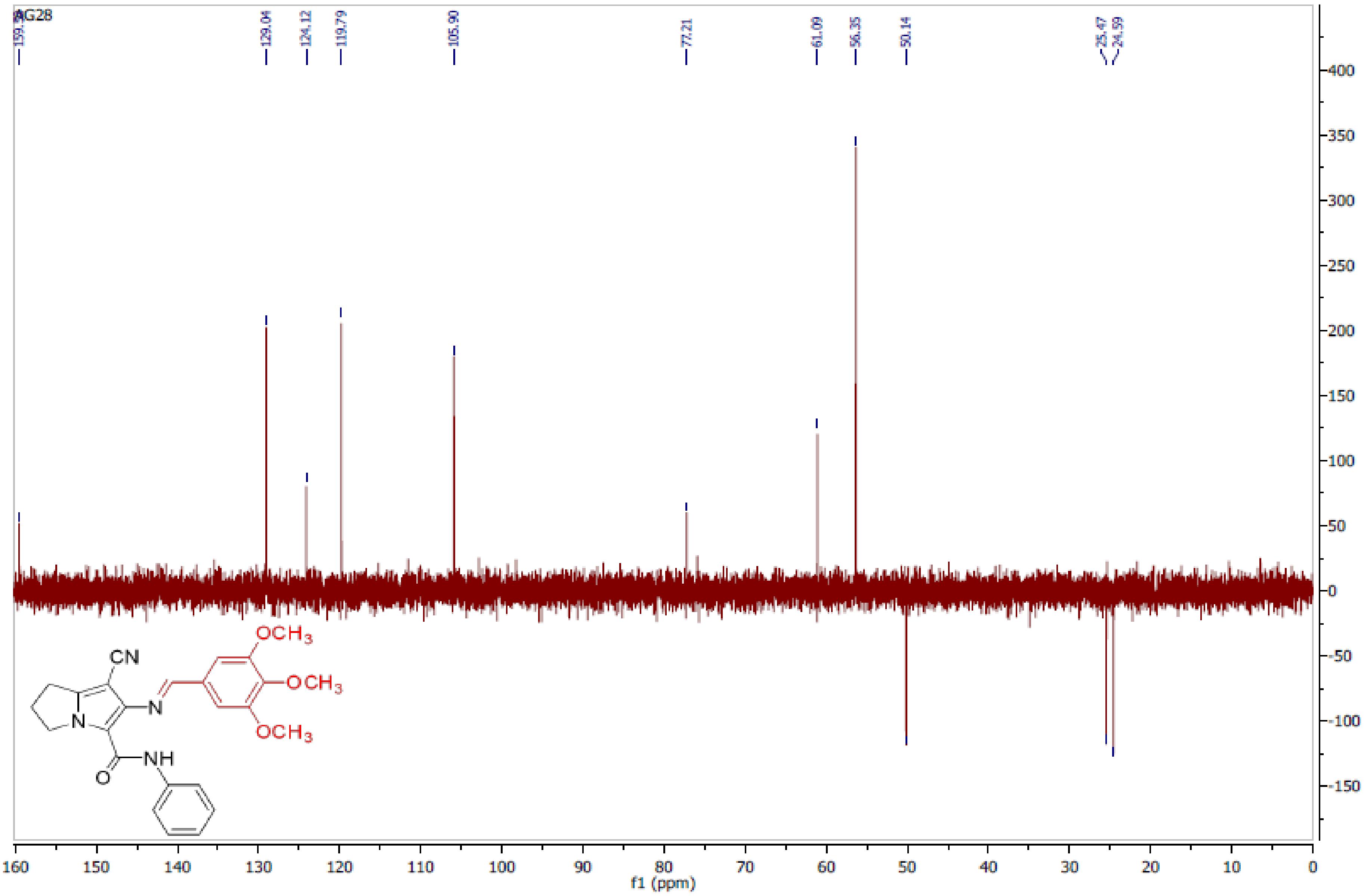

Fig. S22: ^{1}H-NMR ($CDCl_3$, 500 MHz, δ ppm) spectrum of compound **15b**

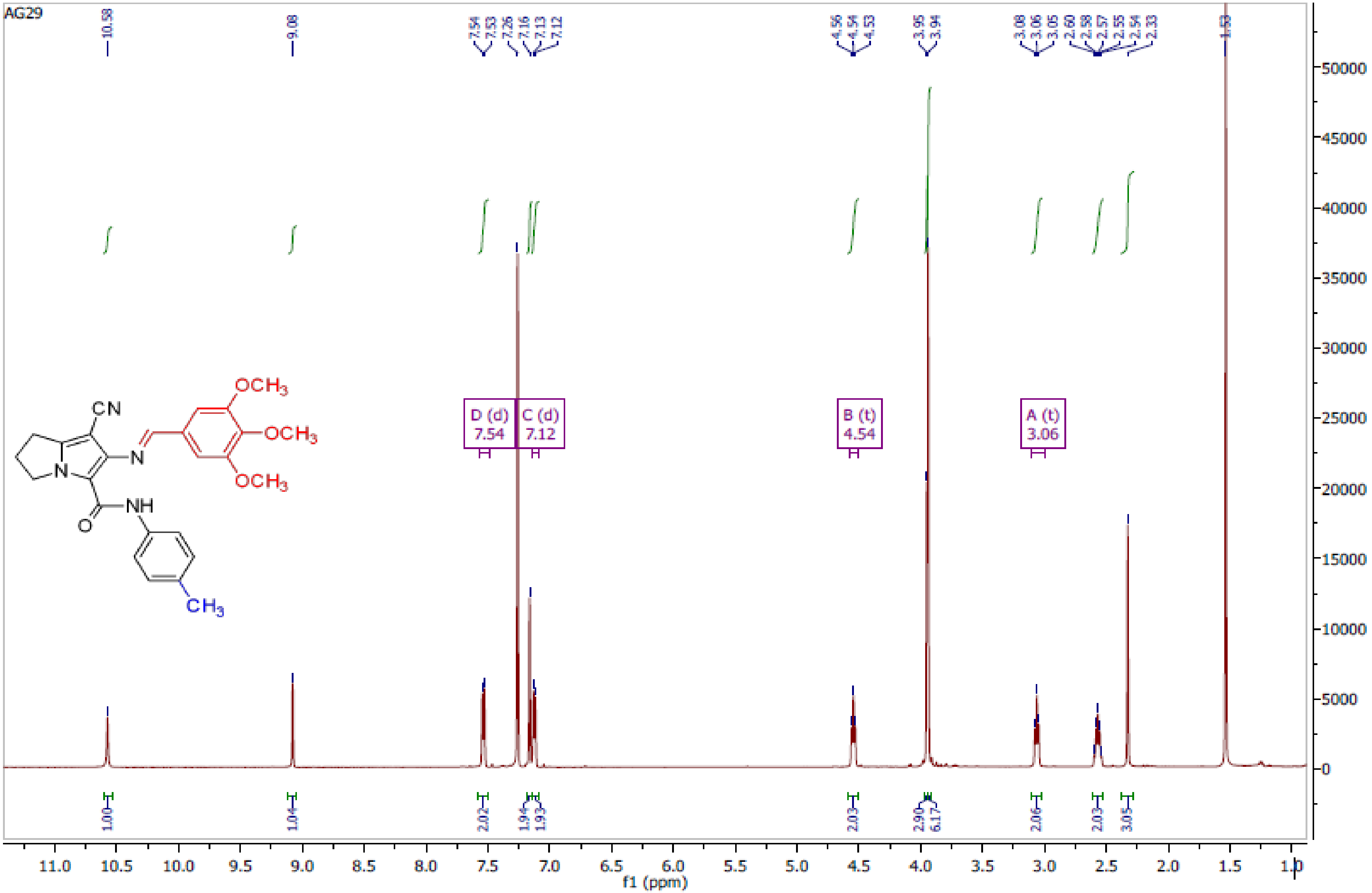

Fig. S23: ^{1}H-NMR ($CDCl_3$, 500 MHz, δ ppm) spectrum of compound **15b** (**ZOOM on Aliphatic Hs**)

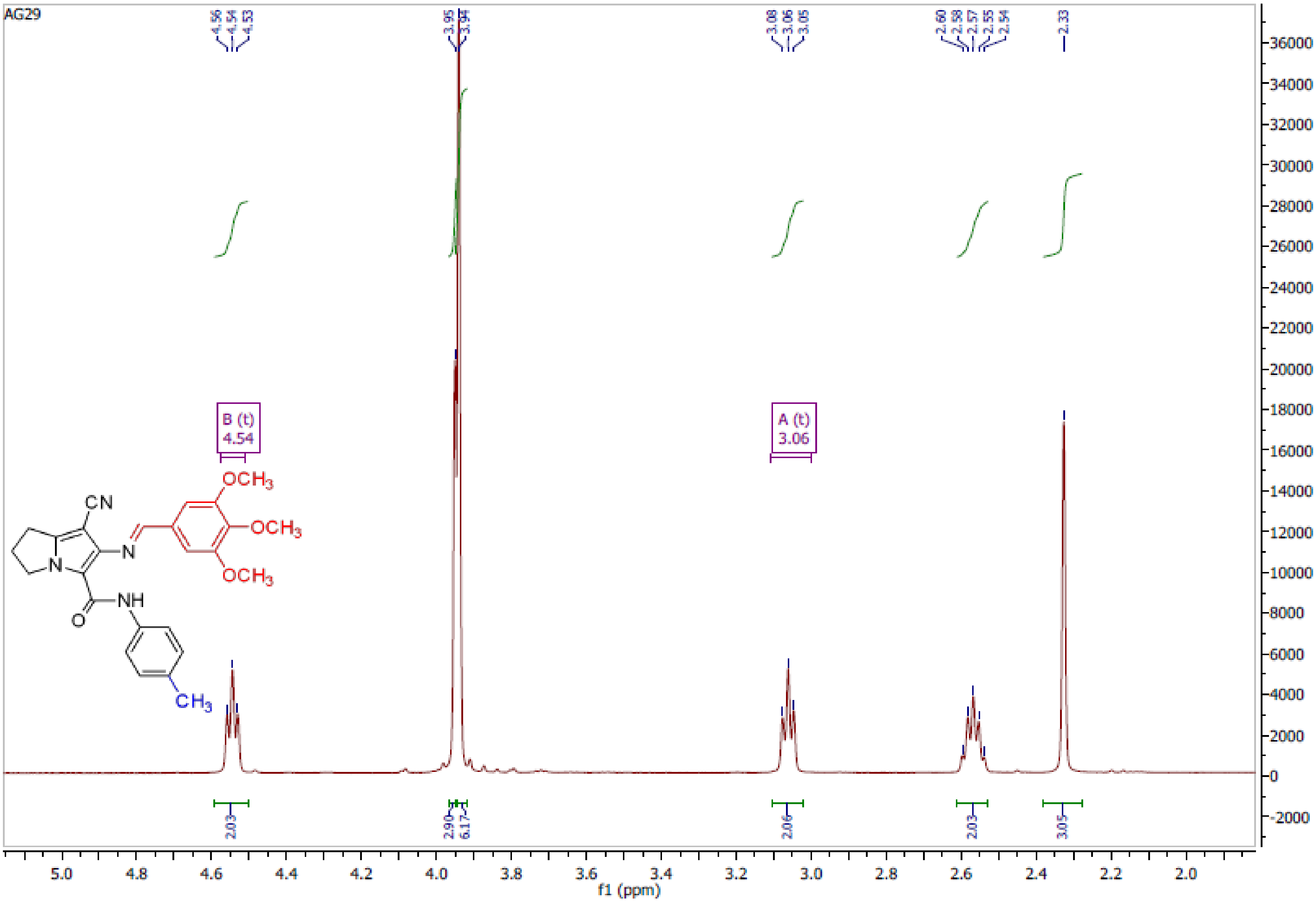

Fig. S24: ^{1}H-NMR ($CDCl_3$, 500 MHz, *δ* ppm) spectrum of compound **15b** (**ZOOM on NH, CH, Aromatic Hs**)

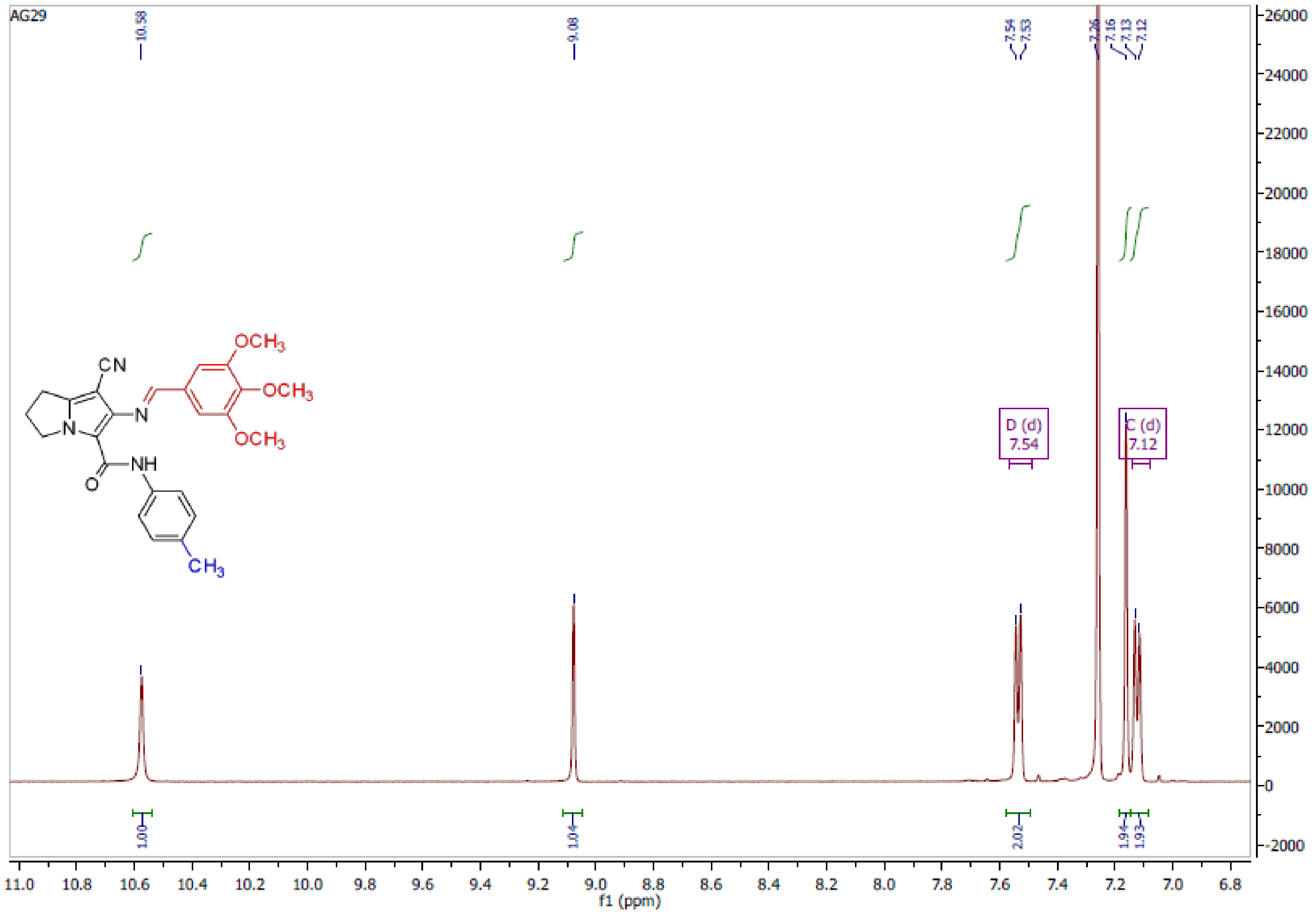

Fig. S25: ^{1}H-NMR ($CDCl_3$, 500 MHz, *δ* ppm) spectrum of compound **15b** (**ZOOM on Aromatic Hs**)

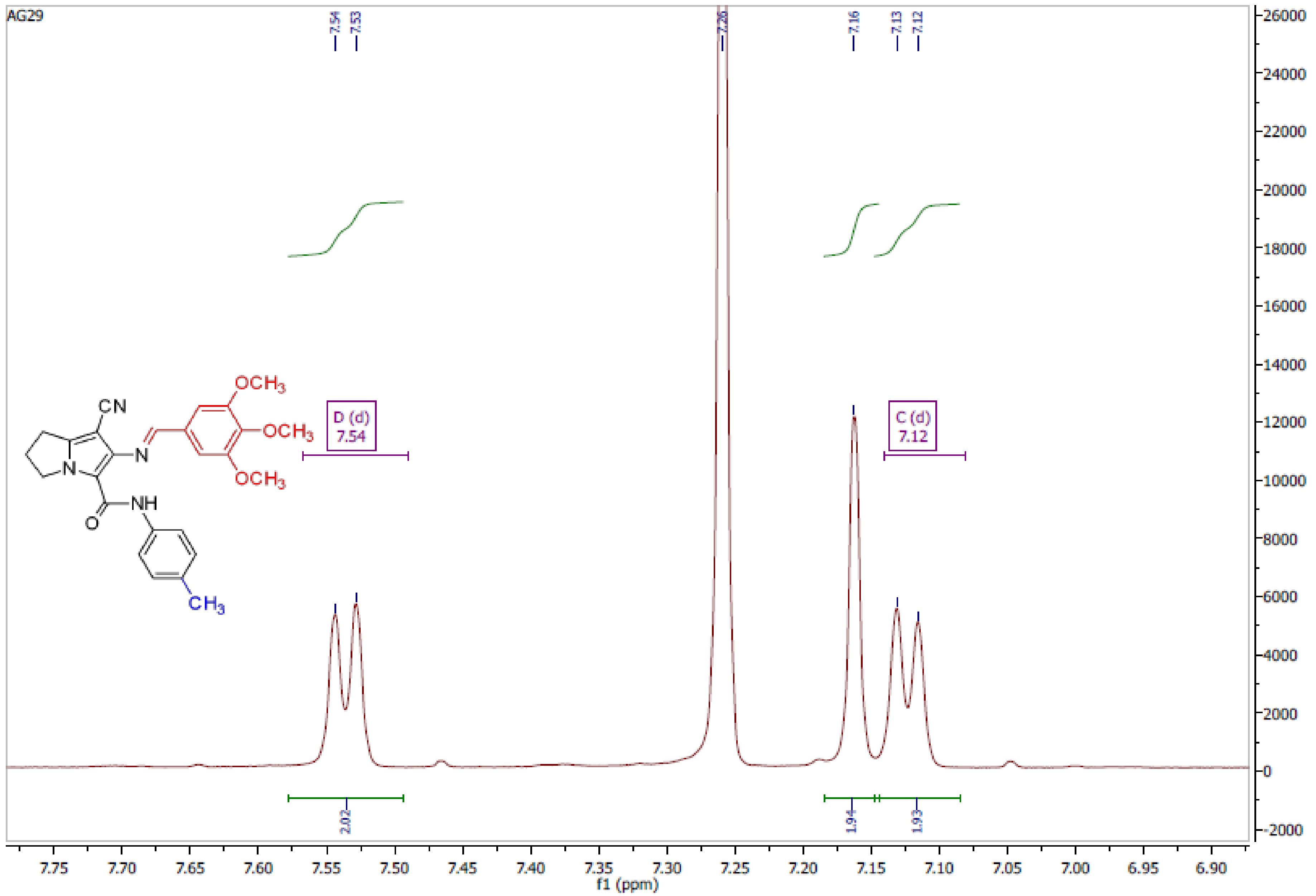

Fig. S26: ^{13}C-NMR ($CDCl_3$, 125 MHz, δ ppm) spectrum of compound **15b**

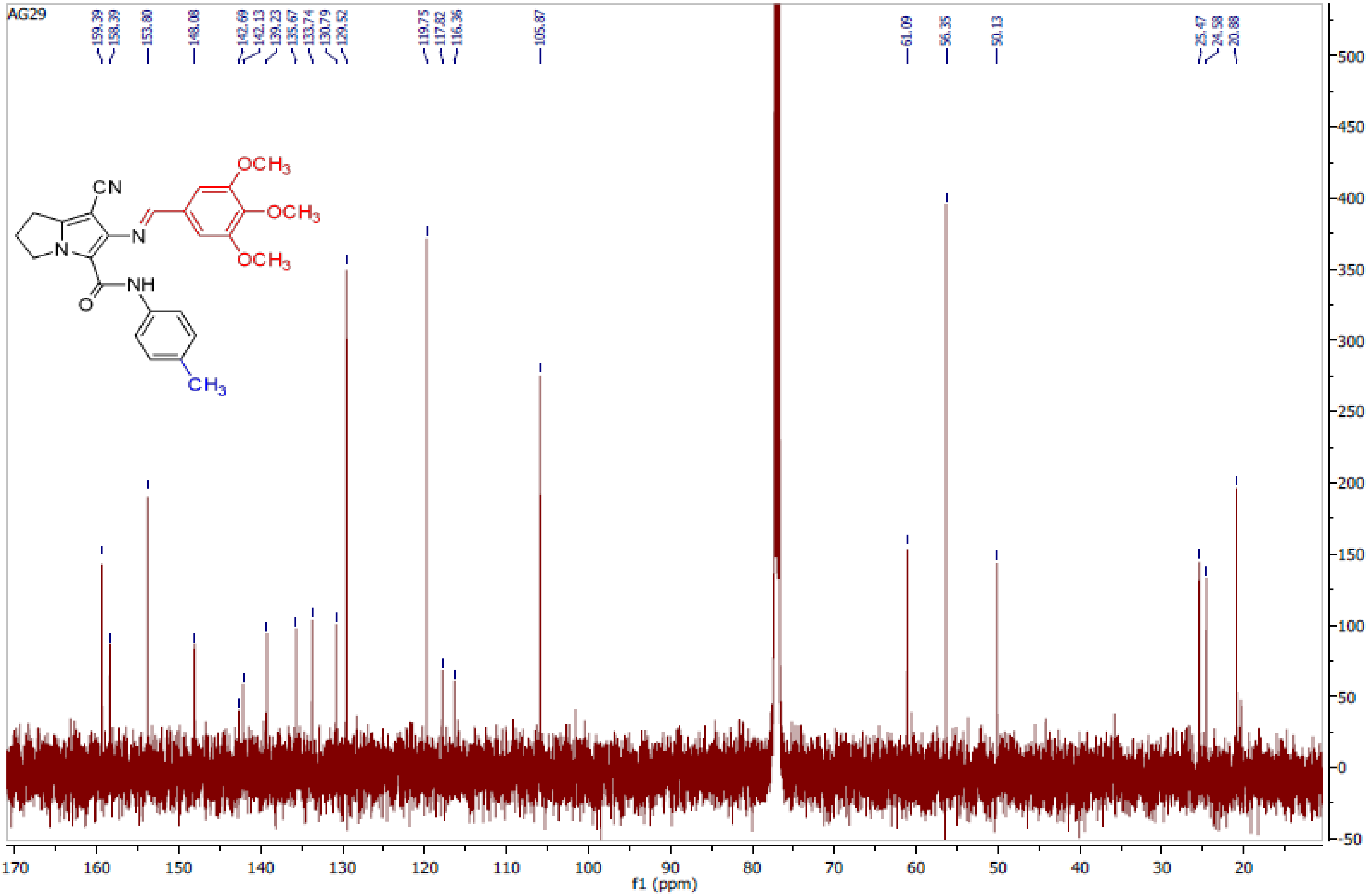

Fig. S27: ^{13}C-NMR ($CDCl_3$, 125 MHz, δ ppm) spectrum of compound **15b** (**ZOOM on Aliphatic Cs**)

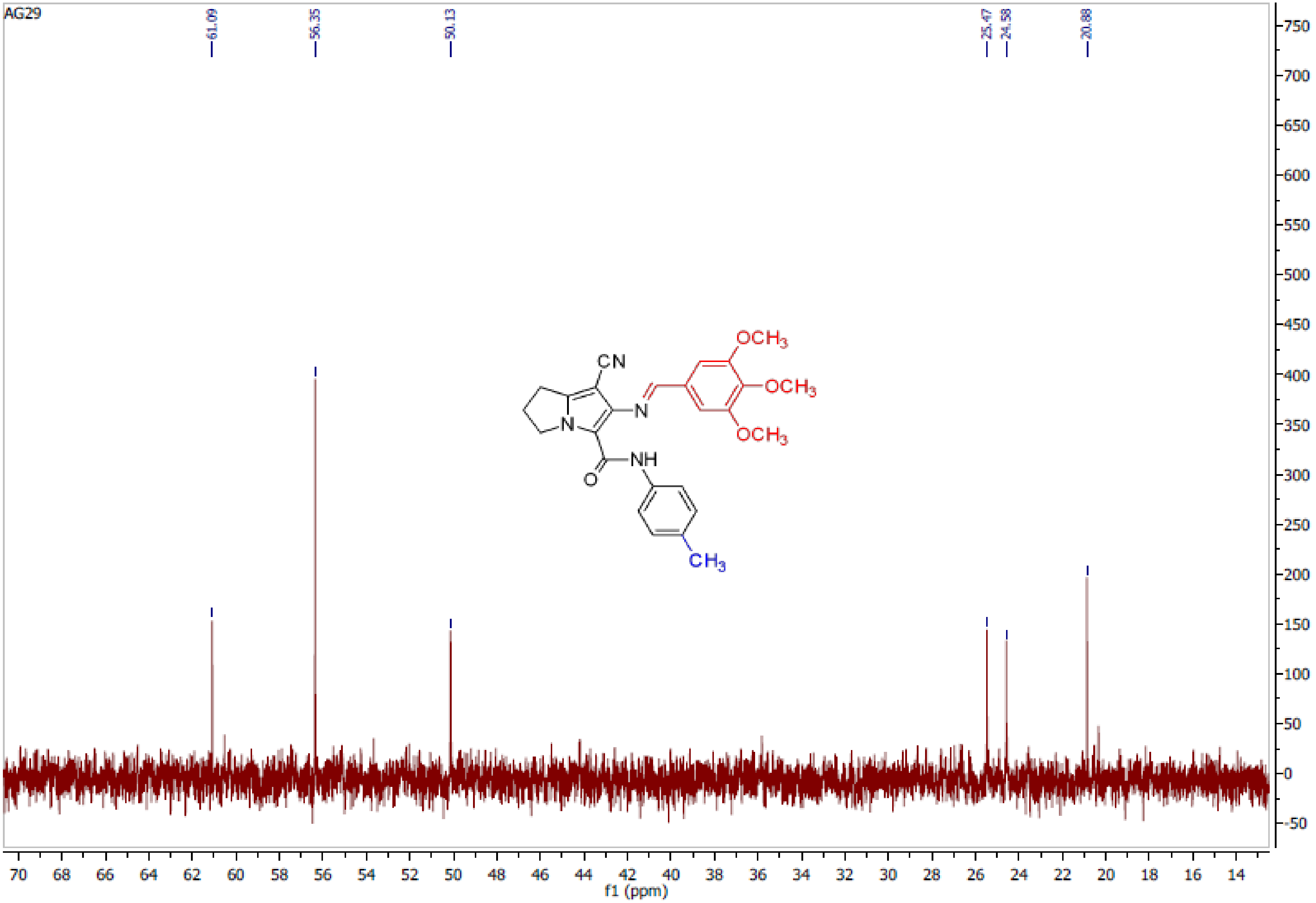

Fig. S28: ^{13}C-NMR ($CDCl_3$, 125 MHz, δ ppm) spectrum of compound **15b** (**ZOOM on aromatic Cs**)

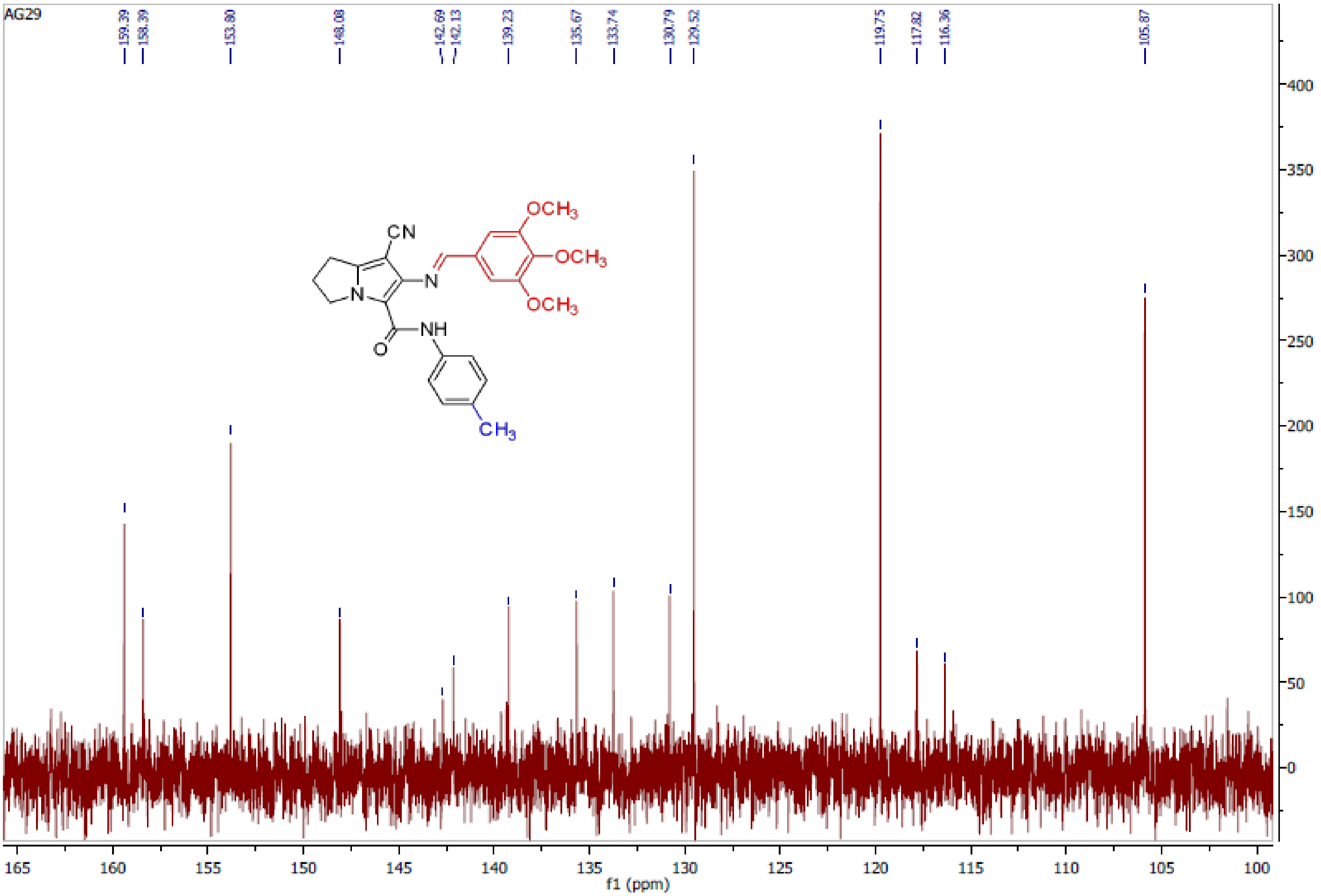

Fig. S29: **DEPT C^{135} spectrum of compound 15b**

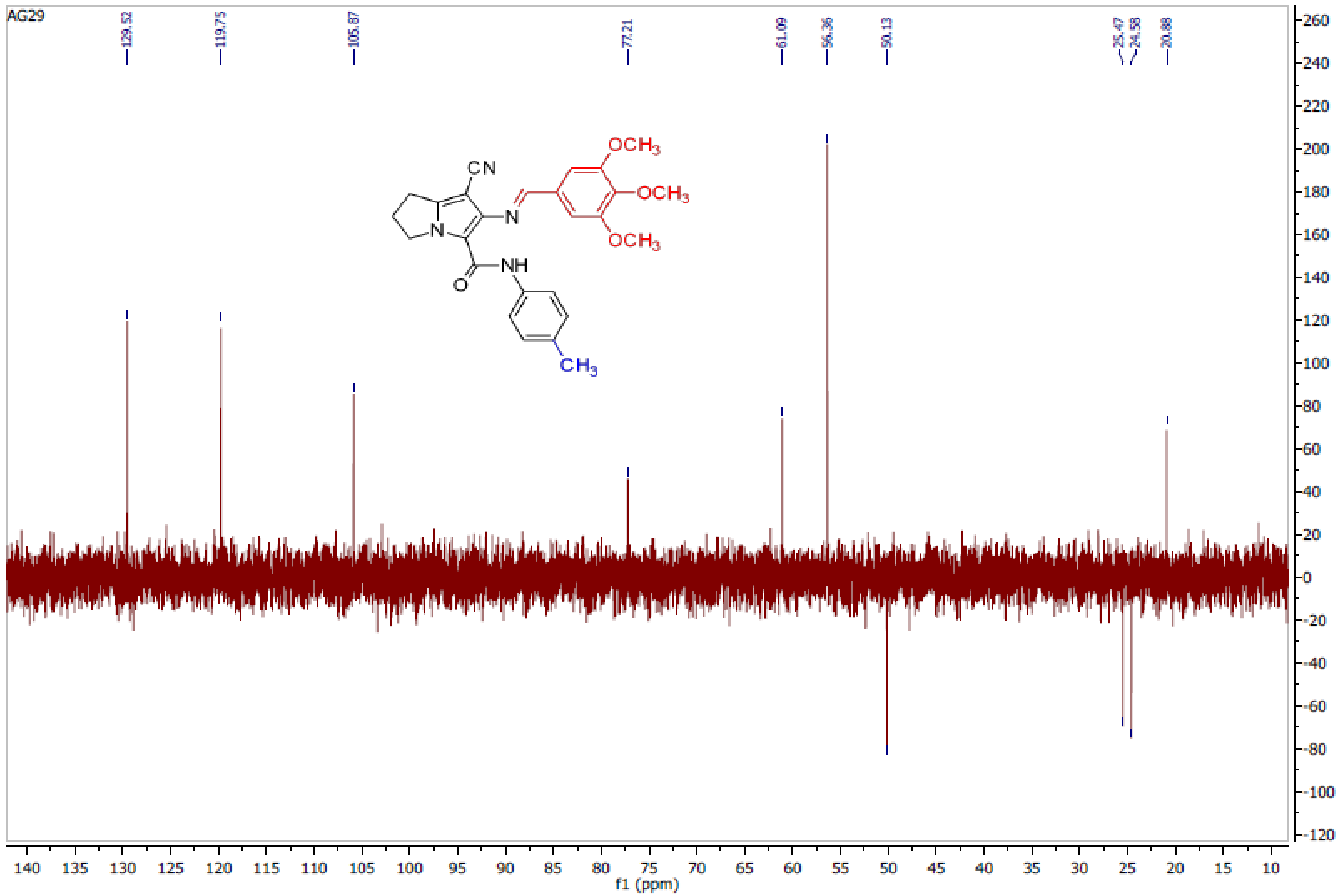

Fig. S30. ^{1}H-NMR ($CDCl_3$, 500 MHz, δ ppm) spectrum of compound **15c**

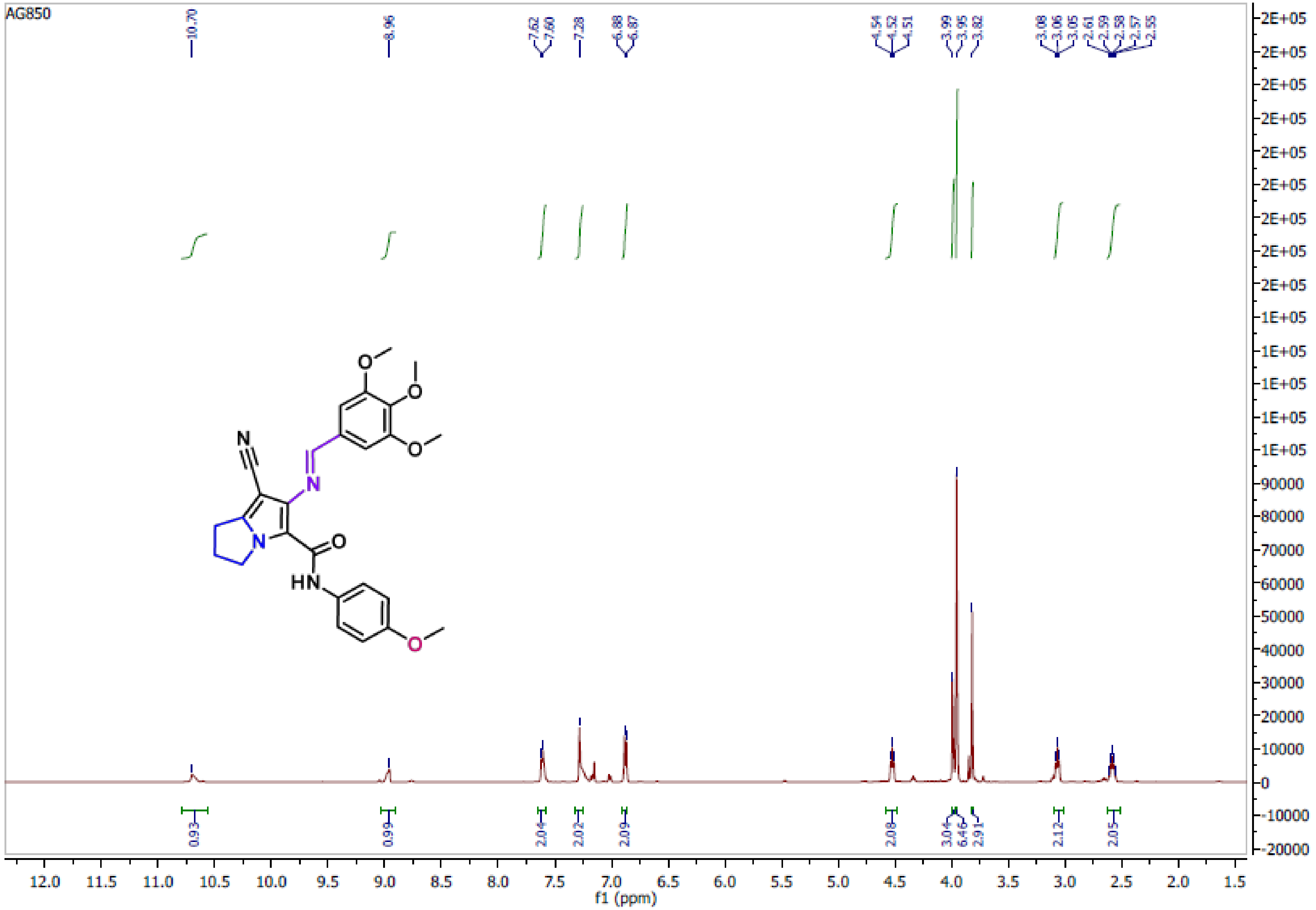

Fig. S31. ^{1}H-NMR ($CDCl_3$, 500 MHz, δ ppm) spectrum of compound **15c** (**zoom on pyrrolizine Hs, two triplet**)

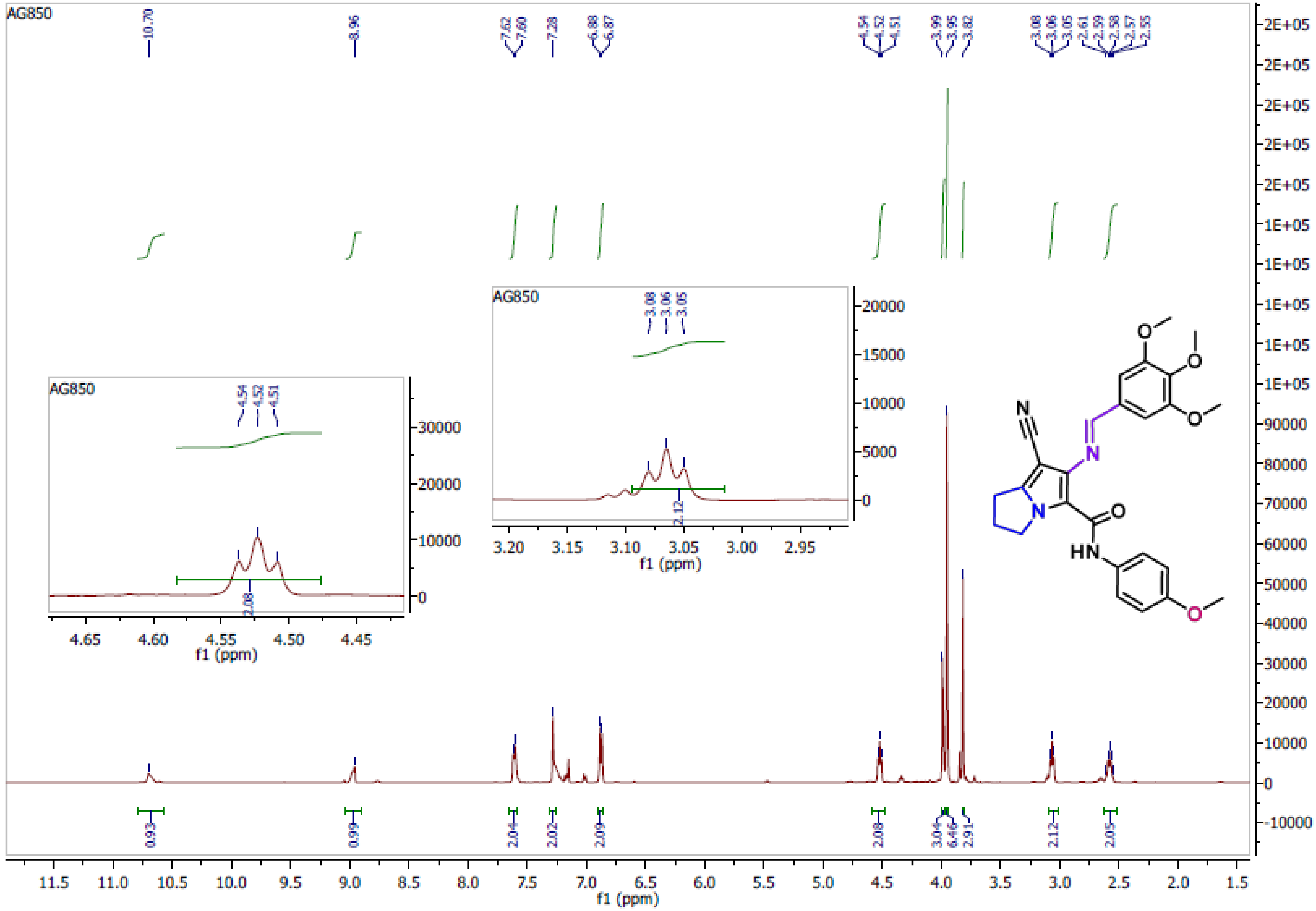

Fig. S32. ^{1}H-NMR ($CDCl_3$, 500 MHz, δ ppm) spectrum of compound **15c** (**zoom, aliphatic Hs**)

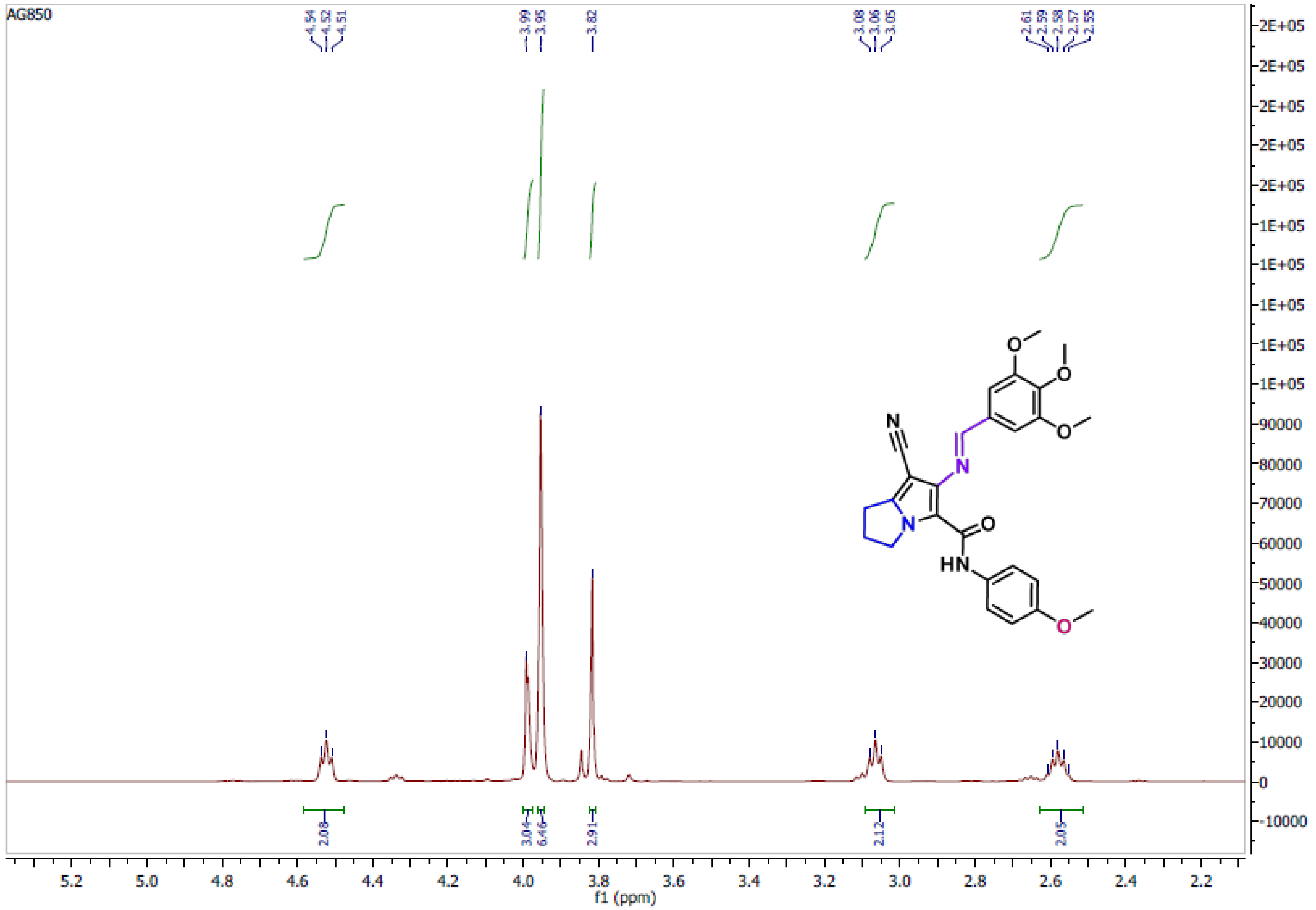

Fig. S33. ^{1}H-NMR ($CDCl_3$, 500 MHz, δ ppm) spectrum of compound **15c** (**zoom on aromatic Hs**)

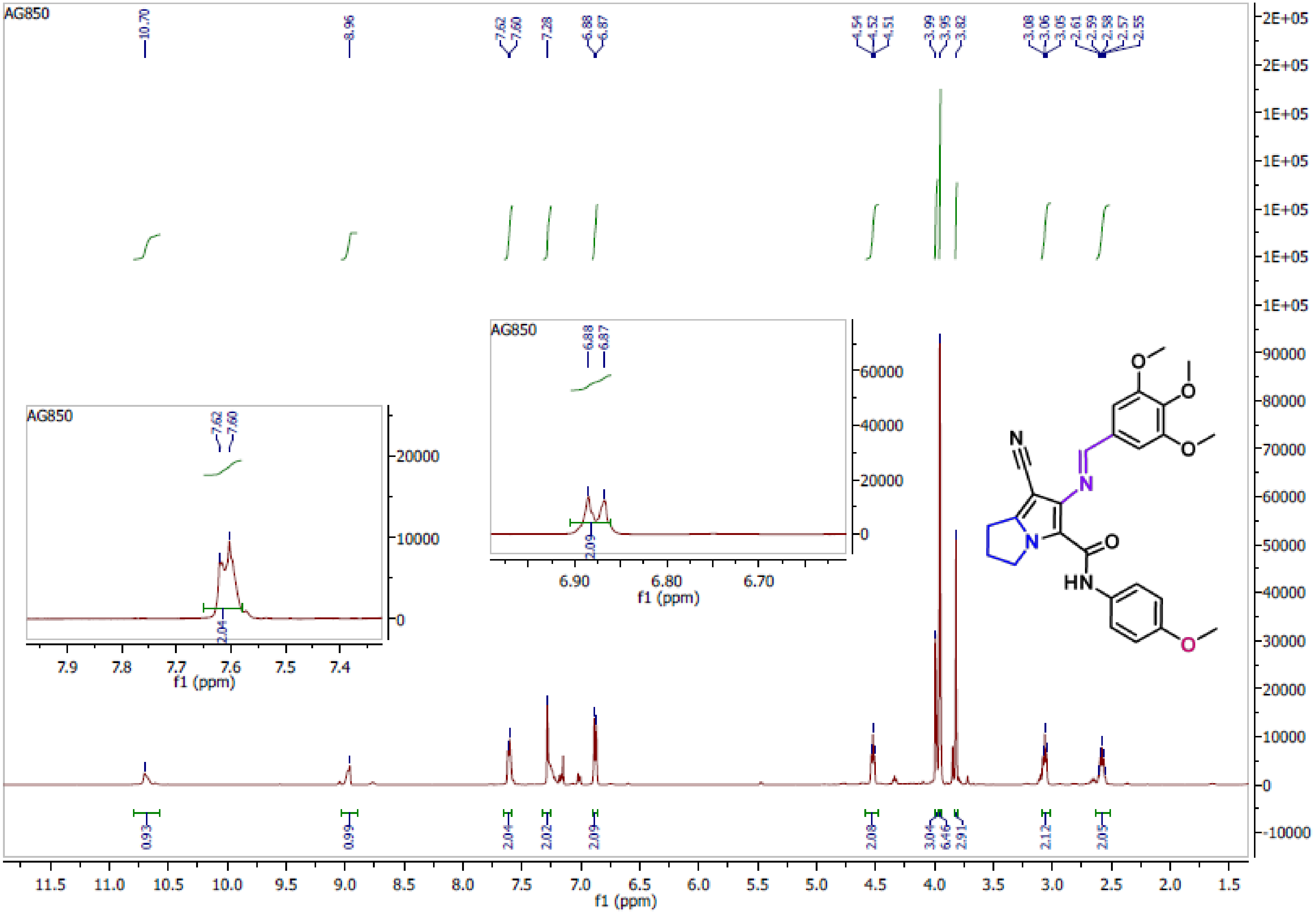

Fig. S34. ^{13}C-NMR ($CDCl_3$, 125 MHz, δ ppm) spectrum of compound **15c** (zoom, aromatic Cs)

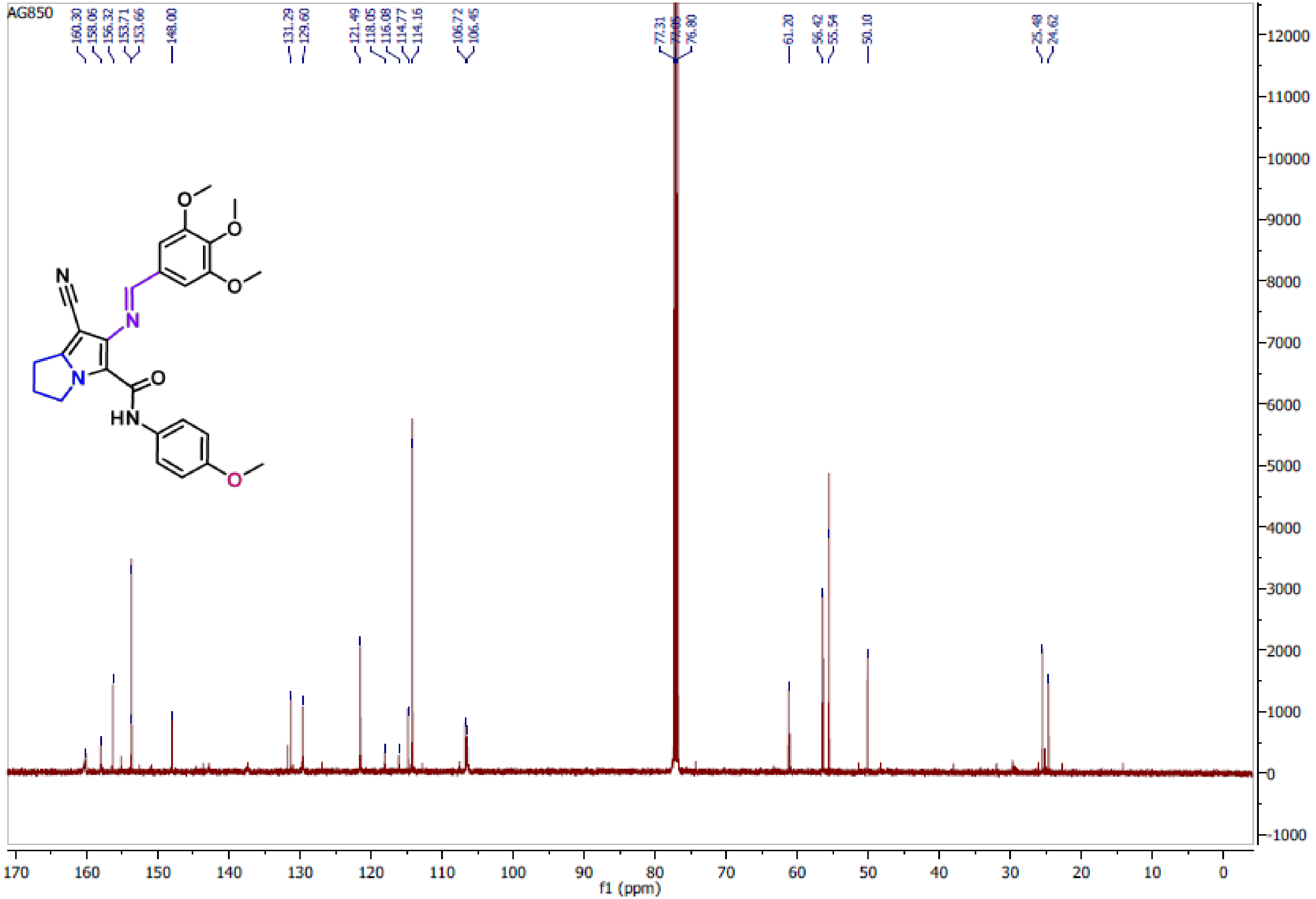

Fig. S35. **DEPT C[135] ($CDCl_3$, 125 MHz, δ ppm) of compound 15c**

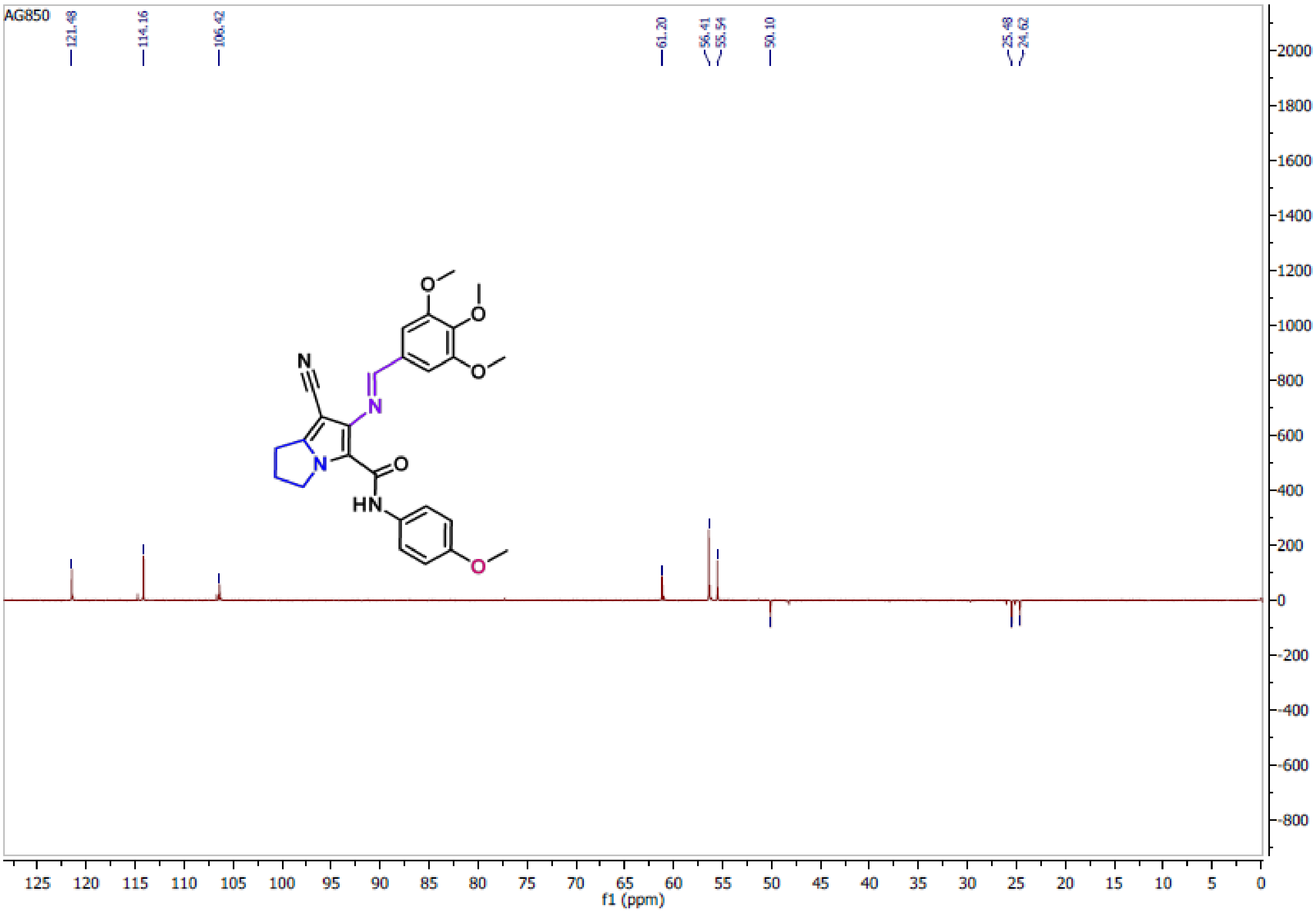

Fig. S36. ^{1}H-NMR ($CDCl_3$, 500 MHz, *δ* ppm) spectrum of compound **15d**

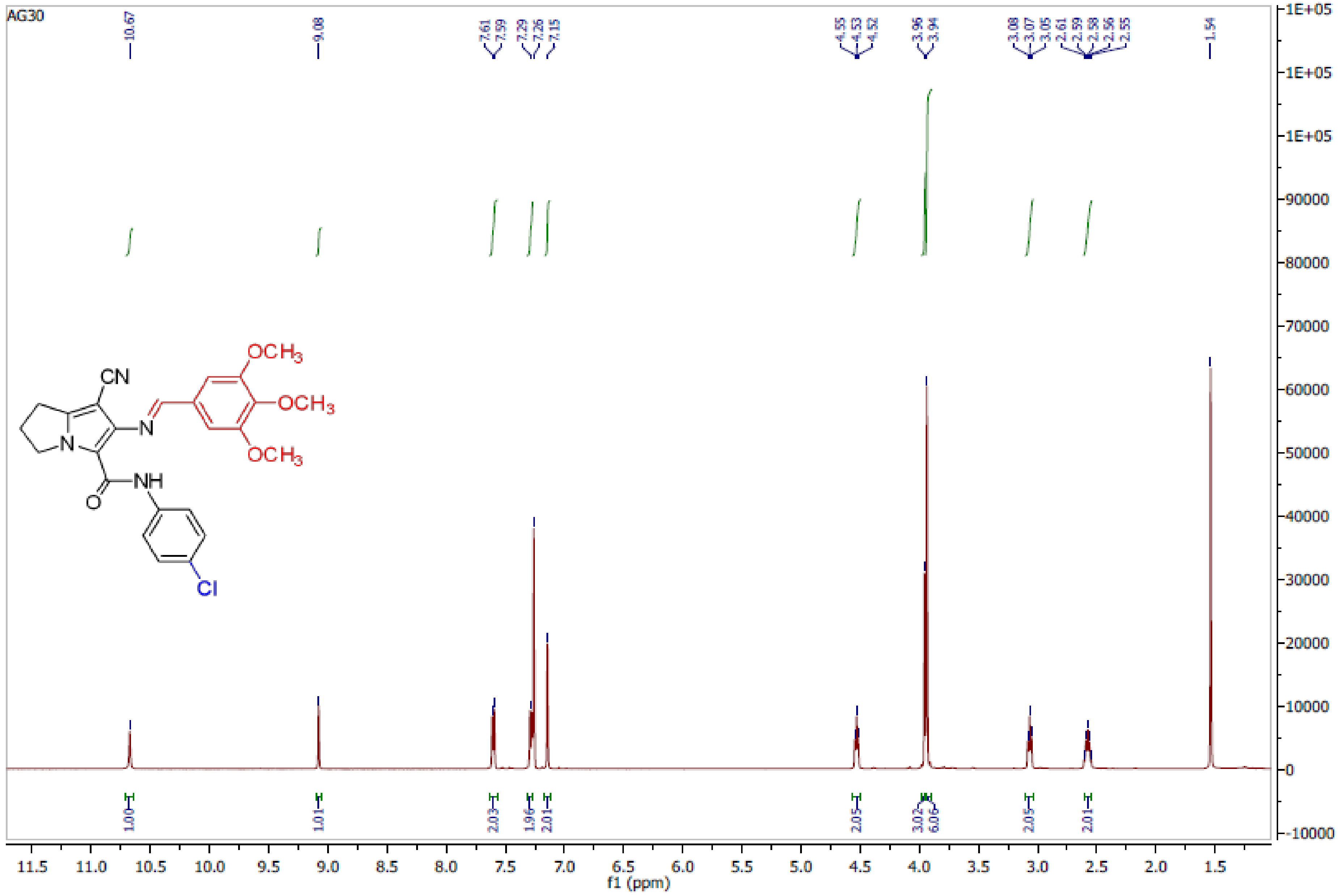

Fig. S37. ^{1}H-NMR ($CDCl_3$, 500 MHz, δ ppm) spectrum of compound **15d** (**ZOOM on aliphatic Hs**)

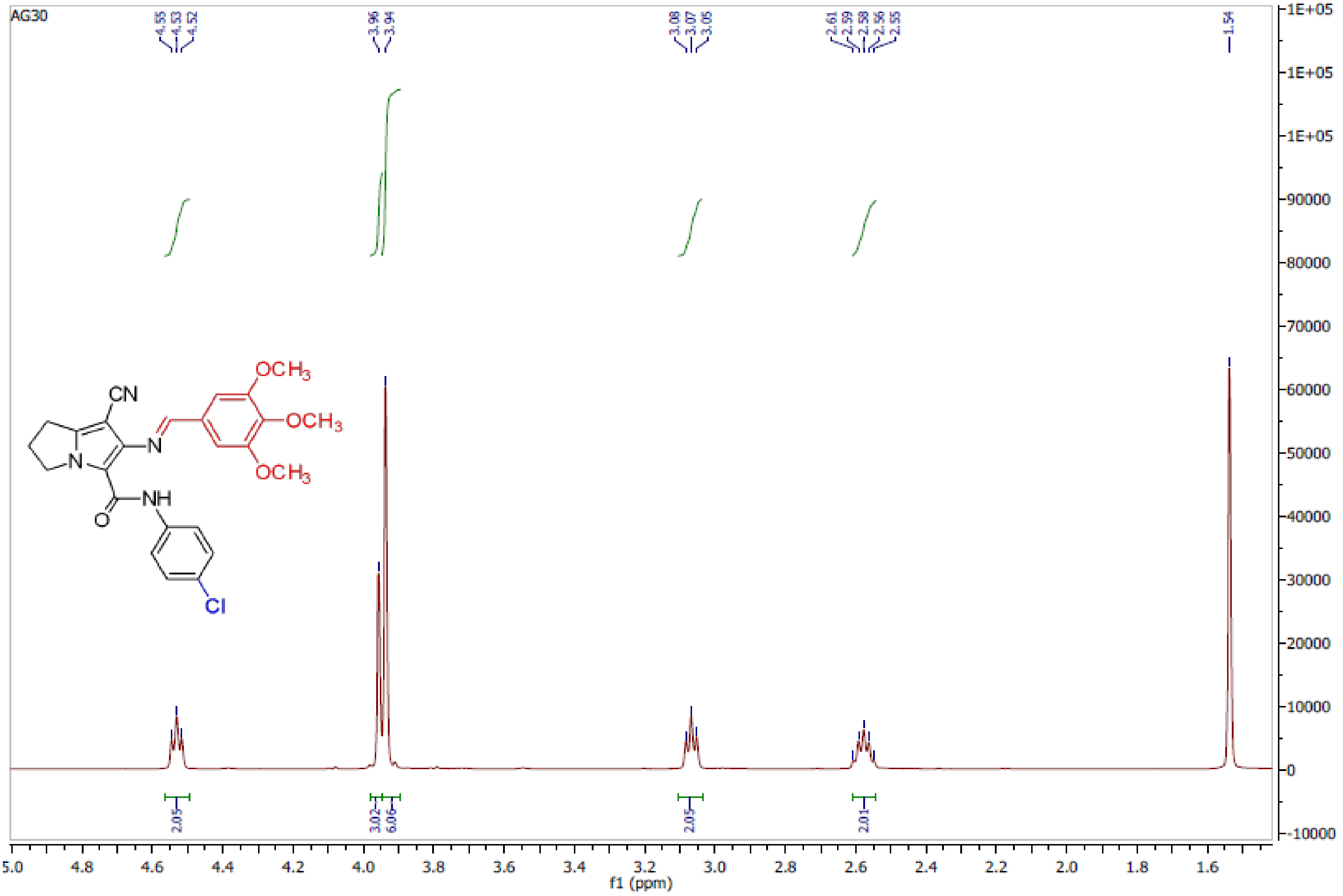

Fig. S38. ^{1}H-NMR ($CDCl_3$, 500 MHz, δ ppm) spectrum of compound **15d** (**ZOOM on NH, CH, aromatic Hs**)

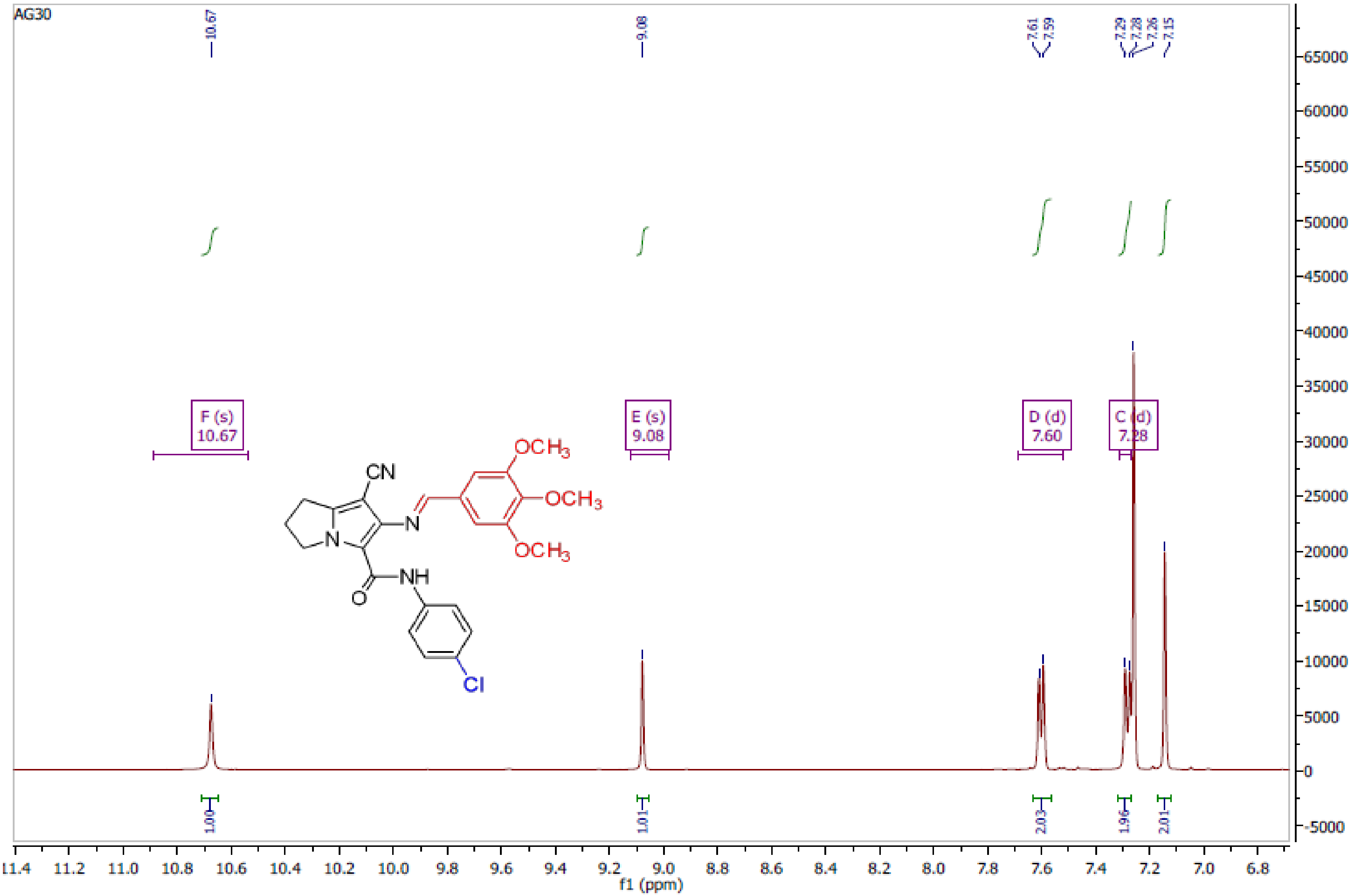

Fig. S39. ^{1}H-NMR ($CDCl_3$, 500 MHz, δ ppm) spectrum of compound **15d** (**ZOOM on aromatic Hs**)

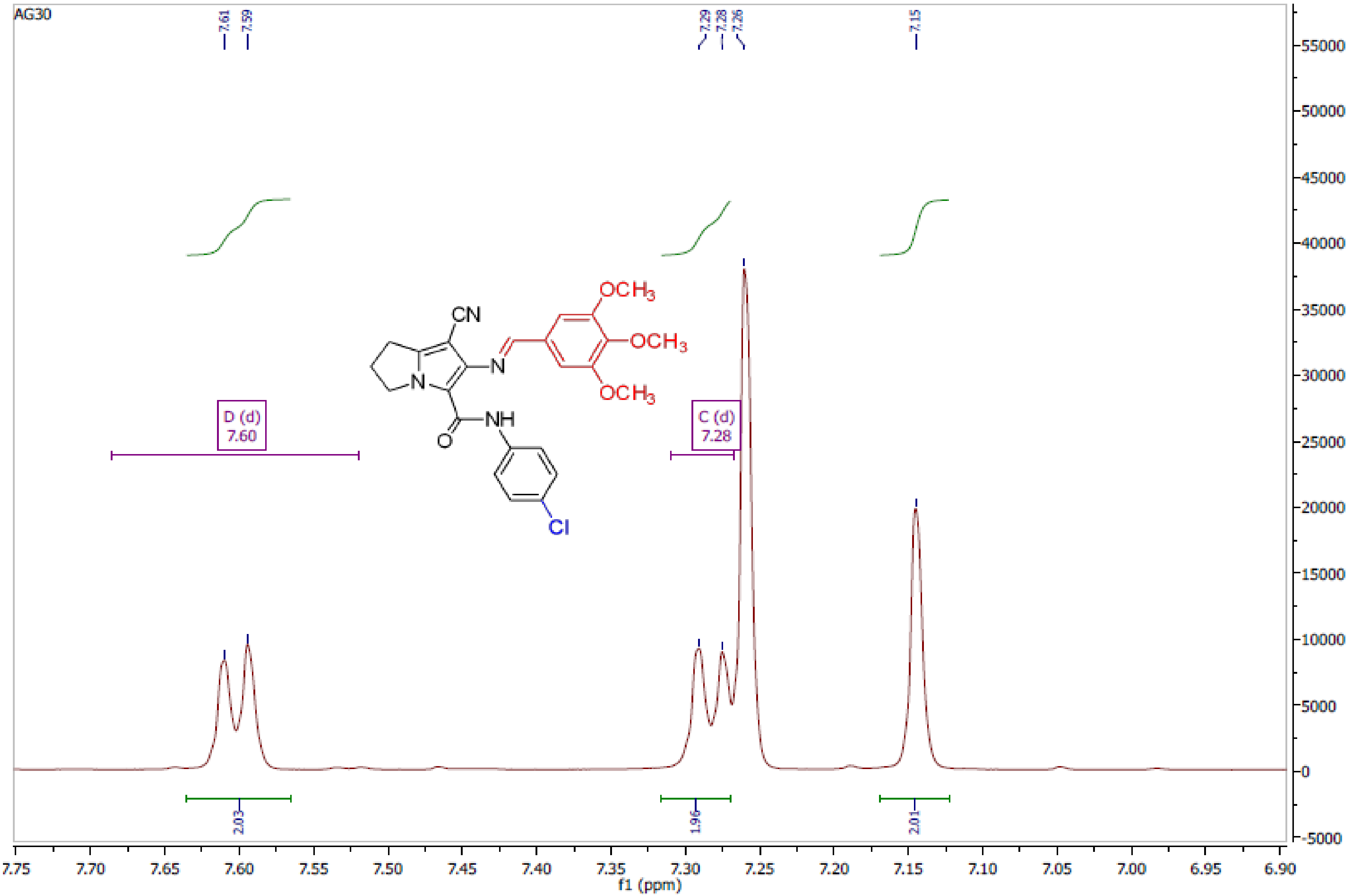

Fig. S40. ^{13}C-NMR ($CDCl_3$, 125 MHz, δ ppm) spectrum of compound **15d**

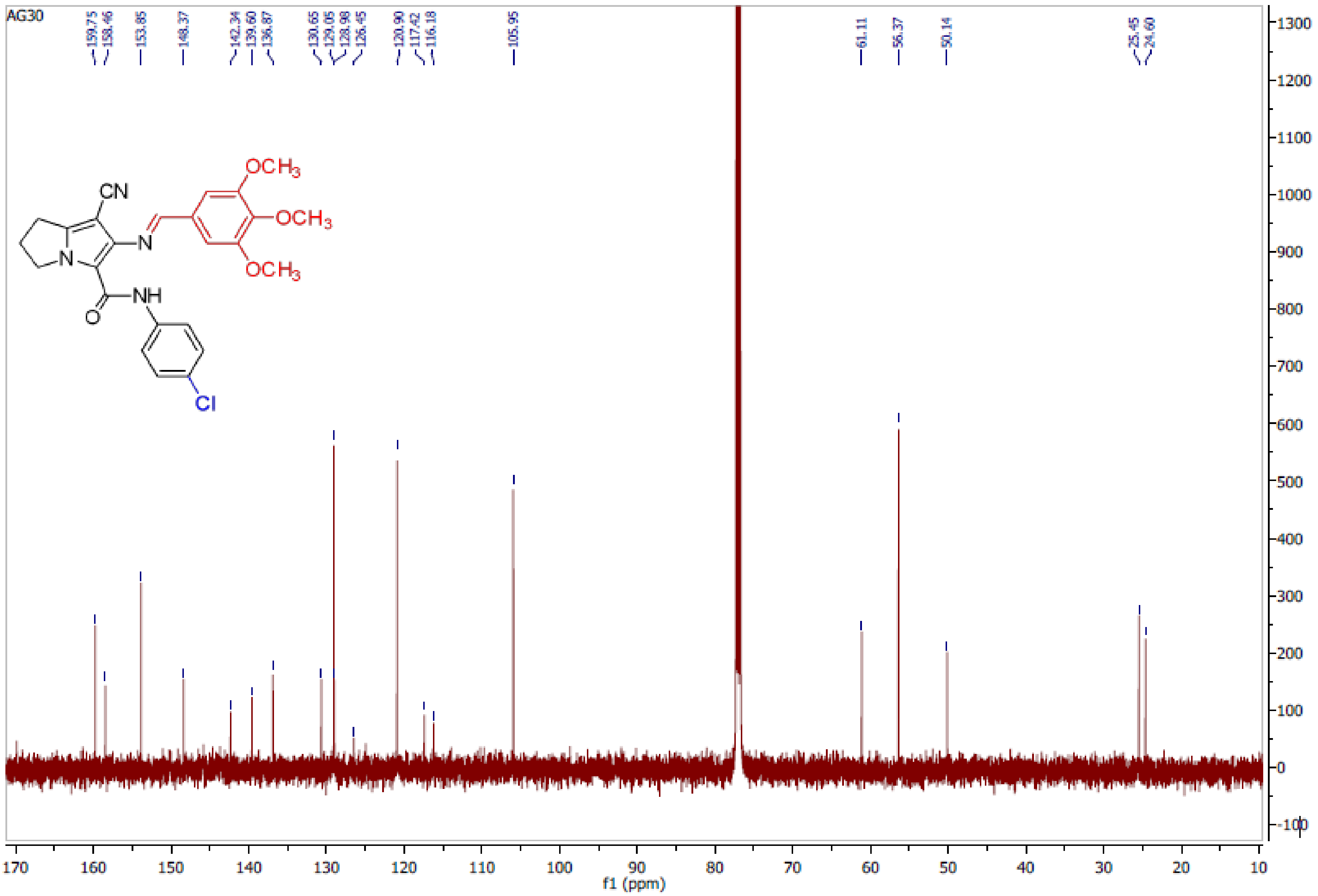

Fig. S41. ^{13}C-NMR ($CDCl_3$, 125 MHz, δ ppm) spectrum of compound **15d** (ZOOM on aliphatic Cs)

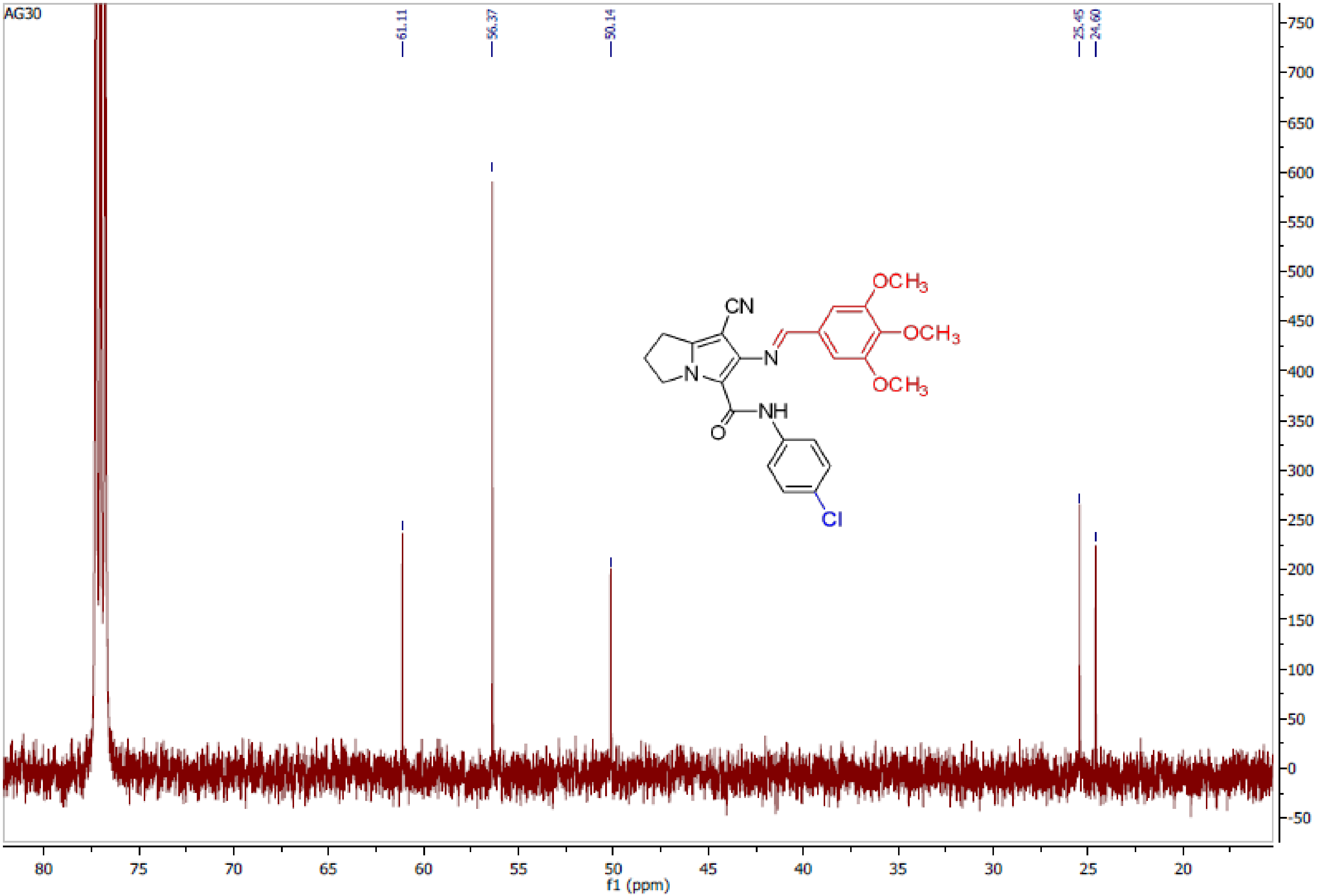

Fig. S42. ^{13}C-NMR ($CDCl_3$, 125 MHz, δ ppm) spectrum of compound **15d** (ZOOM on aromatic Cs)

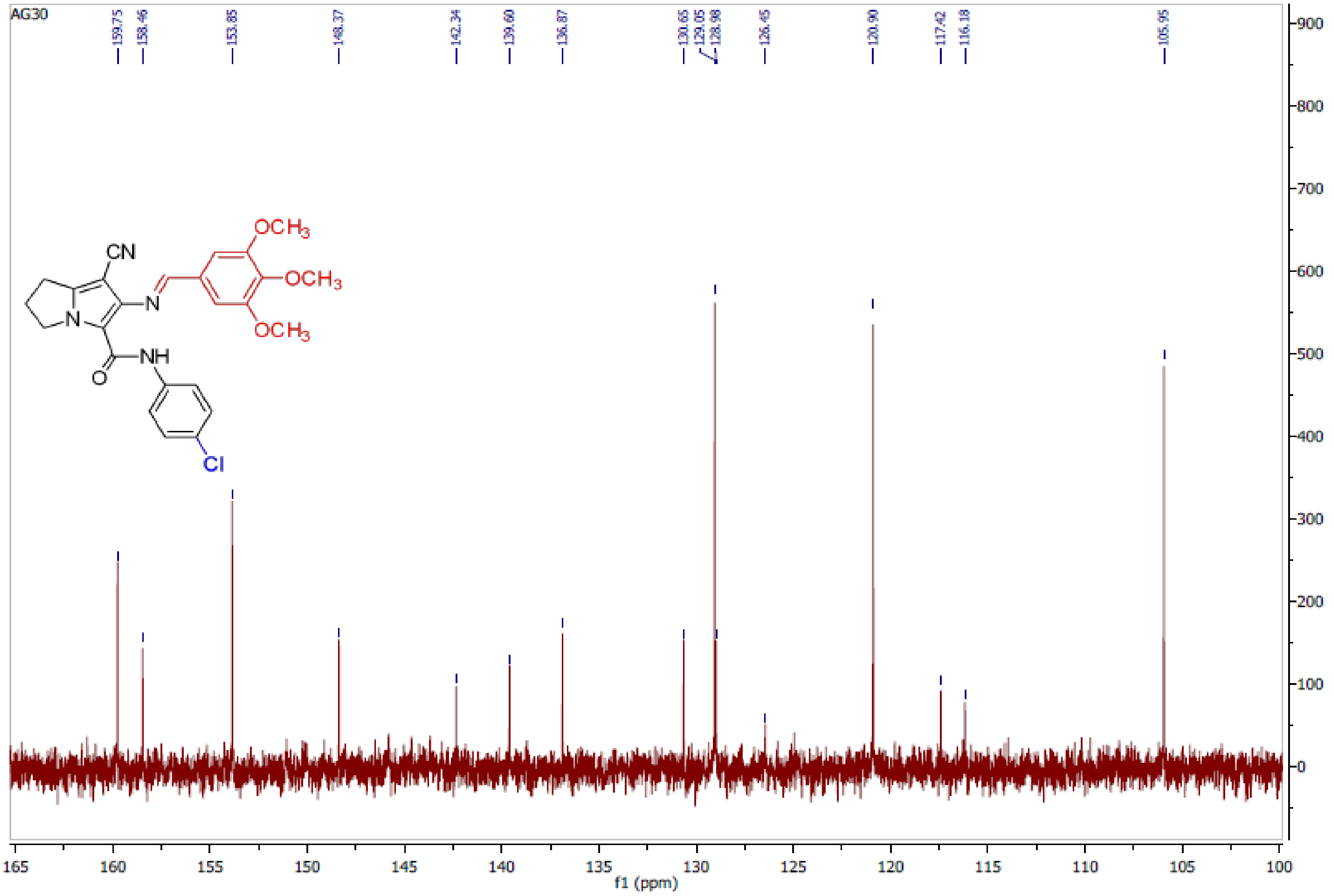

Fig. S43. **DEPT C^{135} spectrum of compound 15d**

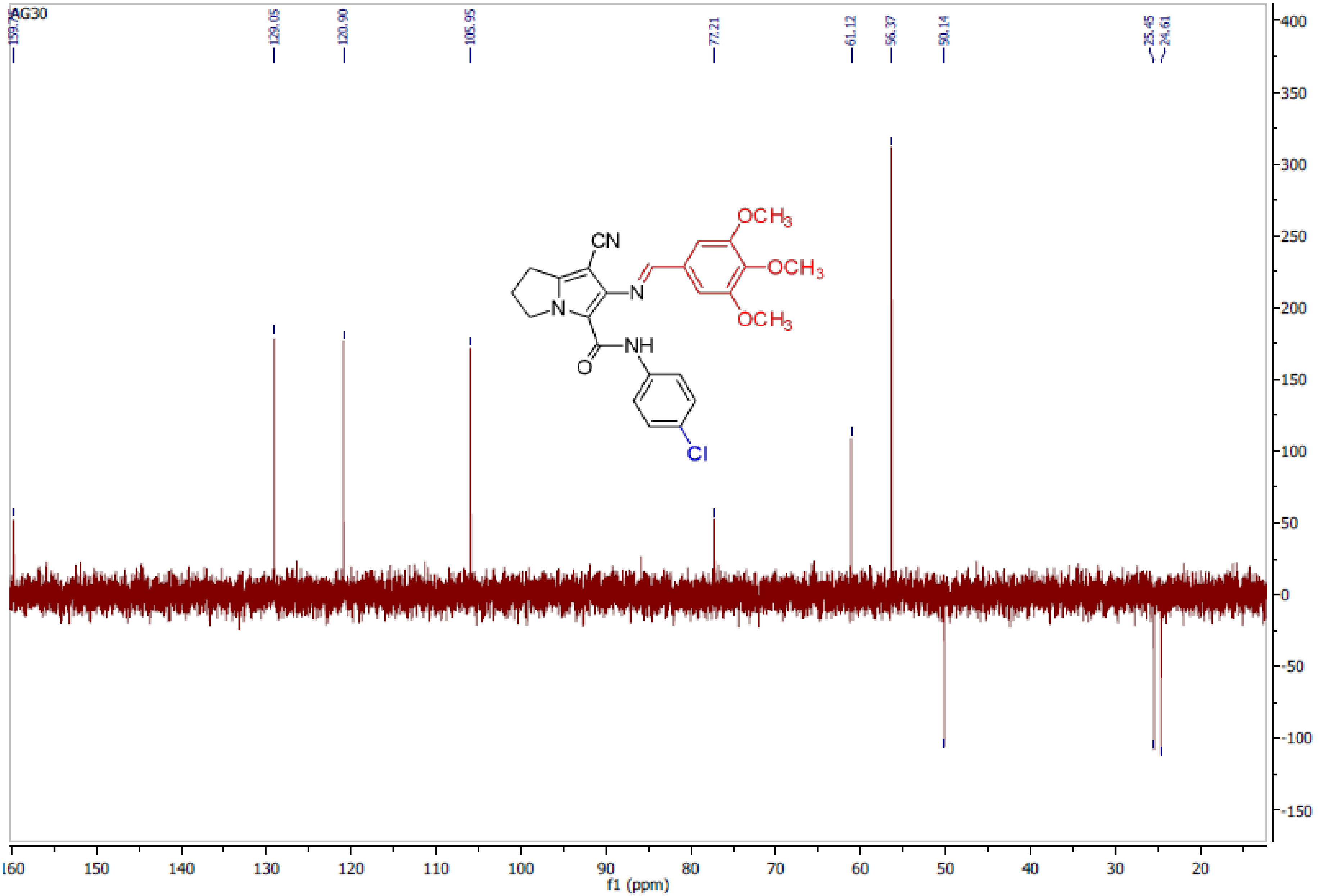

Fig. S44. ^{1}H-NMR ($CDCl_3$, 500 MHz, δ ppm) spectrum of compound **15e**

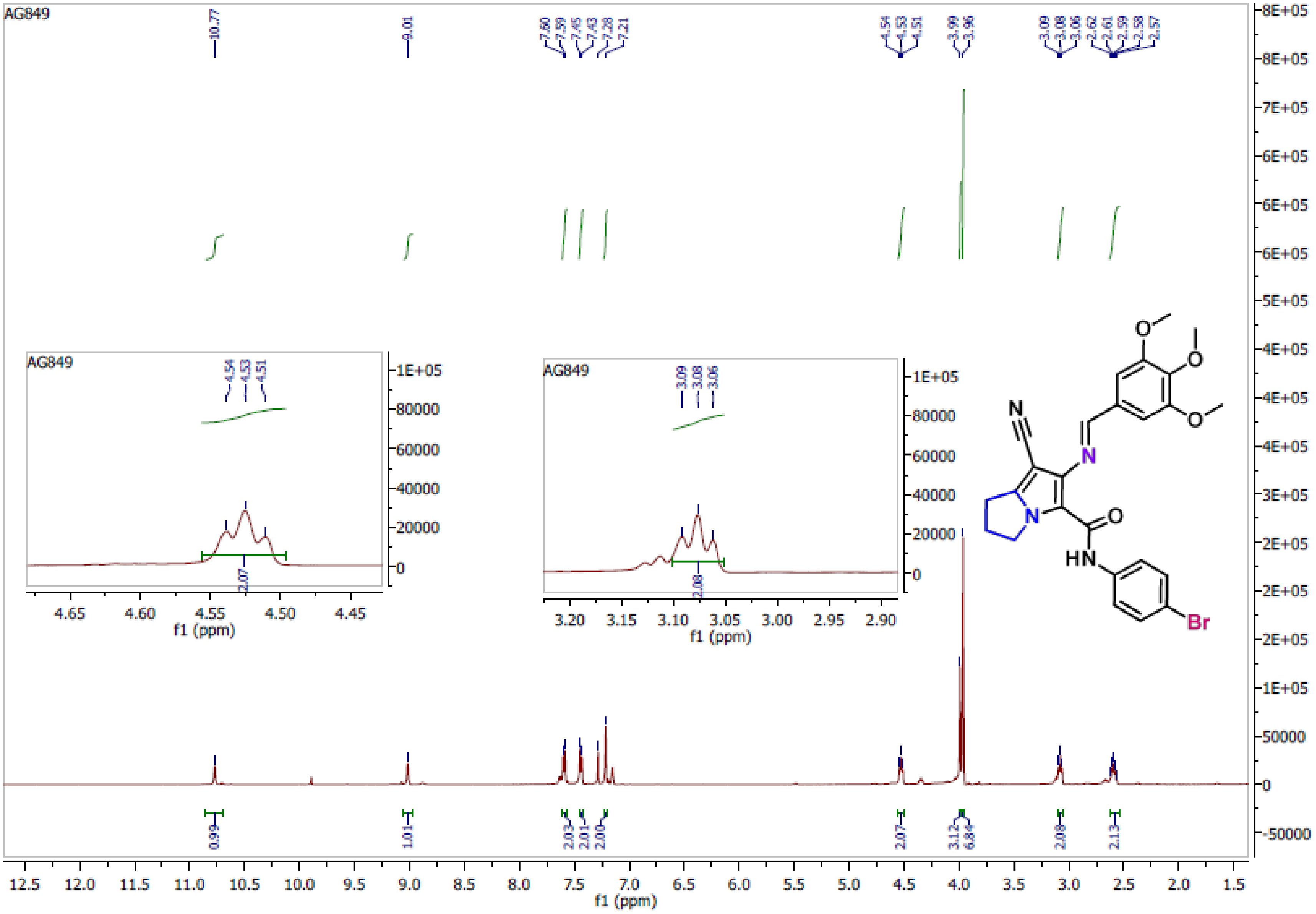

Fig. S45. ^{1}H-NMR ($CDCl_3$, 500 MHz, δ ppm) spectrum of compound **15e**

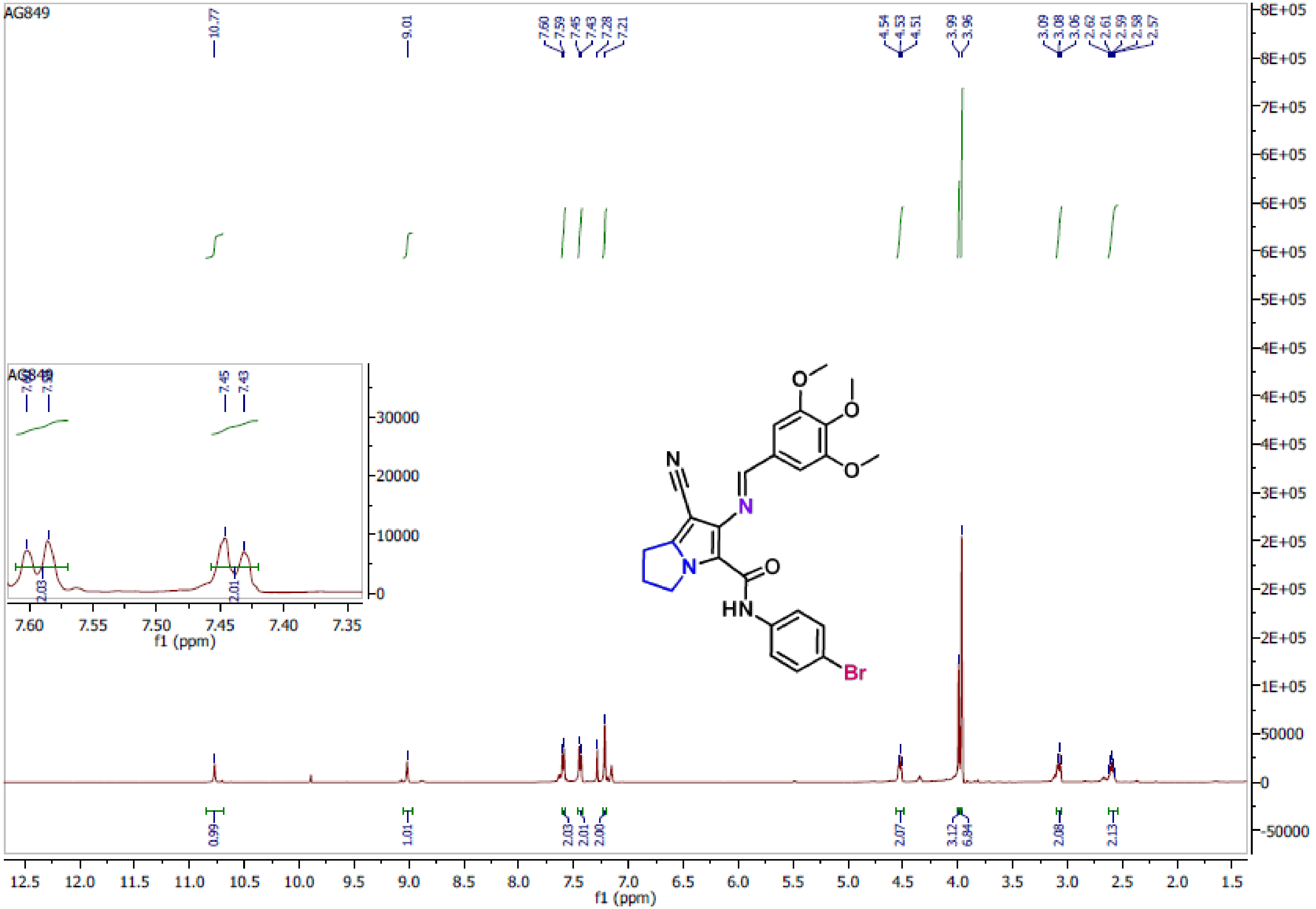

Fig. S46. ^{1}H-NMR ($CDCl_3$, 500 MHz, δ ppm) spectrum of compound **15e** (aliphatic Hs)

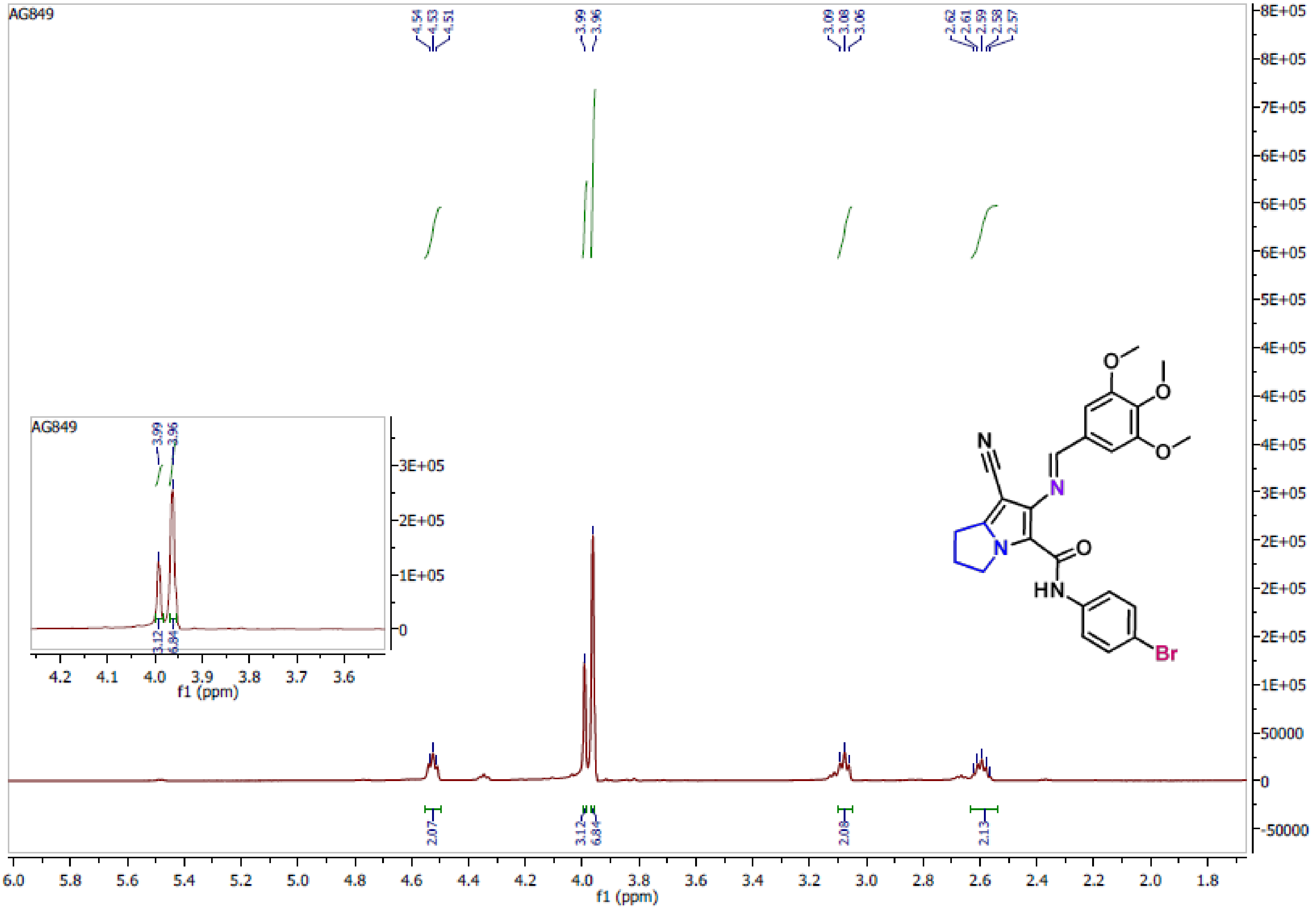

Fig. S47. ^{13}C-NMR ($CDCl_3$, 125 MHz, δ ppm) spectrum of compound **15e**

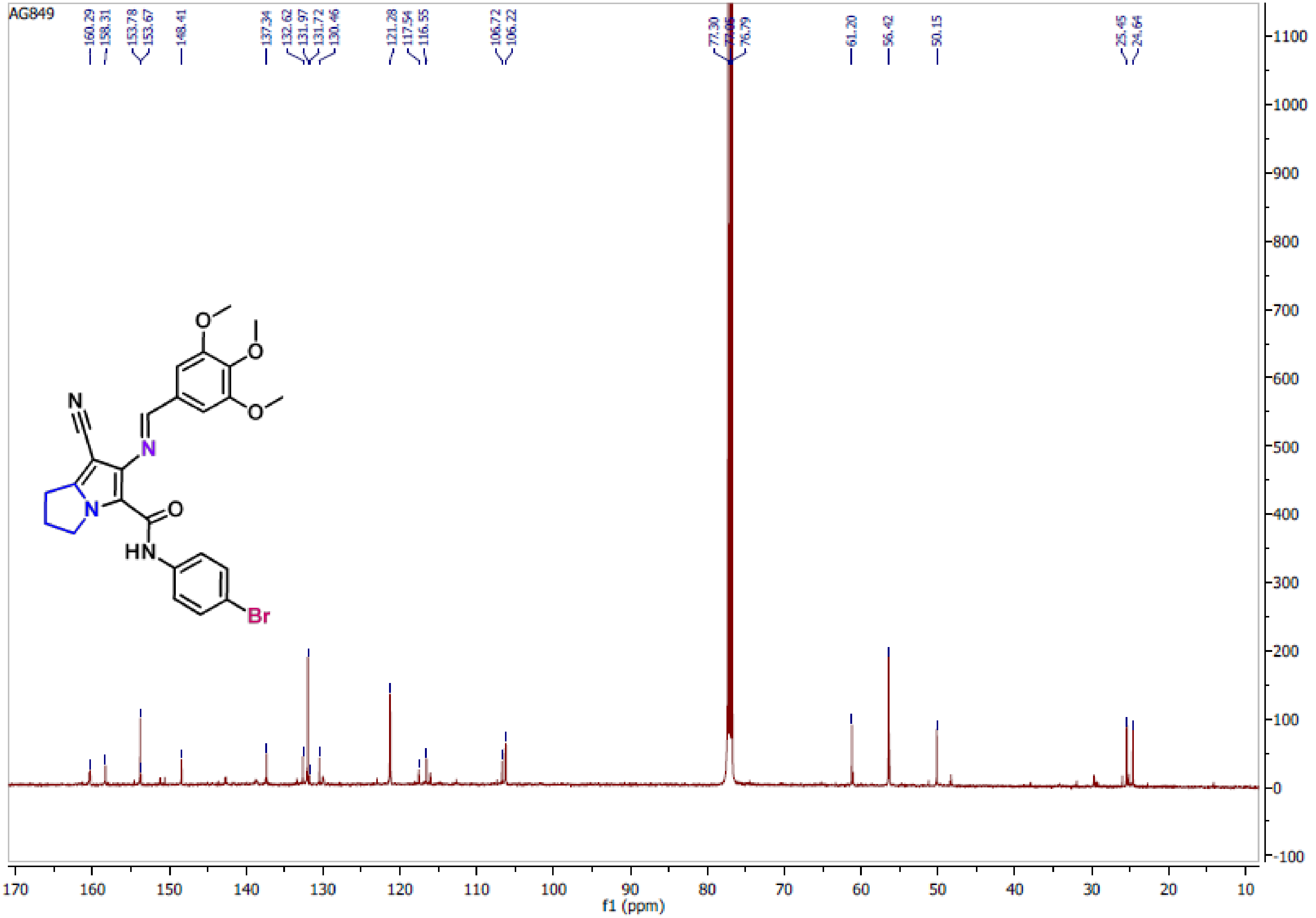

Fig. S48. **DEPT C135 ($CDCl_3$, 125 MHz, δ ppm) of compound 15e**

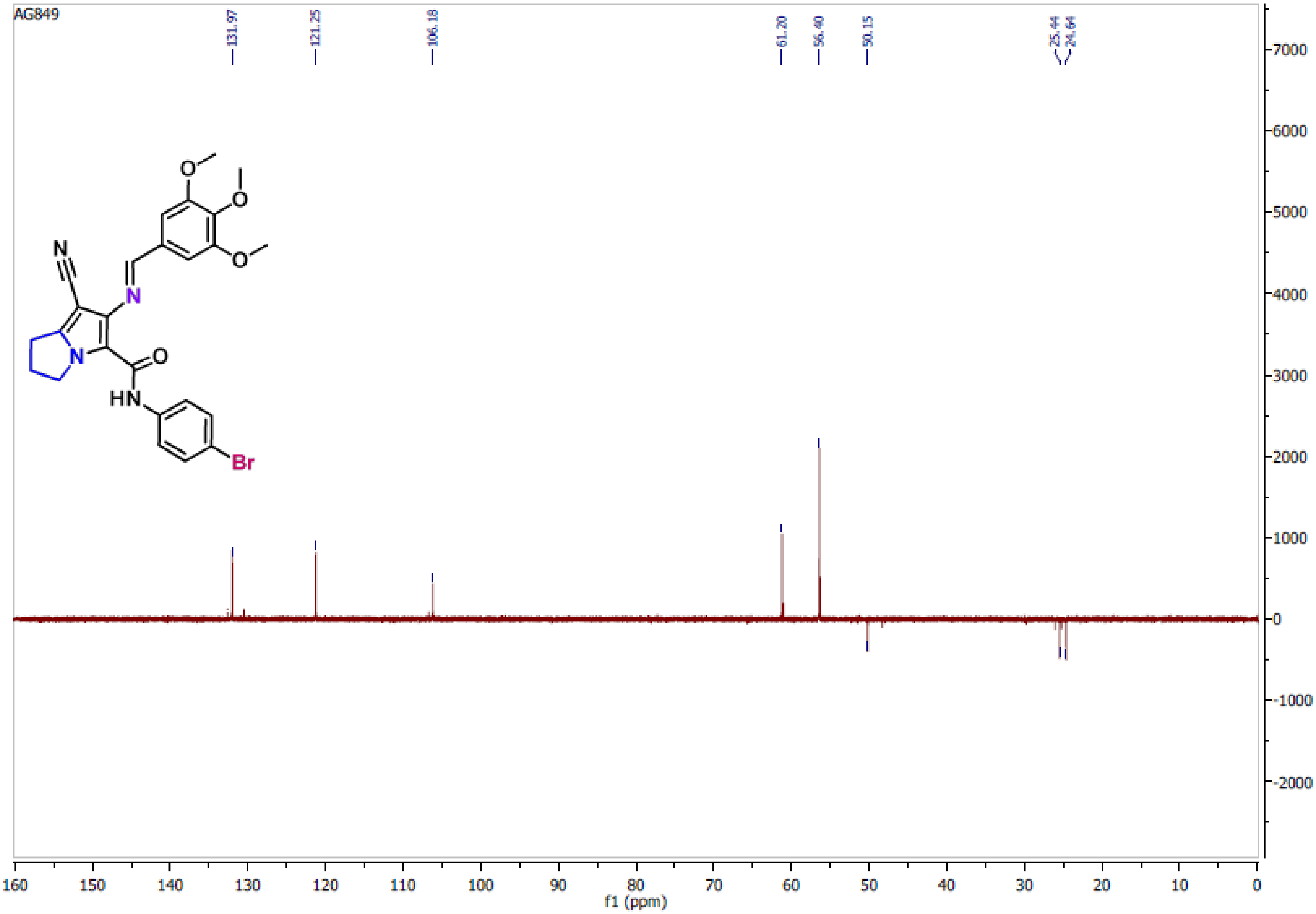

Fig. S49. ^{1}H-NMR ($CDCl_3$, 500 MHz, δ ppm) spectrum of compound **20**

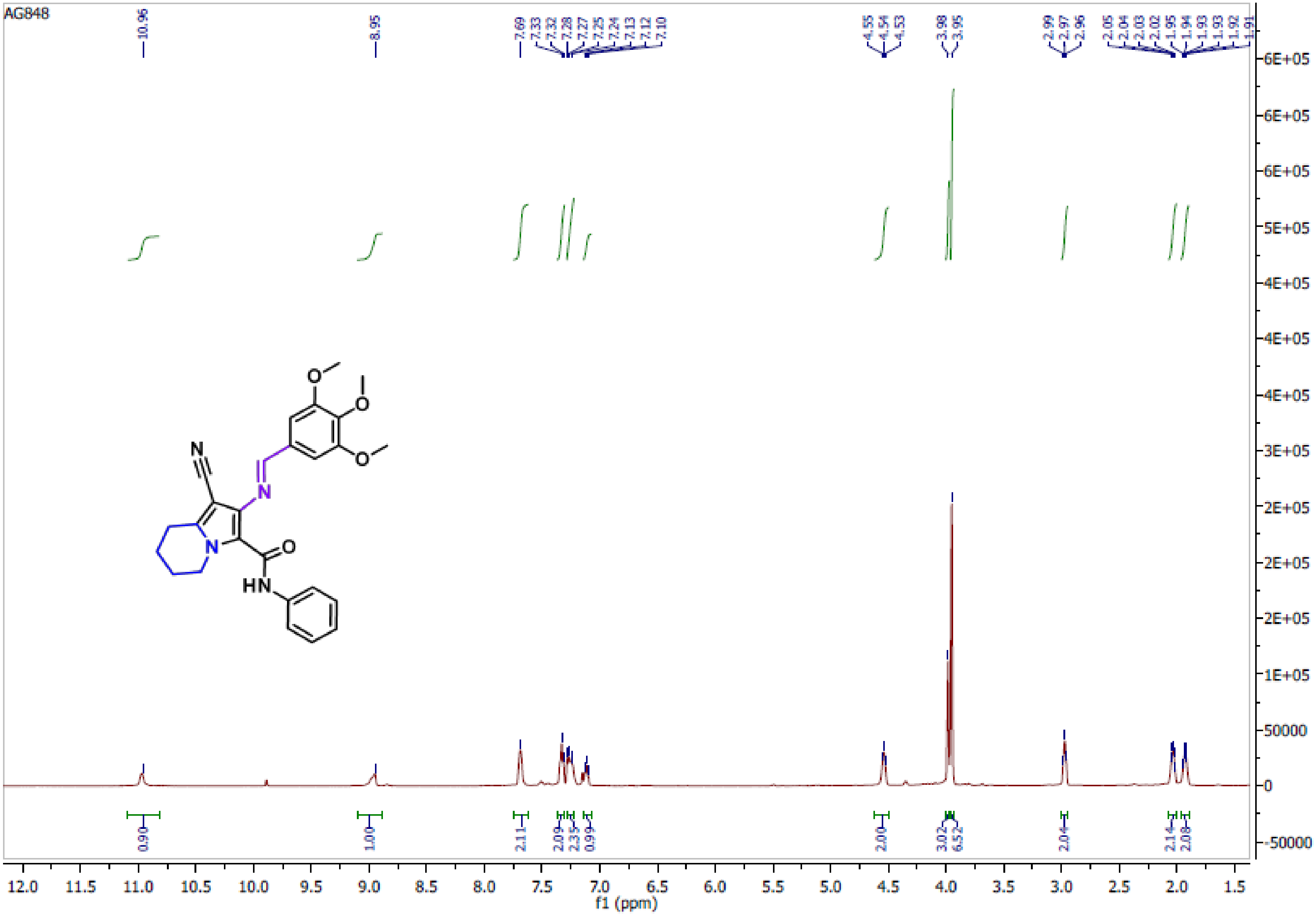

Fig. S50. ^{1}H-NMR ($CDCl_3$, 500 MHz, δ ppm) spectrum of compound **20**.

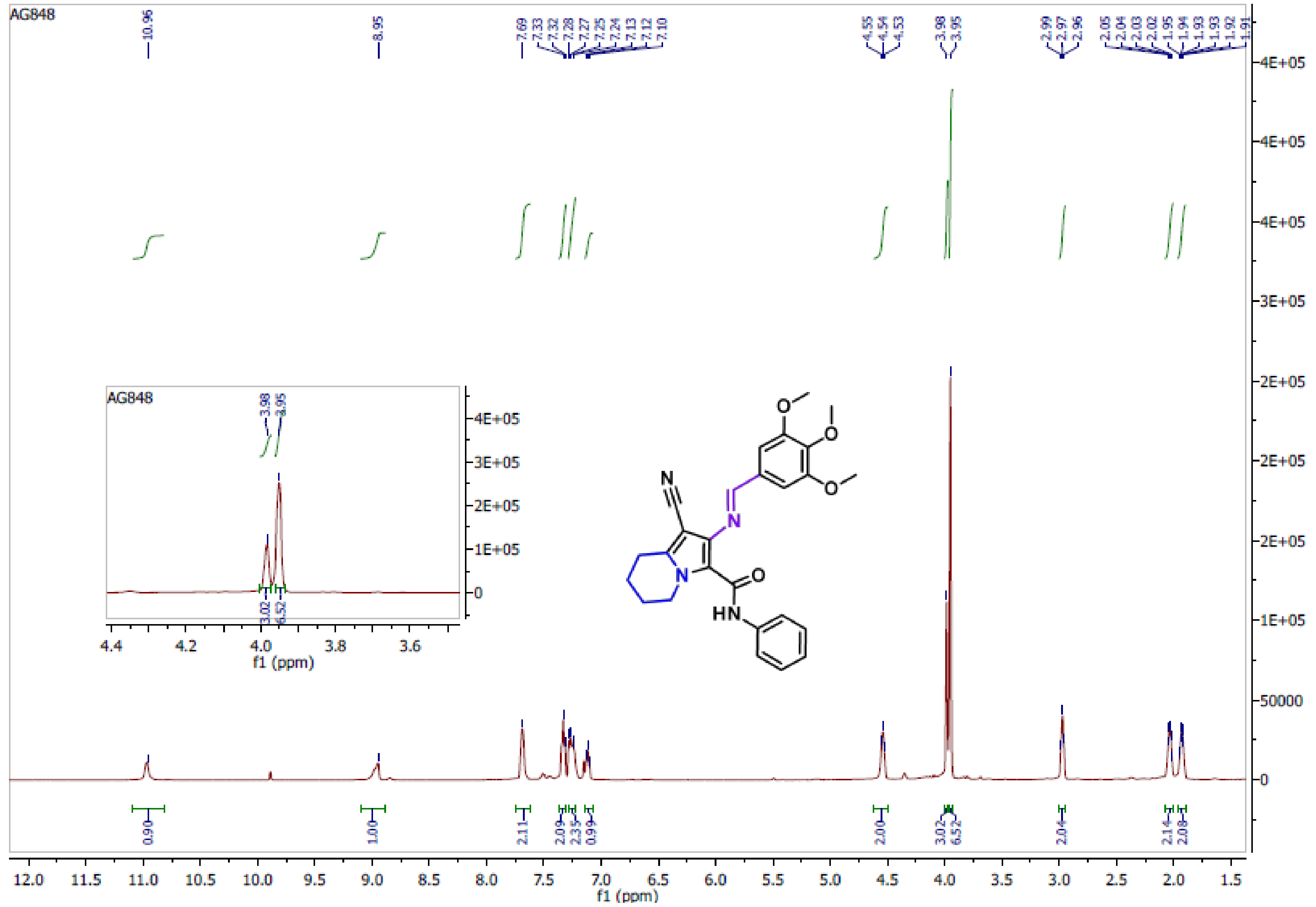

Fig. S51. ^{13}C-NMR ($CDCl_3$, 125 MHz, δ ppm) spectrum of compound **20**.

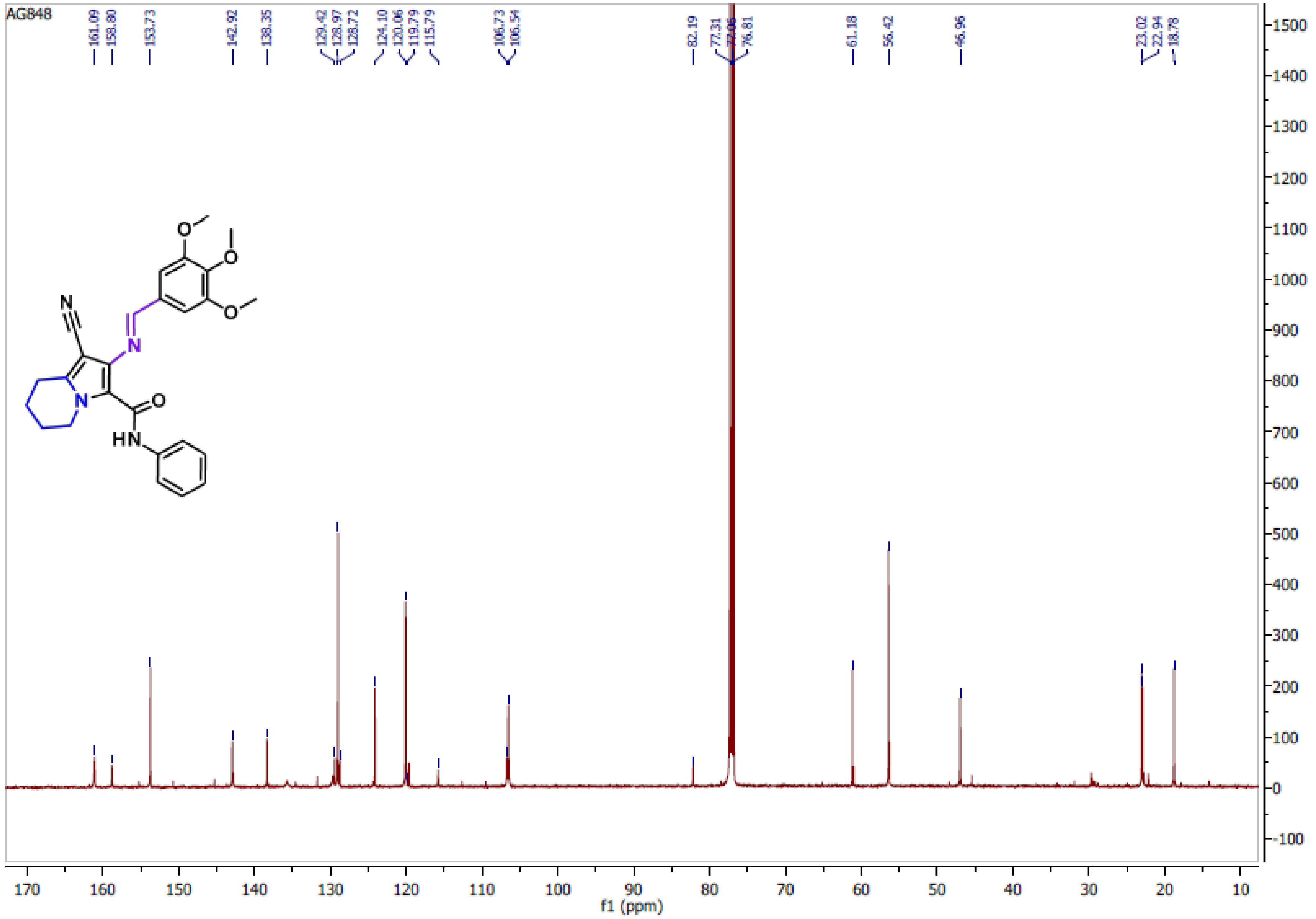

Fig. S52. ^{13}C-NMR ($CDCl_3$, 125 MHz, *δ* ppm) spectrum of compound **20**.

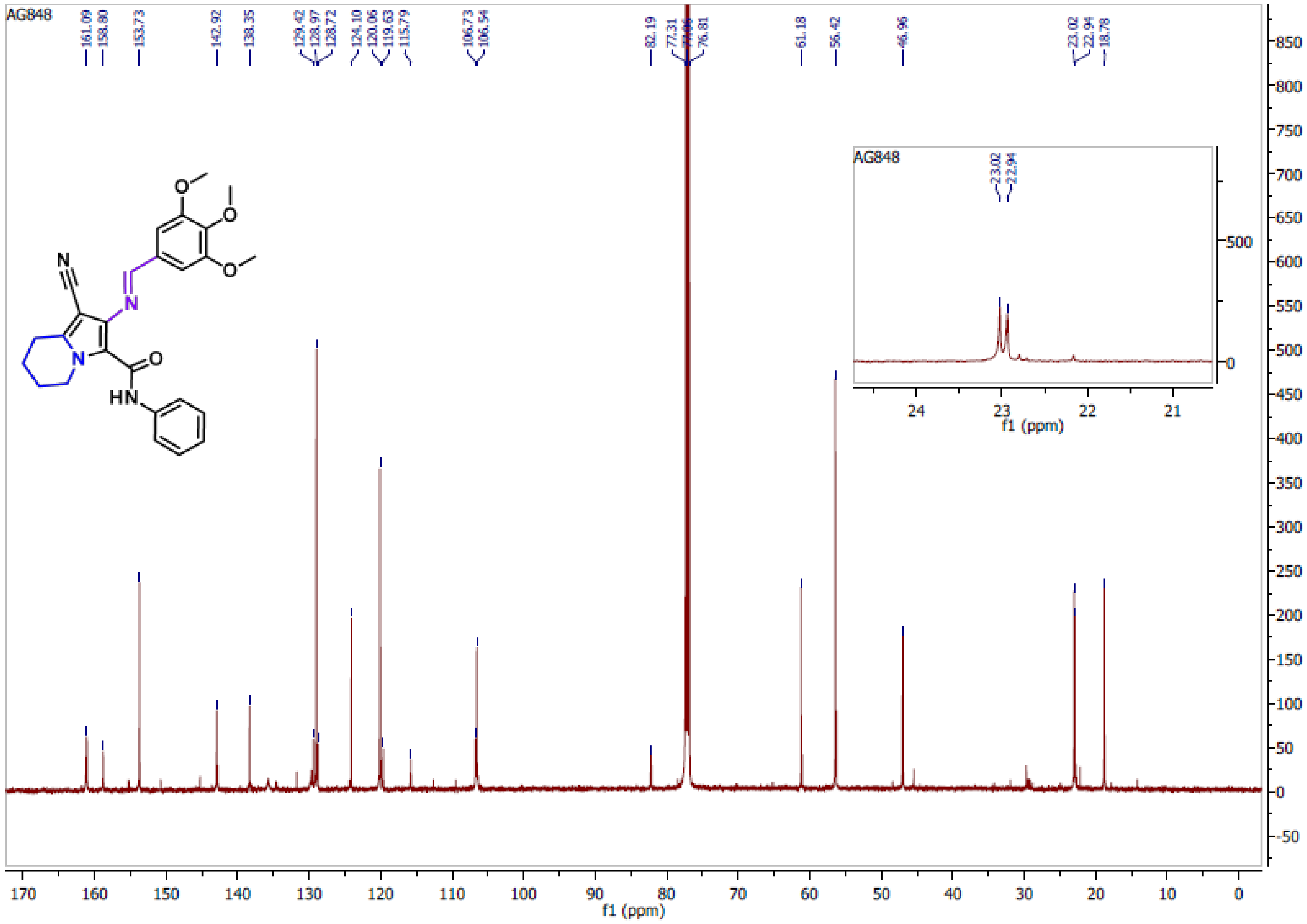

Fig. S53. **DEPT C[135] (CDCl₃, 125 MHz, *δ* ppm) of compound 20.**

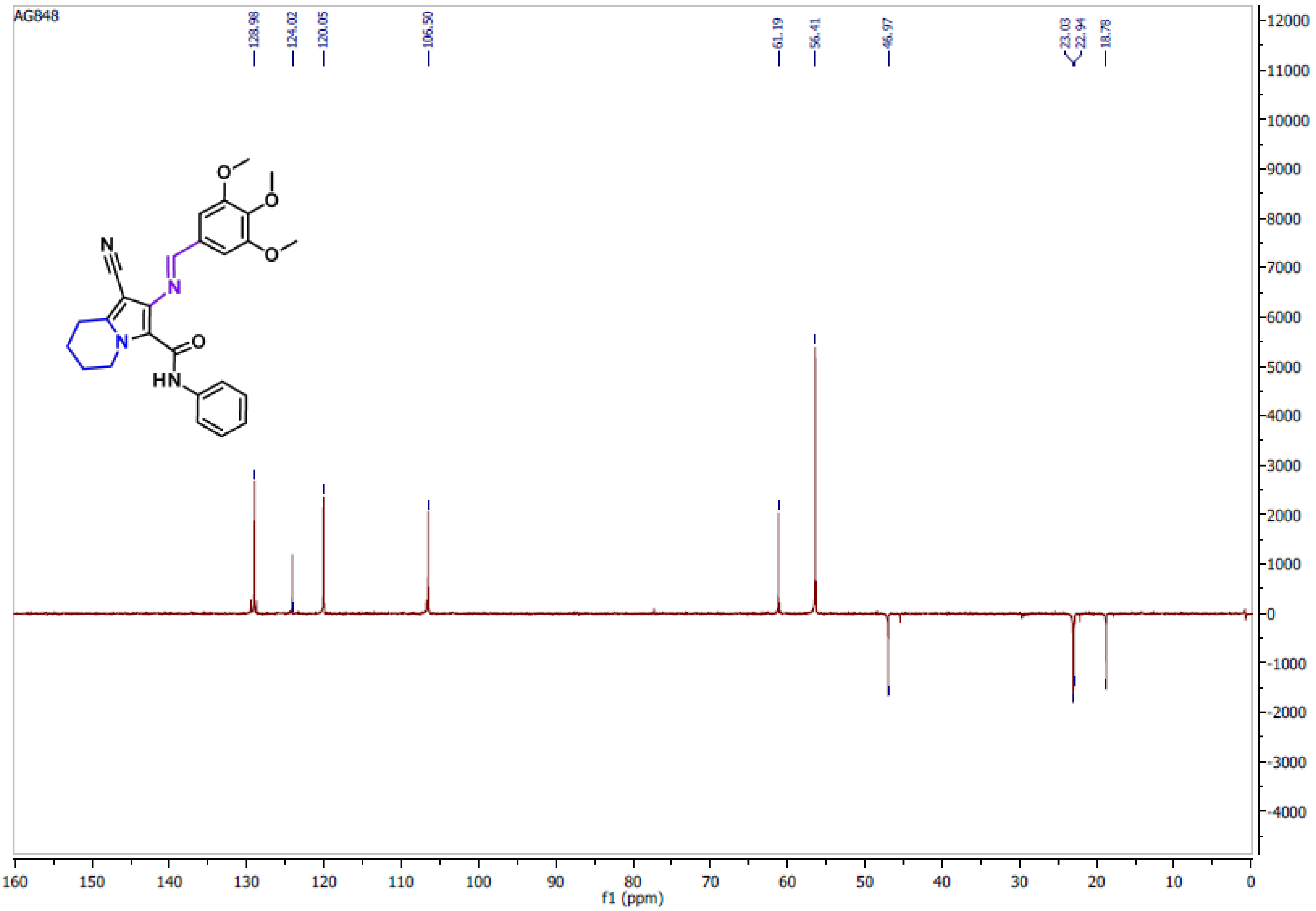

Fig. S54. **DEPT C[135] (CDCl3, 125 MHz, *δ* ppm) of compound 20.**

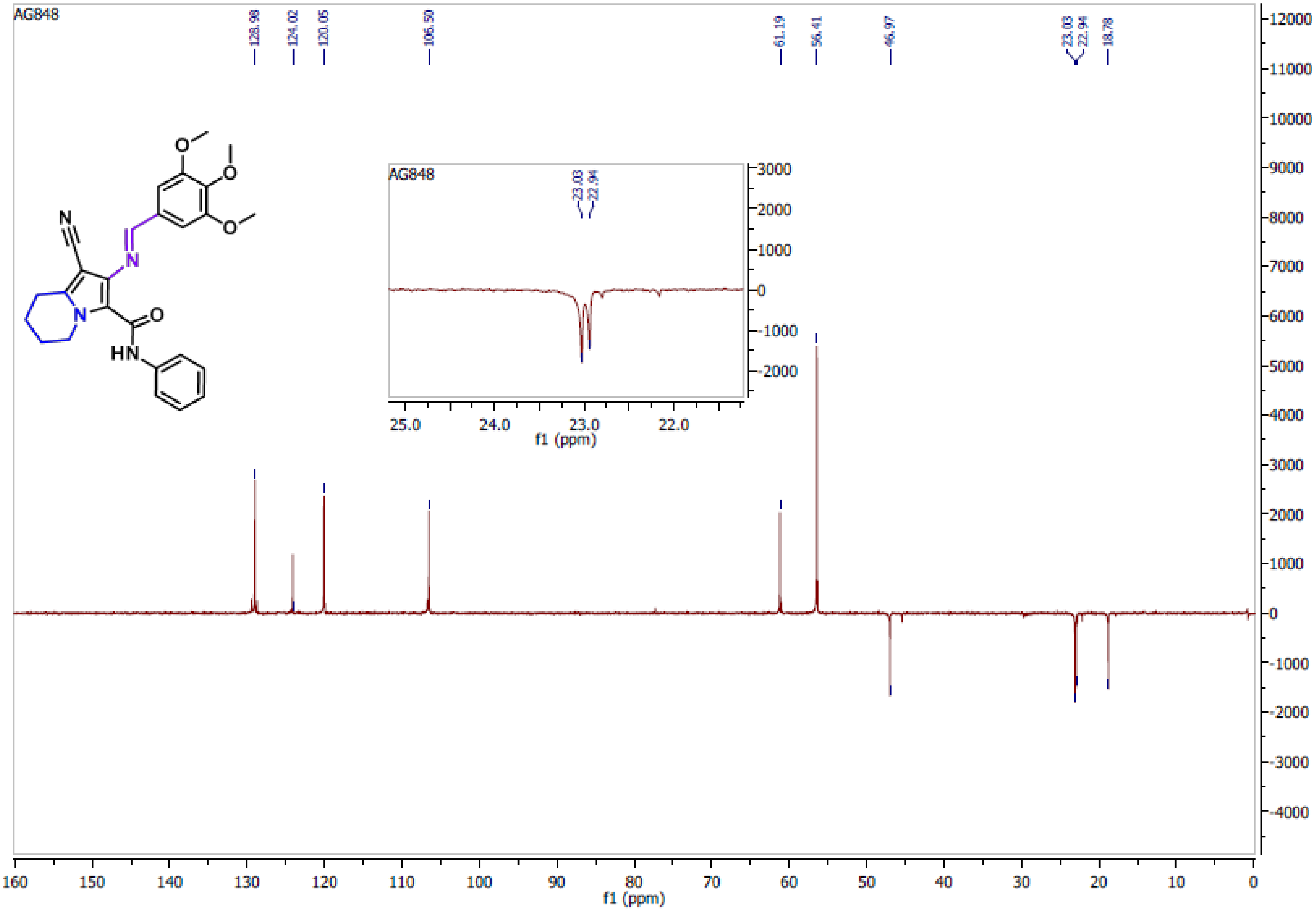

Fig. S55. ^{1}H-NMR (DMSO, 500 MHz, δ ppm) spectrum of compound **16a**

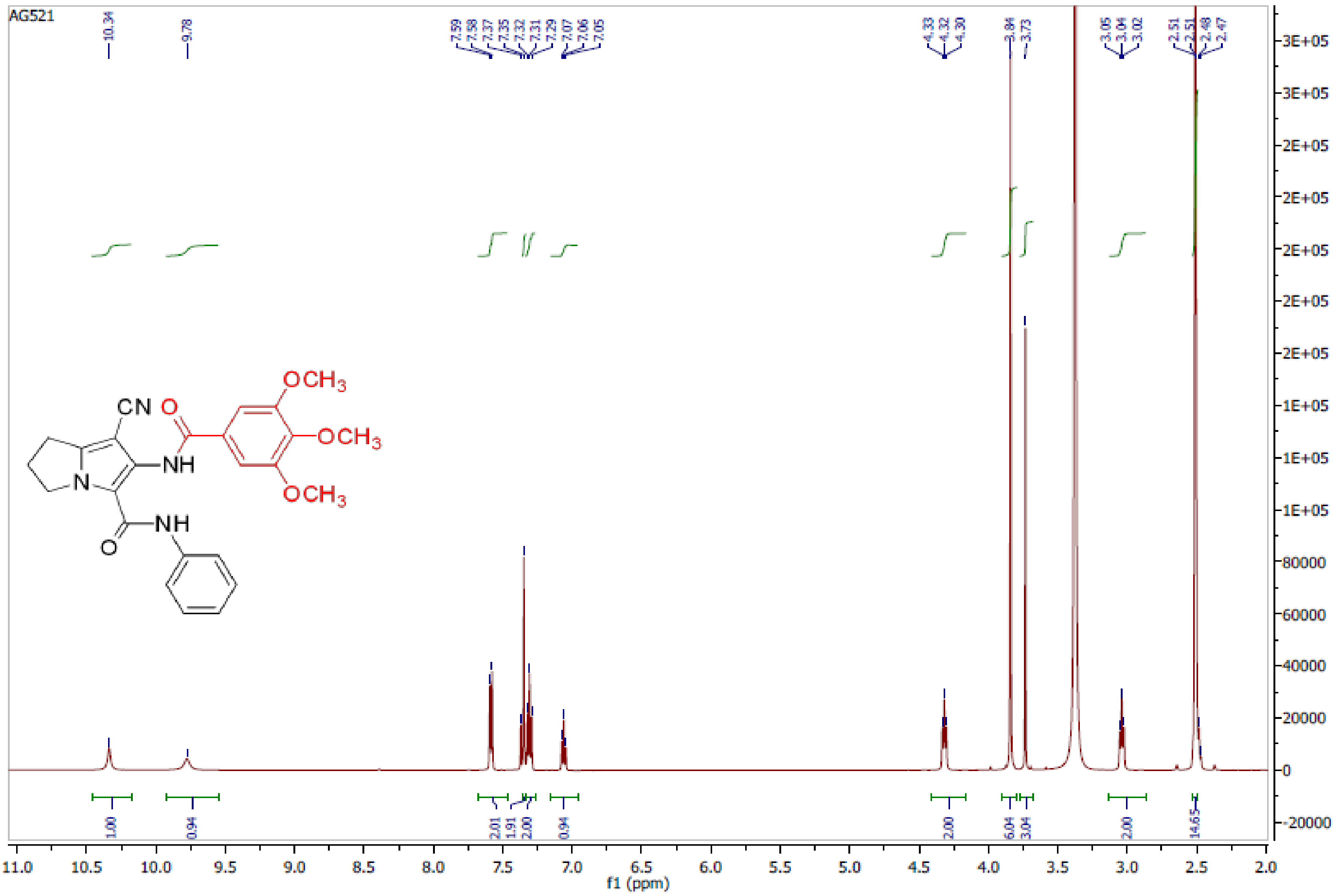

Fig. S56. ^{1}H-NMR (DMSO, 500 MHz, δ ppm) spectrum of compound 16a (ZOOM on aliphatic Hs)

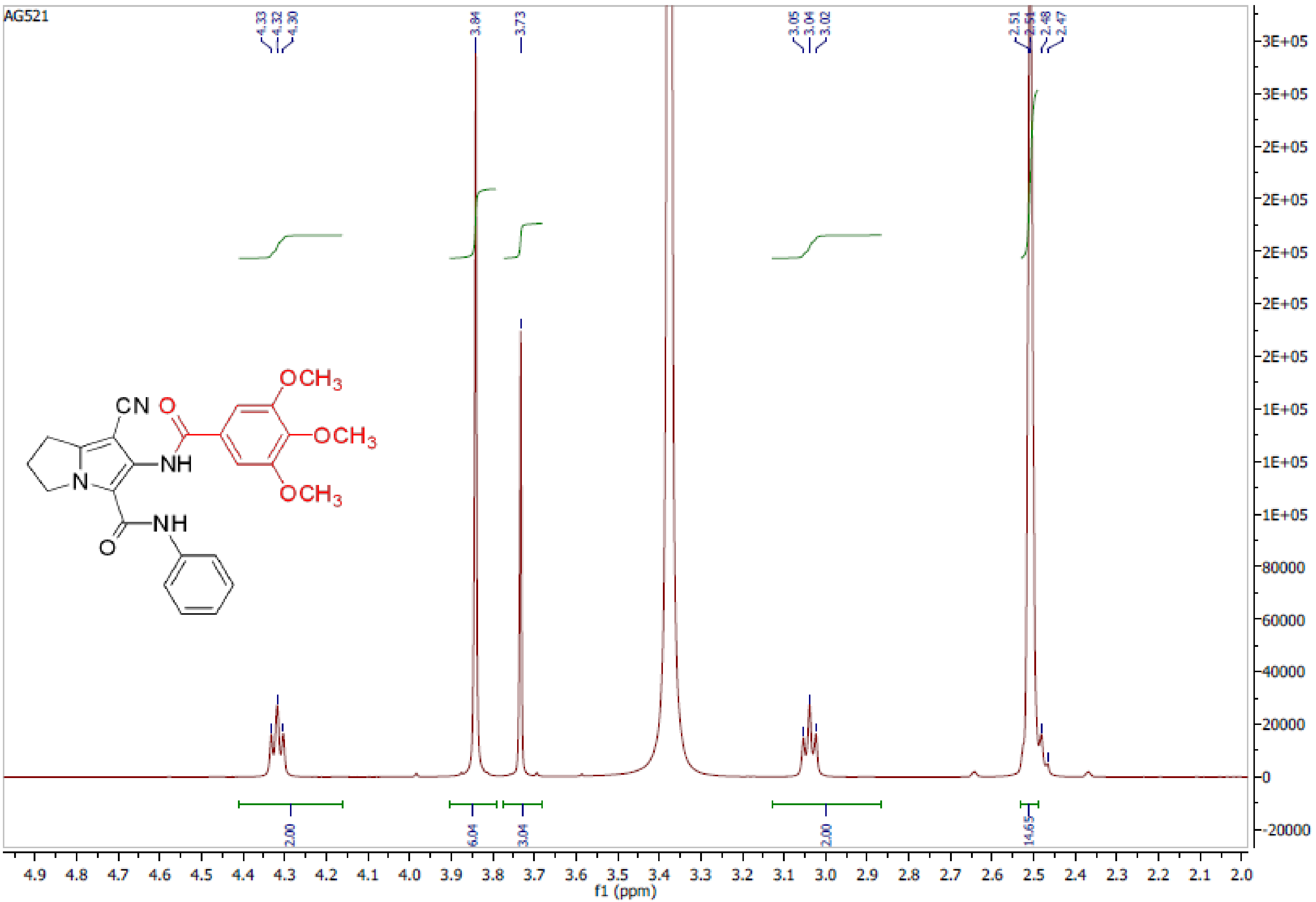

Fig. S57. ^{1}H-NMR (DMSO, 500 MHz, δ ppm) spectrum of compound **16a** (**ZOOM on NHs & aromatic Hs**)

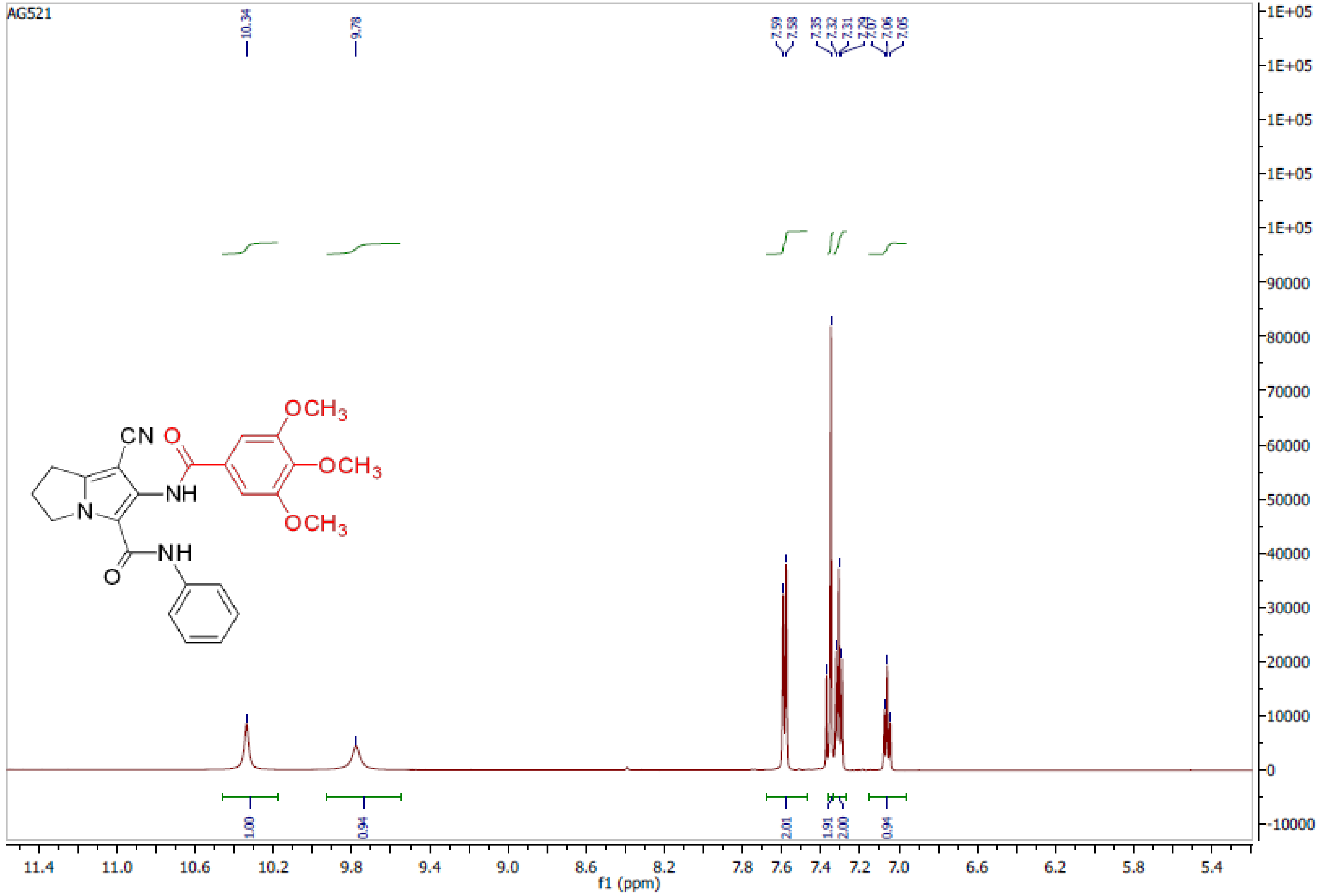

Fig. S58. ^{1}H-NMR (DMSO, 500 MHz, δ ppm) spectrum of compound **16a** (**ZOOM on aromatic Hs**)

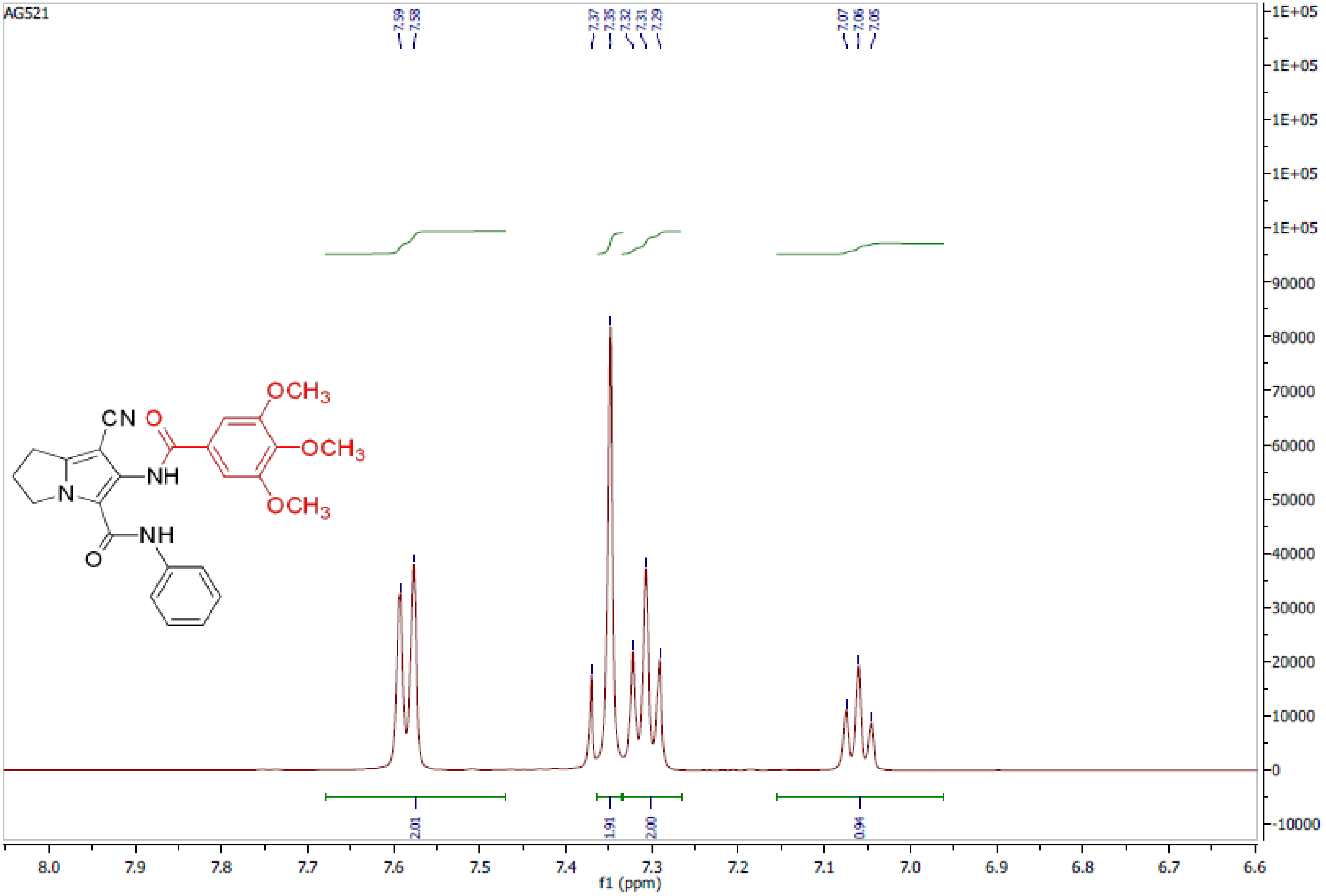

Fig. S59. ^{13}C-NMR (DMSO, 125 MHz, δ ppm) spectrum of compound **16a**

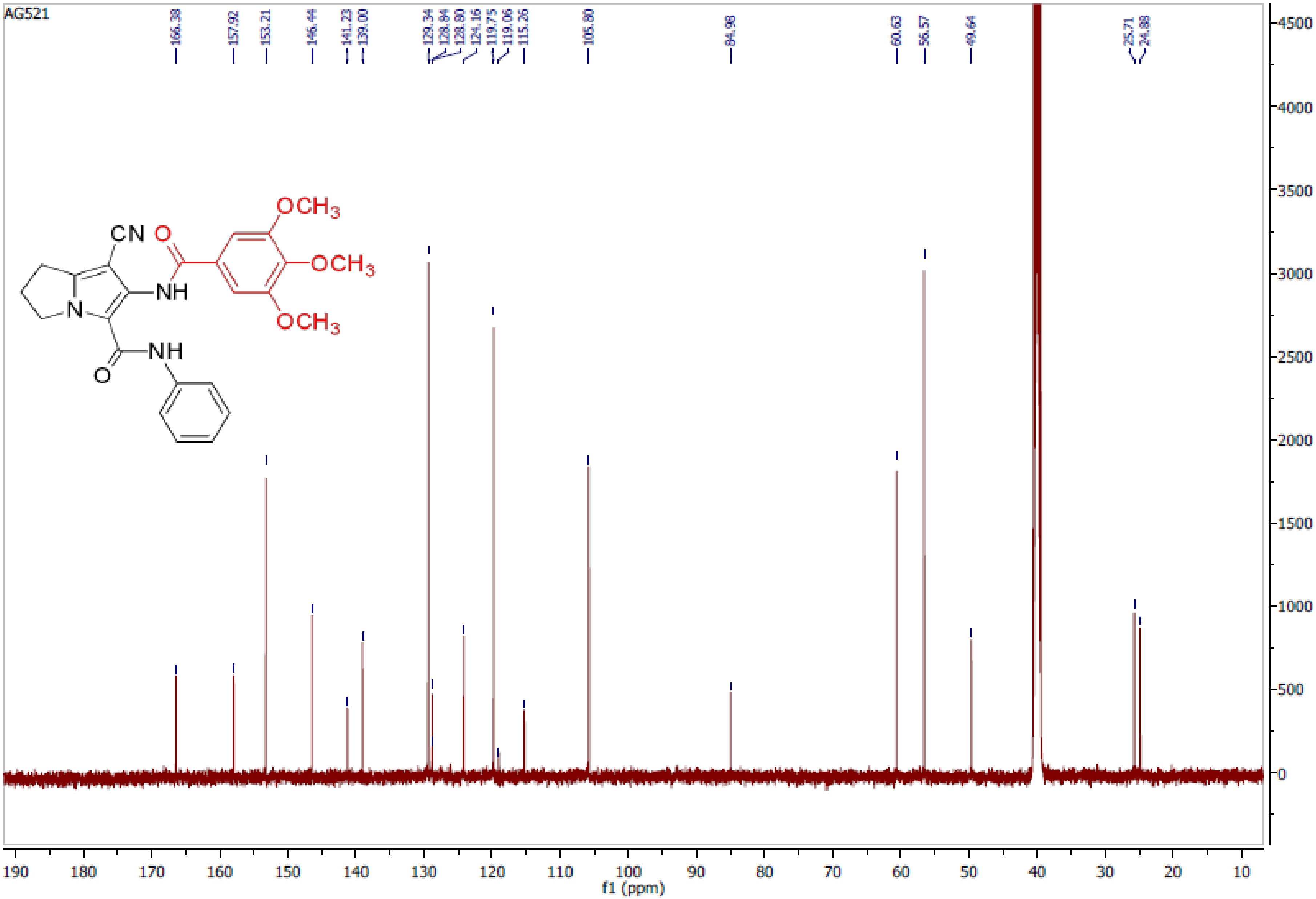

Fig. S60. ^{13}C-NMR (DMSO, 125 MHz, *δ* ppm) spectrum of compound **16a** (**ZOOM on aliphatic Cs**)

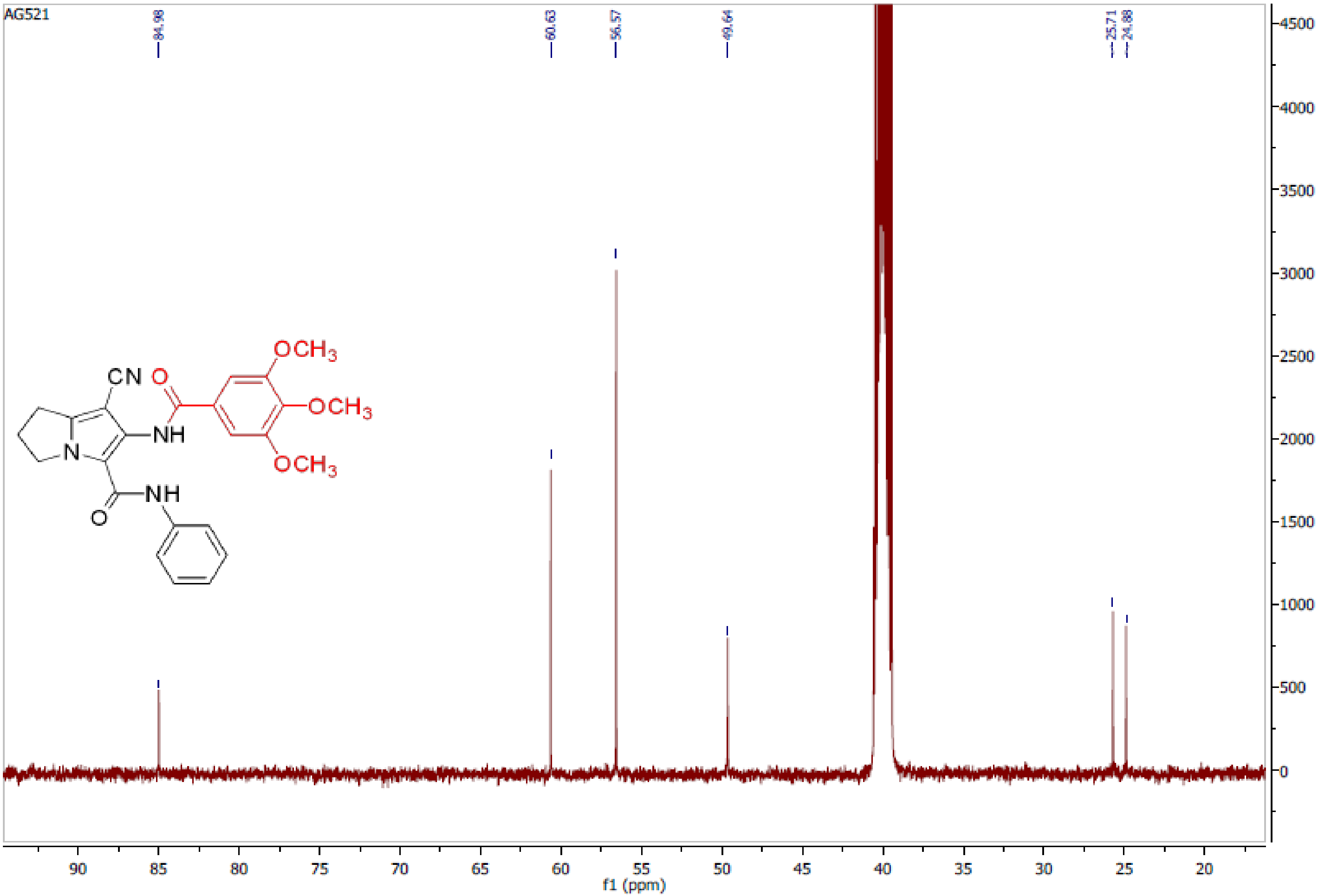

Fig. S61. ^{13}C-NMR (DMSO, 125 MHz, δ ppm) spectrum of compound **16a** (**ZOOM on aromatic Cs**)

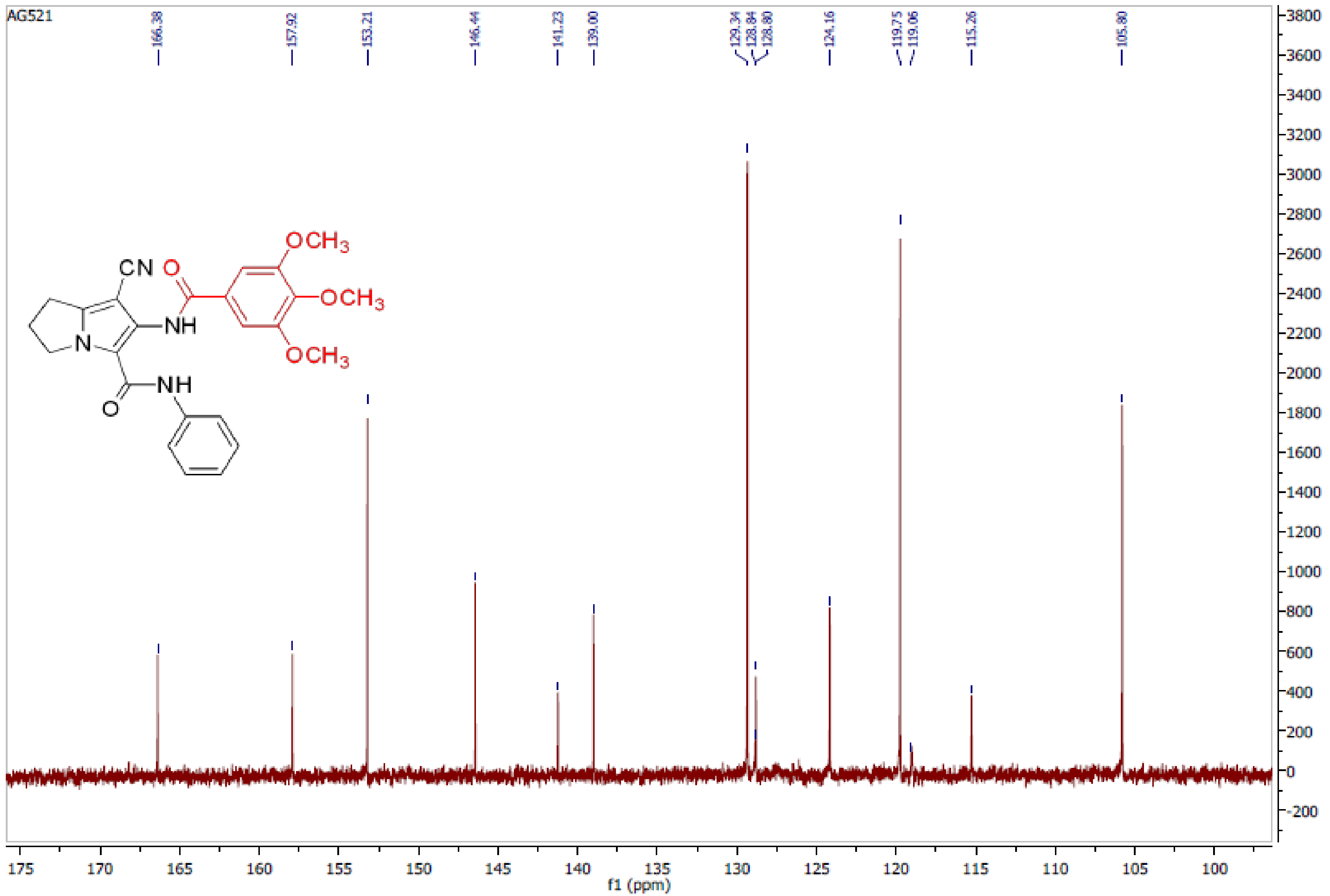

Fig. S62. DEPT C^{135} spectrum of compound **16a**

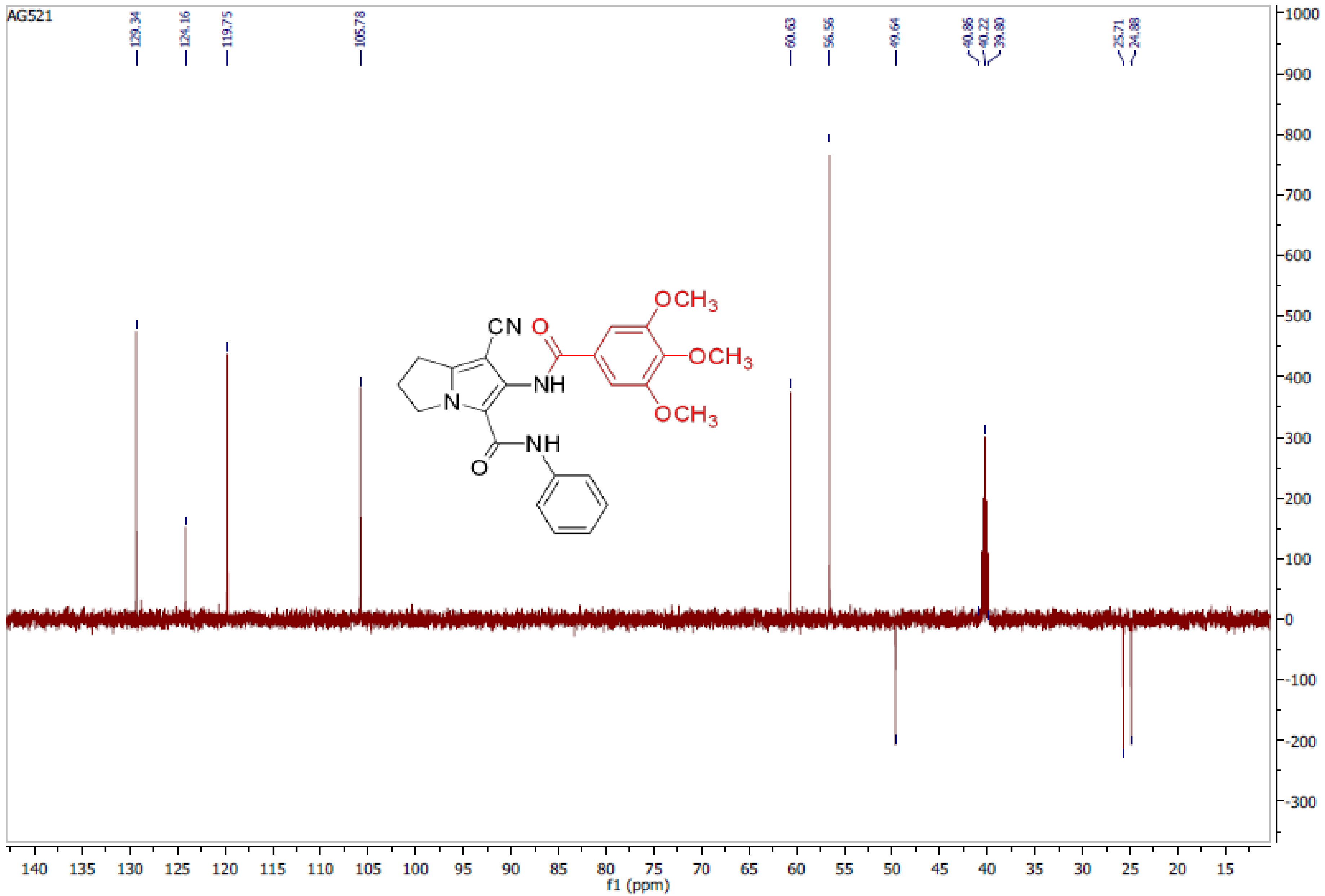

Fig. S63. ^{1}H-NMR (DMSO, 500 MHz, δ ppm) spectrum of compound **16b**

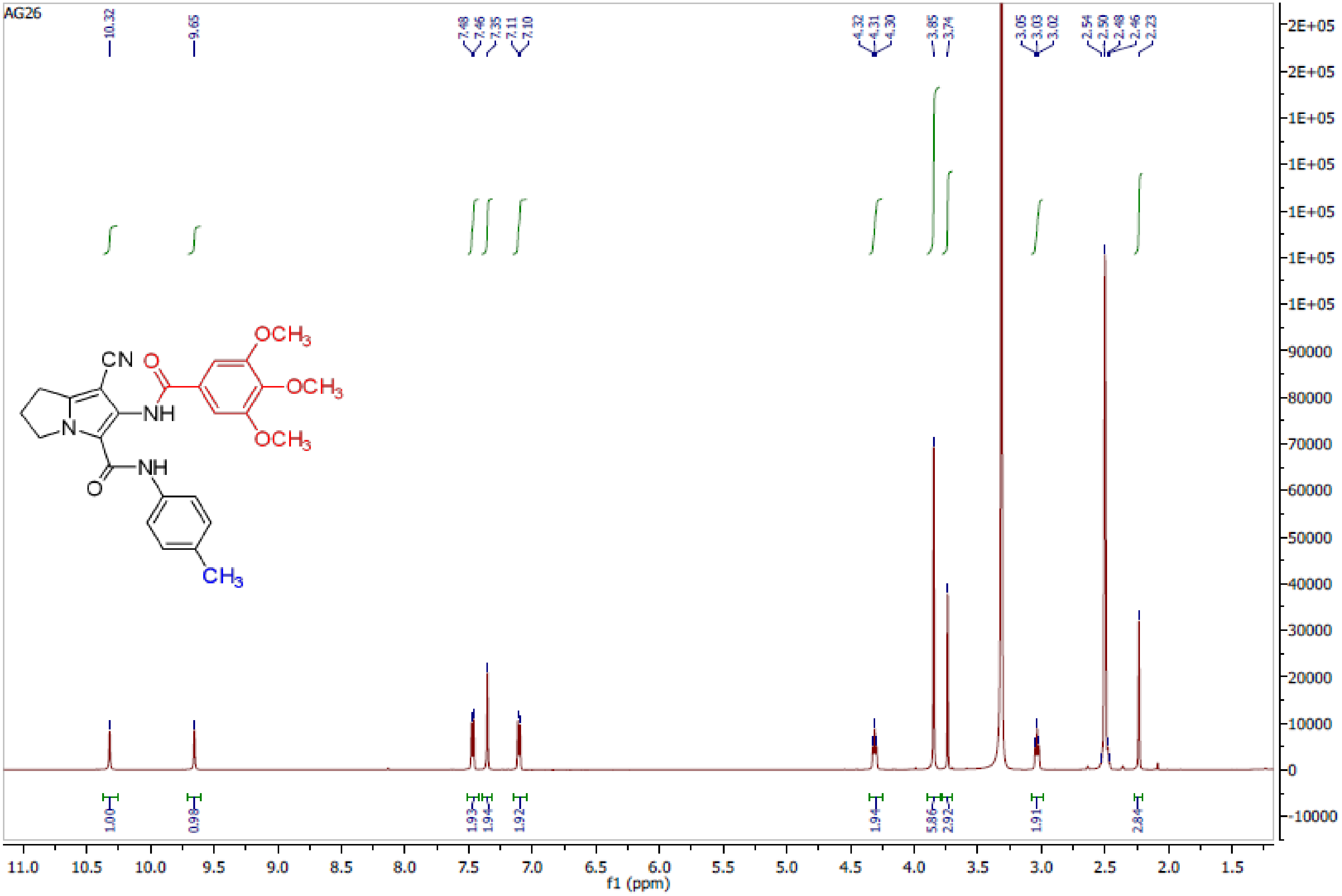

Fig. S64. ^{1}H-NMR (DMSO, 500 MHz, δ ppm) spectrum of compound **16b** (**ZOOM on aliphatic Hs**)

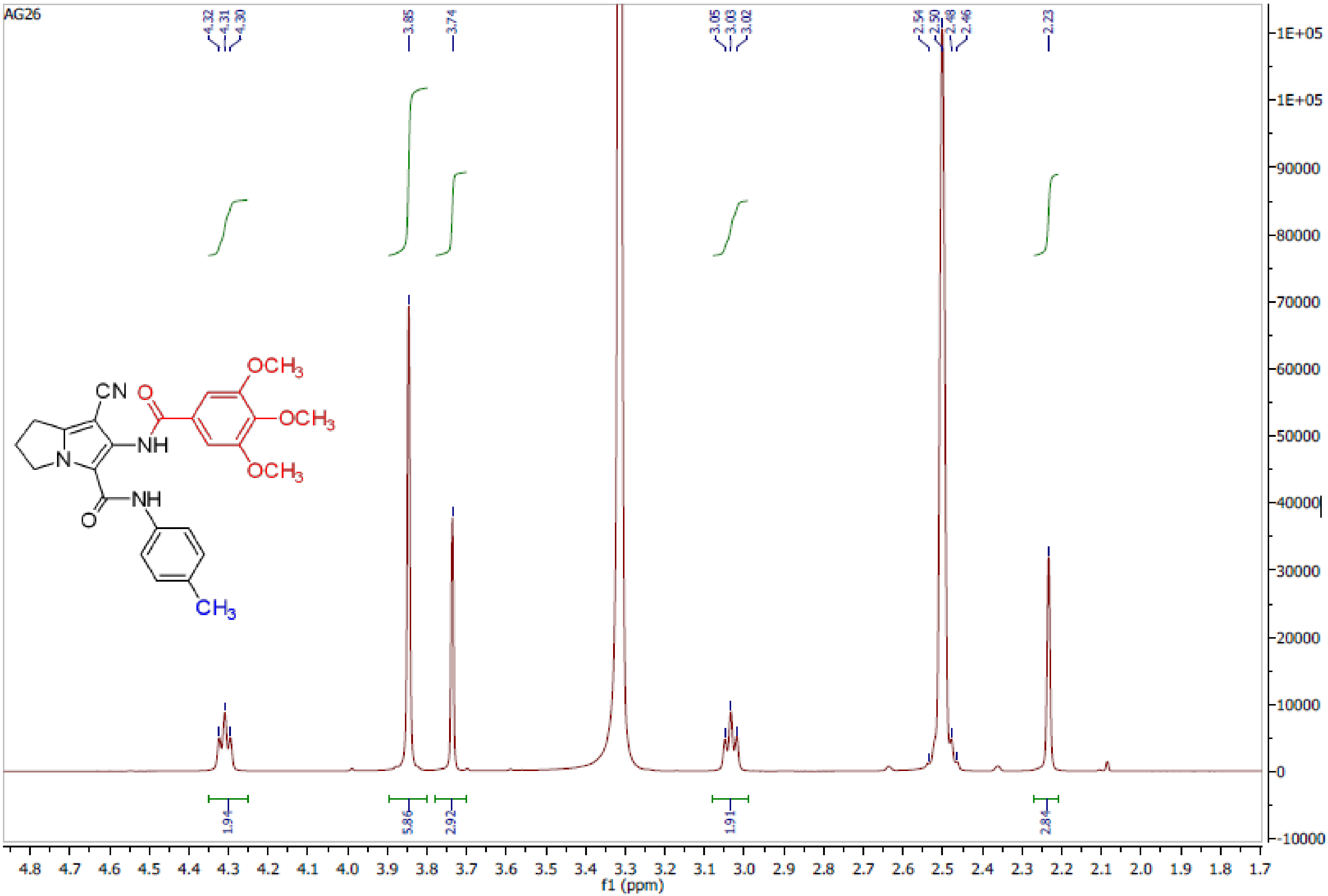

Fig. S65. ^{1}H-NMR (DMSO, 500 MHz, δ ppm) spectrum of compound **16b** (**ZOOM on NHs & aromatic Hs**)

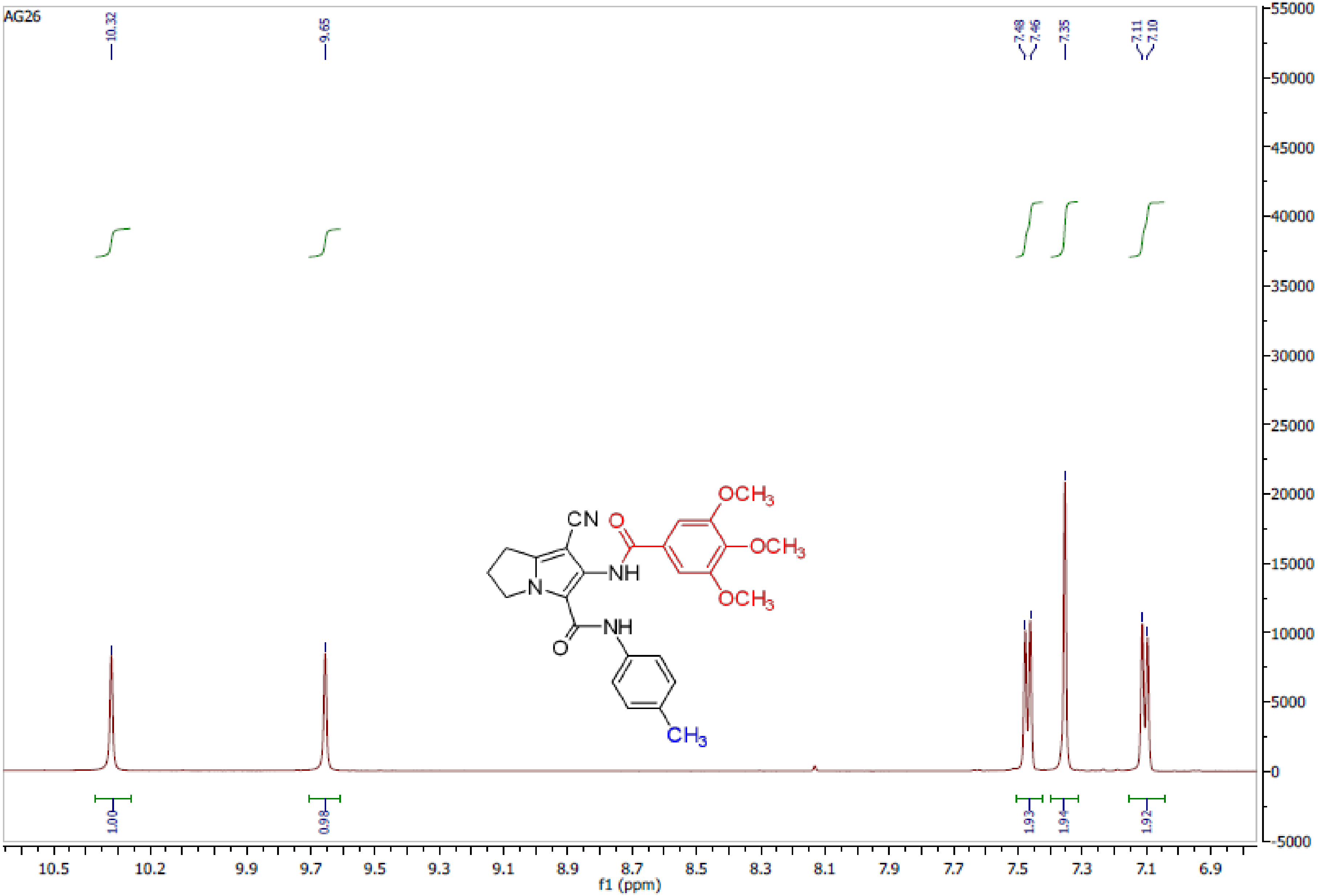

Fig. S66. ^{1}H-NMR (DMSO, 500 MHz, *δ* ppm) spectrum of compound **16b** (**ZOOM on aromatic Hs**)

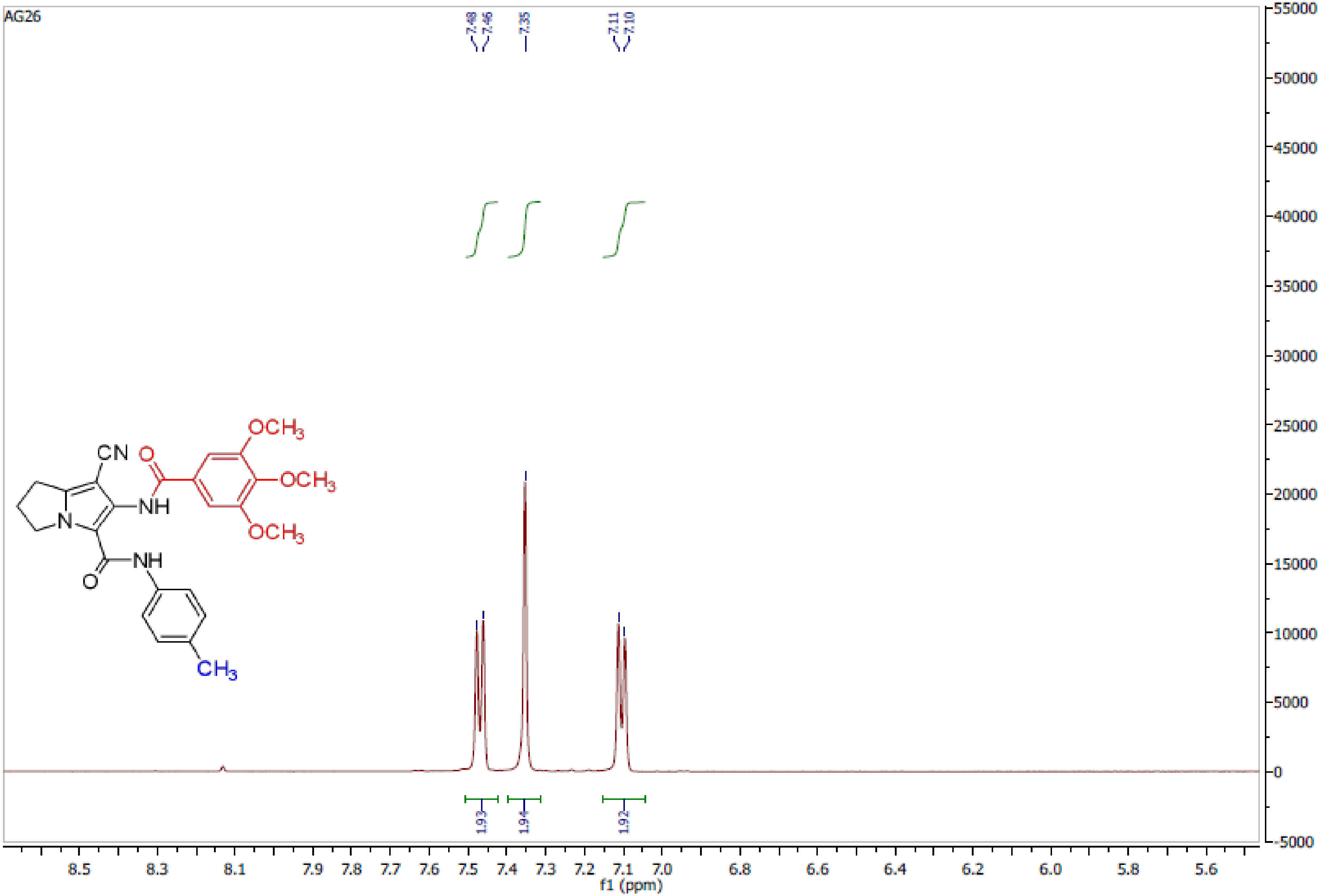

Fig. S67. ^{13}C-NMR (DMSO, 125 MHz, δ ppm) spectrum of compound **16b**

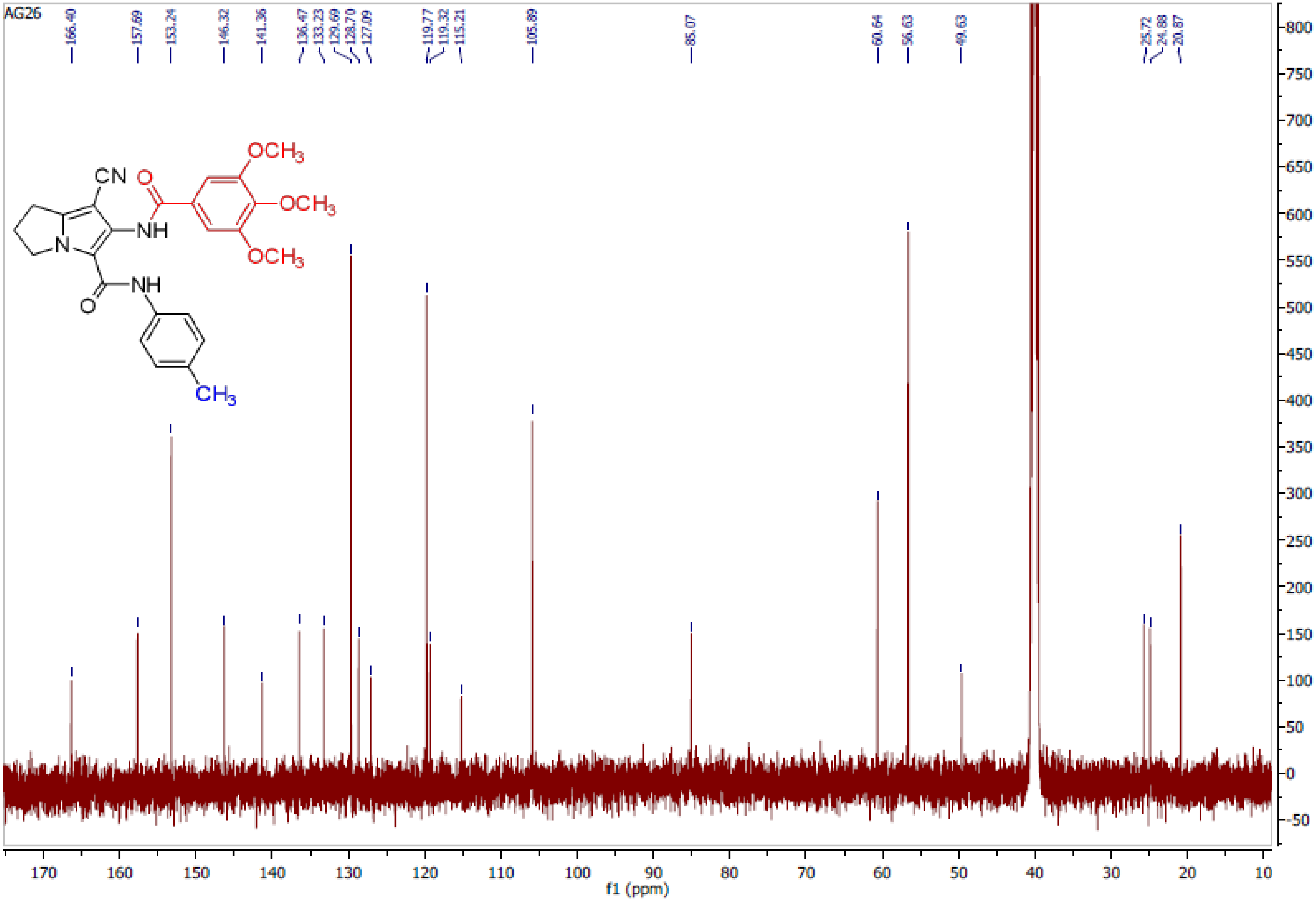

Fig. S68. ^{13}C-NMR (DMSO, 125 MHz, δ ppm) spectrum of compound **16b** (**ZOOM on aliphatic Cs**)

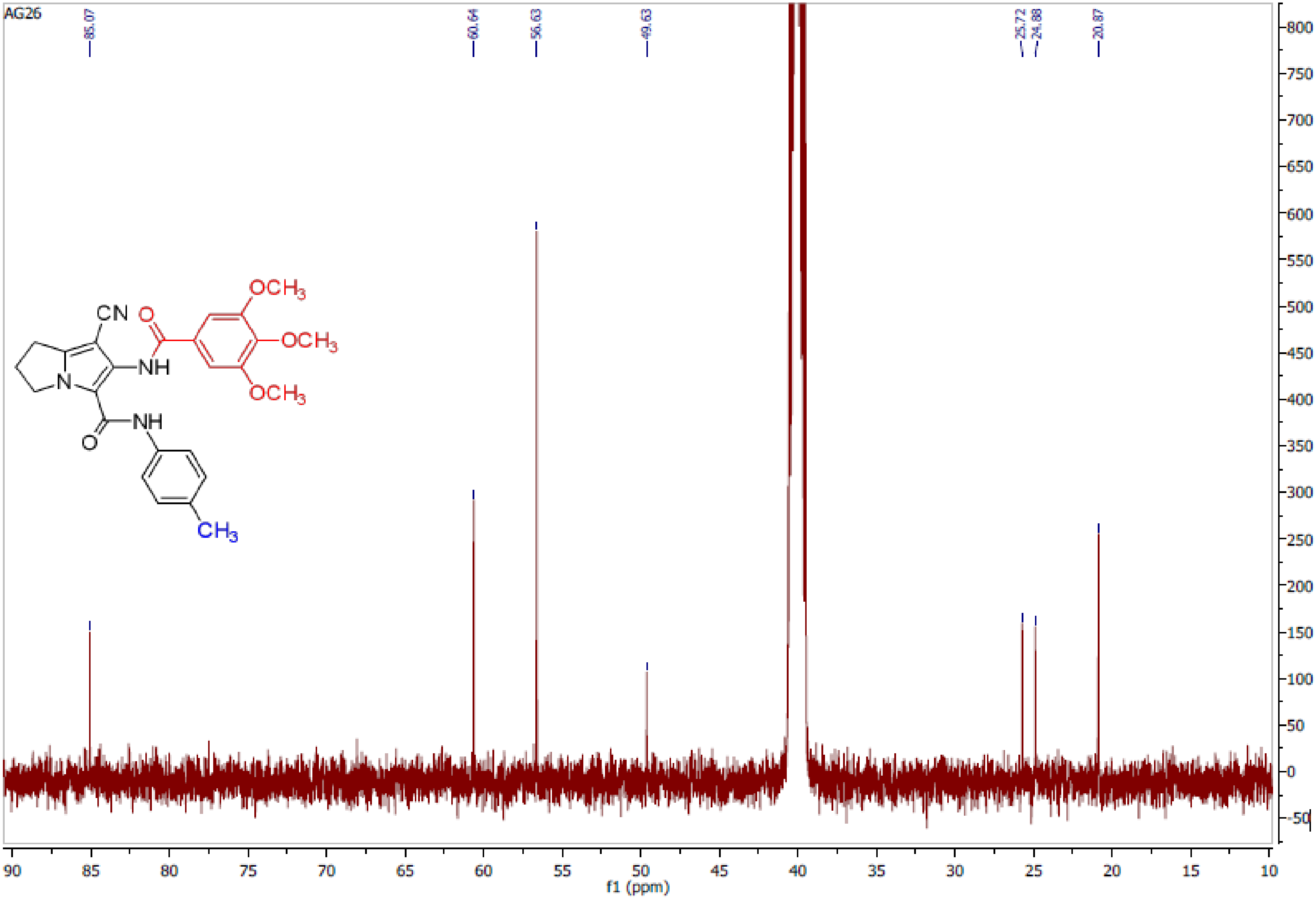

Fig. S69. ^{13}C-NMR (DMSO, 125 MHz, δ ppm) spectrum of compound **16b** (ZOOM on aromatic Cs)

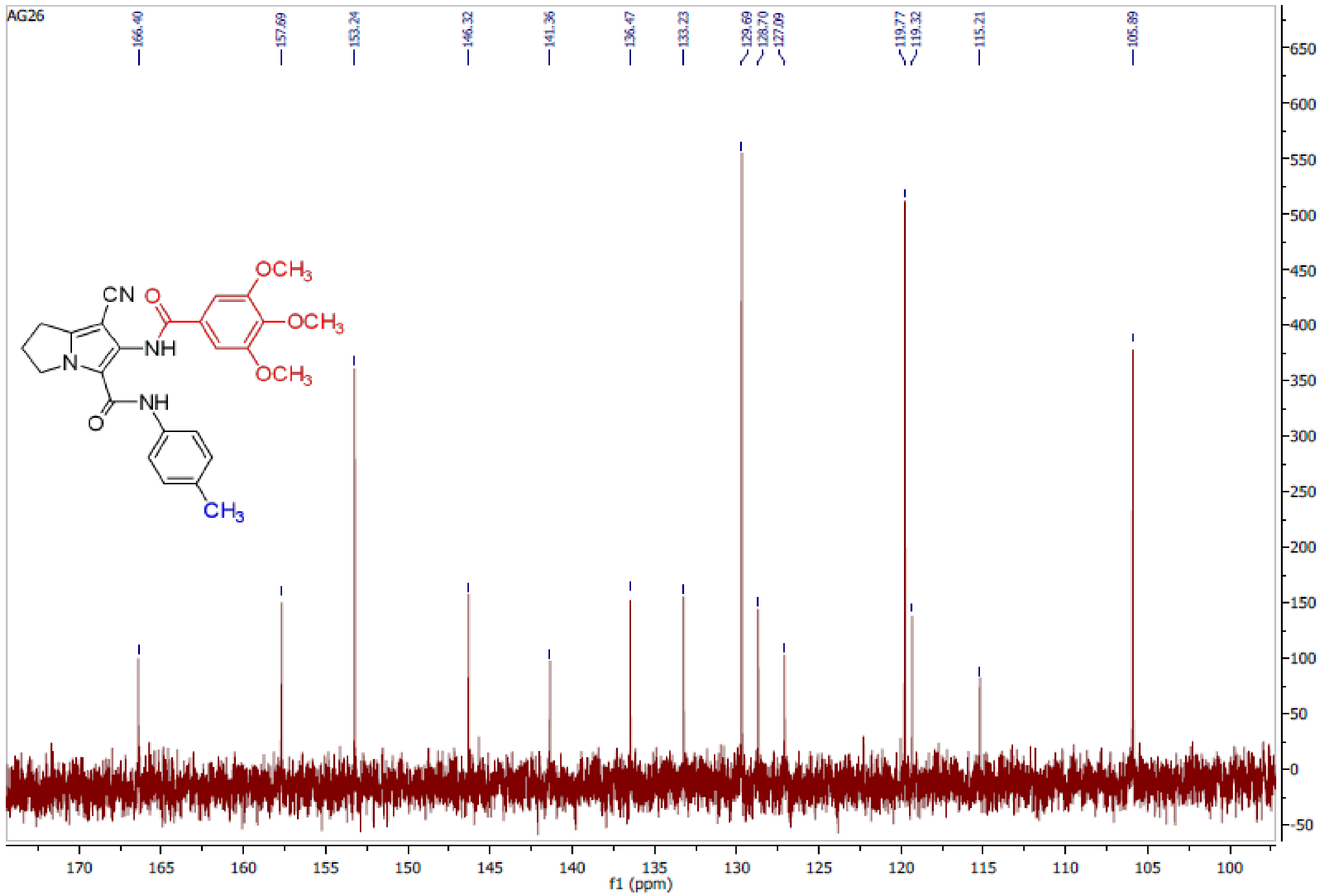

Fig. S70. **DEPT C[135] spectrum of compound 16b**

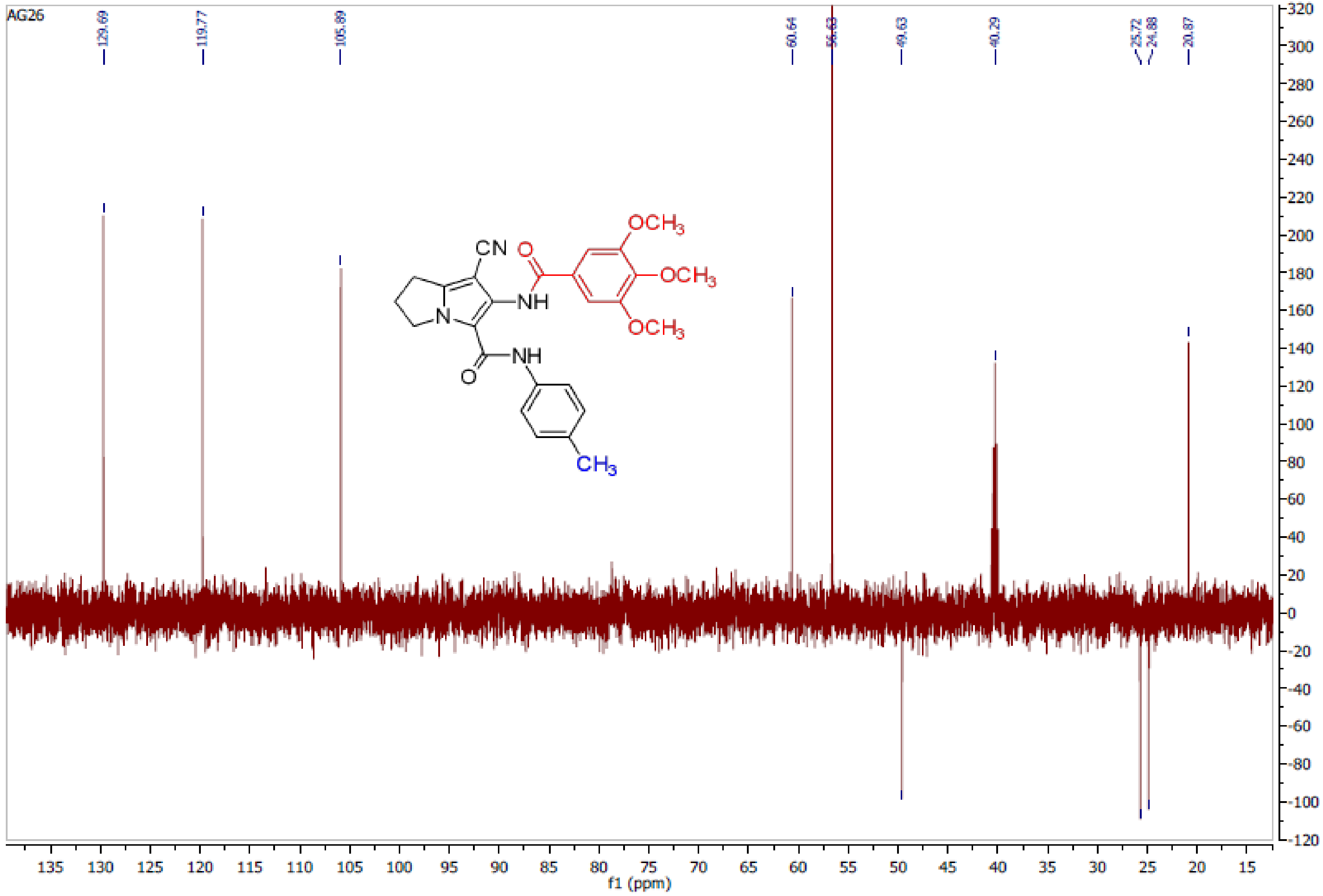

Fig. S71. ¹H-NMR (DMSO, 500 MHz, δ ppm) spectrum of compound **16c**

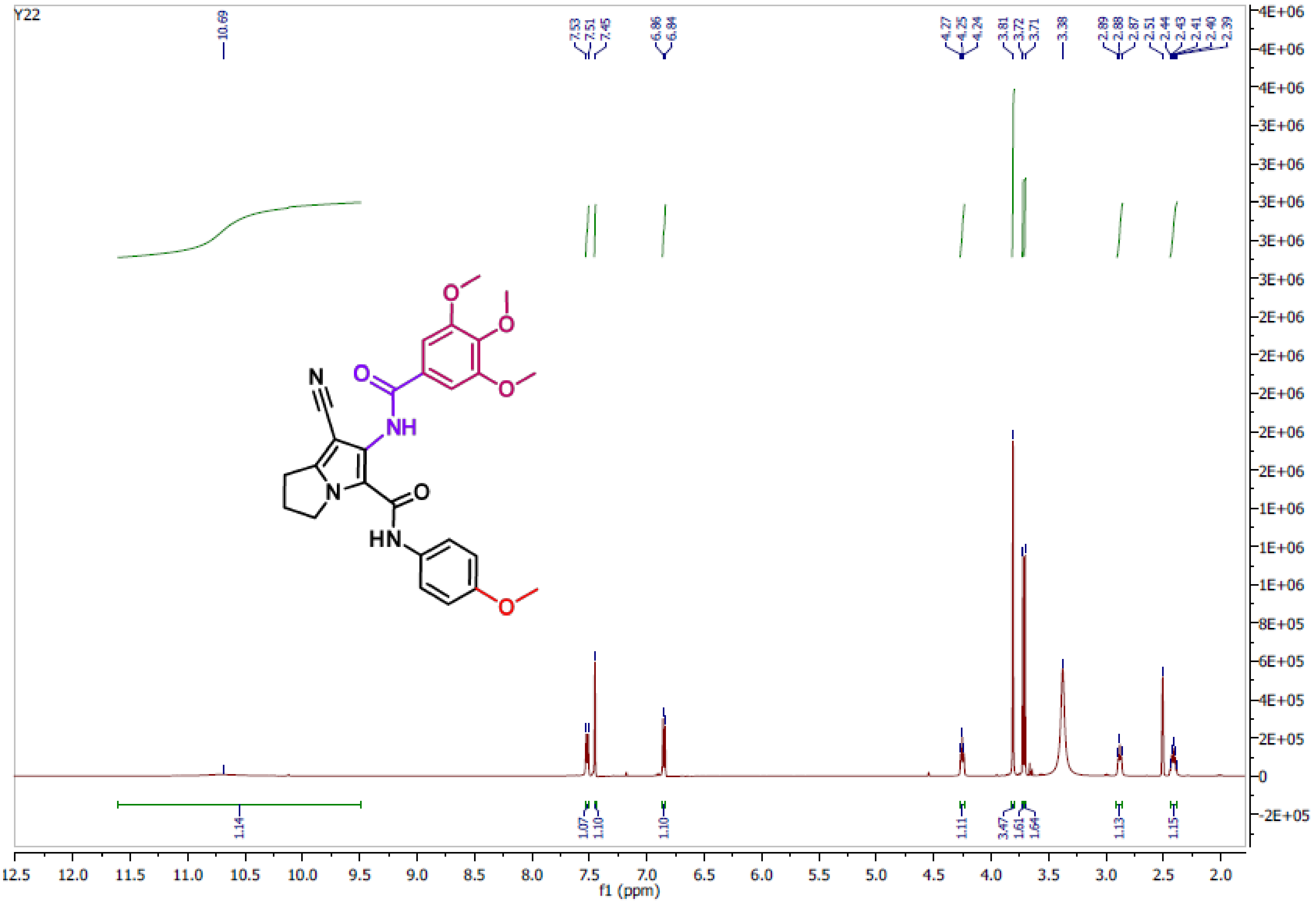

Fig. S72. ^{1}H-NMR (DMSO, 500 MHz, δ ppm) spectrum of compound **16c** (**zoom on the broad singlet**)

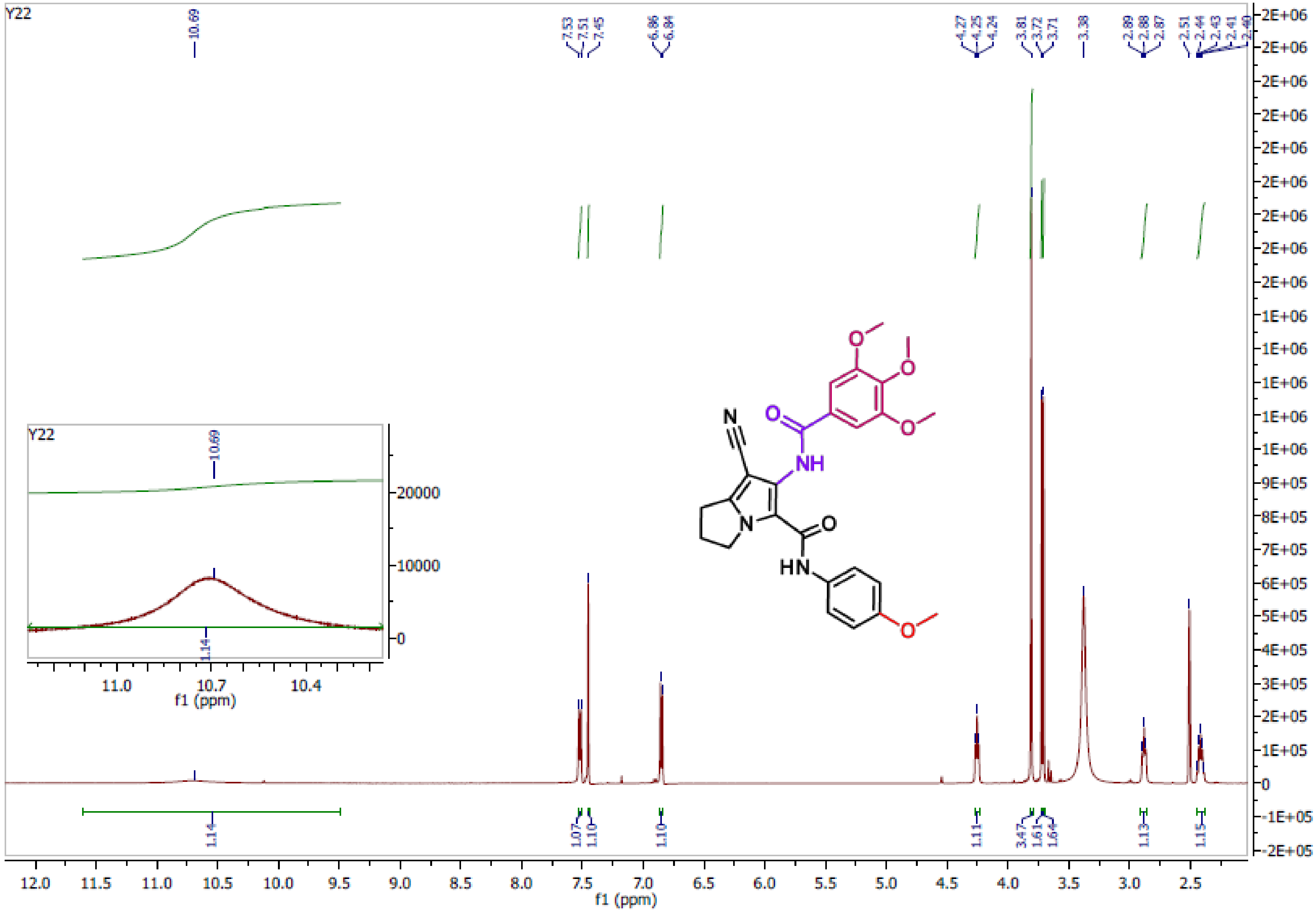

Fig. S73. ^{1}H-NMR (DMSO, 500 MHz, *δ* ppm) spectrum of compound **16c** (**zoom, aliphatic Hs**)

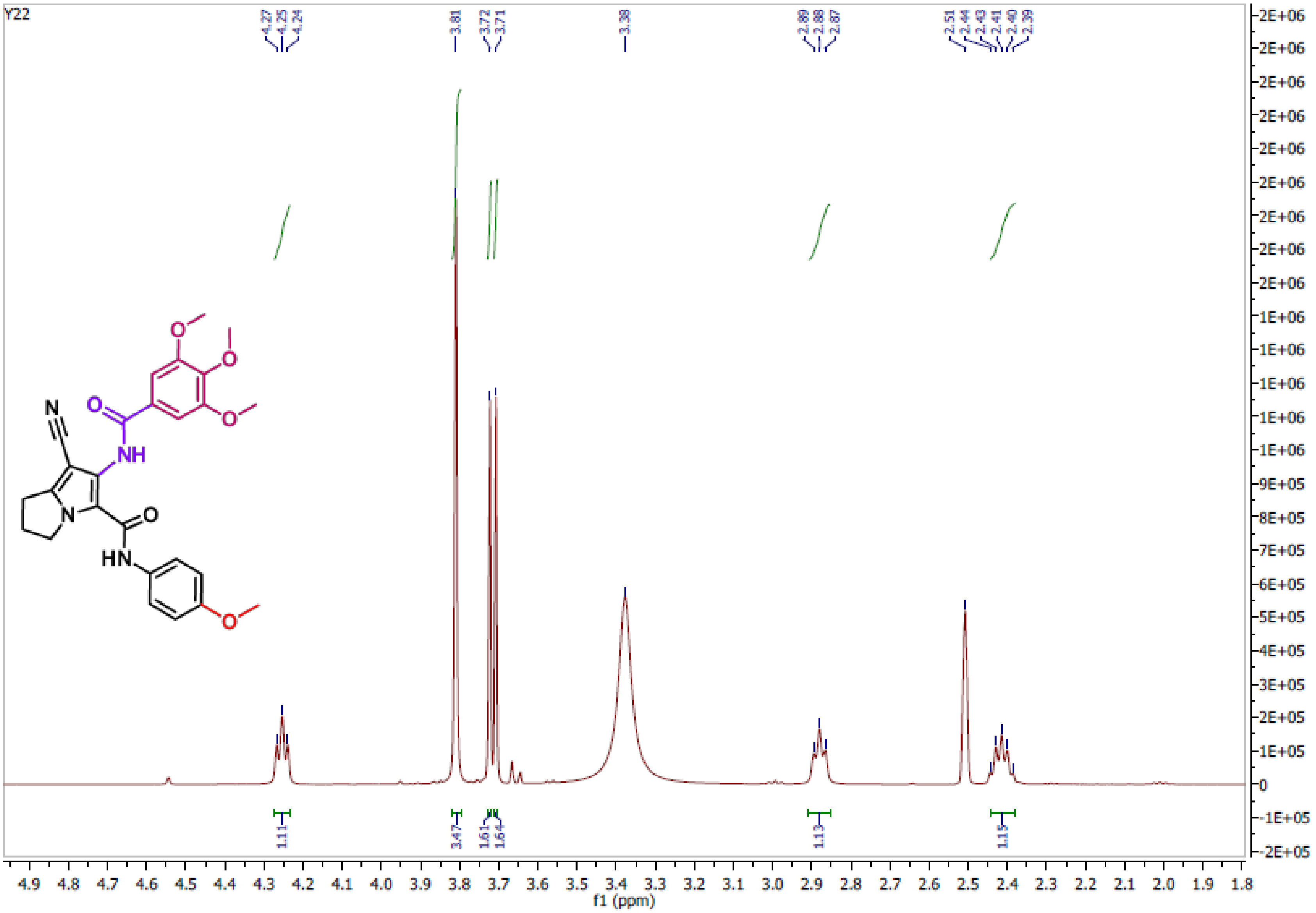

Fig. S74. ^{1}H-NMR (DMSO, 500 MHz, *δ* ppm) spectrum of compound 16c (zoom OCH3 groups)

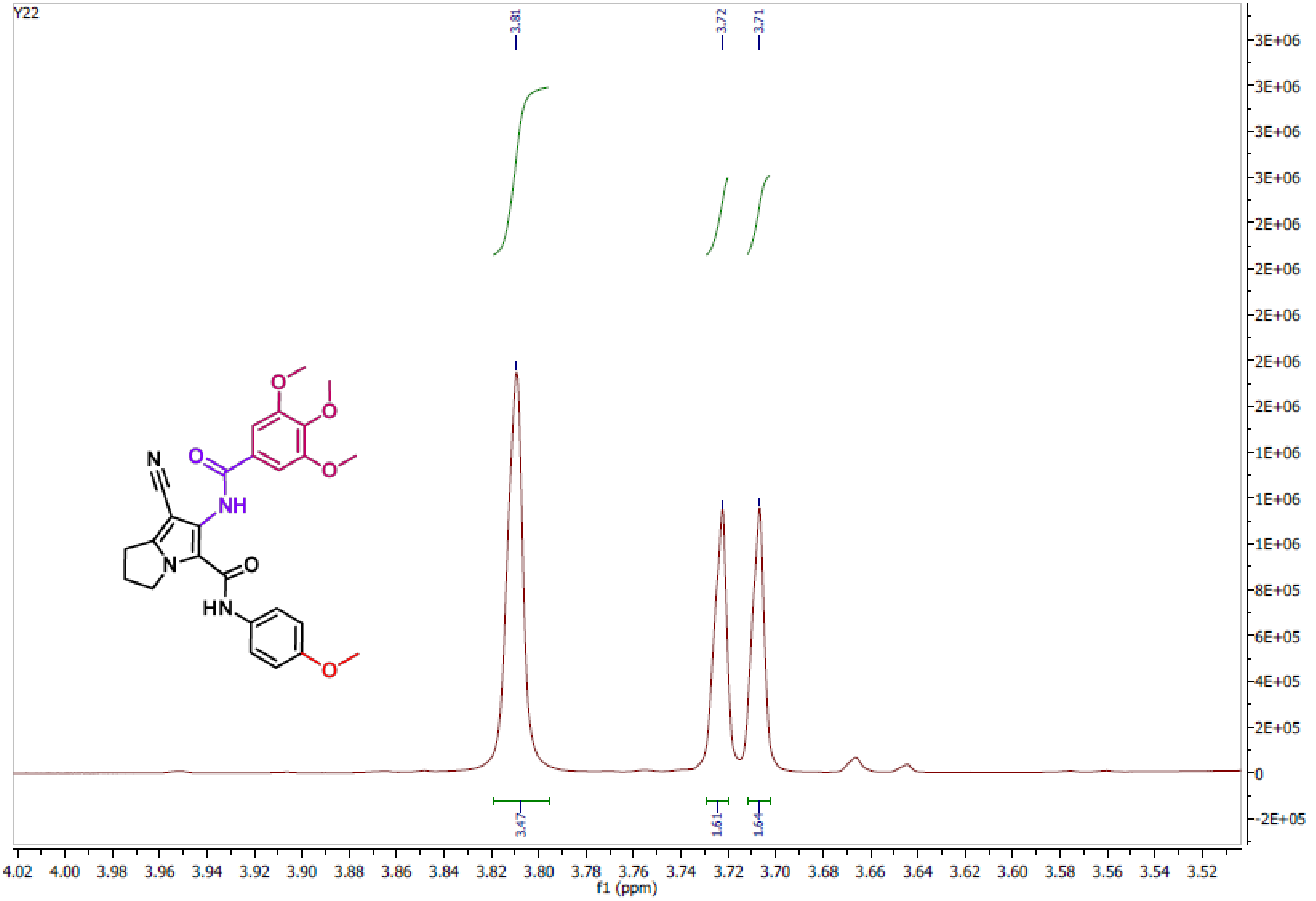

Fig. S75. ^{1}H-NMR (DMSO, 500 MHz, δ ppm) spectrum of compound **16c** (zoom, aromatic Hs & NHs)

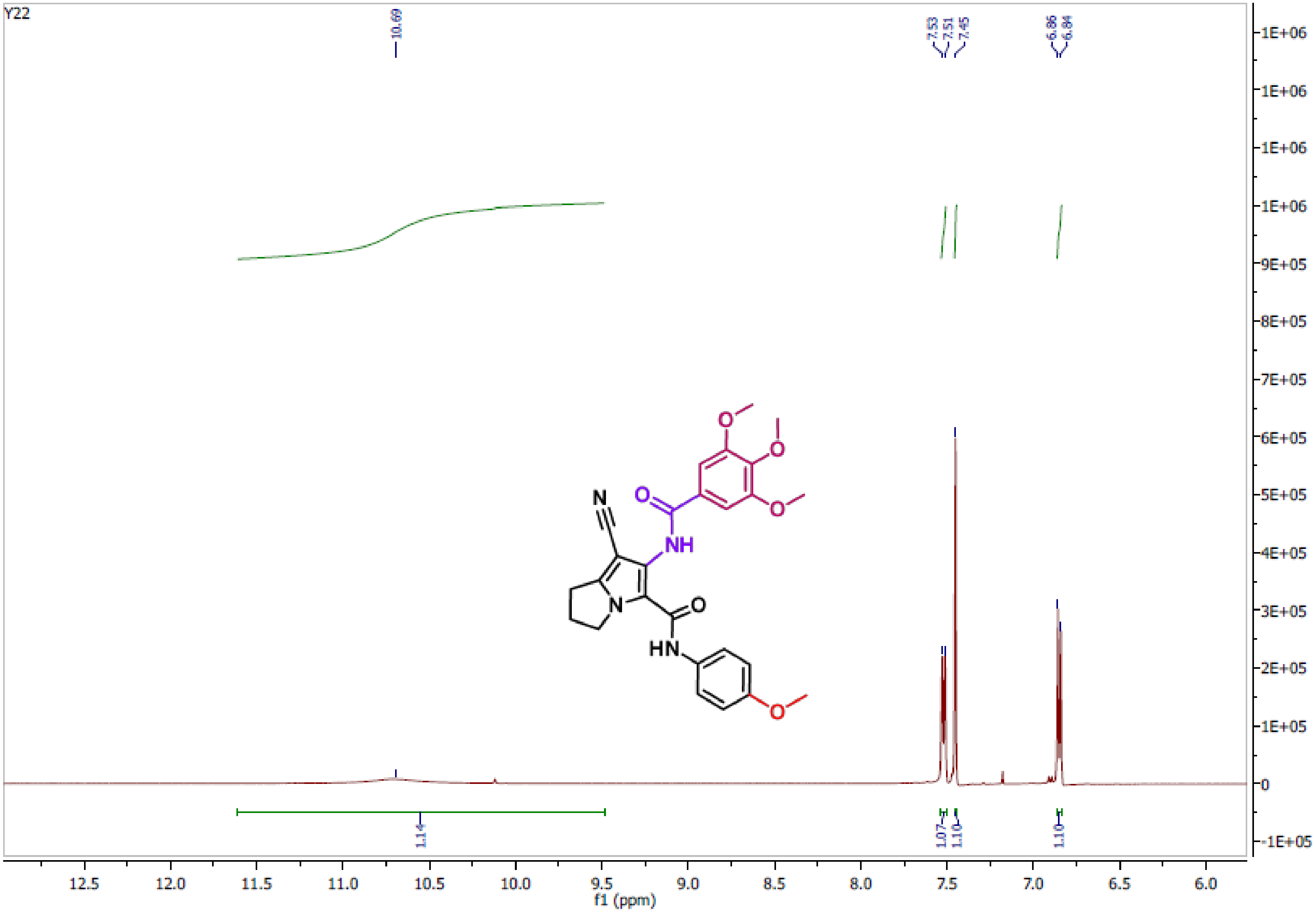

Fig. S76. ^{1}H-NMR (DMSO, 500 MHz, *δ* ppm) spectrum of compound **16c** (zoom, aromatic Hs & NHs)

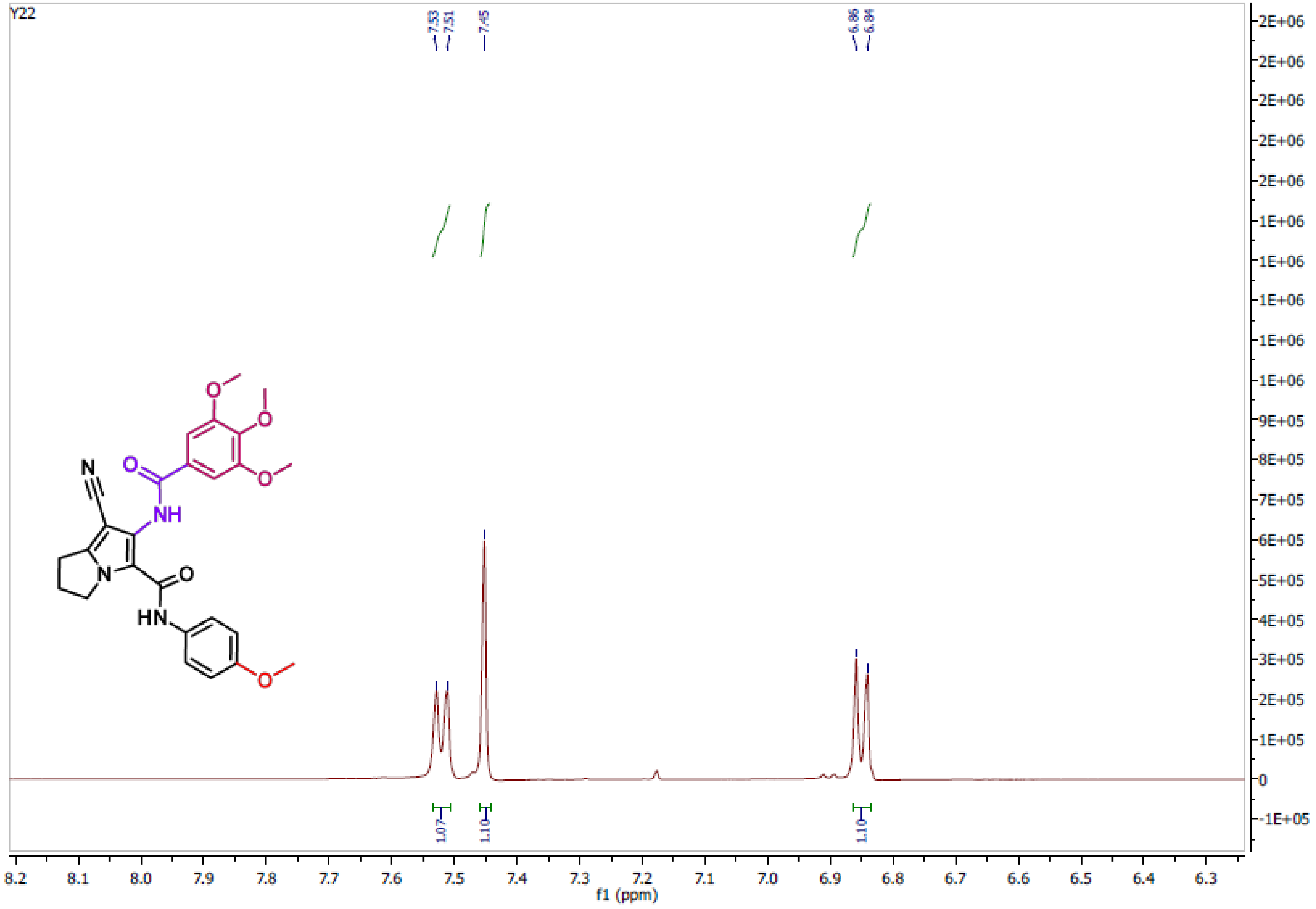

Fig. S77. ^{13}C-NMR (DMSO, 125 MHz, δ ppm) spectrum of compound **16c**

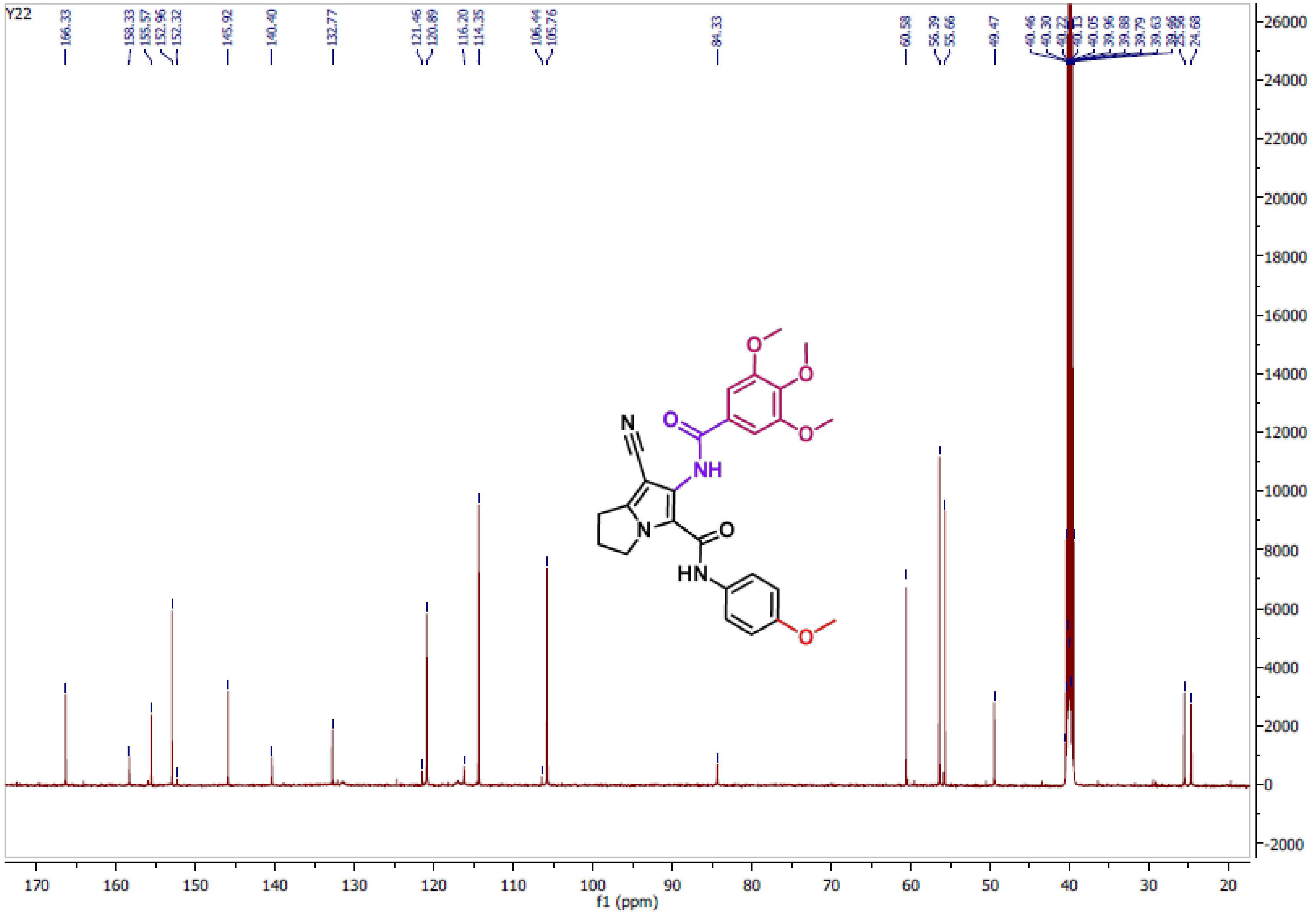

Fig. S78. ^{13}C-NMR (DMSO, 125 MHz, δ ppm) spectrum of compound **16c** (**zoom, aliphatic Cs**)

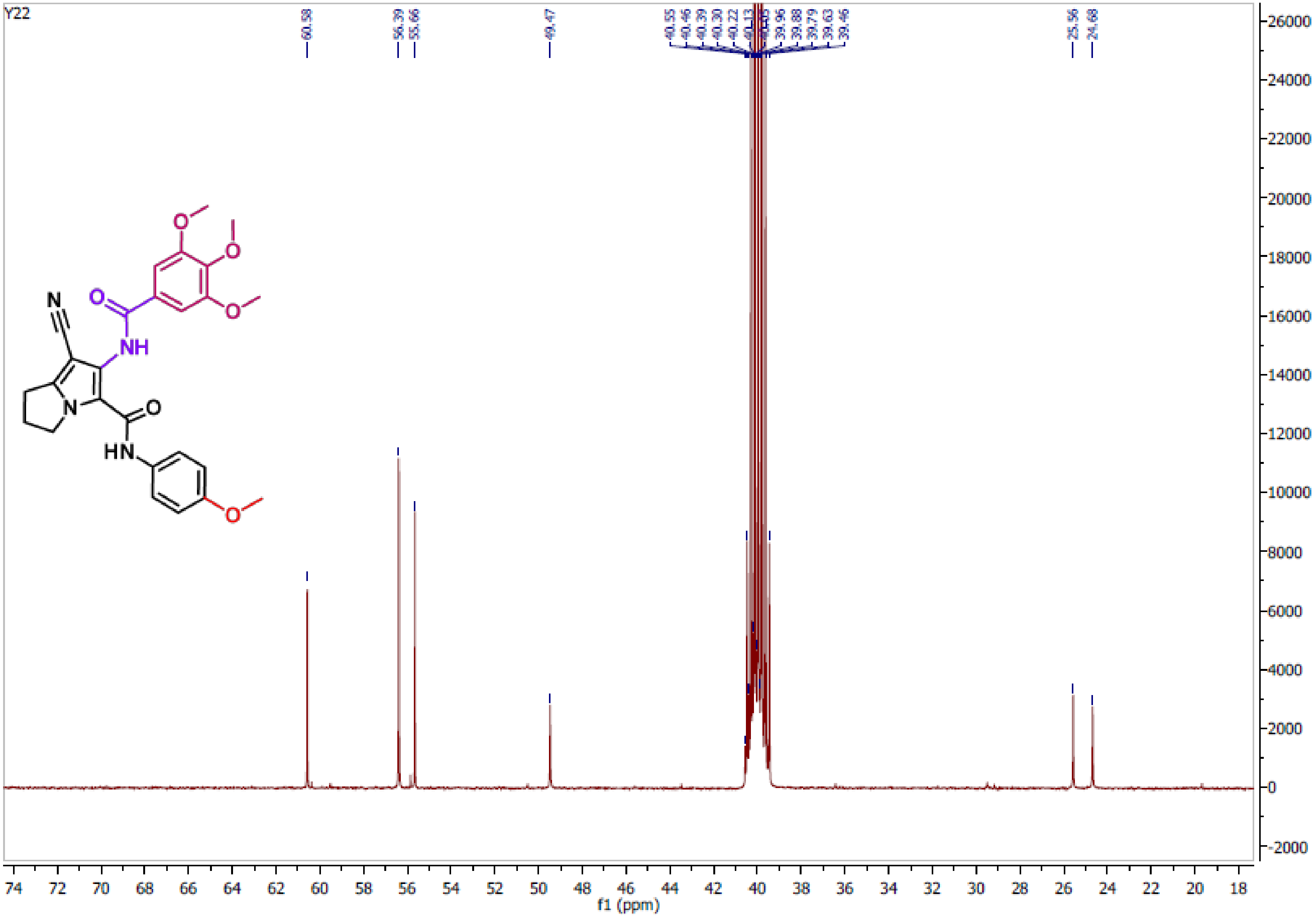

Fig. S79. ^{13}C-NMR (DMSO, 125 MHz, δ ppm) spectrum of compound **16c** (zoom, aromatic Cs)

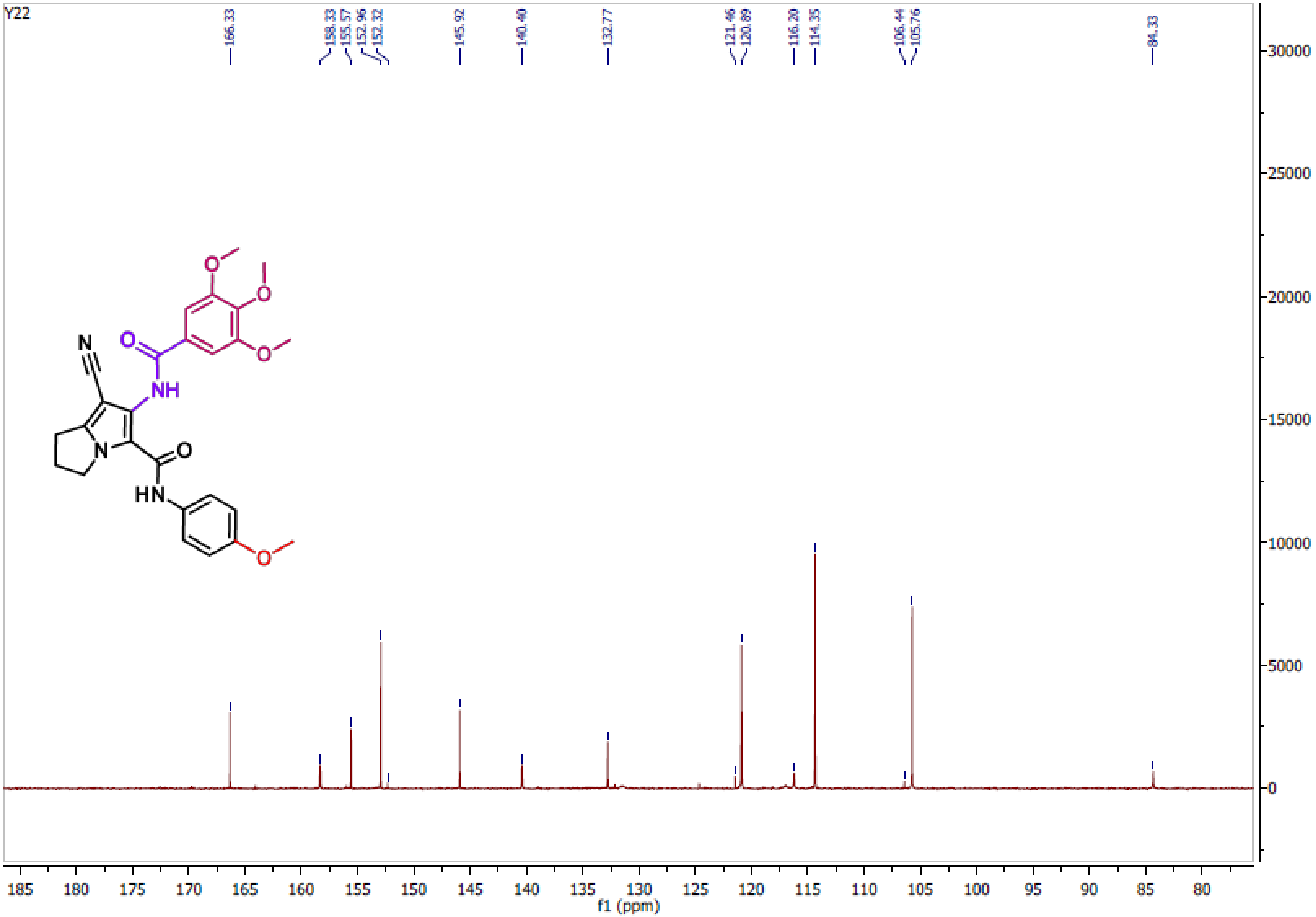

Fig. S80. **DEPT C[135] spectrum (DMSO, 125 MHz, δ ppm) of compound 16c**

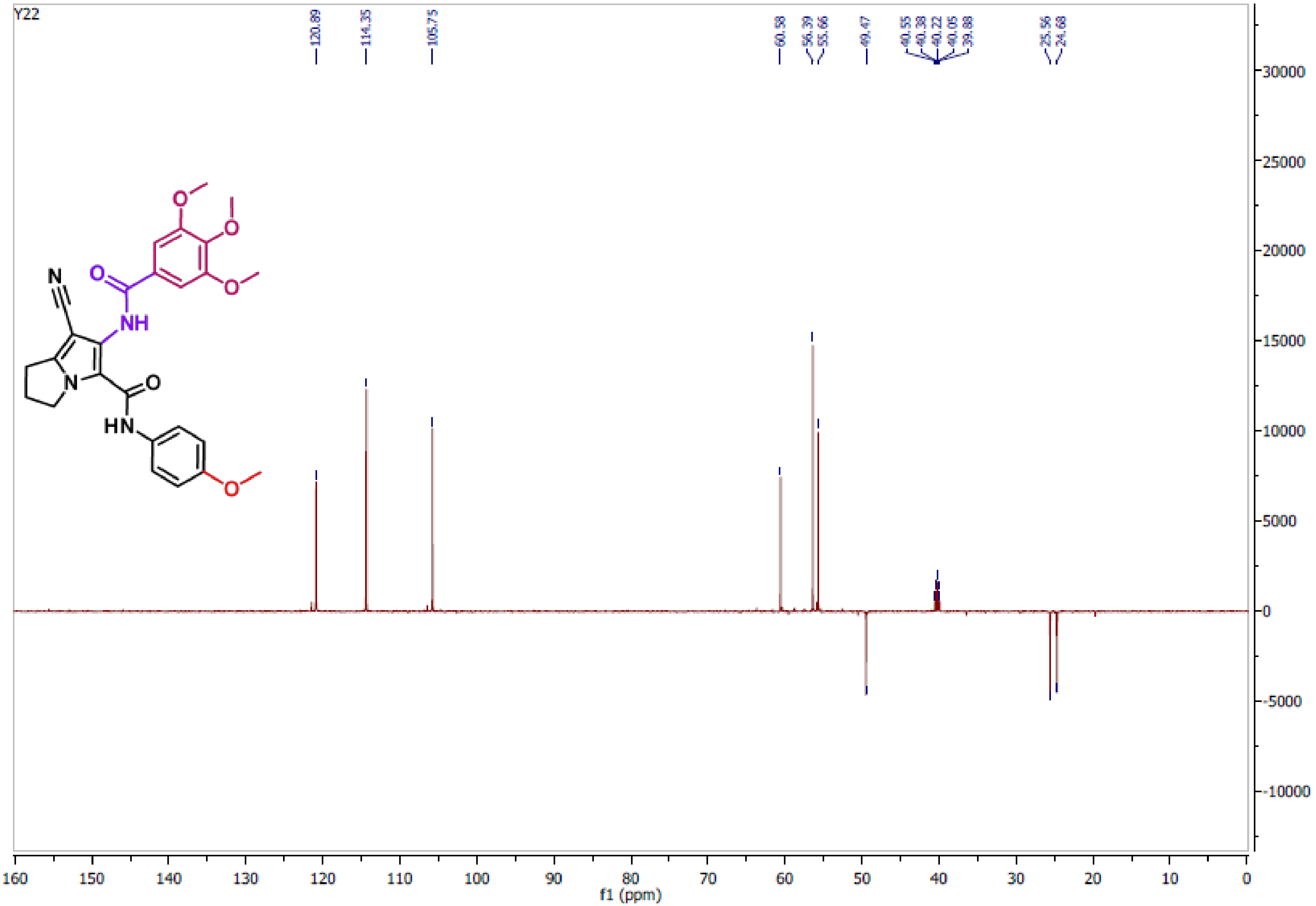

Fig. S81. ^{1}H-NMR (DMSO, 500 MHz, *δ* ppm) spectrum of compound **16d**

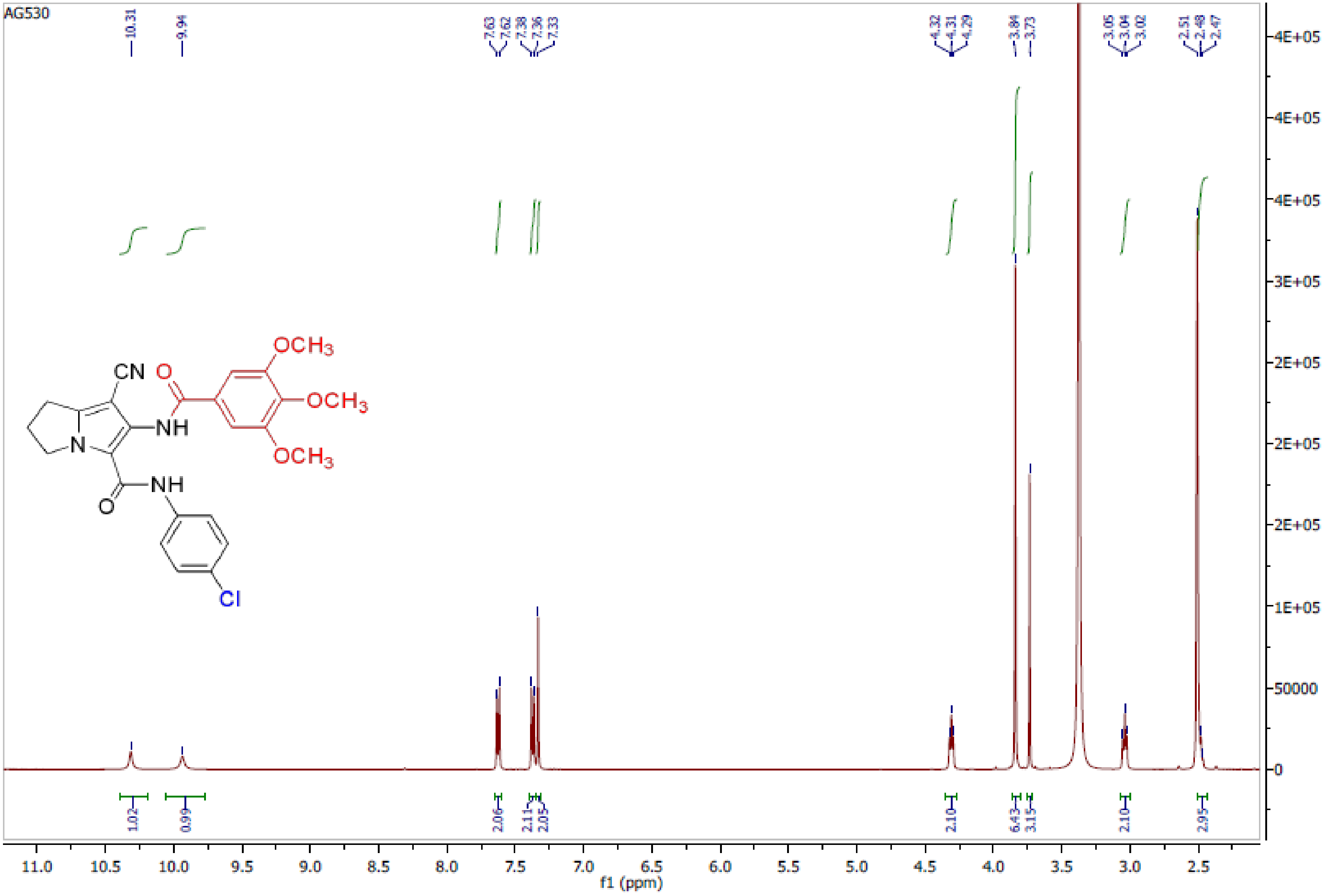

Fig. S82. ^{1}H-NMR (DMSO, 500 MHz, δ ppm) spectrum of compound **16d** (**ZOOM on aliphatic Hs**)

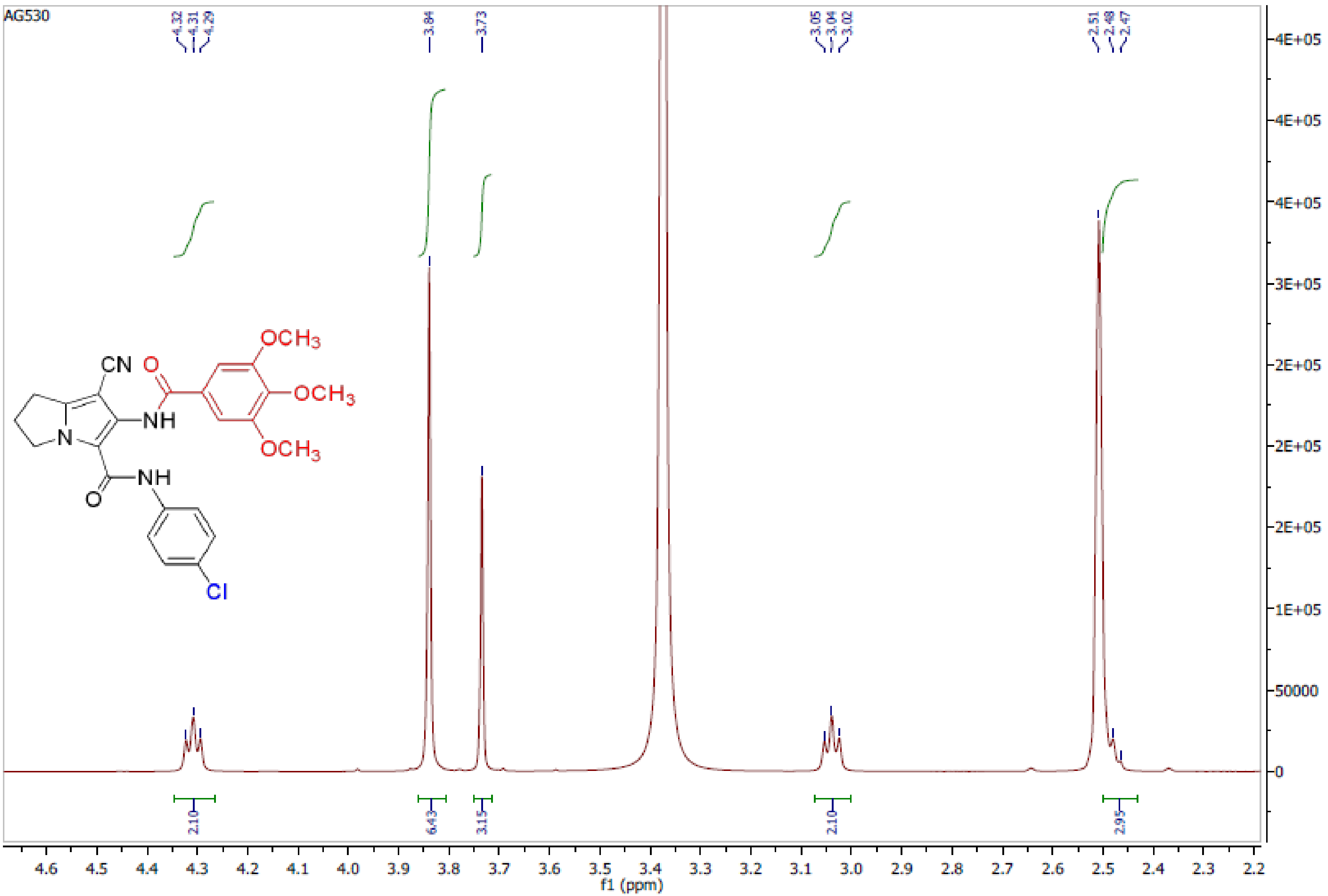

Fig. S83. ^{1}H-NMR (DMSO, 500 MHz, δ ppm) spectrum of compound **16d** (**ZOOM on NHs & aromatic Hs**)

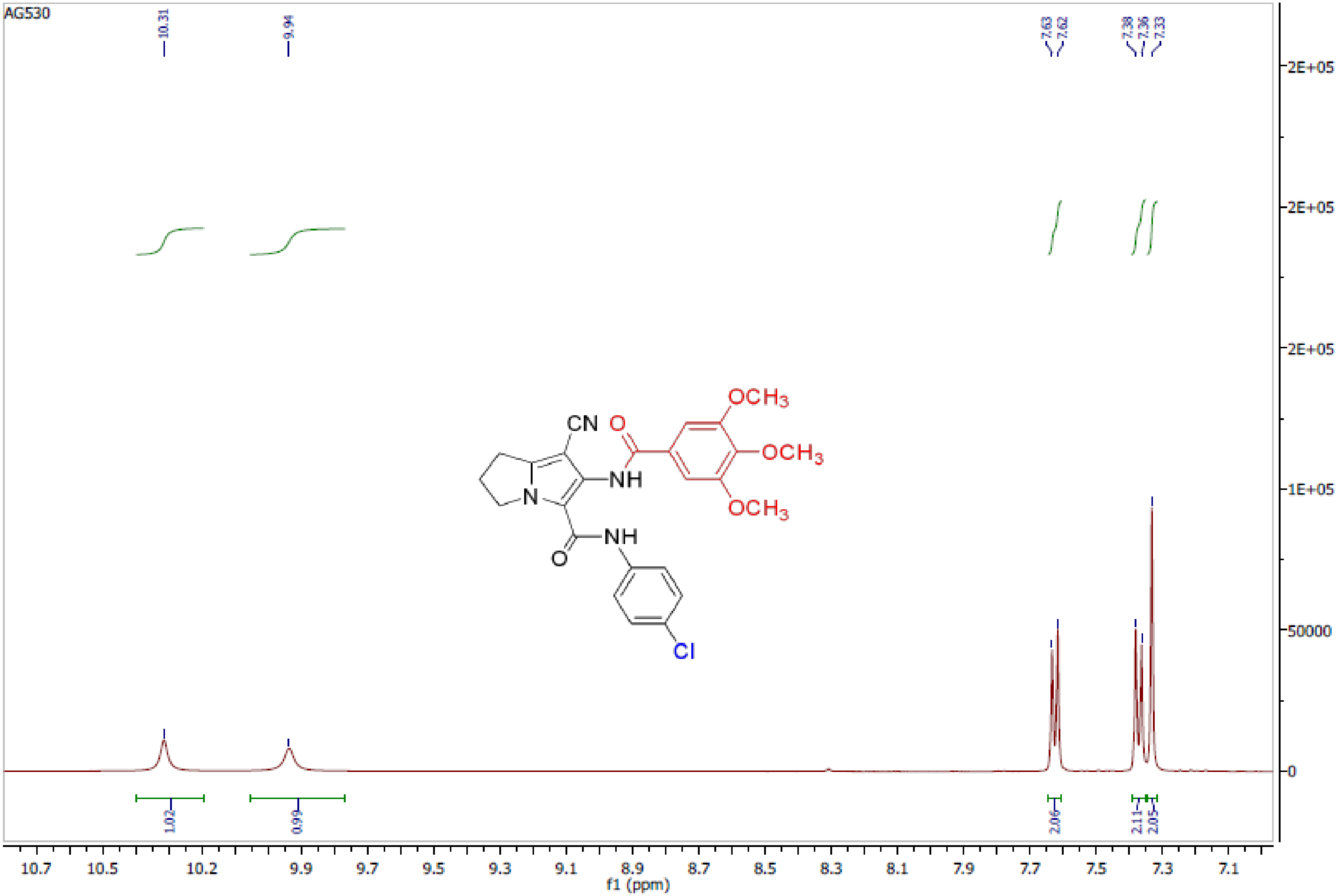

Fig. S84. ^{1}H-NMR (DMSO, 500 MHz, δ ppm) spectrum of compound **16d** (**ZOOM on aromatic Hs**)

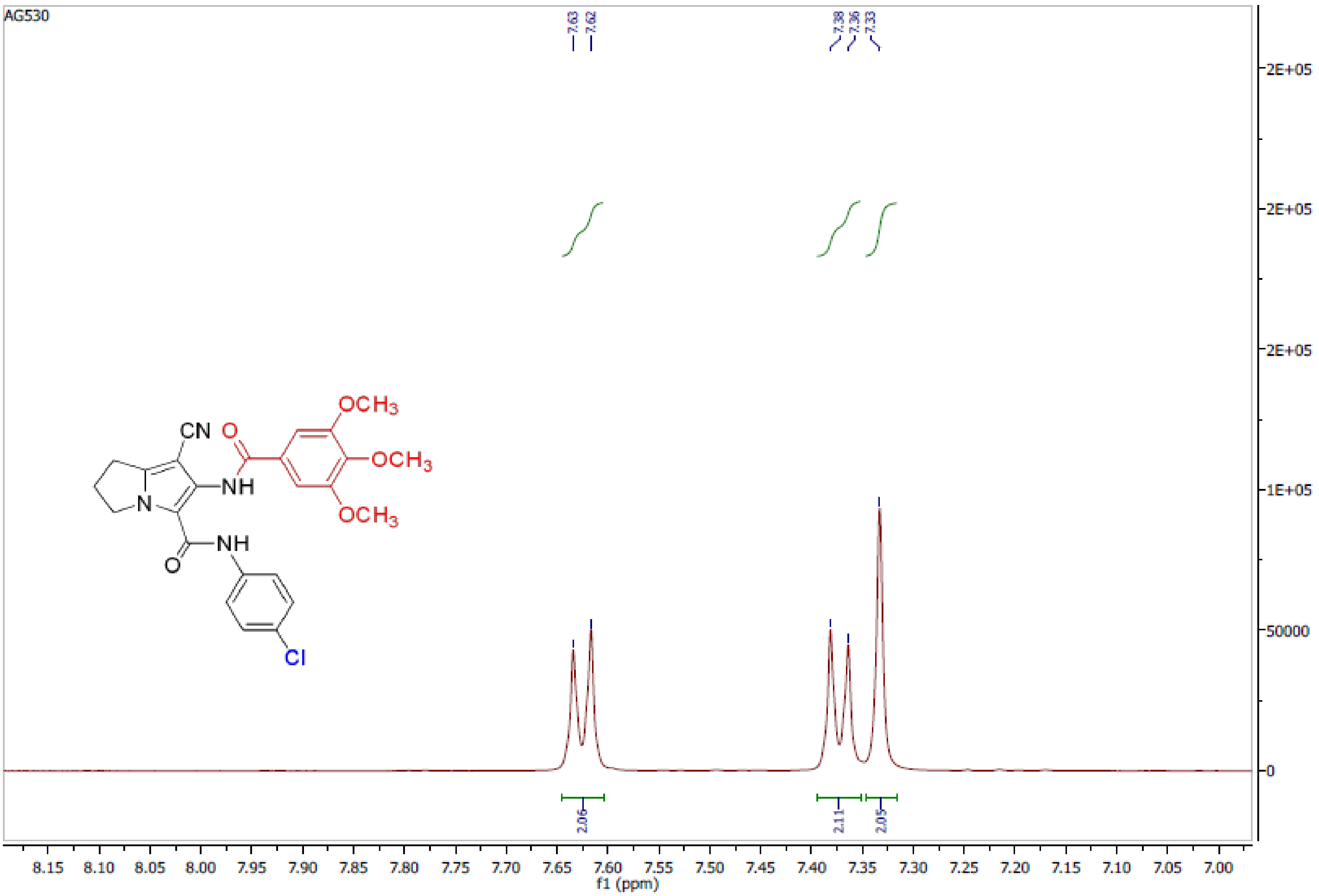

Fig. S85. ^{13}C-NMR (DMSO, 125 MHz, δ ppm) spectrum of compound **16d**

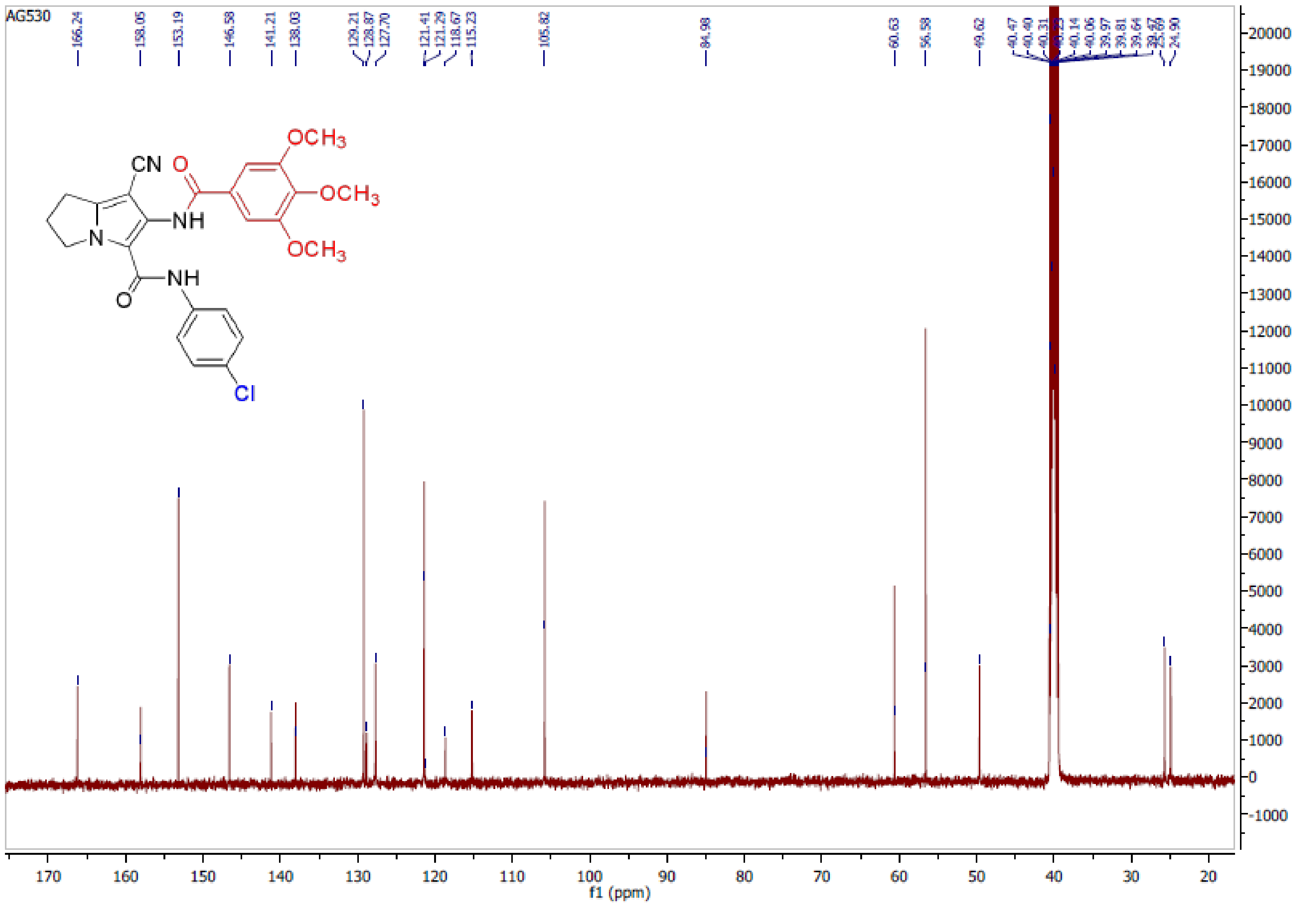

Fig. S86. ^{13}C-NMR (DMSO, 125 MHz, δ ppm) spectrum of compound 16d (ZOOM on aliphatic Cs)

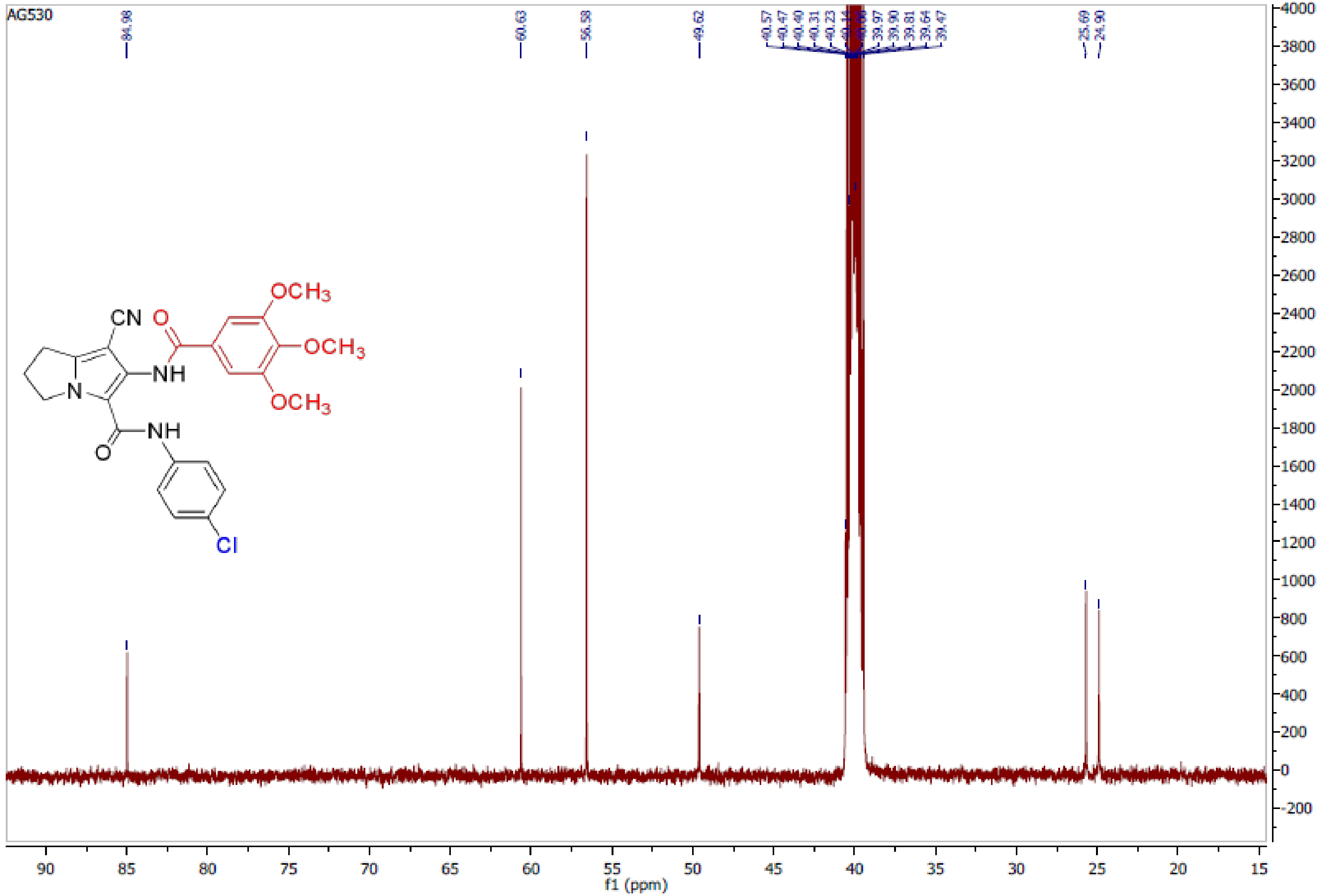

Fig. S87. ^{13}C-NMR (DMSO, 125 MHz, *δ* ppm) spectrum of compound **16d** (**ZOOM on aromatic Cs**)

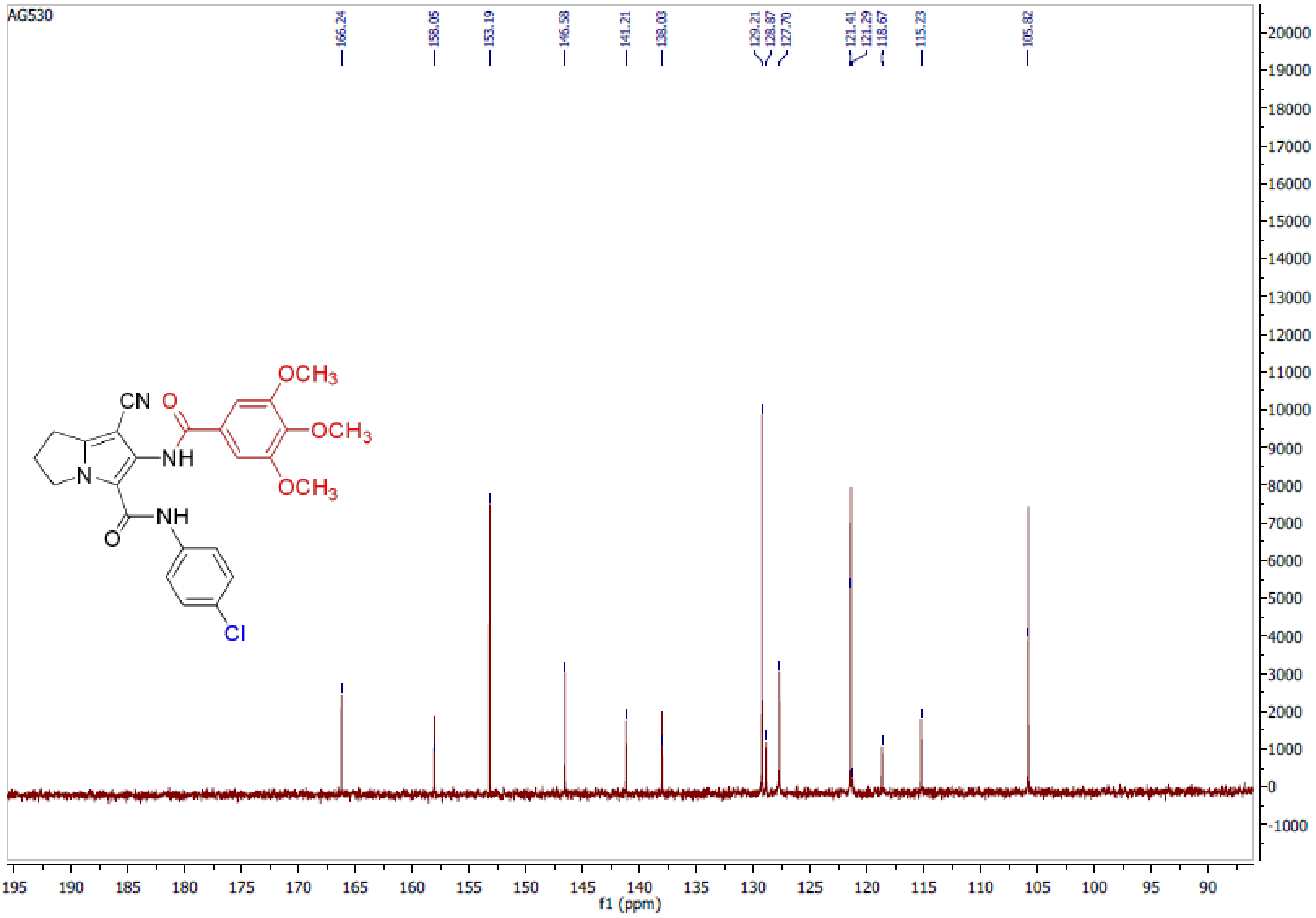

Fig. S88. **DEPT C**[135] **of compound 16d**

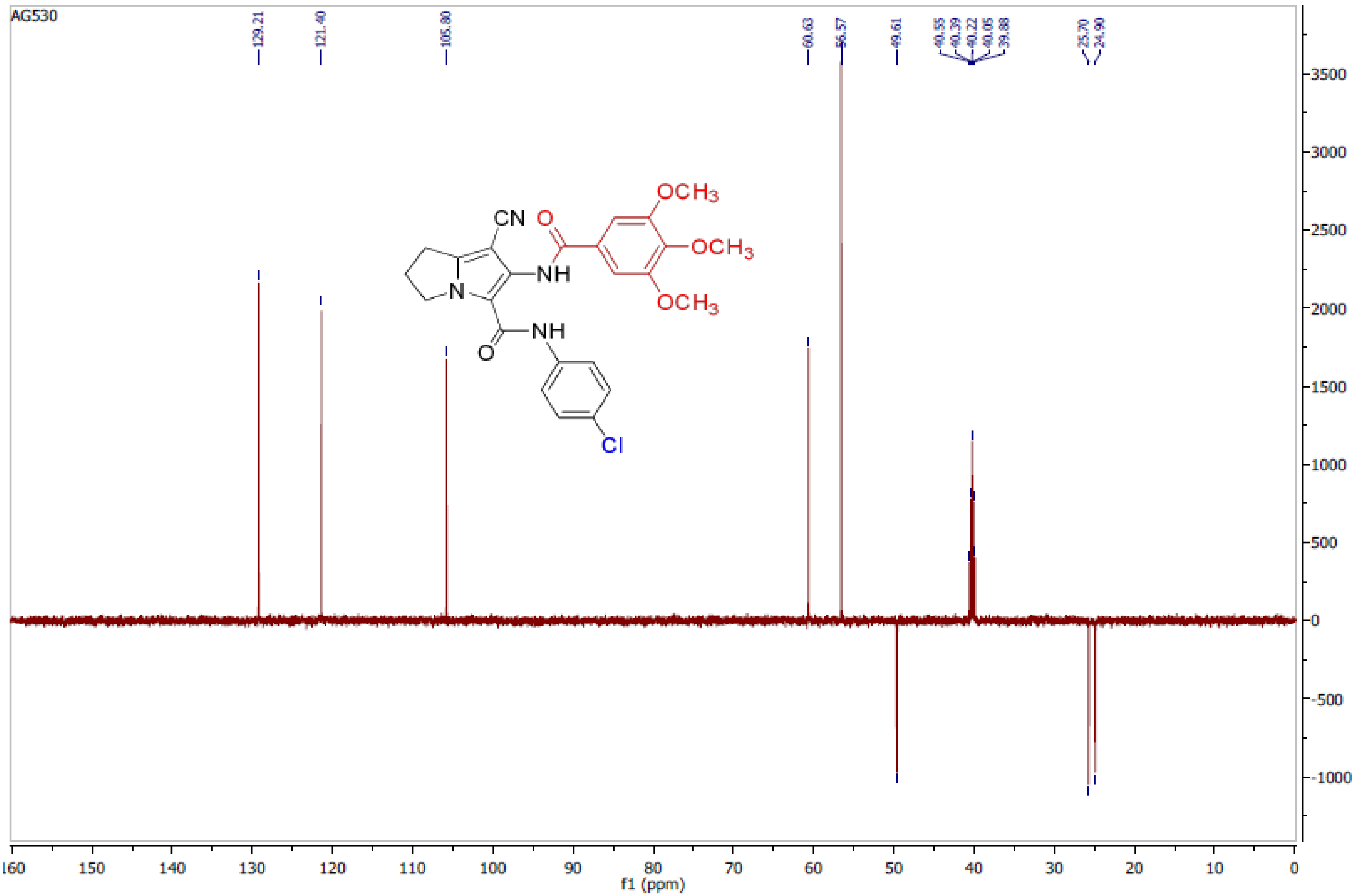

Fig. S89. ¹H-NMR (DMSO, 500 MHz, δ ppm) spectrum of compound **16e**.

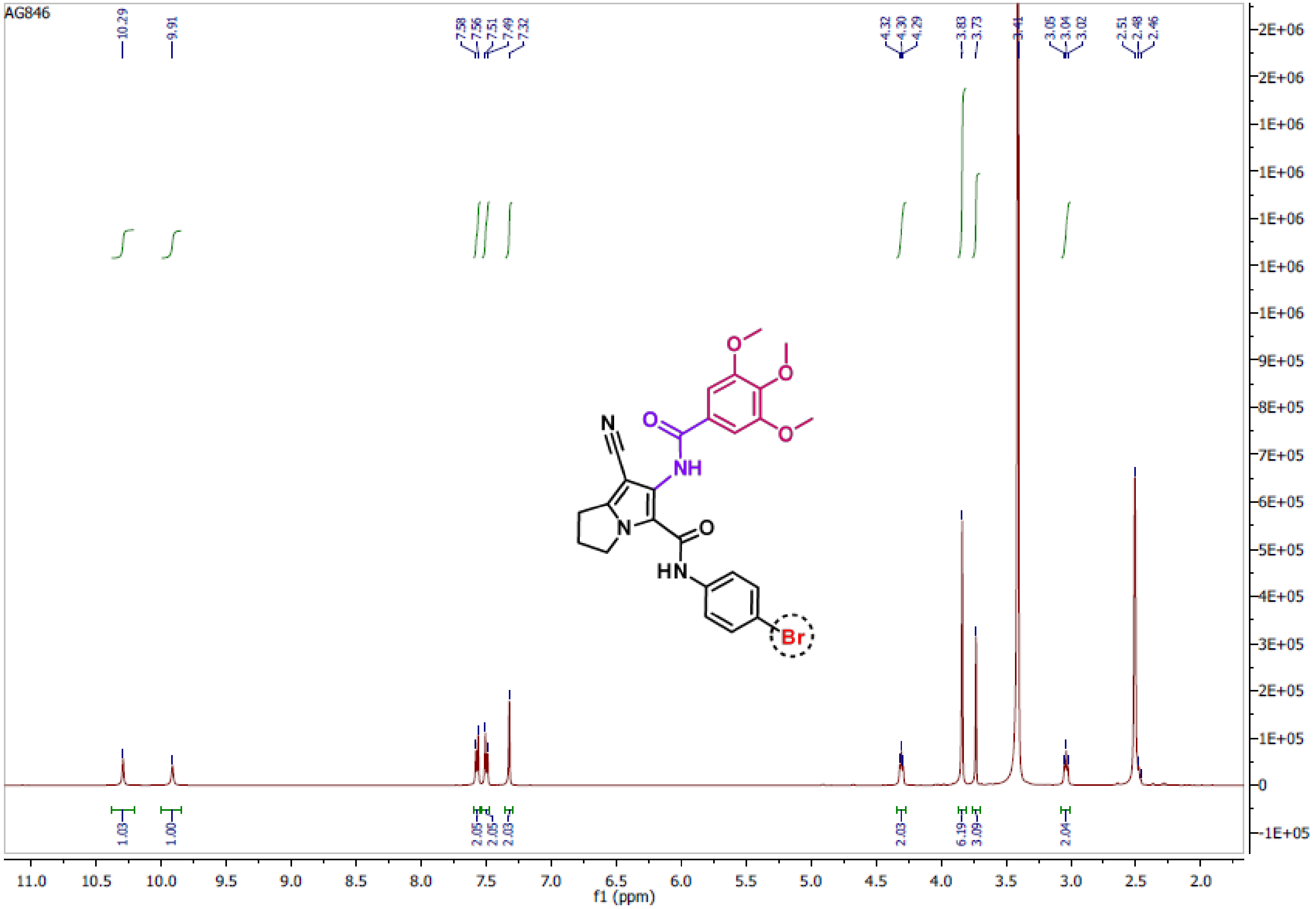

Fig. S90. ^{1}H-NMR (DMSO, 500 MHz, δ ppm) spectrum of compound **16e** (zoom, aliphatic Hs)

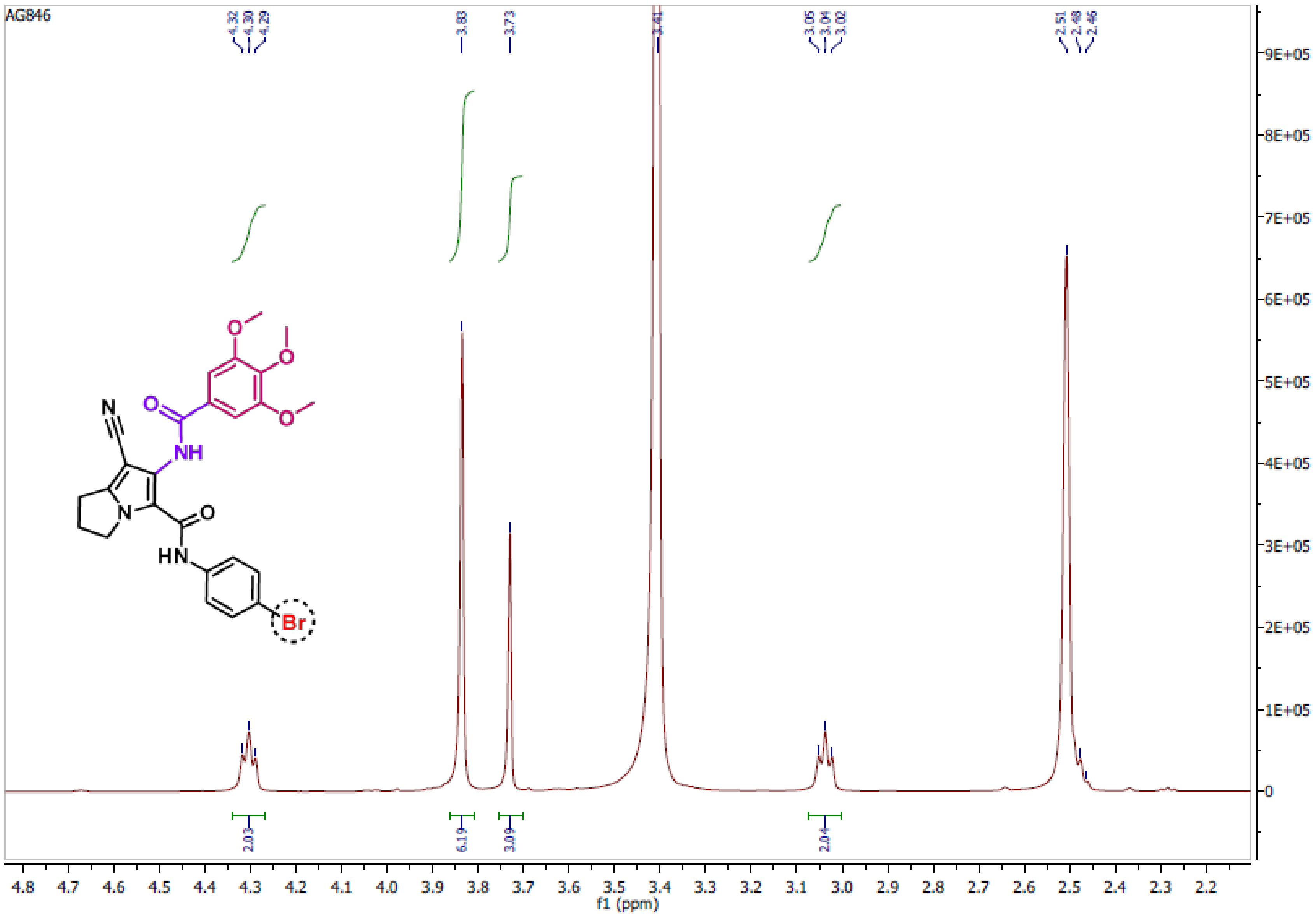

Fig. S91. ¹H-NMR (DMSO, 500 MHz, δ ppm) spectrum of compound **16e** (zoom, aromatic Hs & NHs)

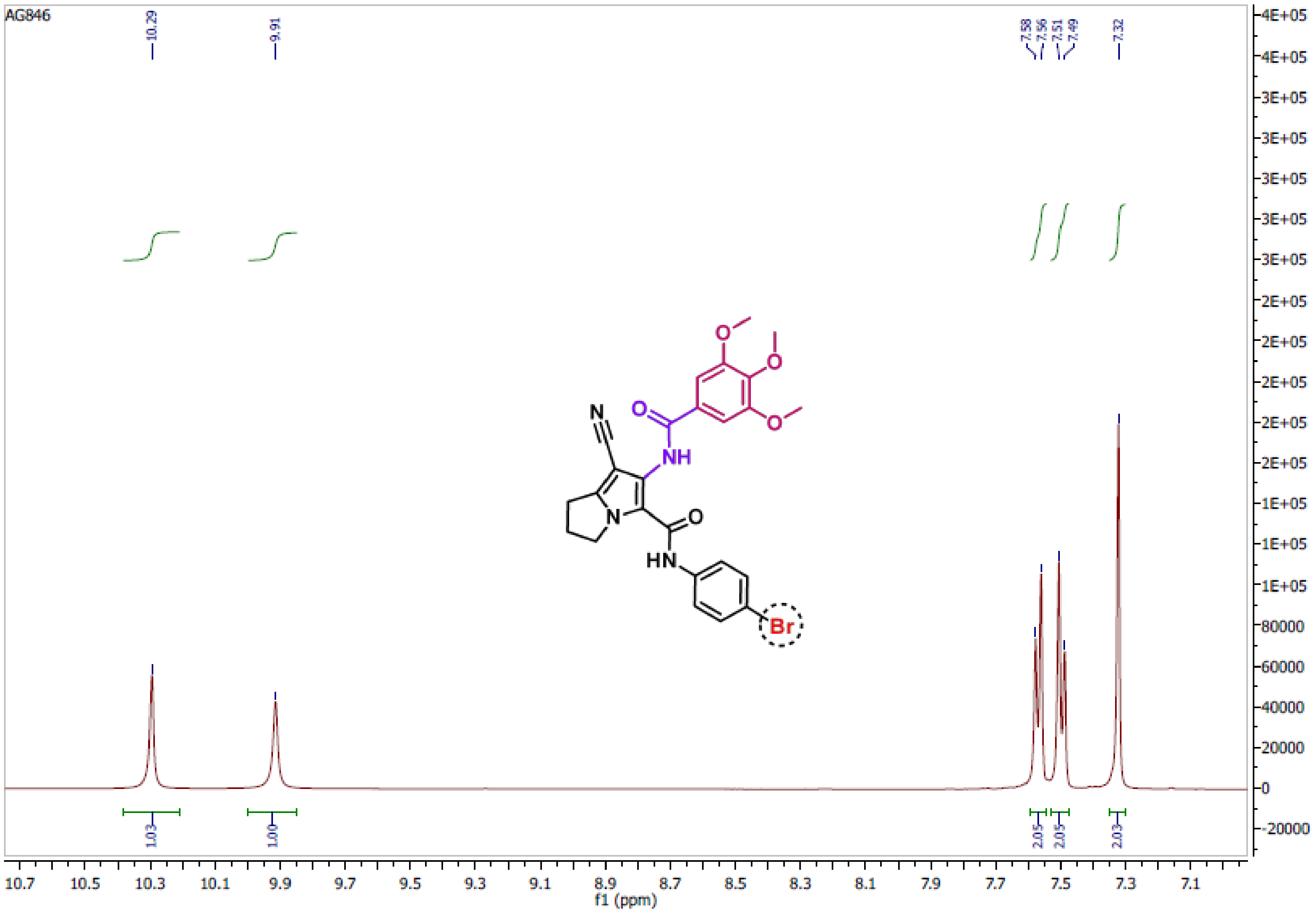

Fig. S92. ^{13}C-NMR (DMSO, 125 MHz, δ ppm) spectrum of compound **16e**

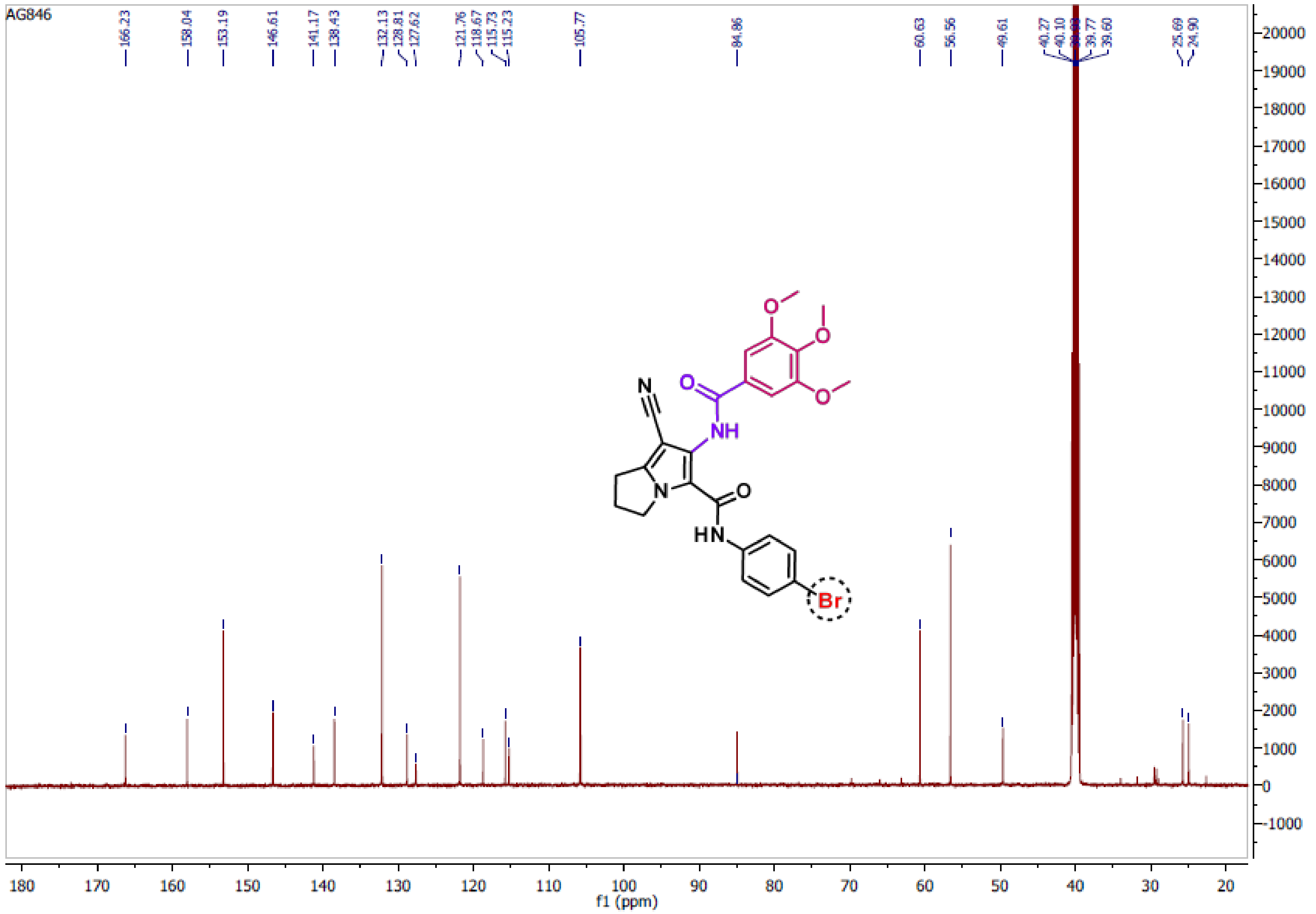

Fig. S93. ^{13}C-NMR (DMSO, 125 MHz, δ ppm) spectrum of compound **16e** (zoom, aliphatic Cs)

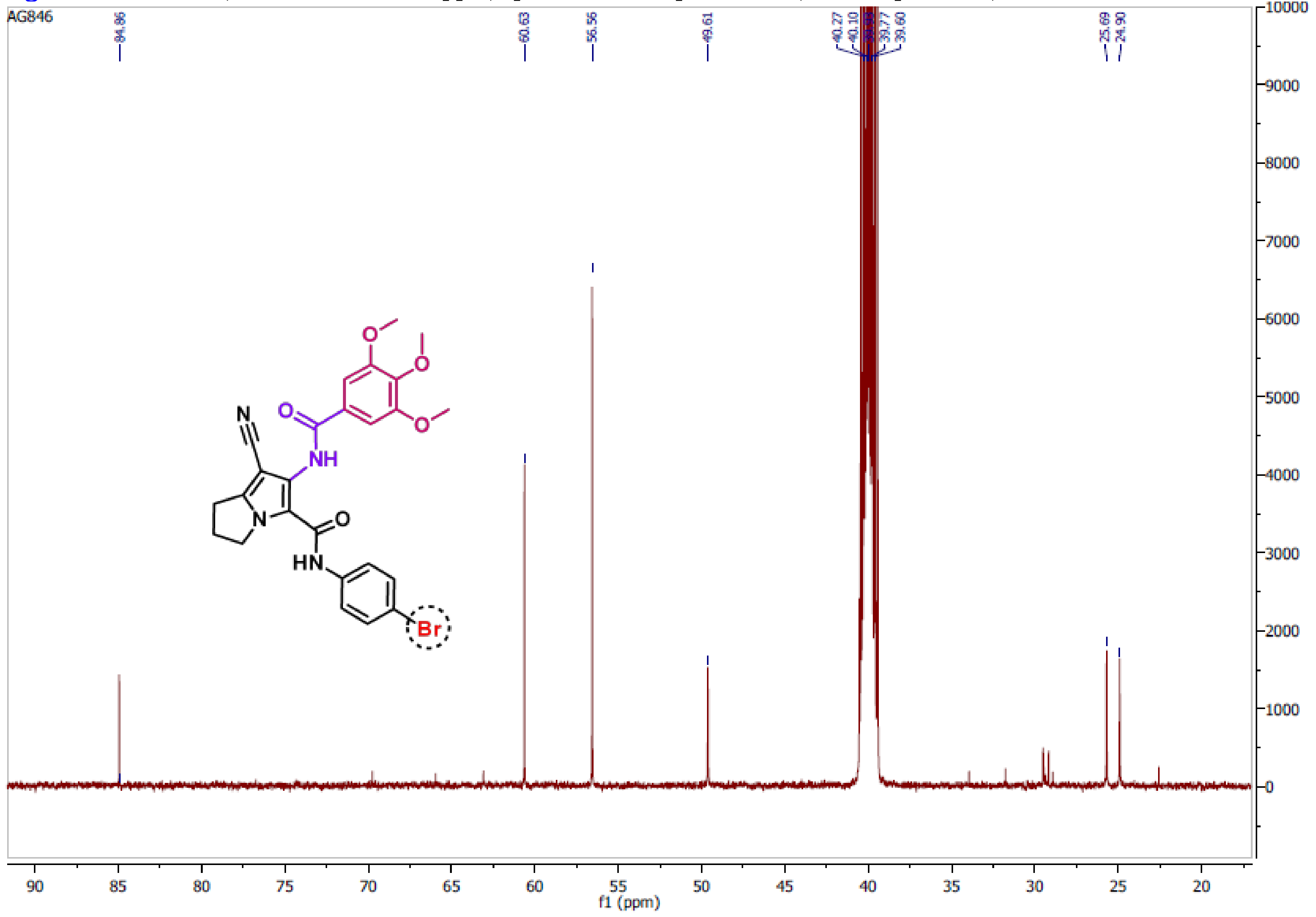

Fig. S94. ^{13}C-NMR (DMSO, 125 MHz, δ ppm) spectrum of compound **16e** (zoom, aromatic Cs)

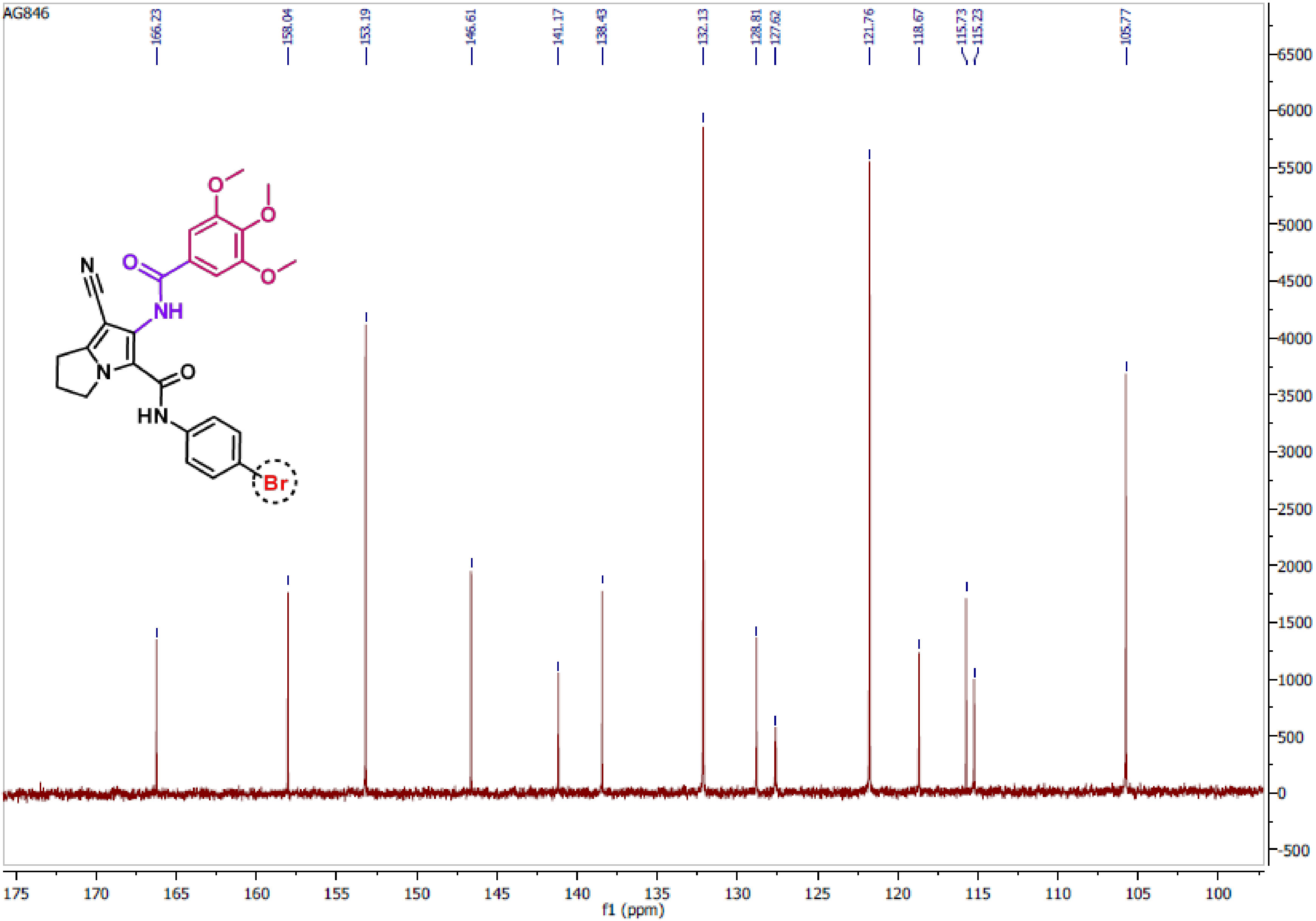

Fig. S95. **DEPT C^{135} (DMSO, 125 MHz, δ ppm) of compound 16e.**

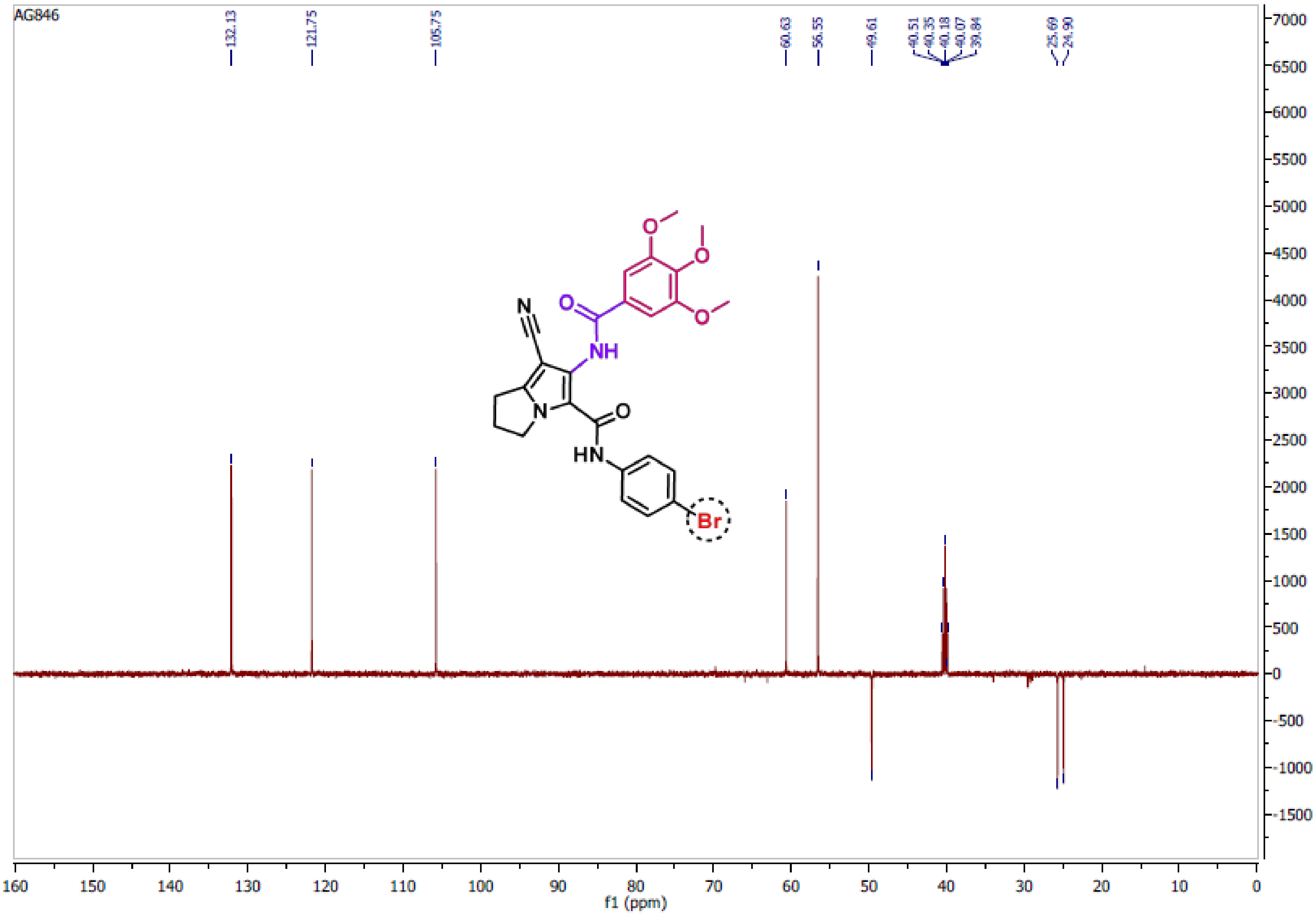

Fig. S96. ^{1}H-NMR (DMSO, 500 MHz, *δ* ppm) spectrum of compound **21**.

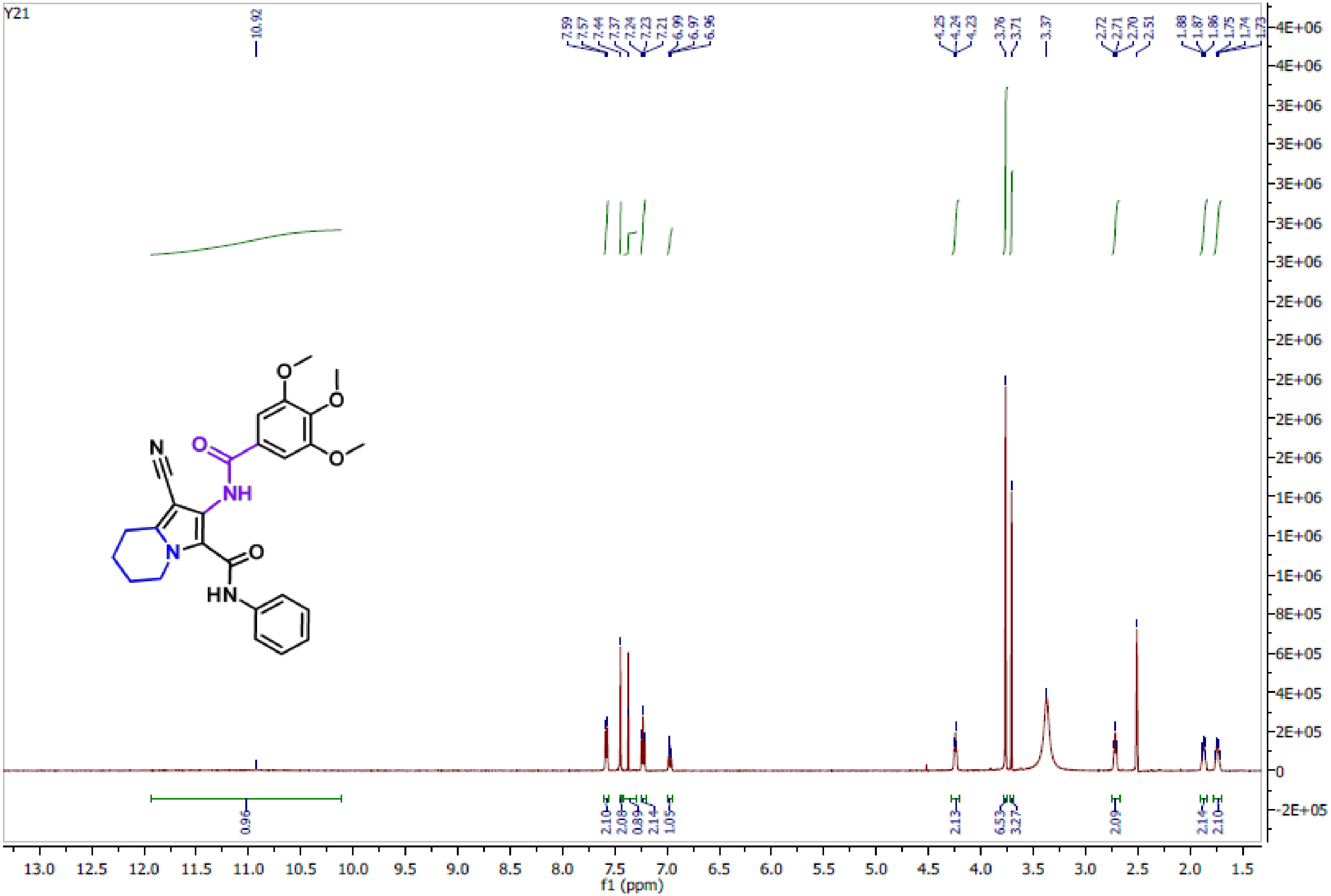

Fig. S97. ^{1}H-NMR (DMSO, 500 MHz, δ ppm) spectrum of compound **21**

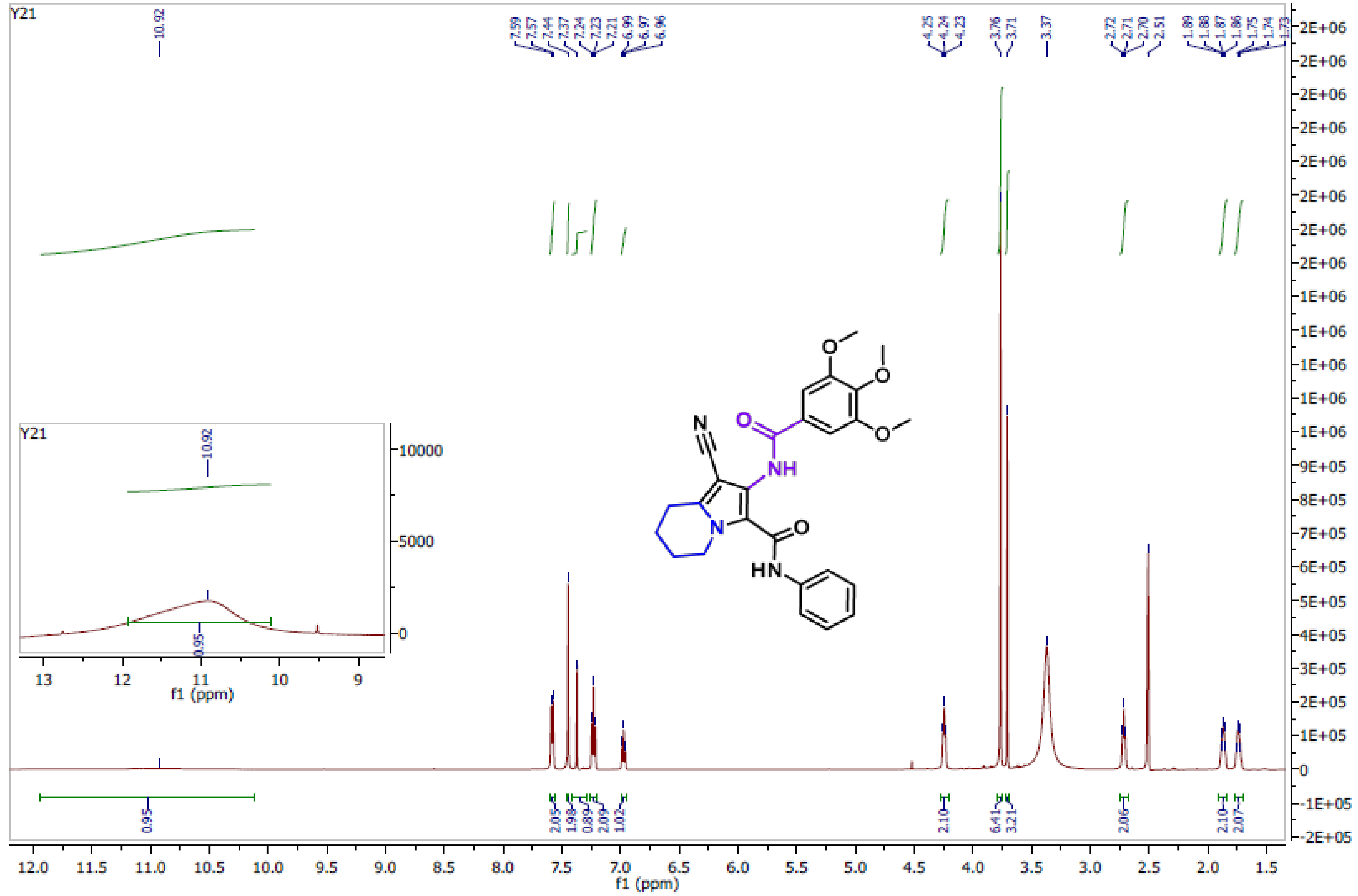

Fig. S98. ^{1}H-NMR (DMSO, 500 MHz, δ ppm) spectrum of compound **21** (zoom, aliphatic Hs)

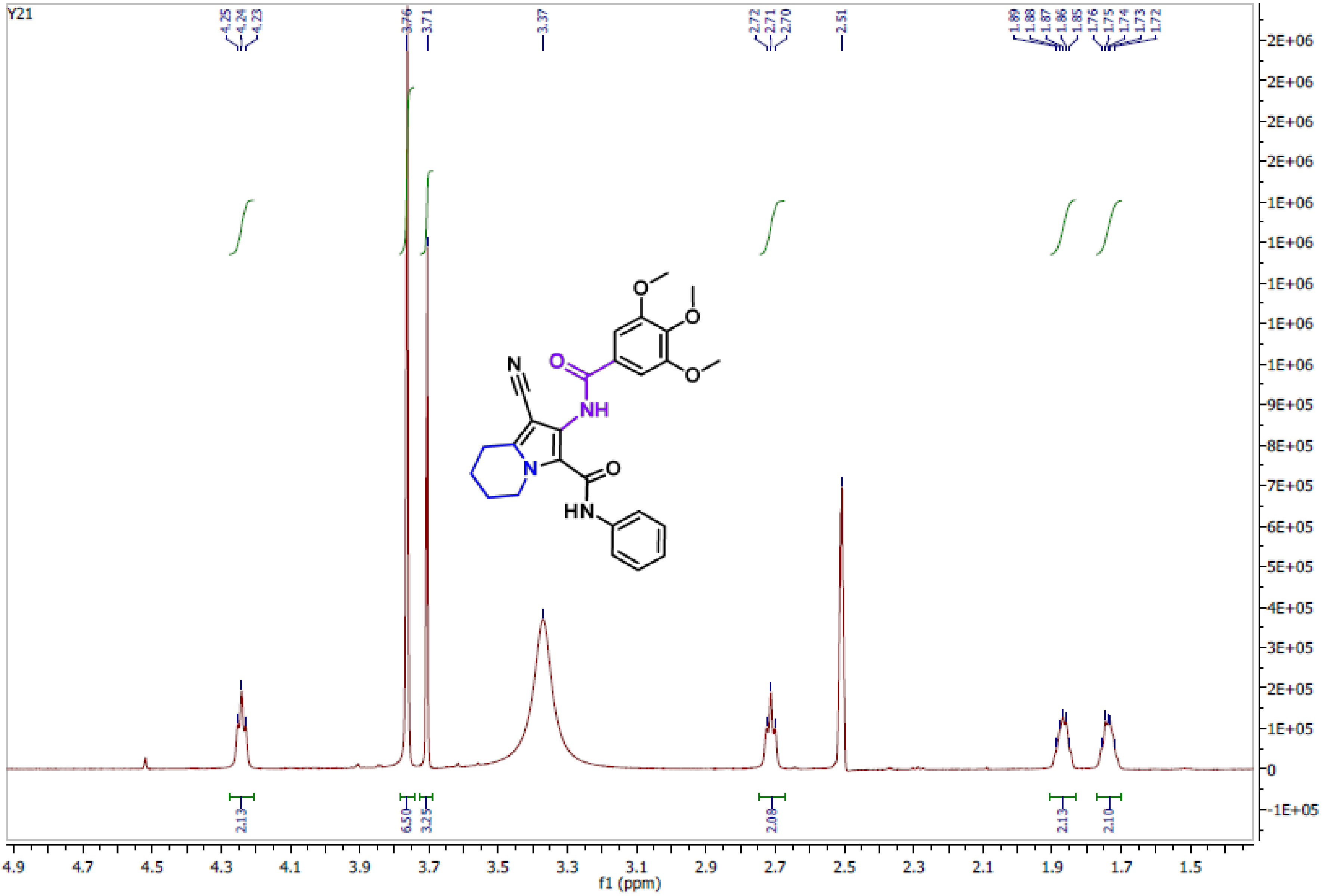

Fig. S99. ^{1}H-NMR (DMSO, 500 MHz, δ ppm) spectrum of compound **21** (**zoom, aromatic Hs & NHs**)

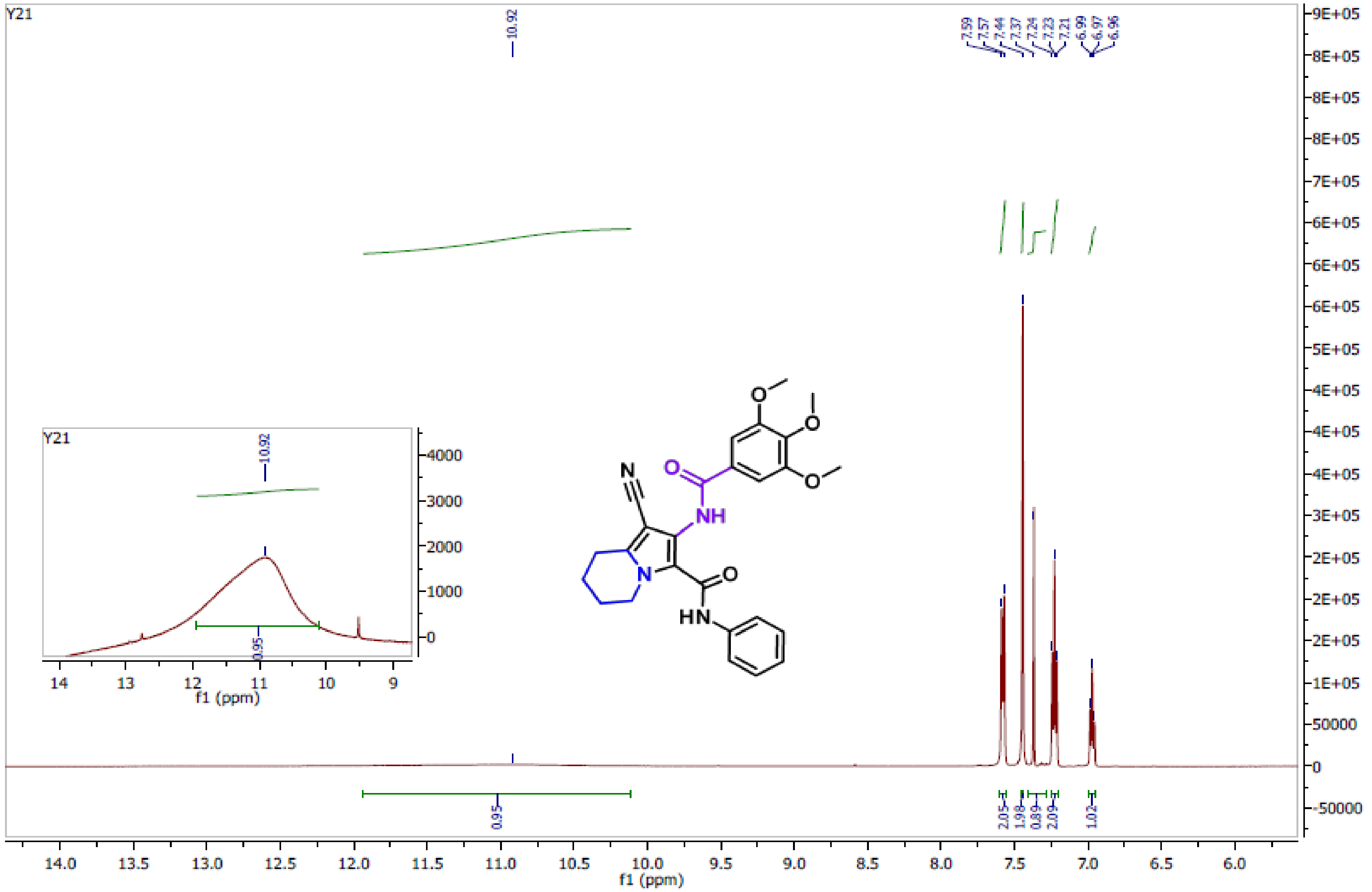

Fig. S100. ^{1}H-NMR (DMSO, 500 MHz, δ ppm) spectrum of compound **21** (zoom, aromatic Hs & NHs)

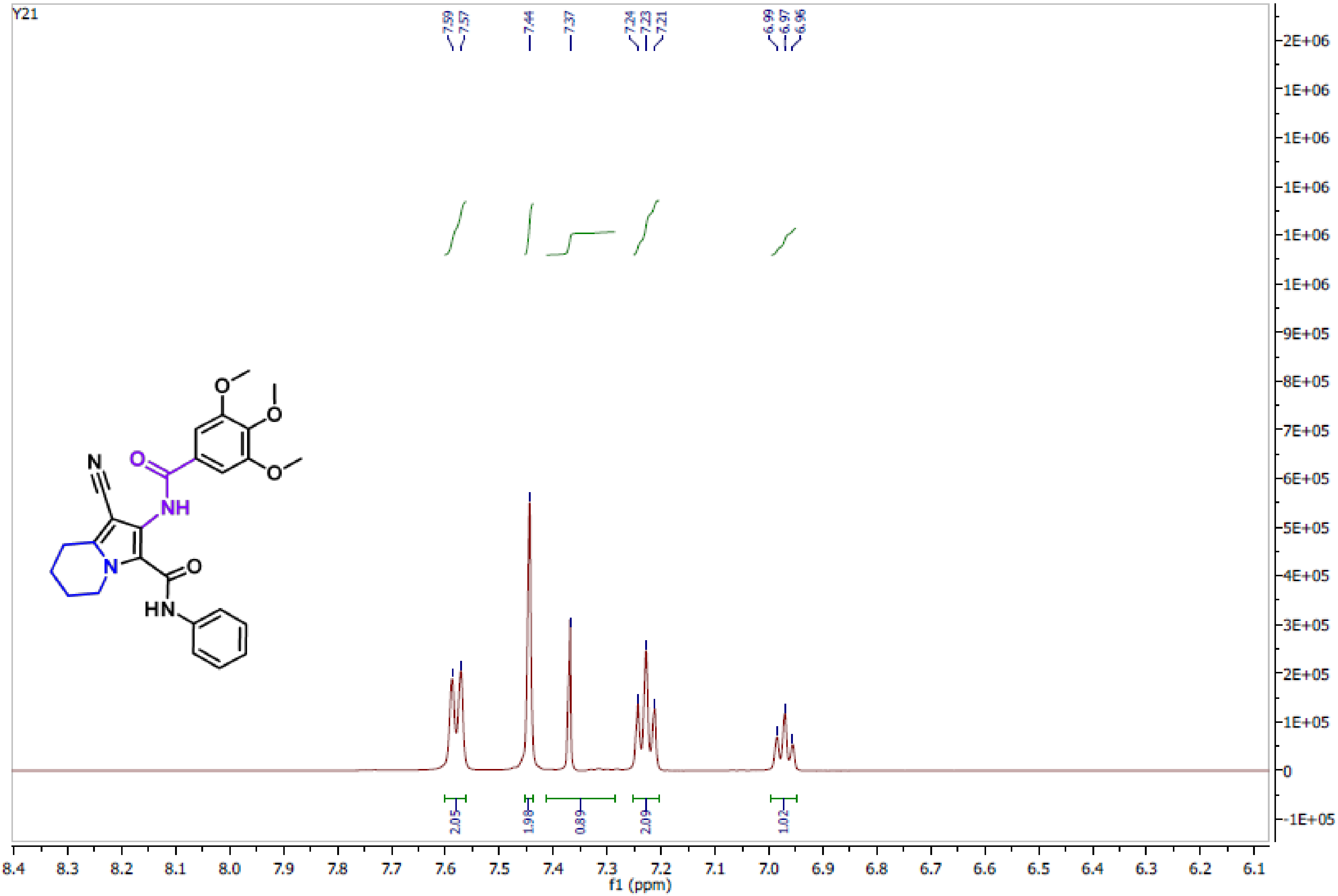

Fig. S101. ^{13}C-NMR (DMSO, 125 MHz, δ ppm) spectrum of compound **21**

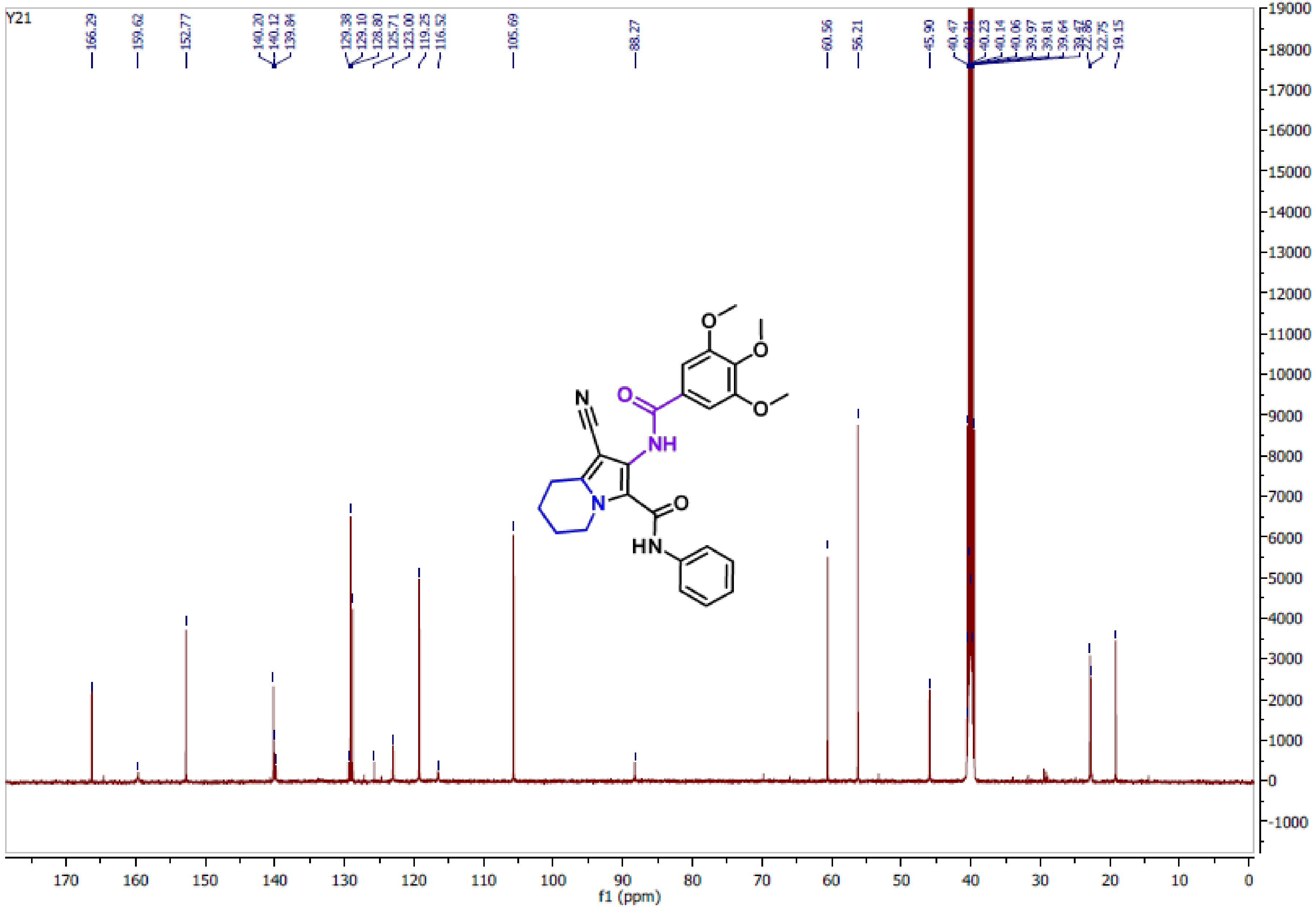

Fig. S102. ^{13}C-NMR (DMSO, 125 MHz, δ ppm) spectrum of compound **21**.

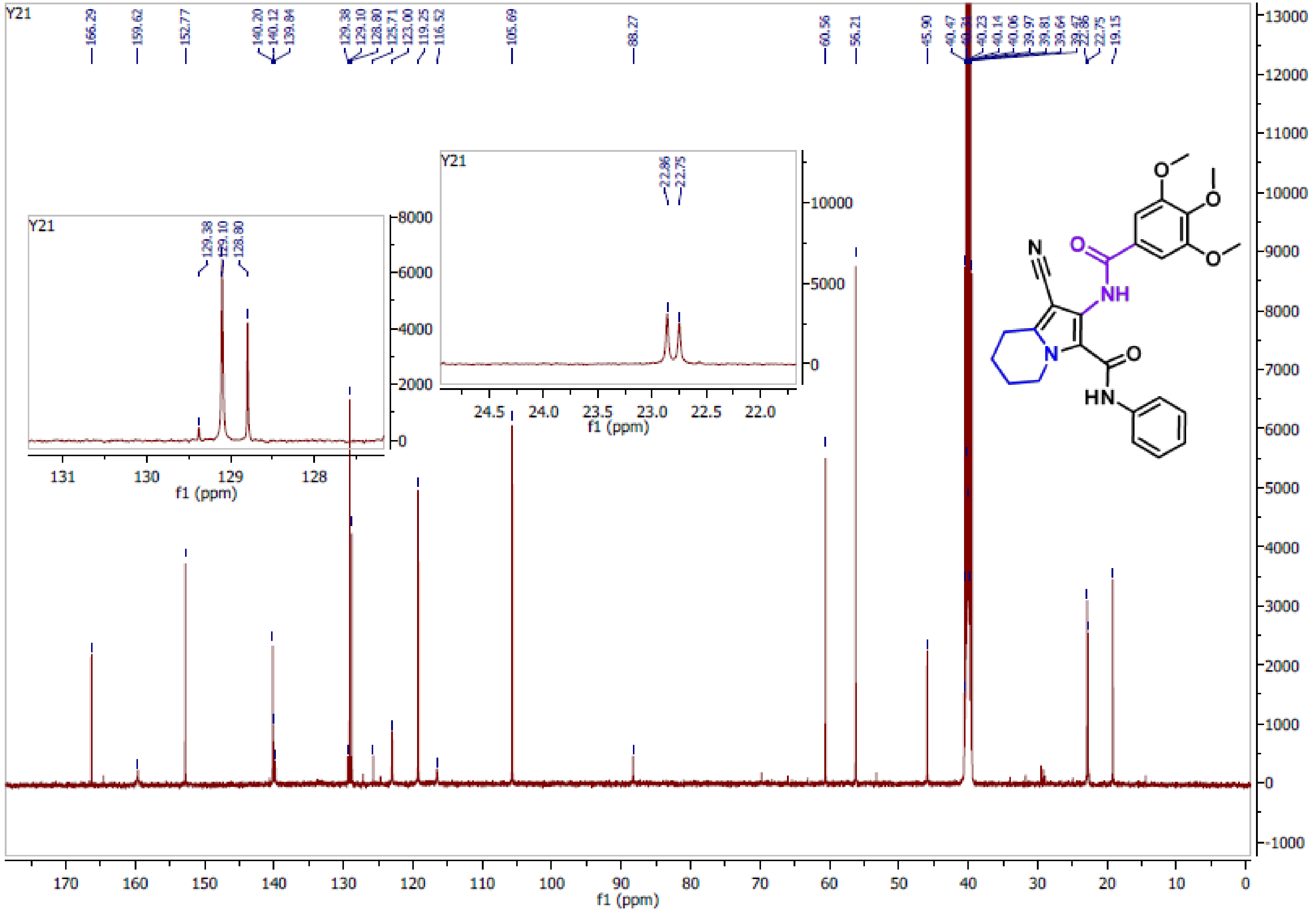

Fig. S103. ^{13}C-NMR (DMSO, 125 MHz, *δ* ppm) spectrum of compound **21** (zoom, aliphatic Cs)

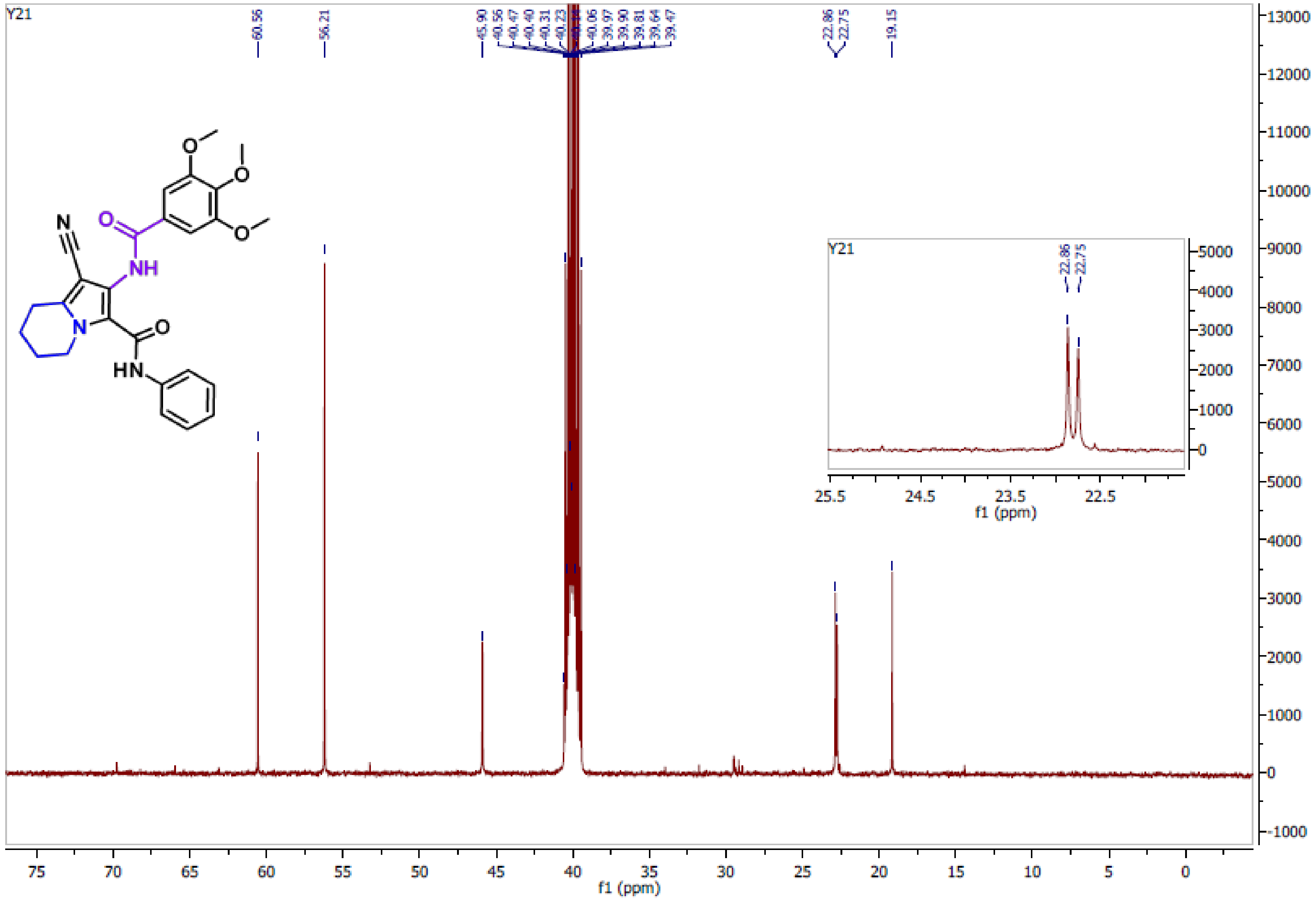

Fig. S104. ^{13}C-NMR (DMSO, 125 MHz, δ ppm) spectrum of compound **21** (zoom, aromatic Cs)

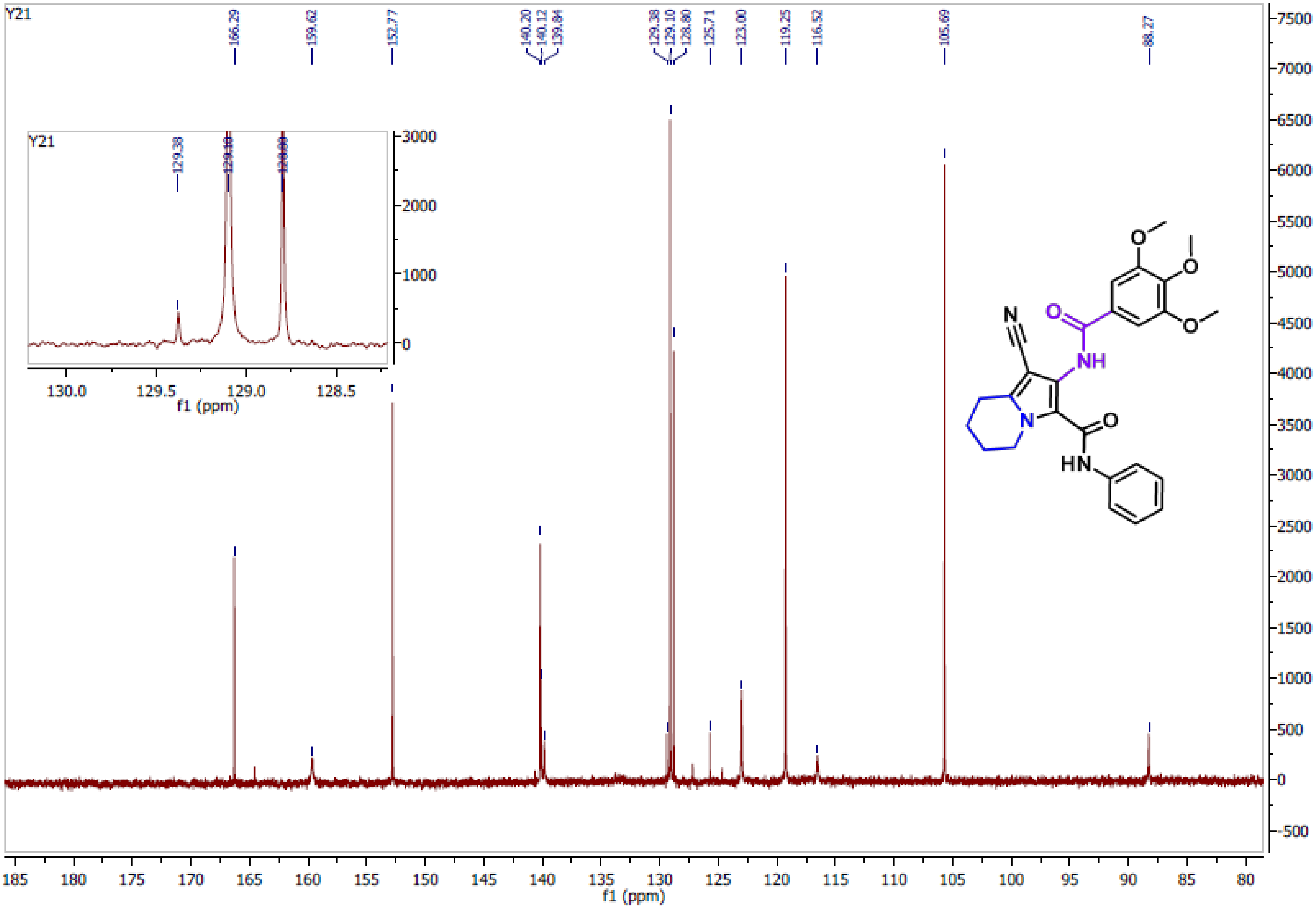

Fig. S105. DEPT C^{135} (DMSO, 125 MHz, δ ppm) of compound **21**.

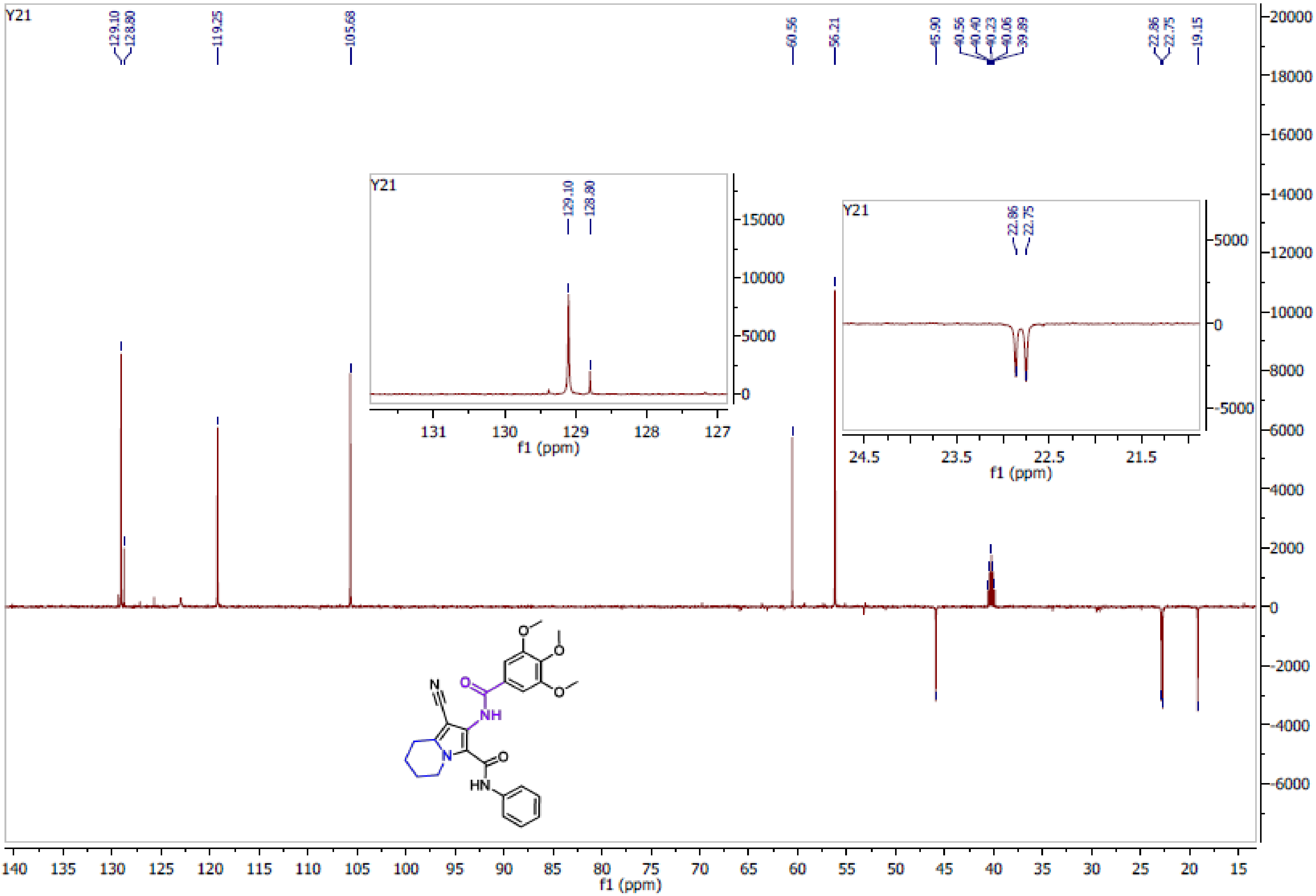

Mass Spectra

Mass spectra were recorded on Shimadzu GCMS QP5050A spectrometer, at 70 eV (EI) at the regional center for mycology and biotechnology, Al-Azhar University.

Fig. S106. **Mass spectrum of compound 15a**

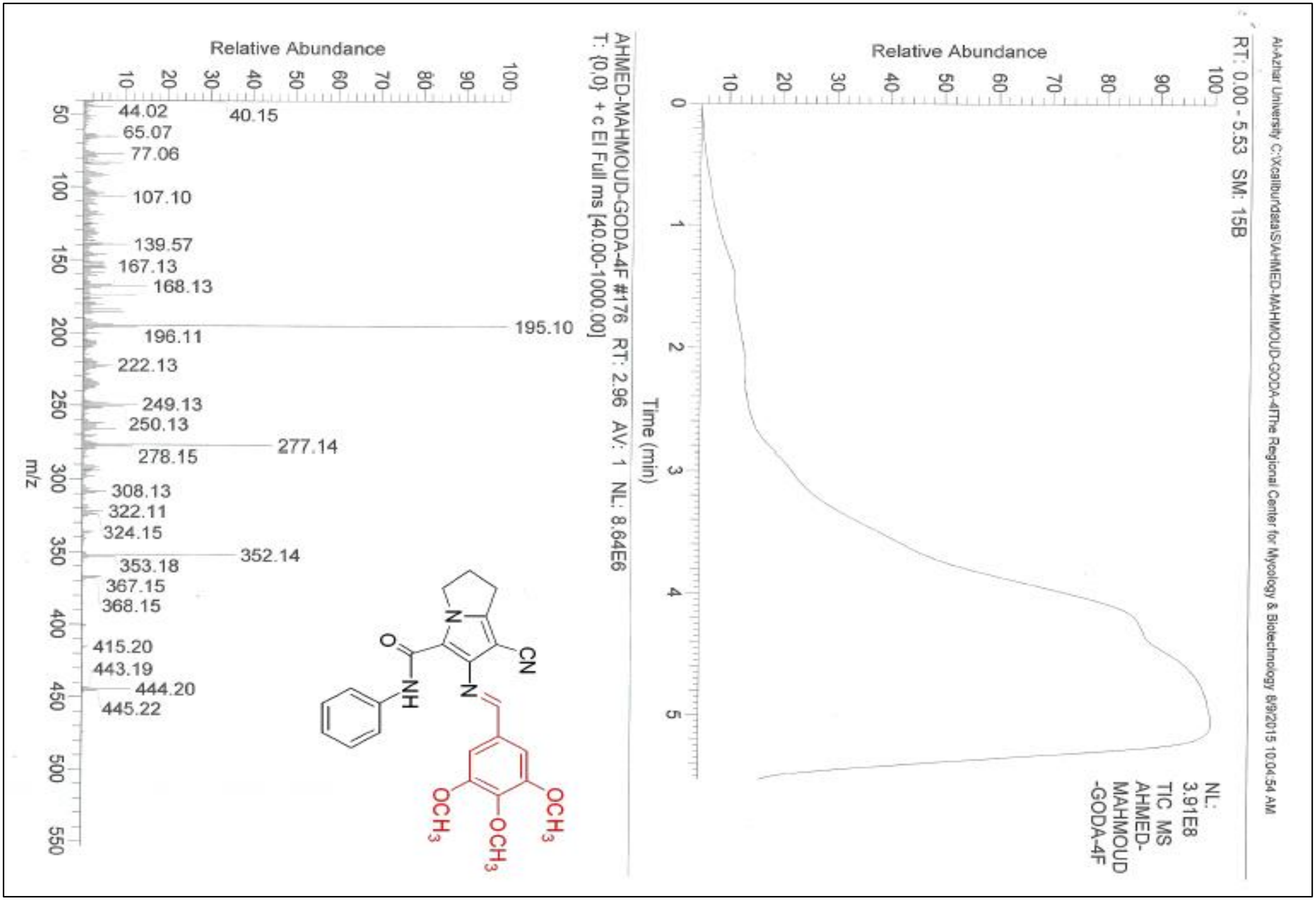

Fig. S107. Mass spectrum of compound 15a (continued)

Al-Azhar University C:\Xcalibur\data\S\AHMED-MAHMOUD-GODA-4F The Regional Center for Mycology & Biotechnology 8/9/2015 10:04:54 AM

AHMED-MAHMOUD-GODA-4F#176 RT: 2.96
T: {0,0} + c EI Full ms [40.00-1000.00]
m/z= 40.00-552.89

m/z	Intensity	Relative
40.15	2835994.8	32.81
41.09	181011.7	2.09
42.09	83336.8	0.96
43.08	195857.2	2.27
44.02	593861.0	6.87
47.00	126717.6	1.47
48.01	65825.2	0.76
50.06	88660.2	1.03
51.04	224049.0	2.59
52.06	97461.1	1.13
53.06	158740.6	1.84
55.07	107137.9	1.24
57.09	68562.6	0.79
63.04	211834.8	2.45
64.05	243346.5	2.82
65.07	687359.5	7.95
66.07	283798.5	3.28
67.07	121368.3	1.40
68.08	71393.7	0.83
69.08	88605.8	1.03
71.04	61597.7	0.71
75.06	109301.9	1.26
76.06	218875.6	2.53
77.06	835851.0	9.67
78.08	239755.0	2.77
79.07	297644.7	3.44
80.07	74263.2	0.86
81.08	214127.0	2.48
82.05	81285.4	0.94
82.98	792916.5	9.17
83.98	137679.0	1.59
84.97	508063.1	5.88
86.98	99260.2	1.15
89.12	135089.1	1.56
90.08	250917.5	2.90
91.07	376070.3	4.35
92.07	519388.5	6.01
93.08	359571.4	4.16
94.10	103360.4	1.20
95.08	146160.3	1.69
96.14	123479.5	1.43
97.16	176763.6	2.05
97.82	120971.2	1.40
98.54	77324.2	0.89
100.08	62457.7	0.72
101.07	77050.5	0.89
102.10	154174.6	1.78
103.11	233417.6	2.70
104.08	428652.3	4.96
105.06	259255.1	3.00
106.09	299900.8	3.47
107.10	864618.3	10.00

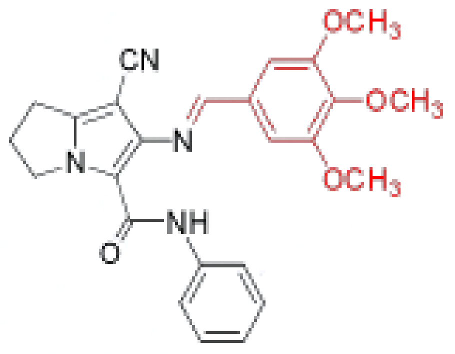

Fig. S108. Mass spectrum of compound 15a (continued)

Al-Azhar University C:\Xcalibur\data\S\AHMED-MAHMOUD-GODA-4F The Regional Center for Mycology & Biotechnology 8/9/2015 10:04:54 AM

AHMED-MAHMOUD-GODA-4F#176 RT: 2.96
T: {0,0} + c EI Full ms [40.00-1000.00]
m/z= 40.00-552.89

m/z	Intensity	Relative
108.10	131634.9	1.52
109.10	231609.7	2.68
110.12	161038.7	1.86
111.06	207948.4	2.41
111.82	273619.8	3.17
113.04	83515.7	0.97
114.08	88736.6	1.03
115.12	105811.6	1.22
116.11	123819.0	1.43
117.11	310305.5	3.59
118.10	272027.9	3.15
119.05	413827.9	4.79
120.06	159338.5	1.84
121.09	95999.4	1.11
122.06	198822.7	2.30
123.11	88725.4	1.03
124.13	167885.3	1.94
125.11	307324.8	3.56
125.82	165081.6	1.91
127.00	140893.0	1.63
128.07	134564.6	1.56
129.09	235831.5	2.73
130.09	204377.7	2.36
131.10	251451.0	2.91
132.09	337993.5	3.91
133.09	284355.6	3.29
134.06	134384.0	1.55
135.06	194907.5	2.26
136.09	76651.6	0.89
137.08	237397.1	2.75
138.55	399618.3	4.62
139.57	903537.4	10.45
140.46	347444.9	4.02
141.20	137245.0	1.59
142.11	122547.0	1.42
143.09	100165.0	1.16
144.10	108165.7	1.25
145.13	175013.3	2.02
146.10	472230.9	5.46
147.10	287796.3	3.33
148.06	104780.0	1.21
149.09	104531.2	1.21
150.07	112136.0	1.30
151.07	145058.6	1.68
152.10	418597.9	4.84
153.10	438534.1	5.07
154.20	395774.9	4.58
154.94	487697.0	5.64
156.06	465480.7	5.39
157.09	160720.7	1.86
158.10	145201.5	1.68
159.08	441377.6	5.11

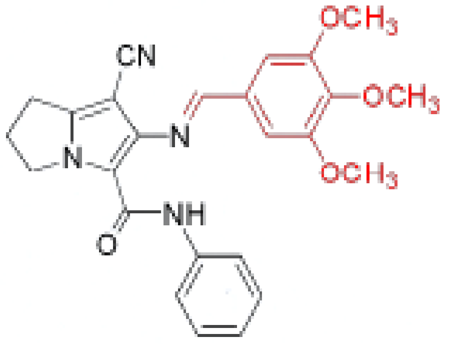

Fig. S109. Mass spectrum of compound 15a (continued)

Al-Azhar University C:\Xcalibur\data\S\AHMED-MAHMOUD-GODA-4FThe Regional Center for Mycology & Biotechnology 8/9/2015 10:04:54 AM

AHMED-MAHMOUD-GODA-4F#176 RT: 2.96
T: {0,0} + c EI Full ms [40.00-1000.00]
m/z= 40.00-552.89

m/z	Intensity	Relative
160.20	166579.7	1.93
161.02	291416.3	3.37
162.06	81718.6	0.95
163.05	93660.7	1.08
164.08	89493.5	1.04
165.08	142853.6	1.65
166.11	145765.3	1.69
167.13	594391.3	6.88
168.13	1308362.3	15.14
169.09	365347.5	4.23
170.12	124162.2	1.44
171.11	108159.5	1.25
172.09	114351.8	1.32
173.11	155326.3	1.80
174.09	1101874.6	12.75
175.14	151895.0	1.76
176.13	372063.8	4.30
177.02	129642.0	1.50
178.07	136892.5	1.58
179.10	231725.6	2.68
180.10	282655.5	3.27
181.11	408203.4	4.72
182.11	179084.6	2.07
183.13	155200.3	1.80
184.08	786907.9	9.10
185.12	165418.7	1.91
186.10	840198.9	9.72
187.10	182402.4	2.11
190.11	60585.8	0.70
191.10	185086.9	2.14
192.12	171759.9	1.99
193.12	286159.4	3.31
194.12	616981.6	7.14
195.10	8643244.0	100.00
196.11	1124772.3	13.01
197.11	214482.8	2.48
198.11	75722.4	0.88
199.09	84214.2	0.97
200.09	359574.1	4.16
201.10	73153.1	0.85
204.11	83111.5	0.96
205.12	149854.9	1.73
206.12	270139.8	3.13
207.11	285876.6	3.31
208.11	271420.4	3.14
209.11	178175.0	2.06
210.12	212056.0	2.45
211.14	88590.3	1.02
212.11	64102.2	0.74
214.12	62171.3	0.72
214.89	133896.9	1.55
217.12	61799.3	0.72

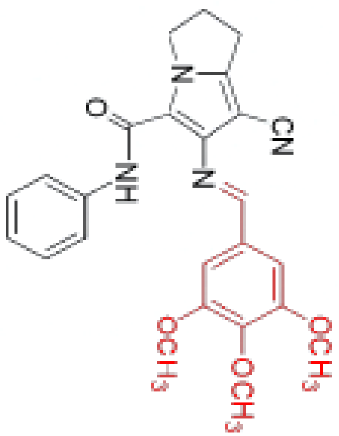

Fig. S110. Mass spectrum of compound 15a (continued)

Al-Azhar University C:\Xcalibur\data\B\AHMED-MAHMOUD-GODA-4F The Regional Center for Mycology & Biotechnology 8/9/2015 10:04:54 AM

AHMED-MAHMOUD-GODA-4F#176 RT: 2.96
T: {0,0} + c EI Full ms [40.00-1000.00]
m/z= 40.00-552.89

m/z	Intensity	Relative
218.12	182109.1	2.11
219.13	208348.1	2.41
220.13	311561.5	3.60
221.14	271084.7	3.14
222.13	605315.8	7.00
223.11	484892.5	5.61
224.12	265833.8	3.08
225.13	121662.8	1.41
226.12	69540.3	0.80
228.13	160678.2	1.86
229.13	92905.1	1.07
230.12	64283.2	0.74
231.11	78003.4	0.90
232.13	175628.2	2.03
233.12	210327.6	2.43
234.13	300943.7	3.48
235.12	345321.0	4.00
236.13	337338.4	3.90
237.15	260221.7	3.01
238.13	245512.1	2.84
239.13	112277.0	1.30
240.13	65693.1	0.76
246.12	81390.8	0.94
247.12	176631.2	2.04
248.13	520322.5	6.02
249.13	1111278.3	12.86
250.13	832947.3	9.64
251.13	419394.9	4.85
252.12	376922.3	4.36
253.12	105808.6	1.22
256.13	122667.6	1.42
260.12	109167.5	1.26
261.14	116535.4	1.35
262.14	449764.0	5.20
263.13	281686.7	3.26
264.14	282706.5	3.27
265.14	269712.9	3.12
266.13	670791.9	7.76
267.15	162087.6	1.88
270.15	298849.1	3.46
271.16	222739.8	2.58
275.12	179874.3	2.08
276.15	288361.6	3.34
277.14	3872188.3	44.80
278.15	1031966.8	11.94
279.16	261232.9	3.02
280.15	255560.2	2.96
281.13	104490.9	1.21
284.16	112429.6	1.30
290.12	237944.9	2.75
291.14	344033.8	3.98
292.13	238427.4	2.76

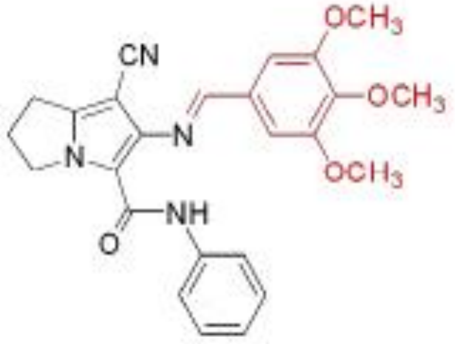

Fig. S111. **Mass spectrum of compound 15a (continued)**

Al-Azhar University C:\Xcalibur\data\S\AHMED-MAHMOUD-GODA-4\The Regional Center for Mycology & Biotechnology 8/9/2015 10:04:54 AM

AHMED-MAHMOUD-GODA-4F#176 RT: 2.96
T: {0,0} + c EI Full ms [40.00-1000.00]
m/z= 40.00-552.89

m/z	Intensity	Relative
293.14	189236.7	2.19
294.13	372419.7	4.31
295.14	143747.5	1.66
296.15	62748.2	0.73
297.15	86830.8	1.00
298.12	232830.7	2.69
299.11	88117.9	1.02
305.13	66876.1	0.77
306.14	184067.1	2.13
307.13	115465.7	1.34
308.13	492649.6	5.70
309.14	262978.2	3.04
310.15	85228.5	0.99
313.16	66952.1	0.77
318.12	121518.8	1.41
319.14	106928.8	1.24
320.13	142898.4	1.65
321.14	74827.3	0.87
322.11	425802.8	4.93
323.15	212631.7	2.46
324.15	323409.4	3.74
325.16	121323.1	1.40
336.12	220579.6	2.55
337.13	130100.0	1.51
341.16	81957.7	0.95
351.10	84574.7	0.98
352.14	3120184.3	36.10
353.18	658090.8	7.61
354.16	111197.0	1.29
367.15	379977.9	4.40
368.15	294313.1	3.41
369.15	70926.5	0.82
400.20	88881.6	1.03
415.20	131578.0	1.52
443.19	135013.5	1.56
444.20	1004933.9	11.63
445.22	315185.3	3.65

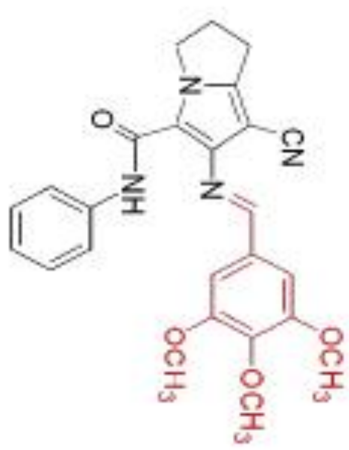

Fig. S112. **Mass spectrum of compound 15b**

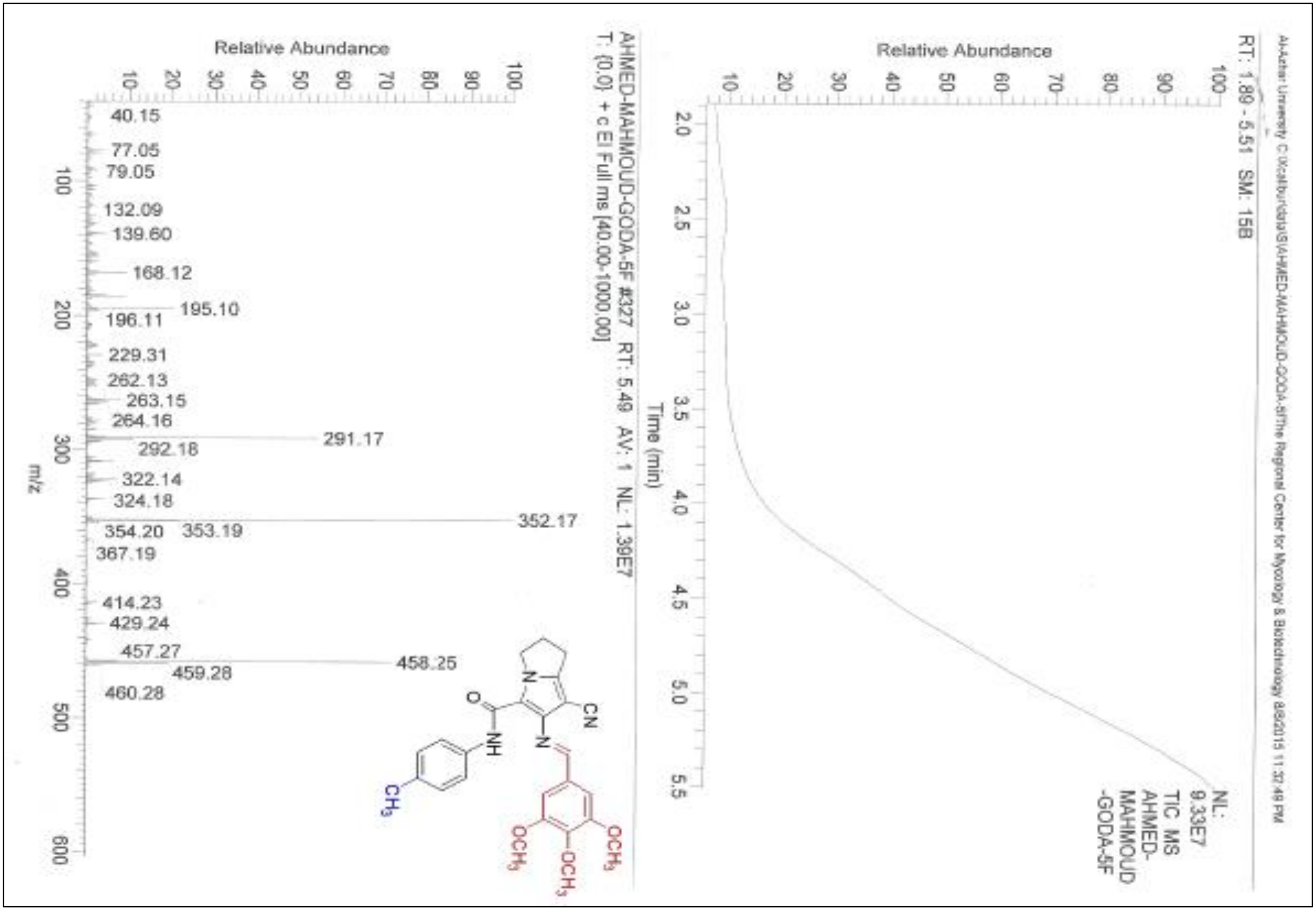

Fig. S113. **Mass spectrum of compound 15b (continued)**

Al-Azhar University C:\Xcalibur\data\S\AHMED-MAHMOUD-GODA-5FThe Regional Center for Mycology & Biotechnology 8/8/2015 11:32:49 PM

AHMED-MAHMOUD-GODA-5F#327 RT: 5.49
T: {0,0} + c EI Full ms [40.00-1000.00]
m/z= 40.00-604.18

m/z	Intensity	Relative
40.15	579009.6	4.16
41.07	104354.0	0.75
44.01	192332.7	1.38
51.03	127855.5	0.92
53.04	122213.3	0.88
63.05	103620.9	0.74
65.04	232282.6	1.67
66.04	134810.8	0.97
76.05	139272.4	1.00
77.05	585604.4	4.20
78.07	201029.0	1.44
79.05	416470.9	2.99
83.28	97688.0	0.70
90.05	166109.9	1.19
91.05	288576.7	2.07
92.06	135134.0	0.97
93.05	102495.9	0.74
96.99	102857.4	0.74
97.81	149114.6	1.07
102.06	98681.4	0.71
103.10	173893.4	1.25
104.06	283444.3	2.03
105.06	168277.6	1.21
106.07	239156.8	1.72
107.07	192449.2	1.38
109.10	105230.6	0.76
110.14	124590.0	0.89
111.05	129949.5	0.93
111.69	214392.3	1.54
117.11	151757.1	1.09
118.10	238179.5	1.71
119.04	160119.3	1.15
120.07	100171.8	0.72
125.52	267433.4	1.92
129.07	172893.7	1.24
130.11	121020.7	0.87
131.09	223789.8	1.61
132.09	324842.4	2.33
133.08	202992.3	1.46
138.50	298950.7	2.15
139.60	627580.8	4.50
140.42	163340.5	1.17
141.09	129531.8	0.93
146.06	155233.0	1.11
147.12	182378.3	1.31
152.12	159248.2	1.14
153.09	342853.3	2.46
154.14	276274.9	1.98
154.92	353711.0	2.54
156.06	400582.9	2.87
157.08	105191.6	0.75
158.09	110324.6	0.79

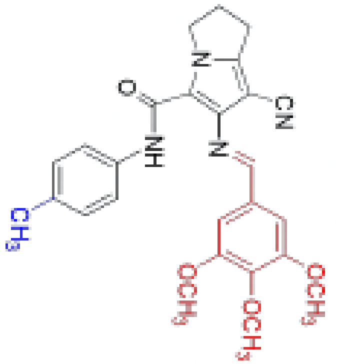

Fig. S114. **Mass spectrum of compound 15b (continued)**

Al-Azhar University C:\Xcalibur\data\S\AHMED-MAHMOUD-GODA-5F The Regional Center for Mycology & Biotechnology 8/8/2015 11:32:49 PM

AHMED-MAHMOUD-GODA-5F#327 RT: 5.49
T: {0,0} + c EI Full ms [40.00-1000.00]
m/z= 40.00-604.18

m/z	Intensity	Relative
159.10	357260.4	2.56
160.22	117197.1	0.84
161.01	174679.0	1.25
167.13	390579.1	2.80
168.12	1304293.0	9.36
169.09	275692.0	1.98
174.11	121606.4	0.87
176.11	150639.4	1.08
179.09	116006.1	0.83
180.10	152812.1	1.10
181.11	242238.6	1.74
182.13	103001.6	0.74
183.12	109734.9	0.79
184.08	638252.3	4.58
185.13	109493.8	0.79
186.11	1259500.9	9.04
187.10	170467.8	1.22
192.08	113649.8	0.82
193.09	172573.0	1.24
194.14	347213.1	2.49
195.10	2856441.0	20.50
196.11	420163.9	3.02
197.14	99499.1	0.71
206.12	187158.7	1.34
207.12	172338.0	1.24
208.11	161435.0	1.16
209.12	100949.0	0.72
210.09	109334.1	0.78
220.13	188663.6	1.35
221.26	324182.4	2.33
222.07	357701.0	2.57
223.10	319335.0	2.29
224.09	165033.6	1.18
229.31	537407.1	3.86
232.13	103075.4	0.74
233.12	104415.2	0.75
234.12	223518.6	1.60
235.12	240778.0	1.73
236.13	278789.3	2.00
237.14	208764.2	1.50
238.11	193817.2	1.39
247.13	240779.5	1.73
248.13	367356.7	2.64
249.12	230428.4	1.65
250.12	338682.5	2.43
251.12	256671.4	1.84
252.12	317315.8	2.28
260.12	105043.8	0.75
261.13	187016.7	1.34
262.13	520805.9	3.74
263.15	1141007.3	8.19
264.16	683702.4	4.91

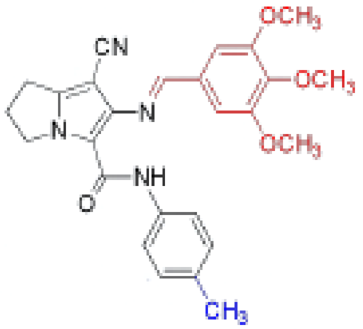

Fig. S115. **Mass spectrum of compound 15b (continued)**

Al-Azhar University C:\Xcalibur\data\S\AHMED-MAHMOUD-GODA-5fThe Regional Center for Mycology & Biotechnology 8/8/2015 11:32:49 PM

AHMED-MAHMOUD-GODA-5F#327 RT: 5.49
T: (0,0) + c EI Full ms [40.00-1000.00]
m/z= 40.00-604.18

m/z	Intensity	Relative
265.14	458940.2	3.29
266.13	365772.4	2.63
276.13	220666.1	1.58
277.14	170186.6	1.22
278.13	482342.8	3.46
279.16	223124.3	1.60
280.15	310510.3	2.23
281.14	131972.2	0.95
284.17	393163.1	2.82
285.19	274428.6	1.97
289.18	227129.0	1.63
290.17	611135.6	4.39
291.17	7590729.0	54.48
292.18	1541891.5	11.07
293.16	435763.7	3.13
294.13	567326.7	4.07
295.13	171385.4	1.23
306.16	304292.9	2.18
307.19	166897.2	1.20
308.16	927223.7	6.65
309.17	510047.4	3.66
310.16	135169.7	0.97
313.18	137048.3	0.98
318.13	294252.3	2.11
319.16	216287.6	1.55
320.15	253978.1	1.82
321.19	168326.6	1.21
322.14	1010354.3	7.25
323.17	458752.4	3.29
324.18	733195.4	5.26
325.18	232112.3	1.67
336.13	620691.8	4.45
337.17	319681.0	2.29
338.18	124301.0	0.89
350.21	164425.1	1.18
351.07	204922.1	1.47
352.17	13933503.0	100.00
353.19	2988715.3	21.45
354.20	427039.5	3.06
367.19	160466.3	1.15
414.23	377099.1	2.71
415.23	160074.3	1.15
426.21	170862.4	1.23
427.24	119321.1	0.86
429.24	622682.9	4.47
430.25	296311.9	2.13
441.24	185245.0	1.33
457.27	1011814.3	7.26
458.25	10007973.0	71.83
459.28	2678139.3	19.22
460.28	471999.5	3.39

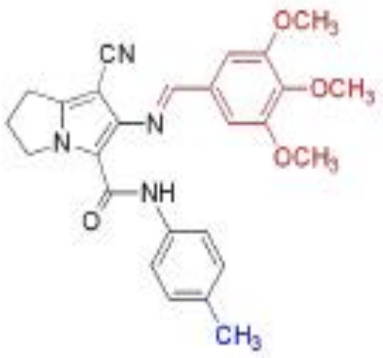

Fig. S116. **Mass spectrum of compound 15c**

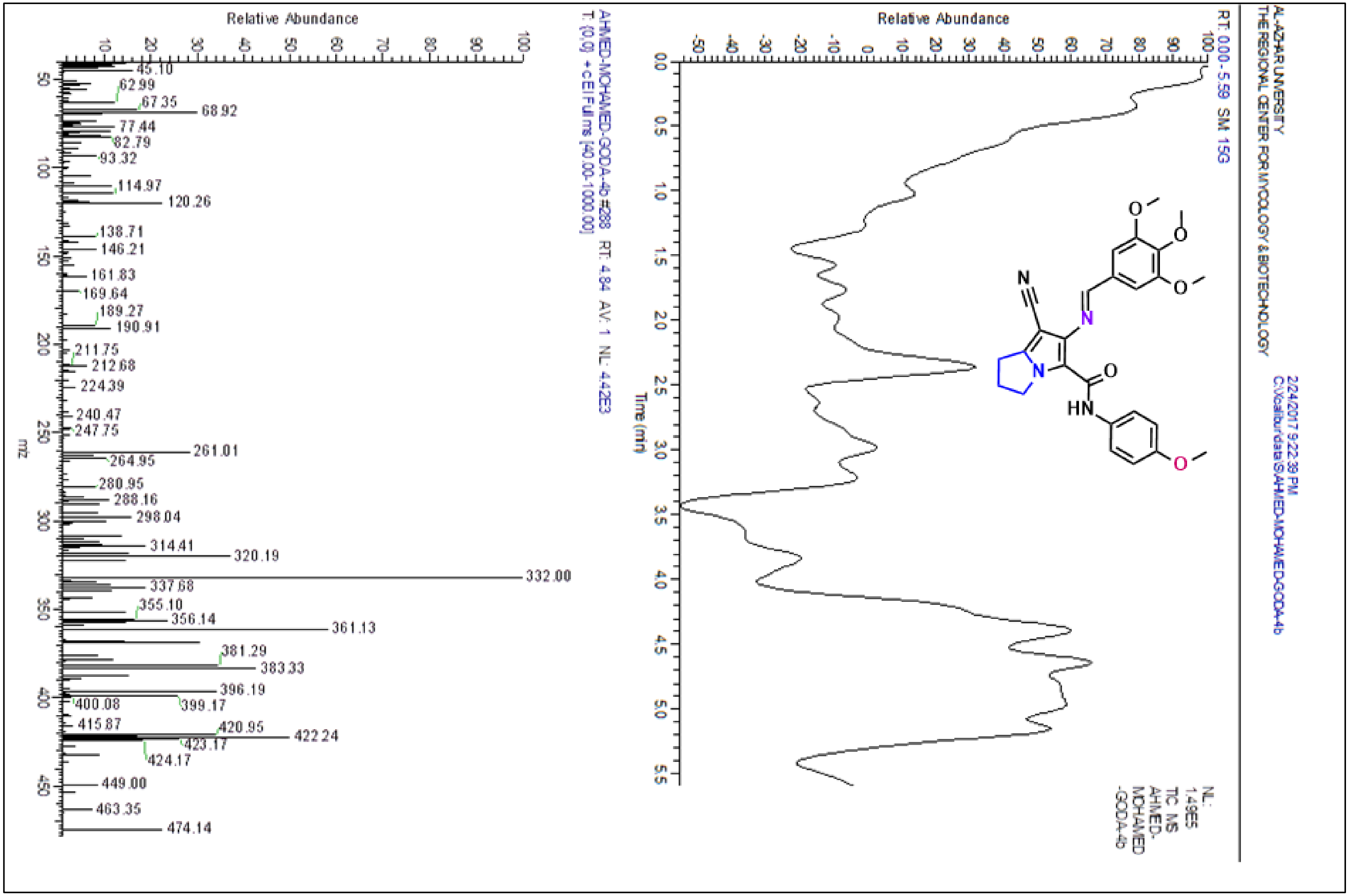

Fig. S117. **Mass spectrum of compound 15d**

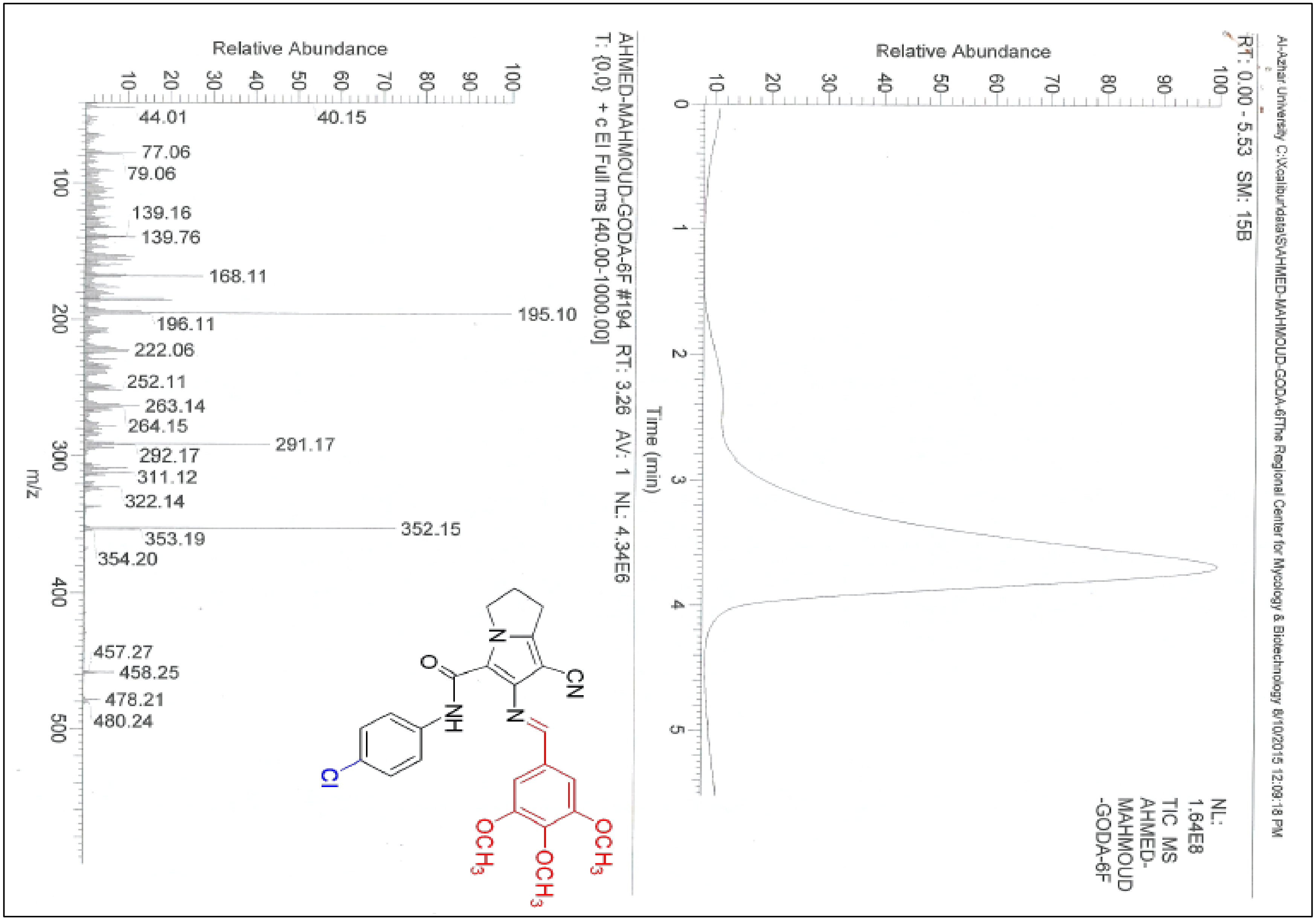

Fig. S118. **Mass spectrum of compound 15d (continued)**

Al-Azhar University C:\Xcalibur\data\S\AHMED-MAHMOUD-GODA-6FThe Regional Center for Mycology & Biotechnology 8/10/2015 12:09:18 PM

AHMED-MAHMOUD-GODA-6F#194 RT: 3.26
T: {0,0} + c EI Full ms [40.00-1000.00]
m/z= 40.00-599.95

m/z	Intensity	Relative
40.15	2297213.8	52.95
41.07	110337.2	2.54
42.10	48544.7	1.12
43.10	100428.7	2.31
44.01	483485.6	11.14
45.05	43726.5	1.01
50.04	41183.2	0.95
51.04	112973.3	2.60
52.07	60177.6	1.39
53.05	108291.8	2.50
54.07	34440.8	0.79
55.07	71289.0	1.64
57.08	53614.1	1.24
63.04	138125.1	3.18
64.06	120645.0	2.78
65.08	186001.4	4.29
66.07	123679.2	2.85
67.06	85491.2	1.97
68.06	44137.4	1.02
69.07	73788.2	1.70
70.18	35515.5	0.82
71.10	43279.0	1.00
73.03	34562.5	0.80
75.05	93446.2	2.15
76.05	161564.0	3.72
77.06	511402.6	11.79
78.07	188687.5	4.35
79.06	364093.5	8.39
80.08	65313.4	1.51
81.06	104239.6	2.40
82.12	43007.8	0.99
83.13	91111.9	2.10
84.03	122600.2	2.83
85.04	76518.3	1.76
88.07	38789.6	0.89
89.11	101645.2	2.34
90.09	241200.0	5.56
91.08	283487.2	6.53
92.06	148258.2	3.42
93.06	127818.3	2.95
94.10	50689.2	1.17
95.08	120389.8	2.77
96.12	64964.2	1.50
97.12	140704.7	3.24
97.99	130180.4	3.00
99.02	180646.7	4.16
100.10	57346.5	1.32
101.07	94456.2	2.18
102.09	129369.1	2.98
103.12	185665.1	4.28
104.06	278766.4	6.43
105.05	191076.8	4.40

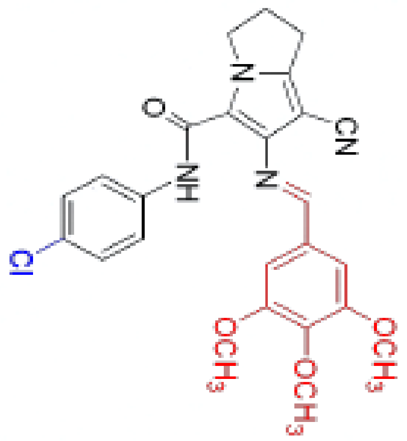

Fig. S119. **Mass spectrum of compound 15d (continued)**

Al-Azhar University C:\Xcalibur\data\S\AHMED-MAHMOUD-GODA-6f The Regional Center for Mycology & Biotechnology 8/10/2015 12:09:18 PM

AHMED-MAHMOUD-GODA-6F#194 RT: 3.26
T: {0,0} + c EI Full ms [40.00-1000.00]
m/z= 40.00-599.95

m/z	Intensity	Relative
106.10	213853.6	4.93
107.09	191311.6	4.41
108.09	55199.9	1.27
109.08	128989.9	2.97
110.10	147541.2	3.40
111.18	282639.1	6.51
111.96	181811.4	4.19
113.06	80478.6	1.86
114.06	76330.8	1.76
115.08	79301.7	1.83
116.07	83494.1	1.92
117.09	196753.6	4.54
118.10	256535.3	5.91
119.06	200501.7	4.62
120.07	114571.8	2.64
121.08	72982.4	1.68
122.05	97736.6	2.25
123.09	59563.5	1.37
124.19	95875.1	2.21
125.06	264968.5	6.11
125.79	198390.7	4.57
126.41	143012.4	3.30
127.11	140900.0	3.25
128.07	137192.1	3.16
129.10	195724.6	4.51
130.09	153859.6	3.55
131.11	236613.7	5.45
132.10	318196.3	7.33
133.09	228175.3	5.26
134.07	82554.8	1.90
135.09	96282.5	2.22
136.10	46135.1	1.06
137.06	111447.2	2.57
138.26	211478.4	4.87
139.16	411027.1	9.47
139.76	506330.5	11.67
140.32	239991.0	5.53
141.01	206443.5	4.76
142.07	101577.3	2.34
143.08	70639.9	1.63
144.09	62153.6	1.43
145.14	114357.3	2.64
146.09	197137.1	4.54
147.12	239819.9	5.53
148.14	72492.5	1.67
149.14	64024.4	1.48
150.10	72932.9	1.68
151.10	82933.5	1.91
152.11	206567.5	4.76
153.07	416892.9	9.61
154.27	502467.9	11.58
155.02	330261.5	7.61

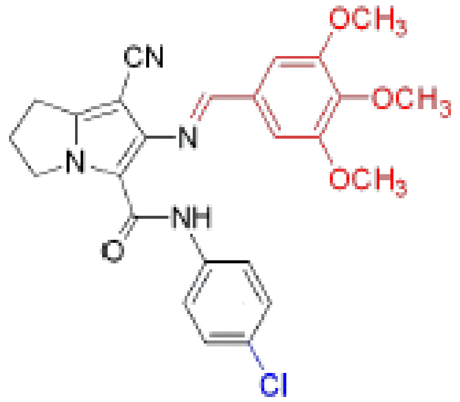

Fig. S120. Mass spectrum of compound 15d (continued)

Al-Azhar University C:\Xcalibur\data\S\AHMED-MAHMOUD-GODA-6FThe Regional Center for Mycology & Biotechnology 8/10/2015 12:09:18 PM

AHMED-MAHMOUD-GODA-6F#194 RT: 3.26
T: {0,0} + c EI Full ms [40.00-1000.00]
m/z= 40.00-599.95

m/z	Intensity	Relative
156.06	463744.9	10.69
157.08	142709.3	3.29
158.11	120691.0	2.78
159.10	375809.6	8.66
160.26	156743.4	3.61
161.05	245880.0	5.67
162.05	72196.3	1.66
163.06	70405.9	1.62
164.09	64124.4	1.48
165.09	95062.3	2.19
166.11	114665.9	2.64
167.12	423739.0	9.77
168.11	1200610.0	27.67
169.06	363144.3	8.37
170.11	94870.7	2.19
171.08	75603.5	1.74
172.07	67153.1	1.55
173.09	98105.1	2.26
174.11	217824.1	5.02
175.12	49502.4	1.14
176.14	352594.9	8.13
177.05	95123.9	2.19
178.09	114461.9	2.64
179.10	189949.9	4.38
180.10	237282.0	5.47
181.11	294241.2	6.78
182.10	156477.4	3.61
183.12	144107.5	3.32
184.09	804831.7	18.55
185.11	148516.3	3.42
186.11	884352.2	20.38
187.12	158333.3	3.65
188.12	35211.3	0.81
190.12	32863.4	0.76
191.10	109673.9	2.53
192.10	136783.3	3.15
193.10	210329.0	4.85
194.13	572452.3	13.19
195.10	4338426.5	100.00
196.11	667242.7	15.38
197.11	146689.6	3.38
198.14	58772.6	1.35
199.06	52960.4	1.22
200.12	86453.5	1.99
201.11	31574.7	0.73
202.09	32375.6	0.75
203.10	35611.1	0.82
204.09	66791.3	1.54
205.13	115426.4	2.66
206.12	240977.5	5.55
207.11	226326.5	5.22
208.11	218981.8	5.05

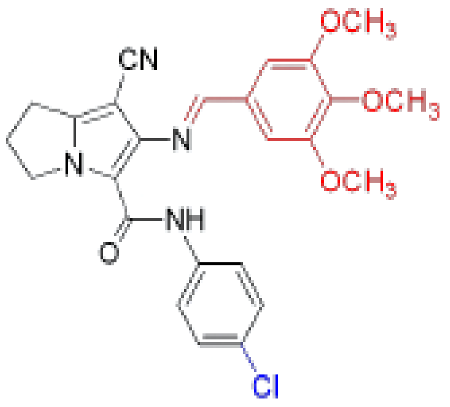

Fig. S121. **Mass spectrum of compound 15d (continued)**

Al-Azhar University C:\Xcalibur\data\S\AHMED-MAHMOUD-GODA-6F The Regional Center for Mycology & Biotechnology 8/10/2015 12:09:18 PM

AHMED-MAHMOUD-GODA-6F#194 RT: 3.26
T: {0,0} + c EI Full ms [40.00-1000.00]
m/z= 40.00-599.95

m/z	Intensity	Relative
209.12	158398.4	3.65
210.12	241663.4	5.57
211.13	83676.0	1.93
212.14	52268.7	1.20
213.08	39123.4	0.90
214.15	66465.7	1.53
217.14	48654.8	1.12
218.11	89382.8	2.06
219.12	110966.7	2.56
220.12	256550.2	5.91
221.22	325422.6	7.50
222.06	451150.8	10.40
223.12	439423.6	10.13
224.13	213508.0	4.92
225.11	83766.6	1.93
226.14	39901.5	0.92
228.15	62192.8	1.43
229.24	329419.5	7.59
230.23	38759.3	0.89
231.17	89744.0	2.07
232.08	187844.0	4.33
233.12	132910.9	3.06
234.12	261201.0	6.02
235.12	248570.5	5.73
236.13	273604.2	6.31
237.14	207316.9	4.78
238.15	214545.7	4.95
239.15	223903.7	5.16
240.11	122337.4	2.82
241.08	37952.8	0.87
246.15	74018.3	1.71
247.13	150061.5	3.46
248.13	275410.3	6.35
249.14	205420.1	4.73
250.13	314324.9	7.25
251.13	241295.4	5.56
252.11	374373.7	8.63
253.12	88984.0	2.05
254.14	39492.2	0.91
255.16	34161.1	0.79
256.15	31482.0	0.73
259.13	35753.0	0.82
260.13	89224.8	2.06
261.13	124615.9	2.87
262.14	357149.4	8.23
263.14	563267.8	12.98
264.15	386639.8	8.91
265.13	249674.3	5.75
266.14	278348.1	6.42
267.15	87883.8	2.03
268.11	170185.1	3.92
269.13	46908.7	1.08

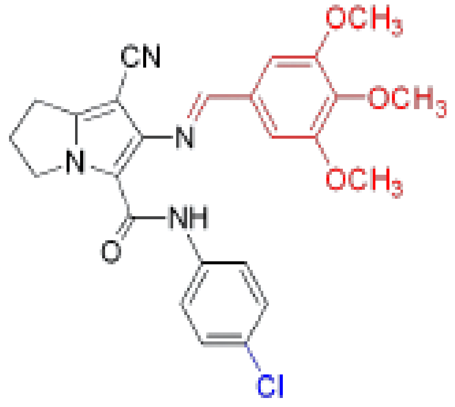

Fig. S122. **Mass spectrum of compound 15d (continued)**

Al-Azhar University C:\Xcalibur\data\S\AHMED-MAHMOUD-GODA-6F The Regional Center for Mycology & Biotechnology 8/10/2015 12:09:18 PM

AHMED-MAHMOUD-GODA-6F#194 RT: 3.26
T: {0,0} + c EI Full ms [40.00-1000.00]
m/z= 40.00-599.95

m/z	Intensity	Relative
270.14	56794.0	1.31
271.14	47757.7	1.10
274.13	41314.5	0.95
275.14	65504.7	1.51
276.12	149304.1	3.44
277.15	129648.0	2.99
278.15	326154.5	7.52
279.17	140601.3	3.24
280.16	182277.9	4.20
281.17	93882.0	2.16
282.15	81858.3	1.89
283.11	151724.3	3.50
284.14	175641.8	4.05
285.16	136518.9	3.15
286.13	48695.9	1.12
289.16	86759.8	2.00
290.15	296724.5	6.84
291.17	1891572.1	43.60
292.17	509643.7	11.75
293.16	185169.2	4.27
294.15	300206.0	6.92
295.14	107543.0	2.48
296.13	45737.5	1.05
299.13	35489.2	0.82
304.14	75213.6	1.73
305.14	75698.9	1.74
306.13	160286.0	3.69
307.15	93936.0	2.17
308.16	436601.1	10.06
309.17	241835.8	5.57
310.20	75409.0	1.74
311.12	483534.6	11.15
312.12	126113.6	2.91
313.13	216197.0	4.98
314.12	46725.6	1.08
318.12	103584.3	2.39
319.15	79819.3	1.84
320.17	91826.5	2.12
321.24	66616.2	1.54
322.14	357416.1	8.24
323.16	152993.0	3.53
324.18	190362.9	4.39
325.18	66210.0	1.53
336.16	177317.4	4.09
337.15	90869.4	2.09
350.20	30789.8	0.71
351.10	61388.7	1.41
352.15	3171725.5	73.11
353.19	565112.8	13.03
354.20	84965.1	1.96
367.17	38657.7	0.89
457.27	45854.1	1.06

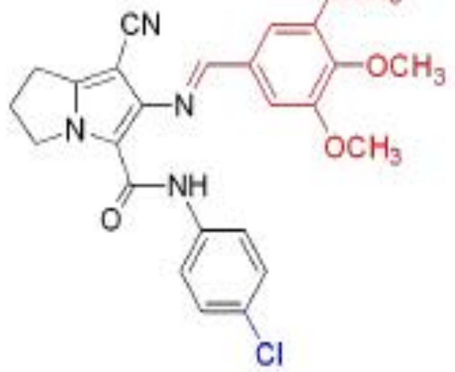

Fig. S123. **Mass spectrum of compound 15d (continued)**

Al-Azhar University C:\Xcalibur\data\S\AHMED-MAHMOUD-GODA-6\The Regional Center for Mycology & Biotechnology 8/10/2015 12:09:18 PM

AHMED-MAHMOUD-GODA-6F#194 RT: 3.26
T: {0,0} + c EI Full ms [40.00-1000.00]
m/z= 40.00-599.95

m/z	Intensity	Relative
323.16	152993.0	3.53
324.18	190362.9	4.39
325.18	66210.0	1.53
336.16	177317.4	4.09
337.15	90869.4	2.09
350.20	30789.8	0.71
351.10	61388.7	1.41
352.15	3171725.5	73.11
353.19	565112.8	13.03
354.20	84965.1	1.96
367.17	38657.7	0.89
457.27	45854.1	1.06
458.25	309632.0	7.14
459.28	105168.8	2.42
478.21	167867.6	3.87
479.21	45198.6	1.04
480.24	49240.7	1.13

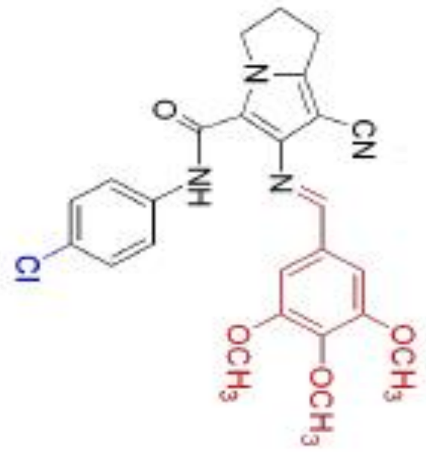

Fig. S124. Mass spectrum of compound **15e**.

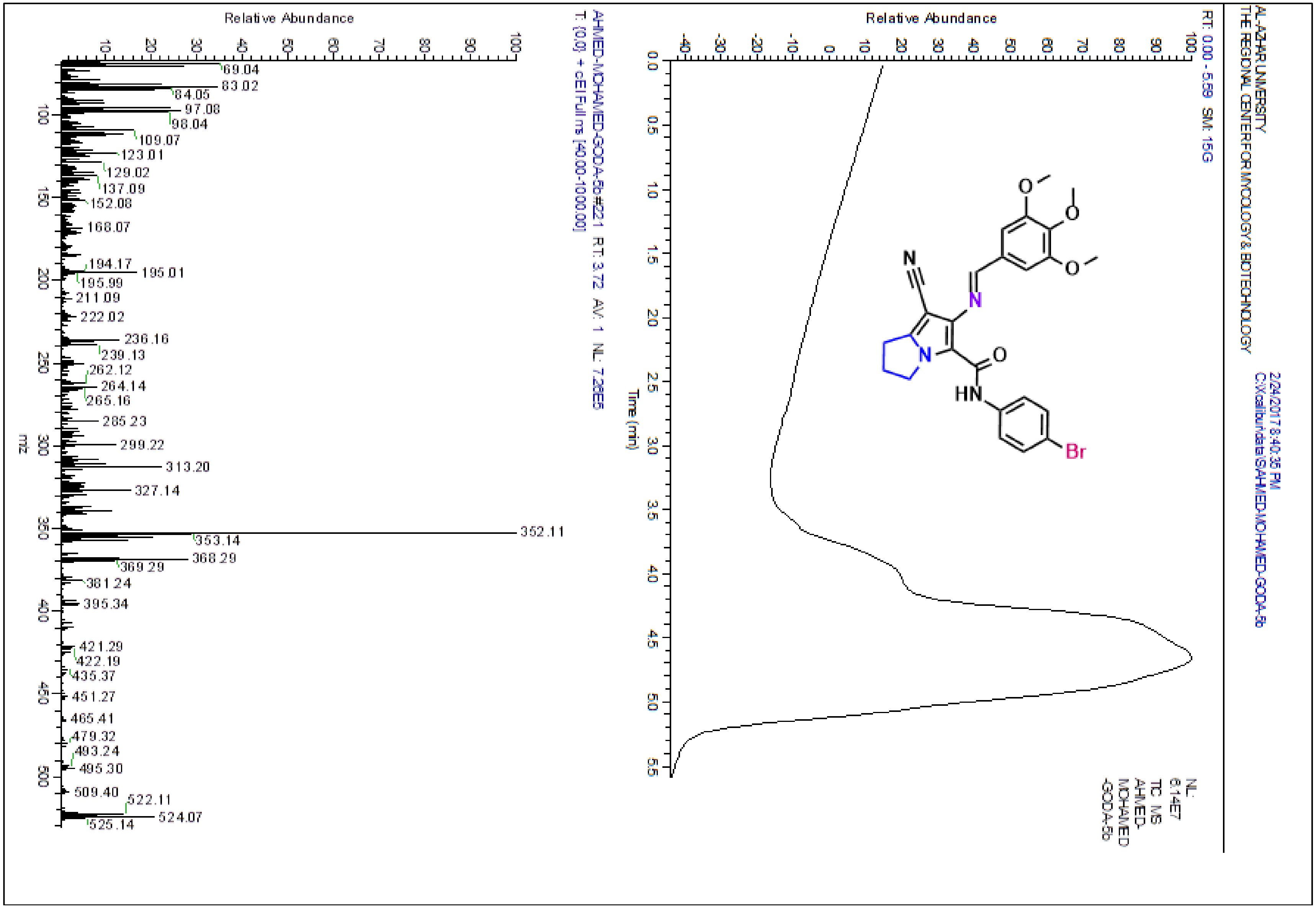

Fig. S125. **Mass spectrum of compound 20**

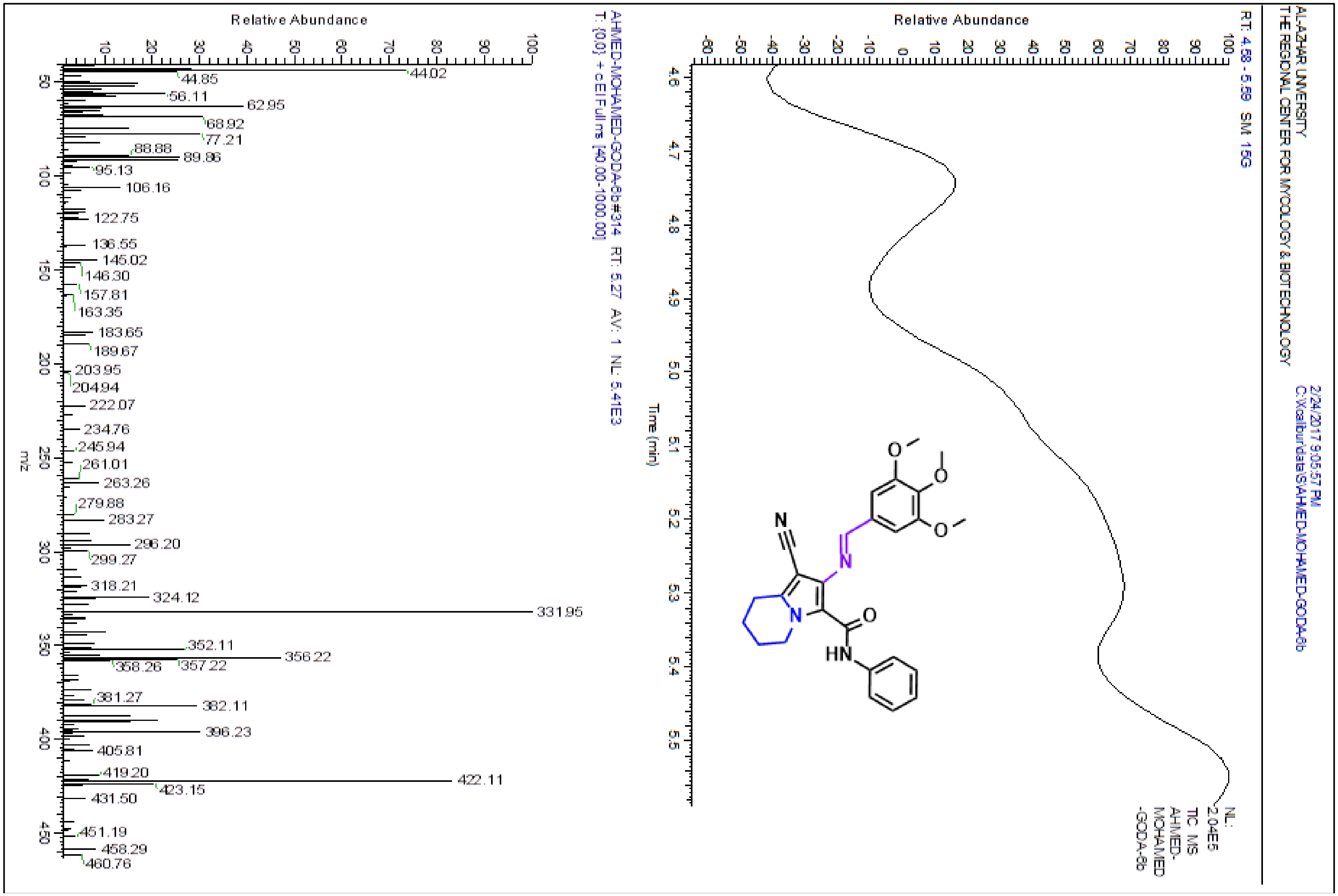

Fig. S126. **Mass spectrum of compound 16a**

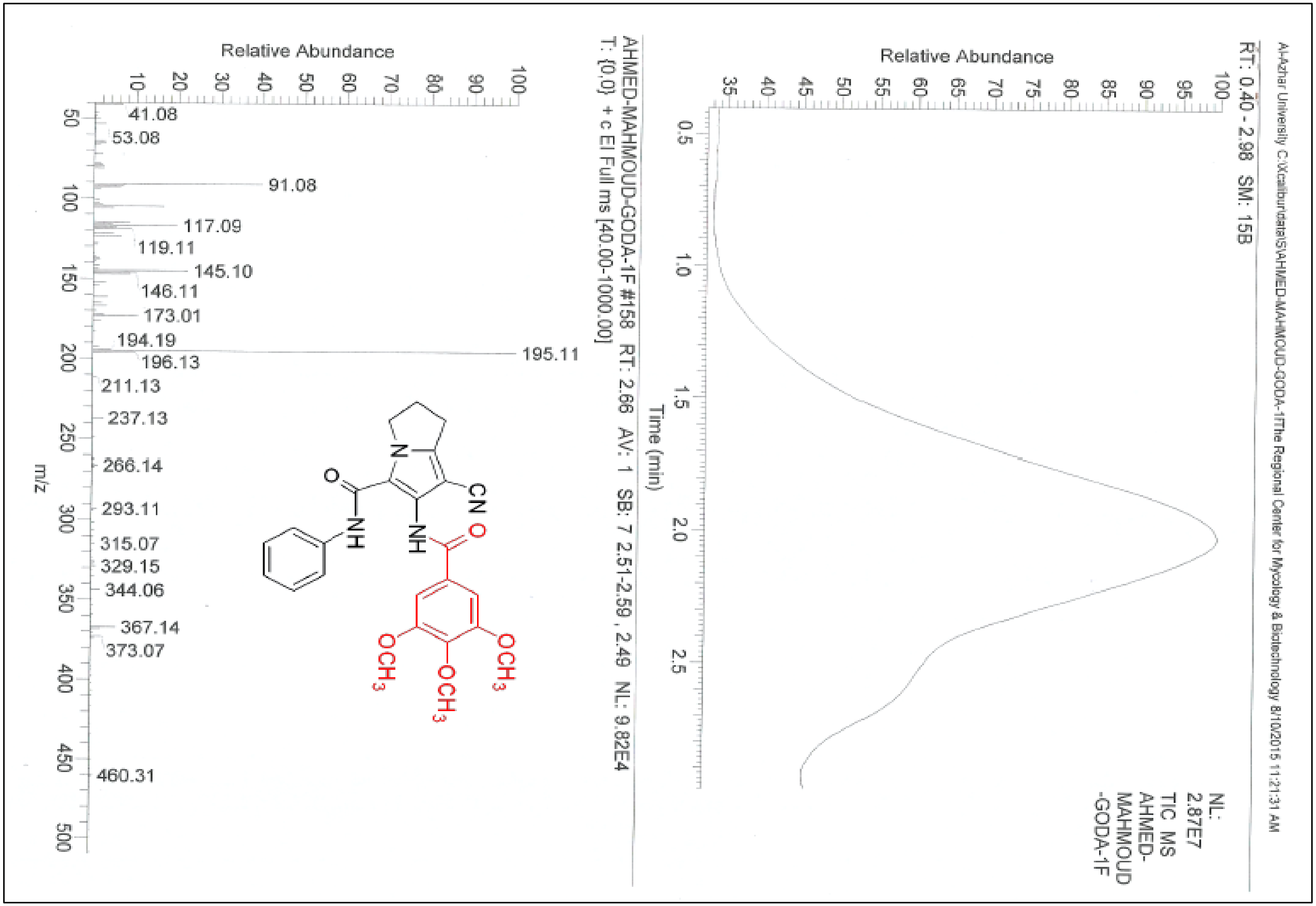

Fig. S127. Mass spectrum of compound 16a (continued)

Al-Azhar University C:\Xcalibur\data\S\AHMED-MAHMOUD-GODA-1F The Regional Center for Mycology & Biotechnology 8/10/2015 11:21:31 AM

AHMED-MAHMOUD-GODA-1F#158 RT: 2.66
SB: 7 2.51-2.59 , 2.49
T: {0,0} + c EI Full ms [40.00-1000.00]
m/z= 40.00-509.44

m/z	Intensity	Relative
41.08	6536.0	6.66
46.04	740.3	0.75
48.00	561.2	0.57
50.05	1229.0	1.25
53.08	2563.7	2.61
64.04	2373.0	2.42
65.07	2542.5	2.59
66.07	1301.0	1.33
72.08	1760.5	1.79
78.08	1544.7	1.57
79.08	1905.6	1.94
80.06	2309.0	2.35
81.09	2205.0	2.25
91.08	39140.5	39.87
92.08	6843.9	6.97
93.09	6238.9	6.35
94.06	1442.0	1.47
101.10	1208.8	1.23
103.09	1158.4	1.18
104.10	2045.4	2.08
105.10	16289.5	16.59
106.11	4528.5	4.61
115.09	8367.6	8.52
116.11	4277.0	4.36
117.09	19295.8	19.65
118.12	5330.4	5.43
119.11	8703.4	8.87
122.09	4572.6	4.66
124.09	6549.9	6.67
128.10	1046.0	1.07
129.09	921.4	0.94
136.12	573.3	0.58
137.08	1374.5	1.40
138.09	1432.7	1.46
139.08	575.2	0.59
142.11	1296.5	1.32
144.11	1120.3	1.14
145.10	21932.0	22.34
146.11	9686.9	9.87
147.14	8667.6	8.83
152.10	2927.8	2.98
154.12	1288.8	1.31
161.17	3421.8	3.49
165.16	2032.6	2.07
167.10	3303.4	3.36
170.07	803.7	0.82
172.07	2485.3	2.53
173.01	10388.3	10.58
176.13	1823.4	1.86
183.12	588.3	0.60
192.13	1354.6	1.38

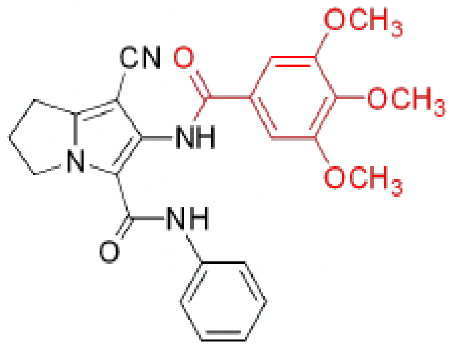

Fig. S128. Mass spectrum of compound 16a (continued)

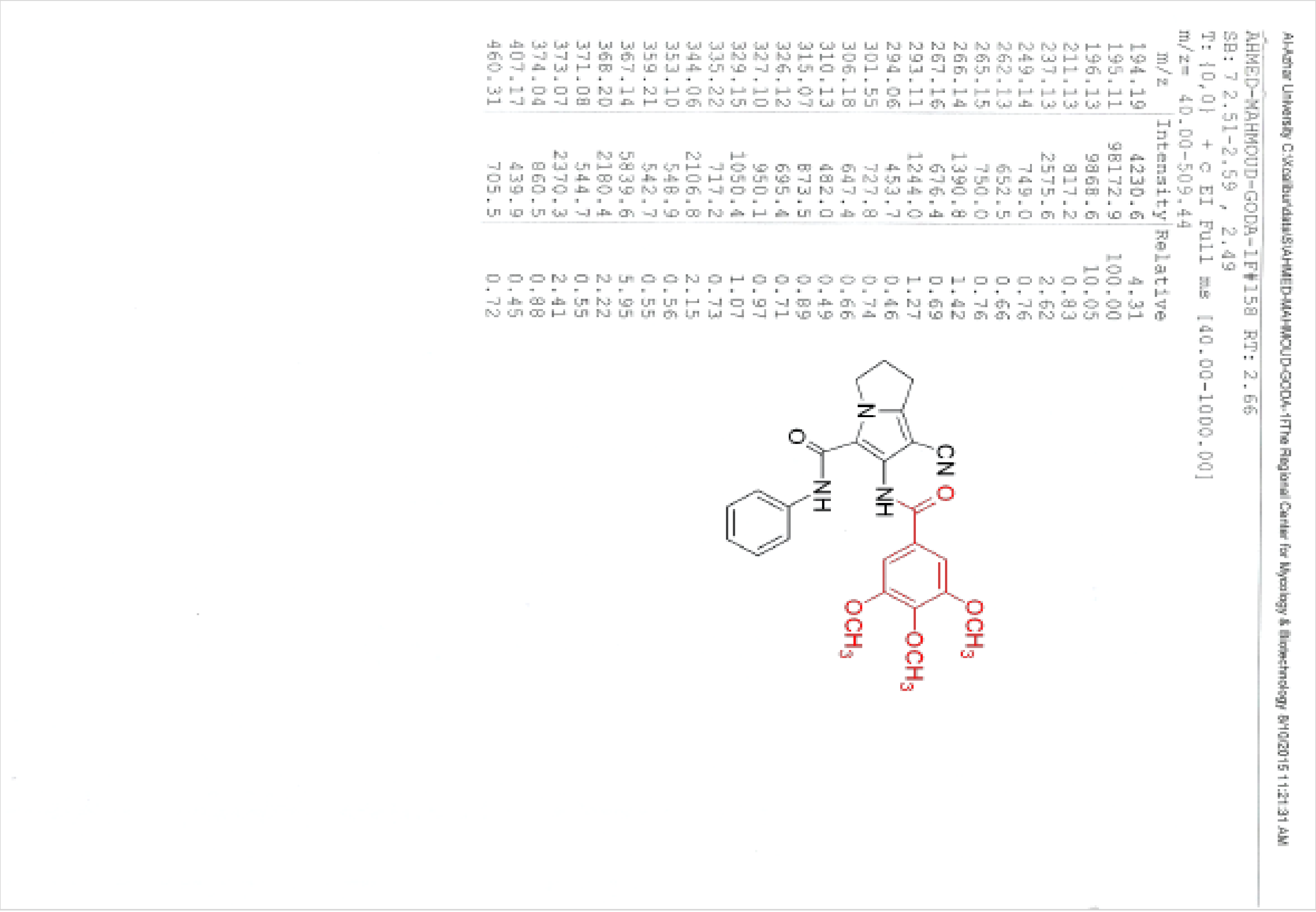

Al-Azhar University C:\Xcalibur\data\S\AHMED-MAHMOUD-GODA-1F The Regional Center for Mycology & Biotechnology 8/10/2015 11:21:31 AM

AHMED-MAHMOUD-GODA-1F#158 RT: 2.66
SB: 7 2.51-2.59 , 2.49
T: {0,0} + c EI Full ms [40.00-1000.00]
m/z= 40.00-509.44

m/z	Intensity	Relative
194.19	4230.6	4.31
195.11	98172.9	100.00
196.13	9868.6	10.05
211.13	817.2	0.83
237.13	2575.6	2.62
249.14	749.0	0.76
262.13	652.5	0.66
265.15	750.0	0.76
266.14	1390.8	1.42
267.16	676.4	0.69
293.11	1244.0	1.27
294.06	453.7	0.46
301.55	727.8	0.74
306.18	647.4	0.66
310.13	482.0	0.49
315.07	873.5	0.89
326.12	695.4	0.71
327.10	950.1	0.97
329.15	1050.4	1.07
335.22	717.2	0.73
344.06	2106.8	2.15
353.10	548.9	0.56
359.21	542.7	0.55
367.14	5839.6	5.95
368.20	2180.4	2.22
371.08	544.7	0.55
373.07	2370.3	2.41
374.04	860.5	0.88
407.17	439.9	0.45
460.31	705.5	0.72

Fig. S129. **Mass spectrum of compound 16b**

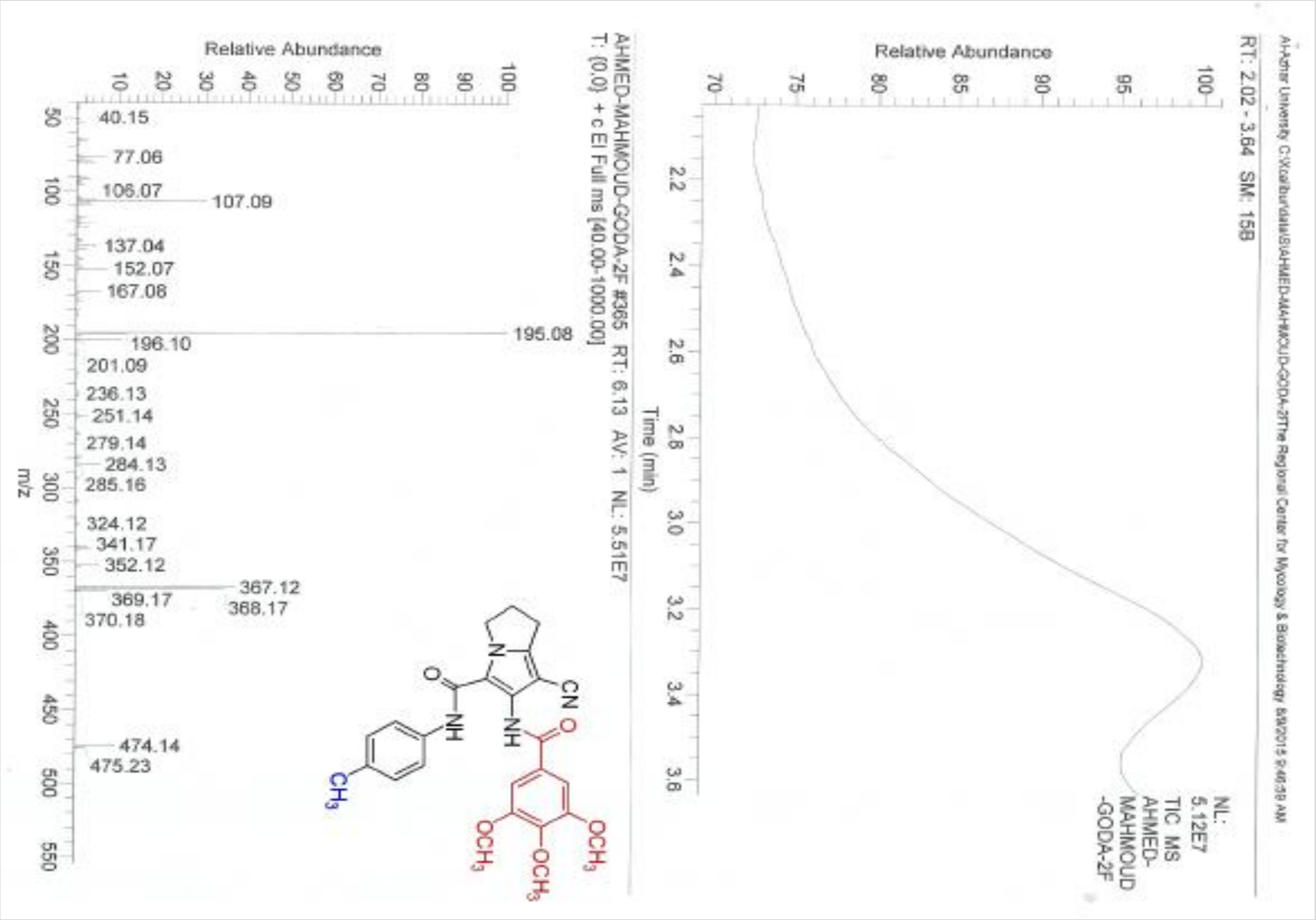

Fig. S130. **Mass spectrum of compound 16b (continued)**

Al-Azhar University C:\Xcalibur\data\S\AHMED-MAHMOUD-GODA-2F The Regional Center for Mycology & Biotechnology 8/9/2015 9:46:59 AM

AHMED-MAHMOUD-GODA-2F#365 RT: 6.13

T: {0,0} + c EI Full ms [40.00-1000.00]

m/z= 40.00-554.10

m/z	Intensity	Relative
40.15	2090644.5	3.80
41.06	484306.2	0.88
44.03	407800.2	0.74
53.05	1019444.7	1.85
63.04	396584.4	0.72
64.05	486893.0	0.88
65.06	1356561.3	2.46
66.04	1328052.6	2.41
67.07	412432.3	0.75
76.07	638280.3	1.16
77.06	3887970.3	7.06
78.07	1063419.0	1.93
79.06	1930000.9	3.50
81.05	2477855.8	4.50
90.08	464143.3	0.84
91.07	1346430.0	2.45
92.07	1855537.6	3.37
93.06	672374.3	1.22
94.06	577372.1	1.05
95.07	547072.7	0.99
96.07	1047420.0	1.90
104.06	618378.4	1.12
105.11	646926.8	1.17
106.07	2489072.3	4.52
107.09	16563391.0	30.08
108.10	1712643.3	3.11
109.06	1966440.8	3.57
117.06	1233788.4	2.24
118.07	494852.8	0.90
119.10	662969.5	1.20
120.02	476185.8	0.86
121.08	489784.8	0.89
122.06	2189740.8	3.98
123.07	538408.4	0.98
124.08	1526422.0	2.77
132.07	471049.4	0.86
133.10	635886.1	1.15
134.04	882863.6	1.60
137.04	2806025.3	5.10
139.10	1306868.5	2.37
145.08	390479.7	0.71
146.09	847821.4	1.54
150.07	496844.8	0.90
151.09	1021583.9	1.86
152.07	4004013.5	7.27
153.09	525807.9	0.95
167.08	3135536.8	5.69
168.12	399440.2	0.73
172.05	437862.6	0.80
174.08	960017.1	1.74
179.06	397382.8	0.72
181.08	407435.6	0.74

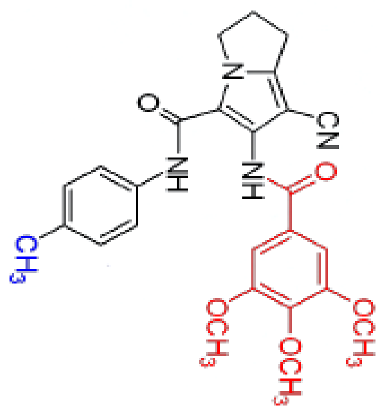

Fig. S131. **Mass spectrum of compound 16b (continued)**

Al-Azhar University C:\Xcalibur\data\S\AHMED-MAHMOUD-GODA-2fThe Regional Center for Mycology & Biotechnology 8/9/2015 9:46:59 AM

AHMED-MAHMOUD-GODA-2F#365 RT: 6.13
T: {0,0} + c EI Full ms [40.00-1000.00]
m/z= 40.00-554.10

m/z	Intensity	Relative
195.08	55066052.0	100.00
196.10	6212096.5	11.28
197.12	803533.5	1.46
200.06	5877819.5	10.67
201.09	666671.4	1.21
236.13	590760.2	1.07
251.14	1462311.0	2.66
252.15	468682.4	0.85
262.15	408817.1	0.74
263.15	597918.4	1.09
279.14	637921.1	1.16
280.14	505084.8	0.92
284.13	3141086.0	5.70
285.16	569444.4	1.03
294.09	422323.1	0.77
324.12	730753.9	1.33
340.17	1606699.0	2.92
341.17	1970292.0	3.58
342.17	426056.8	0.77
352.12	3092407.3	5.62
353.14	685817.9	1.25
367.12	20371146.0	36.99
368.17	18883552.0	34.29
369.17	3932880.3	7.14
370.18	547031.1	0.99
474.14	5064290.5	9.20
475.23	1339881.3	2.43

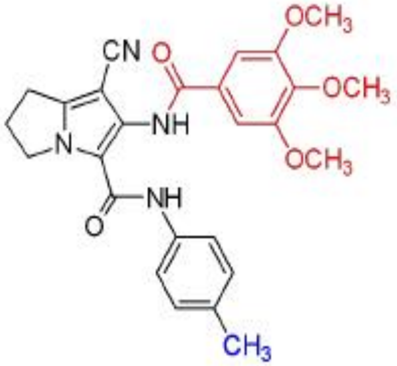

Fig. S132. **Mass spectrum of compound 16c.**

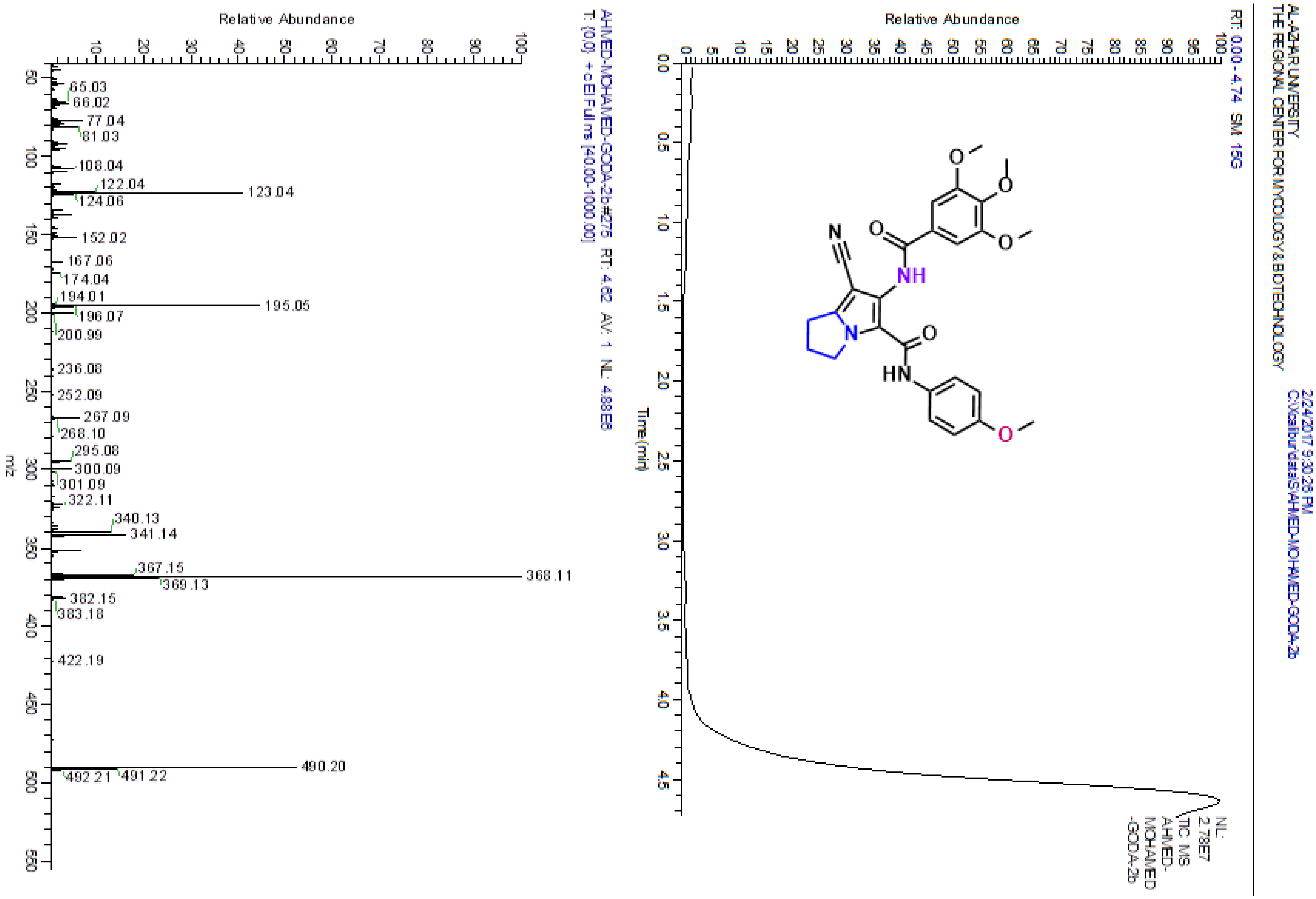

Fig. S133. **Mass spectrum of compound 16d.**

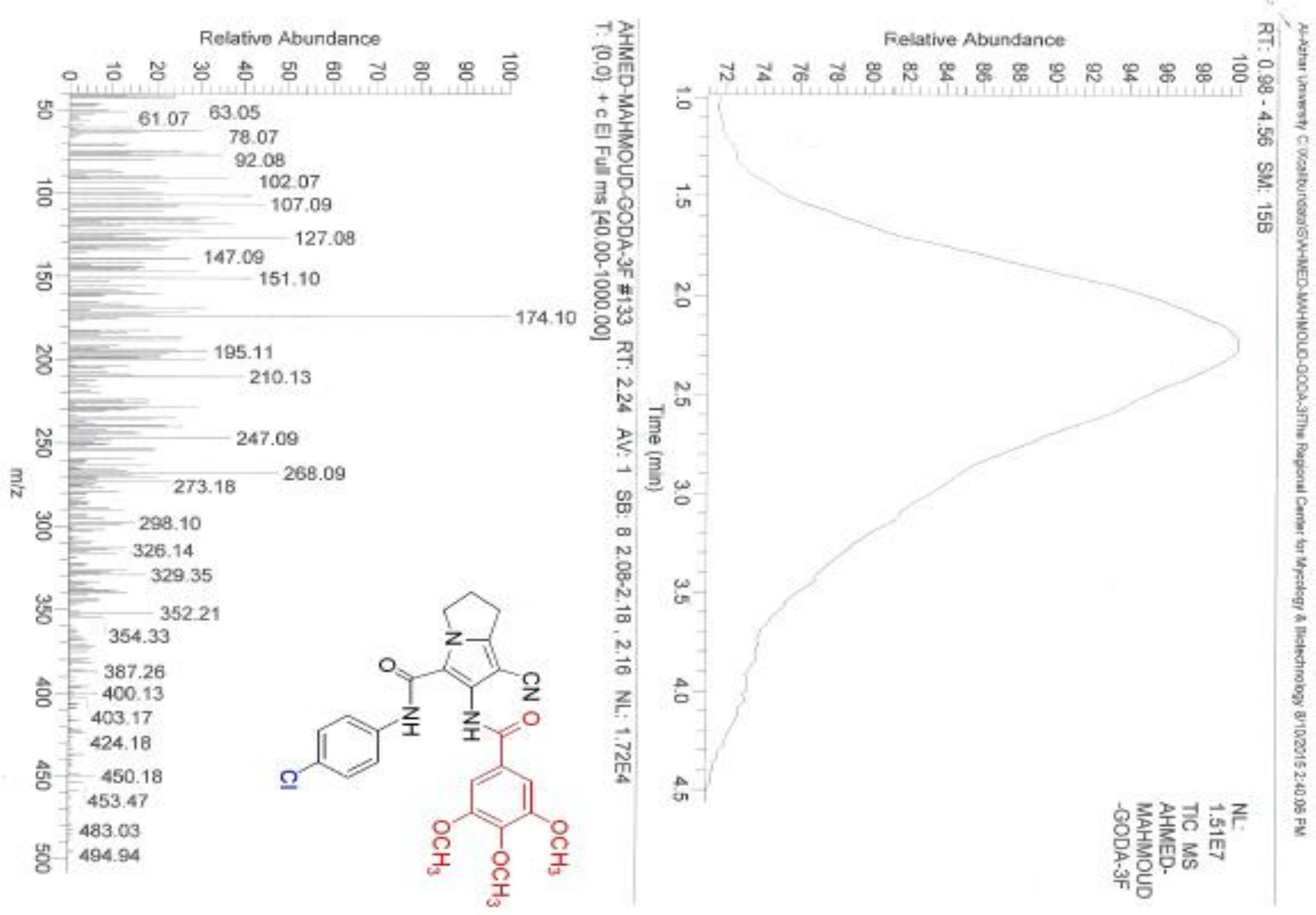

Fig. S134. Mass spectrum of compound **16d** (continued)

Al-Azhar University C:\Xcalibur\data\S\AHMED-MAHMOUD-GODA-3F The Regional Center for Mycology & Biotechnology 8/10/2015 2:40:06 PM

AHMED-MAHMOUD-GODA-3F#133 RT: 2.24
SB: 8 2.08-2.18 , 2.16
T: {0,0} + c EI Full ms [40.00-1000.00]
m/z= 40.00-508.30

m/z	Intensity	Relative
41.08	4171.2	24.29
42.08	2188.8	12.74
43.10	4127.2	24.03
46.08	1130.2	6.58
47.00	1255.4	7.31
48.04	977.7	5.69
50.07	1473.2	8.58
51.07	2241.9	13.05
53.07	981.1	5.71
54.10	331.7	1.93
55.08	335.5	1.95
56.10	815.9	4.75
59.10	526.4	3.07
61.07	2488.8	14.49
62.06	260.7	1.52
63.05	5226.2	30.43
64.06	2751.1	16.02
65.07	430.2	2.50
67.10	222.2	1.29
71.11	2290.4	13.34
72.15	2118.6	12.34
75.05	4338.3	25.26
76.06	5364.3	31.24
77.07	2377.4	13.84
78.07	5975.5	34.79
80.08	3356.1	19.54
86.09	1672.5	9.74
88.08	2066.0	12.03
89.09	661.2	3.85
90.08	3612.1	21.03
91.08	768.0	4.47
92.08	6237.9	36.32
94.09	3879.2	22.59
98.12	2908.0	16.93
100.11	3683.9	21.45
102.07	7151.2	41.64
104.07	3612.8	21.04
107.09	7618.9	44.36
108.08	4340.7	25.28
109.08	4176.6	24.32
111.09	3686.6	21.47
112.10	196.3	1.14
115.08	5724.3	33.33
116.09	5183.0	30.18
117.08	4930.6	28.71
118.08	1280.9	7.46
119.08	6340.2	36.92
120.10	2090.6	12.17
121.09	240.9	1.40
123.09	5311.3	30.93
125.10	2922.9	17.02

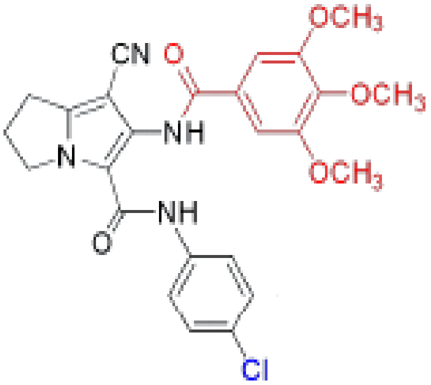

Fig. S135. Mass spectrum of compound 16d (continued)

Al-Azhar University C:\Xcalibur\data\S\AHMED-MAHMOUD-GODA-3F The Regional Center for Mycology & Biotechnology 8/10/2015 2:40:06 PM

AHMED-MAHMOUD-GODA-3F#133 RT: 2.24
SB: 8 2.08-2.18 , 2.16
T: {0,0} + c EI Full ms [40.00-1000.00]
m/z= 40.00-508.30

m/z	Intensity	Relative
126.11	1782.0	10.38
127.08	8556.6	49.82
128.09	4227.7	24.62
129.09	3838.4	22.35
131.09	3181.9	18.53
132.09	3894.4	22.68
133.09	1124.3	6.55
134.08	3681.5	21.44
136.11	697.4	4.06
139.06	4773.1	27.79
140.09	4686.1	27.29
142.09	2916.8	16.98
143.09	863.7	5.03
144.09	2889.4	16.82
145.09	2989.2	17.41
146.09	2642.8	15.39
147.09	4999.1	29.11
151.10	7162.5	41.71
153.08	1558.4	9.07
156.06	3007.3	17.51
158.08	2017.0	11.74
159.07	2538.6	14.78
160.11	3547.6	20.66
161.10	1241.1	7.23
162.10	1436.3	8.36
165.11	1160.8	6.76
166.11	2117.8	12.33
168.12	2887.2	16.81
169.08	5319.3	30.97
170.08	785.6	4.57
171.12	4607.2	26.83
173.07	3314.9	19.30
174.10	17173.9	100.00
175.12	1935.1	11.27
176.13	583.4	3.40
182.14	3176.3	18.49
183.13	1192.0	6.94
184.09	2290.1	13.33
185.15	1240.0	7.22
186.13	4512.7	26.28
188.11	4366.7	25.43
191.12	1968.4	11.46
192.17	1556.6	9.06
193.15	1065.9	6.21
194.14	4259.4	24.80
195.11	5403.9	31.47
196.13	3913.3	22.79
197.09	3598.1	20.95
198.12	2730.9	15.90
199.09	3560.0	20.73
200.11	5338.2	31.08

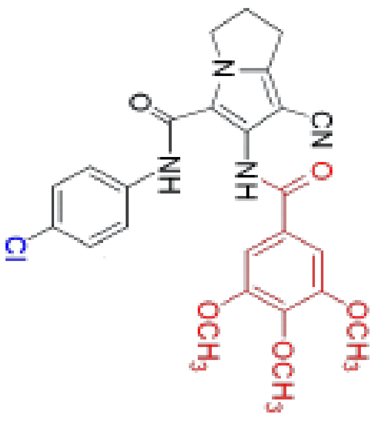

Fig. S136. Mass spectrum of compound 16d (continued)

Al-Azhar University C:\Xcalibur\data\S\AHMED-MAHMOUD-GODA-3f The Regional Center for Mycology & Biotechnology 8/10/2016 2:40:06 PM

AHMED-MAHMOUD-GODA-3F#133 RT: 2.24
SB: 8 2.08-2.18 , 2.16
T: {0,0} + c EI Full ms [40.00-1000.00]
m/z= 40.00-508.30

m/z	Intensity	Relative
201.13	160.1	0.93
203.12	2336.9	13.61
204.13	300.0	1.75
205.12	1398.7	8.14
207.14	1512.9	8.81
209.14	2445.6	14.24
210.13	6831.8	39.78
211.14	246.0	1.43
212.11	1068.6	6.22
214.13	244.4	1.42
215.10	732.8	4.27
216.12	1243.1	7.24
218.12	839.2	4.89
220.15	1260.3	7.34
224.12	3148.2	18.33
225.11	2077.6	12.10
226.11	3113.9	18.13
227.15	243.1	1.42
228.12	5071.2	29.53
229.10	2727.7	15.88
230.10	2320.2	13.51
231.13	1440.9	8.39
233.13	273.6	1.59
234.12	4199.2	24.45
236.14	3303.6	19.24
237.16	670.9	3.91
238.11	1031.4	6.01
239.13	3809.5	22.18
240.09	4493.4	26.16
241.09	369.0	2.15
242.10	1483.2	8.64
243.11	2586.9	15.06
244.08	2863.2	16.67
245.11	1065.6	6.20
247.09	6339.6	36.91
248.13	1658.6	9.66
249.13	961.1	5.60
250.13	1618.6	9.43
251.13	1450.3	8.44
252.10	672.0	3.91
253.11	3319.4	19.33
254.12	3400.2	19.80
259.09	2504.9	14.59
260.10	652.9	3.80
261.11	731.0	4.26
262.12	603.8	3.52
263.12	3084.4	17.96
264.11	321.3	1.87
265.51	488.8	2.85
266.07	1871.9	10.90
267.16	2319.8	13.51

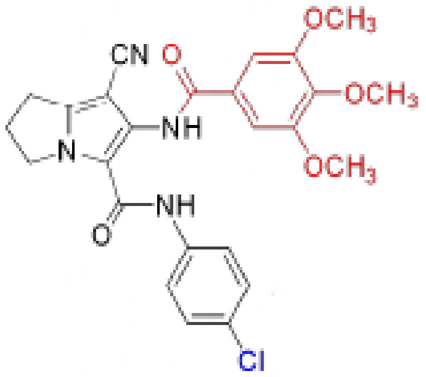

Fig. S137. **Mass spectrum of compound 16d (continued)**

Al-Azhar University C:\Xcalibur\data\S\AHMED-MAHMOUD-GODA-3F The Regional Center for Mycology & Biotechnology 8/10/2015 2:40:06 PM

AHMED-MAHMOUD-GODA-3F#133 RT: 2.24
SB: 8 2.08-2.18 , 2.16
T: {0,0} + c EI Full ms [40.00-1000.00]
m/z= 40.00-508.30

m/z	Intensity	Relative
268.09	8187.3	47.67
269.10	2536.5	14.77
270.06	1089.5	6.34
271.10	3464.7	20.17
272.10	1102.2	6.42
273.18	3898.7	22.70
274.09	1090.5	6.35
275.11	920.0	5.36
276.08	532.1	3.10
277.07	1364.2	7.94
279.09	1957.5	11.40
280.12	677.6	3.95
281.07	733.4	4.27
283.10	645.4	3.76
284.09	442.7	2.58
285.56	834.3	4.86
286.12	709.0	4.13
287.03	770.6	4.49
288.11	276.6	1.61
289.10	1600.8	9.32
290.06	2182.8	12.71
293.11	751.3	4.37
294.13	732.4	4.26
295.15	1390.7	8.10
298.10	2547.8	14.84
299.10	2091.9	12.18
300.12	288.4	1.68
301.06	123.2	0.72
302.07	1451.5	8.45
303.08	944.1	5.50
306.10	220.1	1.28
307.11	474.3	2.76
308.08	134.9	0.79
309.02	969.9	5.65
310.08	885.6	5.16
311.09	292.0	1.70
313.16	2268.7	13.21
314.17	1500.0	8.73
315.09	778.1	4.53
316.14	1898.8	11.06
318.43	293.7	1.71
319.19	328.4	1.91
322.20	745.3	4.34
323.07	571.3	3.33
324.06	638.6	3.72
325.13	124.0	0.72
326.14	2285.3	13.31
327.12	1625.2	9.46
328.12	1239.9	7.22
329.35	2993.4	17.43
330.10	286.7	1.67

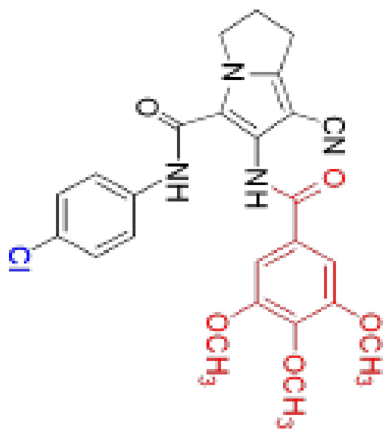

Fig. S138. **Mass spectrum of compound 16d (continued)**

Al-Azhar University C:\Xcalibur\data\S\AHMED-MAHMOUD-GODA-3F The Regional Center for Mycology & Biotechnology 8/10/2015 2:40:06 PM

AHMED-MAHMOUD-GODA-3F#133 RT: 2.24
SB: 8 2.08-2.18 , 2.16
T: (0,0) + c EI Full ms [40.00-1000.00]
m/z= 40.00-508.30

m/z	Intensity	Relative
333.02	596.1	3.47
334.06	1224.5	7.13
335.09	744.6	4.34
336.07	345.4	2.01
337.09	964.5	5.62
338.13	1644.3	9.57
339.10	1899.3	11.06
340.07	2327.7	13.55
341.15	758.3	4.42
343.10	1234.4	7.19
345.11	1151.0	6.70
346.10	534.2	3.11
347.08	250.3	1.46
351.23	435.4	2.54
352.21	3353.5	19.53
353.09	421.4	2.45
354.33	1351.8	7.87
360.12	183.1	1.07
362.47	344.8	2.01
363.14	374.0	2.18
364.11	321.6	1.87
366.13	443.6	2.58
367.04	570.0	3.32
368.05	288.0	1.68
368.56	678.8	3.95
371.08	688.0	4.01
372.08	1043.7	6.08
373.13	878.5	5.12
375.12	775.4	4.51
379.05	812.6	4.73
381.05	543.4	3.16
382.18	914.5	5.32
386.18	671.6	3.91
387.26	1174.3	6.84
391.06	295.7	1.72
393.21	217.8	1.27
396.15	813.7	4.74
397.03	482.5	2.81
399.19	208.7	1.22
400.13	1121.6	6.53
401.10	278.0	1.62
403.17	681.6	3.97
406.23	288.1	1.68
406.99	437.4	2.55
408.65	354.3	2.06
409.49	300.6	1.75
416.05	199.5	1.16
416.68	416.4	2.42
421.44	130.3	0.76
422.16	440.5	2.56
423.12	512.8	2.99

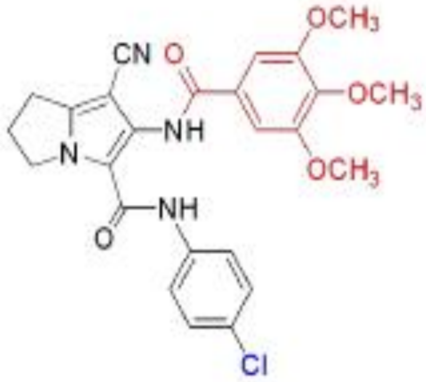

Fig. S139. Mass spectrum of compound 16d (continued)

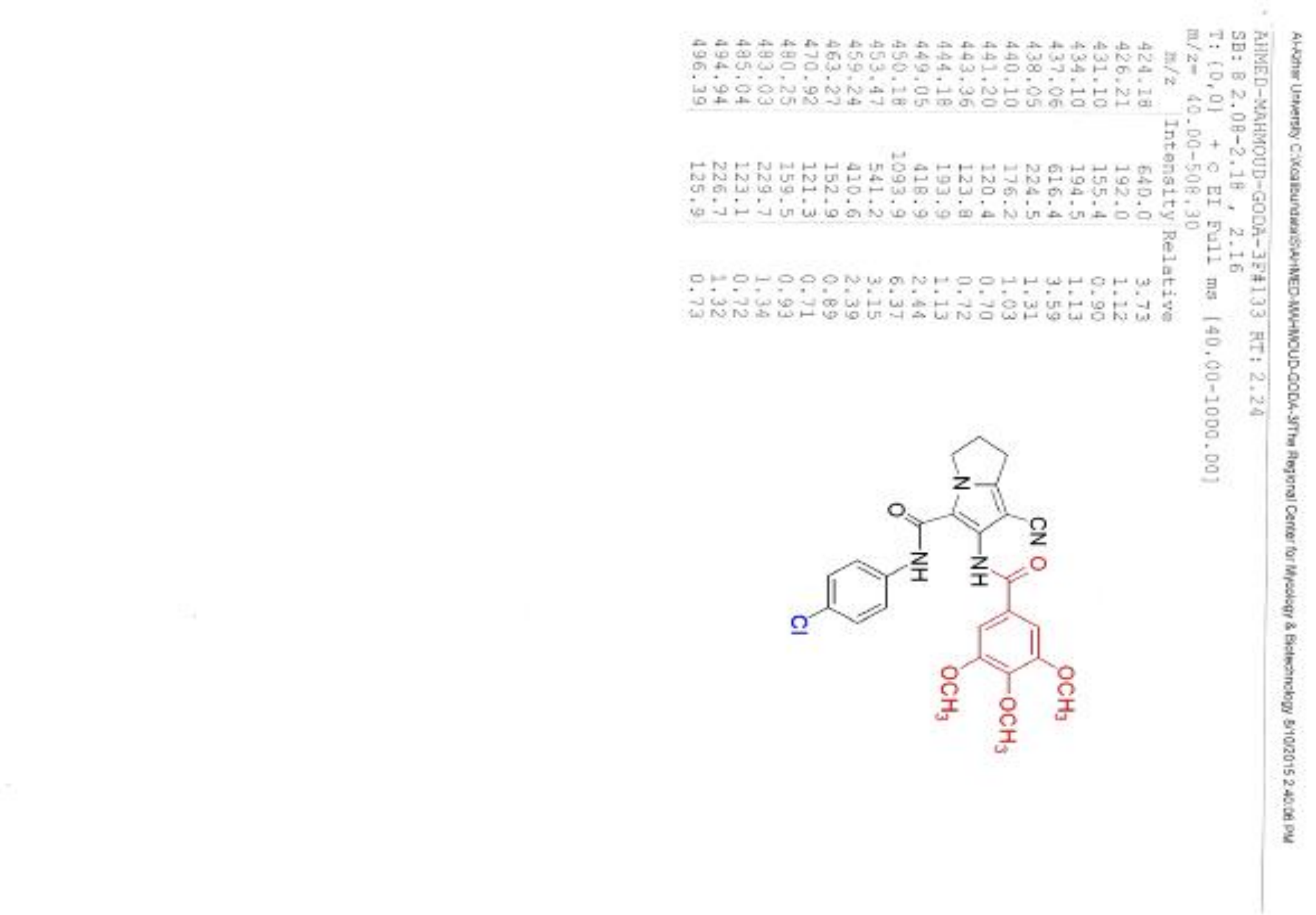
Al-Azhar University C:\Xcalibur\data\SA\AHMED-MAHMOUD-GODA-3\The Regional Center for Mycology & Biotechnology 8/10/2015 2:40:06 PM

AHMED-MAHMOUD-GODA-3F#133 RT: 2.24
SB: 8 2.08-2.18 , 2.16
T: {0,0} + c EI Full ms [40.00-1000.00]
m/z= 40.00-508.30

m/z	Intensity	Relative
424.18	640.0	3.73
426.21	192.0	1.12
431.10	155.4	0.90
434.10	194.5	1.13
437.06	616.4	3.59
438.05	224.5	1.31
440.10	176.2	1.03
441.20	120.4	0.70
443.36	123.8	0.72
444.18	193.9	1.13
449.05	418.9	2.44
450.18	1093.9	6.37
453.47	541.2	3.15
459.24	410.6	2.39
463.27	152.9	0.89
470.92	121.3	0.71
480.25	159.5	0.93
493.03	229.7	1.34
485.04	123.1	0.72
494.94	226.7	1.32
496.39	125.9	0.73

Fig. S140. Mass spectrum of compound **16e**

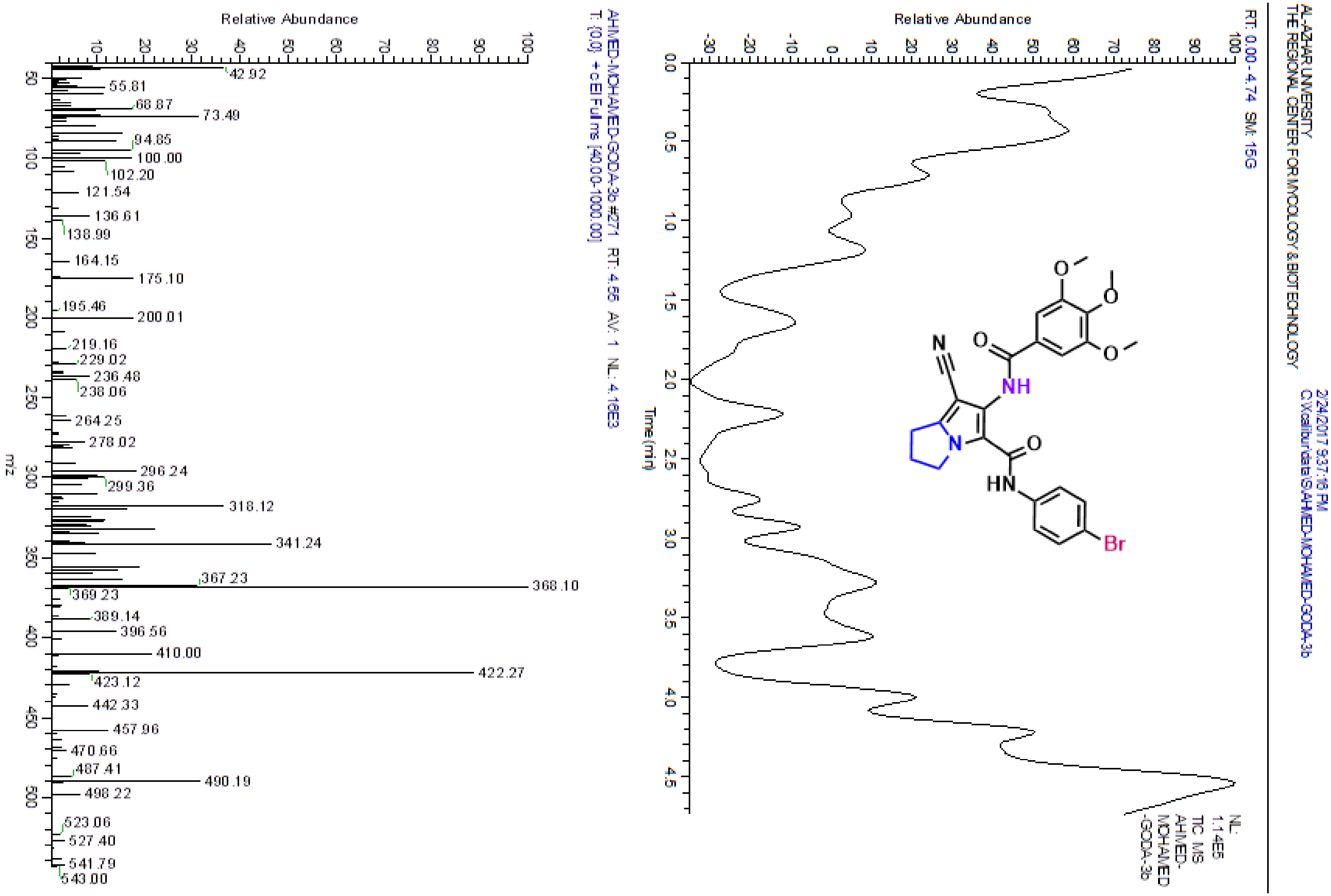

Fig. S141. Mass spectrum of compound 21

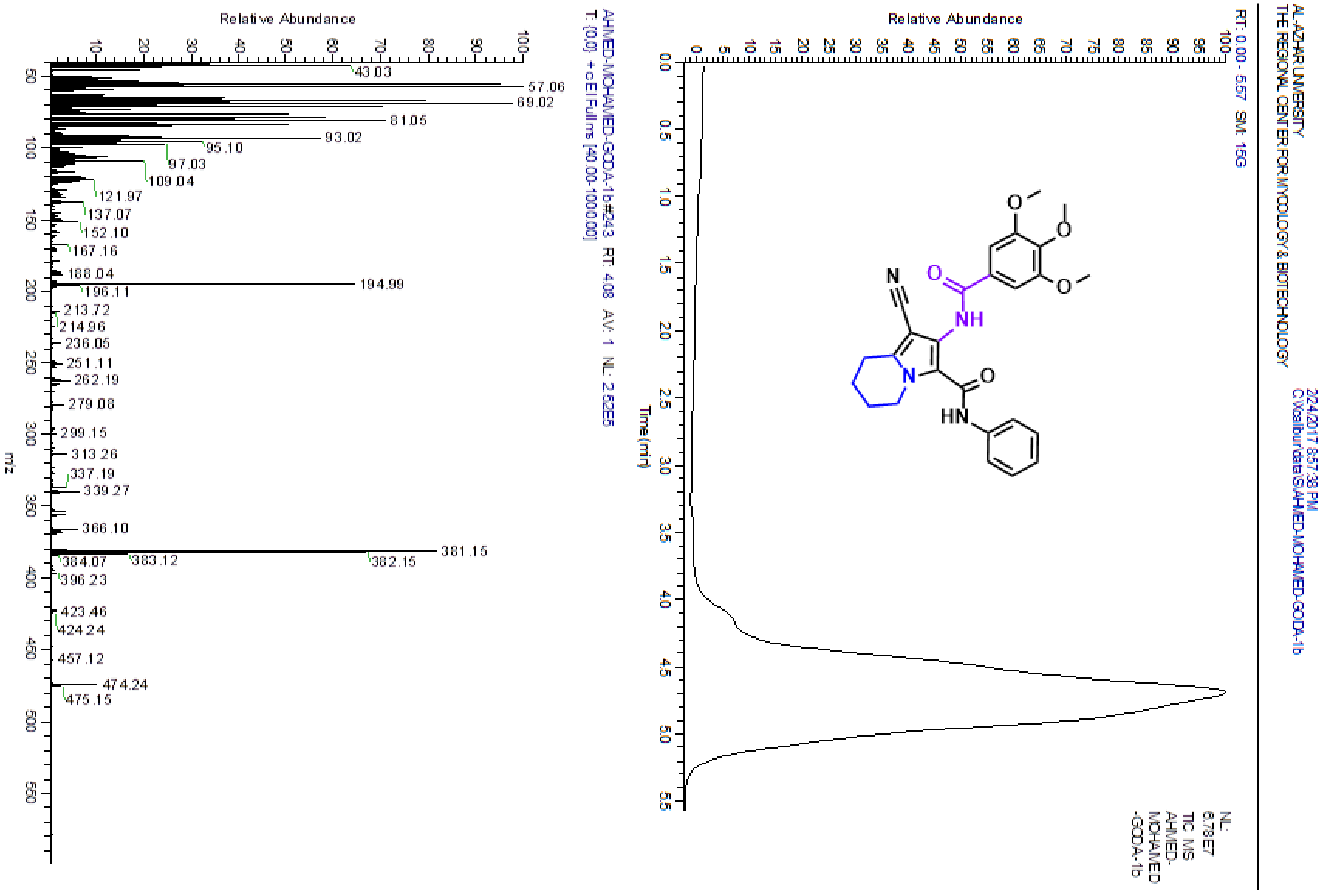

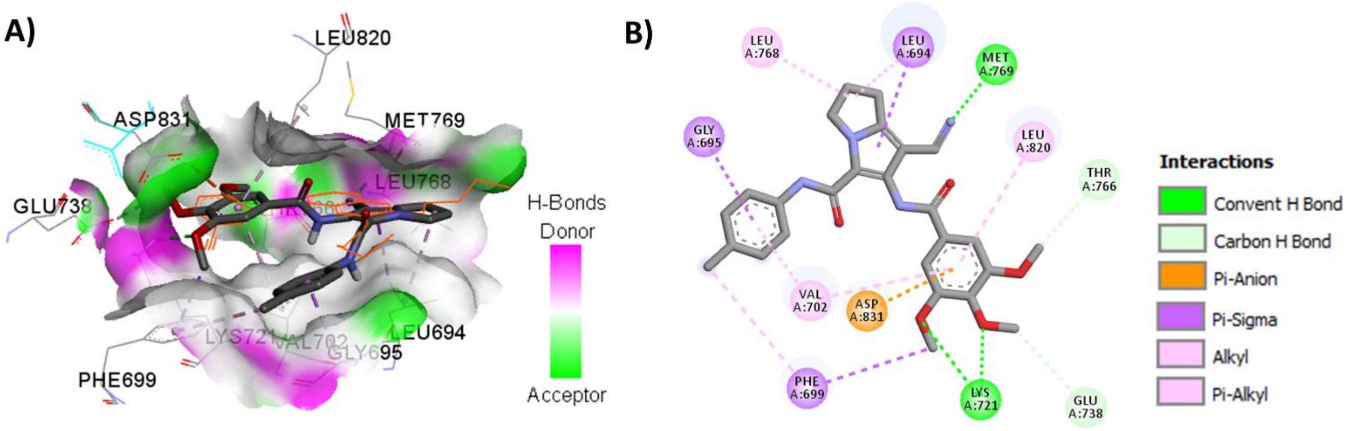

Fig. S142. Binding modes/interactions of compound **16b** into EGFR (PDB code: 1M17): A) 3D binding mode of compound **16b** into the active site of EGFR, the co-crystallized erlotinib shown as orang line, receptor shown as hydrogen bond surface, hydrogen atoms were omitted for clarity; B) 2D binding mode of compound **16b** into EGFR showing different types of interactions with amino acids in the active site of the protein, hydrogen atoms were omitted for clarity.

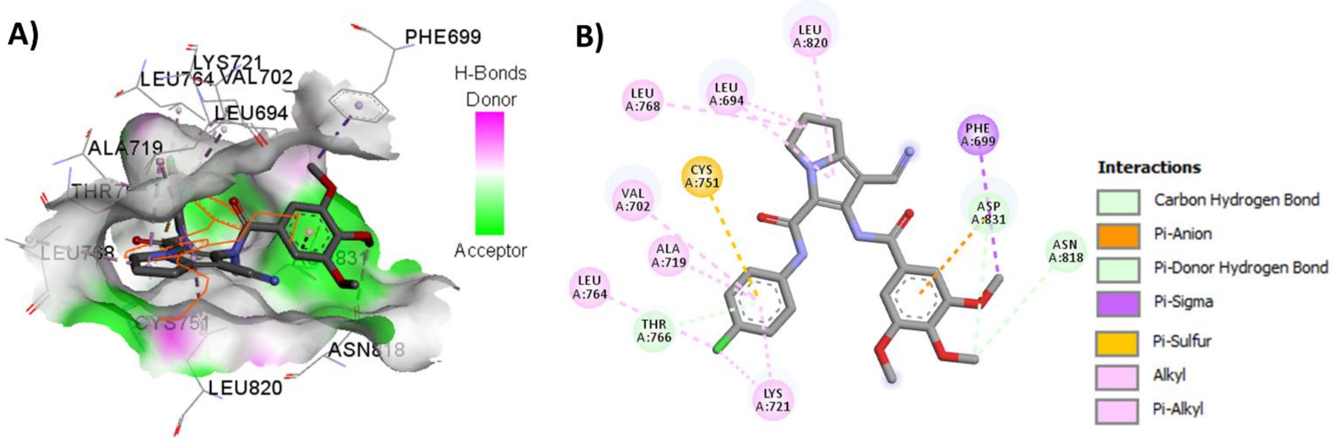

Fig. S143. Binding modes/interactions of compound **16d** into EGFR (PDB code: 1M17): A) 3D binding mode of compound **16d** into the active site of EGFR, the co-crystallized erlotinib shown as orang line, receptor shown as hydrogen bond surface, hydrogen atoms were omitted for clarity; B) 2D binding mode of compound **16d** into EGFR showing different types of interactions with amino acids in the active site of the protein, hydrogen atoms were omitted for clarity.

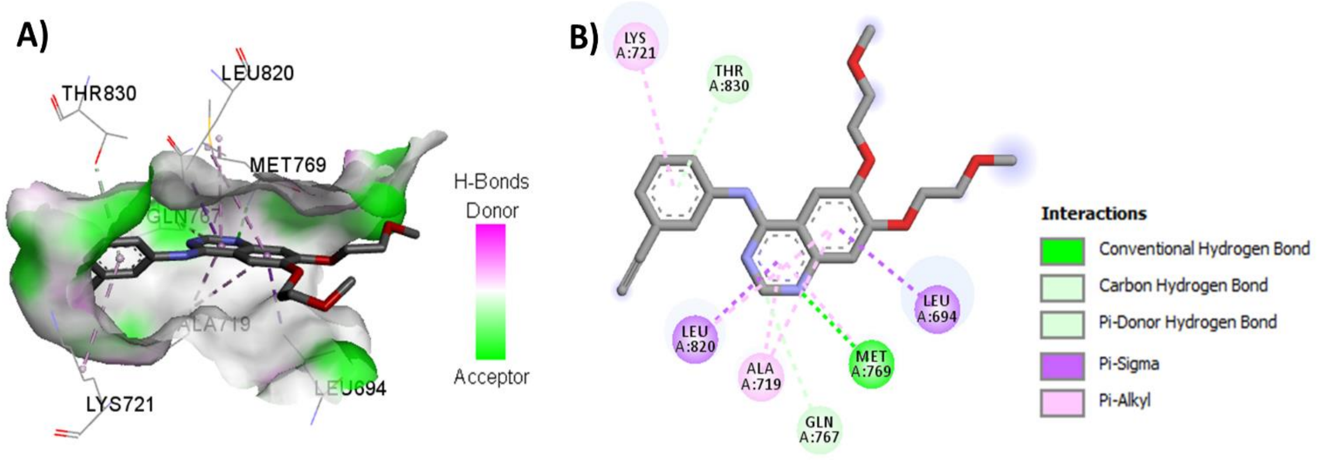

Fig. S144. Binding modes/interactions of erlotinib into EGFR (PDB code: 1M17): A) 3D binding mode of erlotinib into the active site of EGFR, receptor shown as hydrogen bond surface, hydrogen atoms were omitted for clarity; B) 2D binding mode of erlotinib into EGFR showing different types of interactions with amino acids in the active site of the protein, hydrogen atoms were omitted for clarity.

Table S3. Docking results of compounds **9a**, **16a**,**b**,**d** into Aurora A kinase in comparison to the native ligand, VX6.

**PK** (pdb)	**Ligand**	ΔG_b [a]	K_i [b]
**Aurora A** (3E5A)	**16a**	-9.37	134.65 nM
	**16b**	-9.65	84.31 nM
	**16d**	-9.74	72.01 nM
	**9a**	-8.81	346.04 nM
	**VX6** [c]	-8.49	602.62 nM

[a] Binding free energy (kcal/mol).
[b] Inhibition constant (μ/nM).
[c] VX6, *N*-(4-((4-((3-methyl-1*H*-pyrazol-5-yl)amino)-6-(4-methylpiperazin-1-yl)pyrimidin-2-yl)thio)phenyl)cyclopropane-carboxamide.

Table S4. Docking results of compounds **9a**, **15a-e**, **16a-e**, **20**, **21** into tubulin (pdb: 5LYJ)

**Comp. No**	ΔG_b [a]	K_i [b]	**HBs** [c]	**Atoms in H-bonding**		**Length** [e] (Å)
				**In ligands**	**In tubulin**	
**15c**	-6.63	13.83 µM	2	4-CH_3O	NH_2 of ASN258	2.80
				C=O	NH of ALA317	2.42
			2*	3-CH_3O	LEU248, ASN249	2.00, 2.22
**15e**	-8.07	1.21 µM	1	CN	NH of ALA354	2.85
**20**	-5.88	49.29 µM	2	4-CH_3O	NH_2 of ASN258	2.86
				C=O	NH of ALA317	2.08
			3*	3/4-CH_3O	LEU248, ASN249, ASN101	1.83-2.26
**16c**	-9.08	222.49 nM	2	7- CN	NH of ASP251	2.11
				PhNHC=O	NH_2 of LYS352	1.60
			5*	3/4-CH_3O, CH_2-3	CYS241, GLY237, VAL238, VLA315, ALA316, THR179	1.68-2.84
**16e**	-11.87	1.99 nM	2	C=O	NH_2 of LYS352	1.48
				PhNHC=O	C=O of THR179	2.11
			5*	3/4-CH_3O, CH_2-3	CYS241, GLY237, VAL238, ALA316, THR179	1.68-2.84
**21**	-12.16	1.23 nM	1	PhNHC=O	NH_2 of LYS352	1.84
			4*	3/4-CH_3O, CH_2-3	CYS241, GLY237, LYS352, THR376	1.91-2.60
**9a**	-8.72	407.88 nM	1	OH	C=O of THR179	2.18
			3*	CH_3O groups	VAL238, VAL315, and ASN350	1.93-2.75

[a] *Binding free energy (kcal/mol);* [b] *Inhibition constant (µ/nM);* [c]*HBs, number of hydrogen bonds;* [d] *No hydrogen bond detected;* [e] *length in angstrom (Å);* compound 9, 7BA (CA4); values with asterisks indicated carbon hydrogen bonds, the underlines atoms are the atoms involved in H-bond (acceptor and donner)

List of abbreviations:

ADP, adenosine diphosphate; ADR, adriamycin; ADT, AutoDock tools; ALK1, activin receptor-like kinase 1; AMPK, 5'adenosine monophosphate-activated protein kinase; ASK1, apoptosis signal-regulating kinase 1; ATCC, American type culture collection; BLK, B-lymphoid tyrosine kinase; CA-4, combretastatin A-4; CDK2, cyclin-dependent kinase 2; CK1, casein kinase 1; 2/3D, two/three dimensional; DAPI, 4′,6-diamidino-2-phenylindole; DEPT, distortionless enhancement by polarization transfer; DMEM , Dulbecco's modified eagle's medium; DMSO, dimethyl sulfoxide; DYRK3, dual specificity tyrosine phosphorylation-regulated kinase; EGFR, epidermal growth factor receptor; EGTA, ethylene glycol-bis(2-aminoethylether)-N,N,N',N'-tetraacetic acid; EI, electron impact; EMEM, Eagle's minimum essential medium; eV, electron volte; FBS, fetal bovine serum; FITC, fluorescein isothiocyanate; FLT1, fms-related tyrosine kinase 1 (vascular endothelial growth factor/vascular permeability factor receptor); FTIR, Fourier-transform infrared spectroscopy; GSK3 alpha, glycogen synthase kinase 3 alpha; GTP, guanosine-5'-triphosphate; HDAC, histone deacetylases; IC_{50}, half maximal inhibitory concentration; IR, infrared; K_i, inhibition constants; MAPK, mitogen-activated protein kinase; MSK1, mitogen- and stress-activated kinases 1; MTT, 3-(4,5-dimethylthiazol-2-yl)-2,5-diphenyl-2H-tetrazolium bromide; NEK1, NIMA (never in mitosis gene a)-related kinase 1; OD, optical density; p38 Alpha, mitogen-activated protein kinase 14; PDGFR-β, platelet-derived growth factor receptor β; PDK1, PDK1 pyruvate dehydrogenase kinase 1; PI, propidium iodide; PKs, protein kinases; RFU, relative fluorescence units; RPMI-1640, Roswell Park Memorial Institute-1640 medium; SAR, structure activity relationship; SGK1, serum/glucocorticoid regulated kinase 1; TLC, thin-layer chromatography; VEGFR2, vascular endothelial growth factor receptor 2.
